# Supplementary material for: Lanatoside C activates the E3 ligase STUB1 to inhibit FOXP3 transcriptional activity and promote antitumor immunity
Source: EMBO Mol Med. 2025 Feb 20;17(3):563–88. doi: 10.1038/s44321-025-00200-y (PMC11904033; doi:10.1038/s44321-025-00200-y)
Supplement: Supplementary file 3 — Appendix [file 44321_2025_200_MOESM3_ESM.pdf]

Table of Contents

1. Appendix Fig.S1. High-throughput screening of drugs capable of inhibiting luciferase activity of 293T-NF+RC. (page 2-page 3)

2. Appendix Fig.S2. Impact of Lac on differentiation of Tregs. (page 4)

3. Appendix Fig.S3. Lac does not affect transcription of RUNX1. (page 5-page 6)

4. Appendix Fig.S4. Lac rewires tumor microenvironment by inhibiting function of Tregs. (page 7)

5. Appendix Fig.S5. Lac synergizes with PD-1 inhibitor to treat mutant KRAS driven lung cancer. (page 8-page 9)

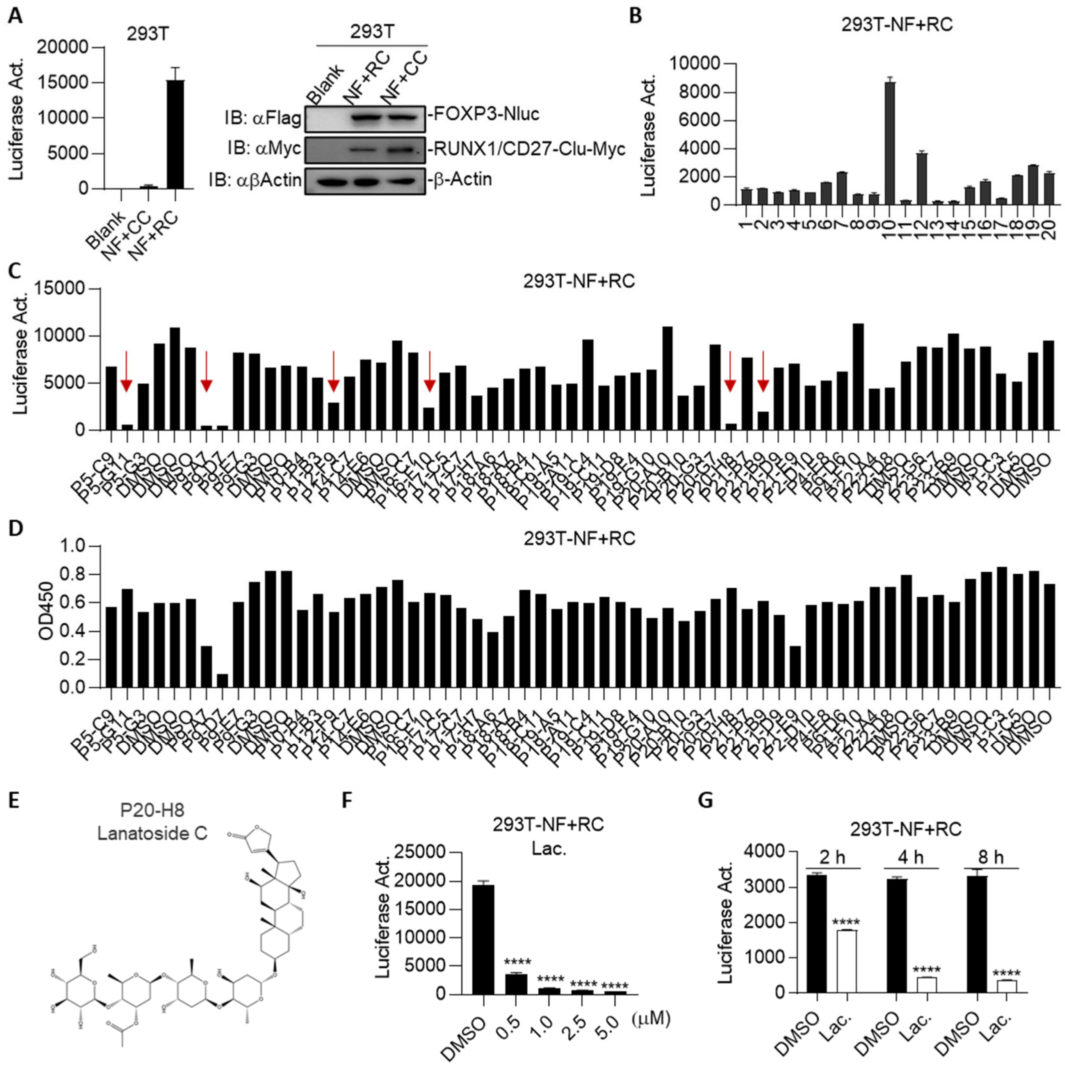

Appendix Fig. S1. High-throughput screening of drugs capable of inhibiting luciferase activity of 293T-NF+RC

A: Bimolecular fluorescence complementation assay. 293T cells (1×10^5) were co-transfected with FOXP3 fused N-terminal half of firefly luciferase (Nlu-FOXP3-Flag) and RUNX1 fused C-terminal half of firefly luciferase (RUNX1-Clu-Myc) (designated NF+RC) or FOXP3 fused N-terminal half of firefly luciferase and CD27 fused C-terminal half of firefly luciferase (designated NF+CC), respectively. 36 hours after transfection, luciferase assay (left panel) and IB (right panel) were performed.

B: Generation of monoclonal of 293T-NF+RC for high-throughput screening of drugs. Monoclone (Nb. 10) was selected through limiting dilution and designated 293T-NF+RC.

C: Validation screening of candidate compounds capable of inhibiting luciferase activity in 293T-NF+RC.

D: Impact of candidate compounds on 293T-NF+RC cell viability.

E: Chemical structure of Lac (No. P20-H8).

F: Impact of Lac on 293T-NF+RC luciferase activity. Data are representative of three independent experiments. $^{****}P < 0.0001$ by Student's *t*-test. Error bars denote mean ± SD.

G: Lac suppresses luciferase activity of 293T-NF+RC in time dependent manner. Data are representative of three independent experiments. $^{****}P < 0.0001$ by Student's *t*-test. Error bars denote mean ± SD.

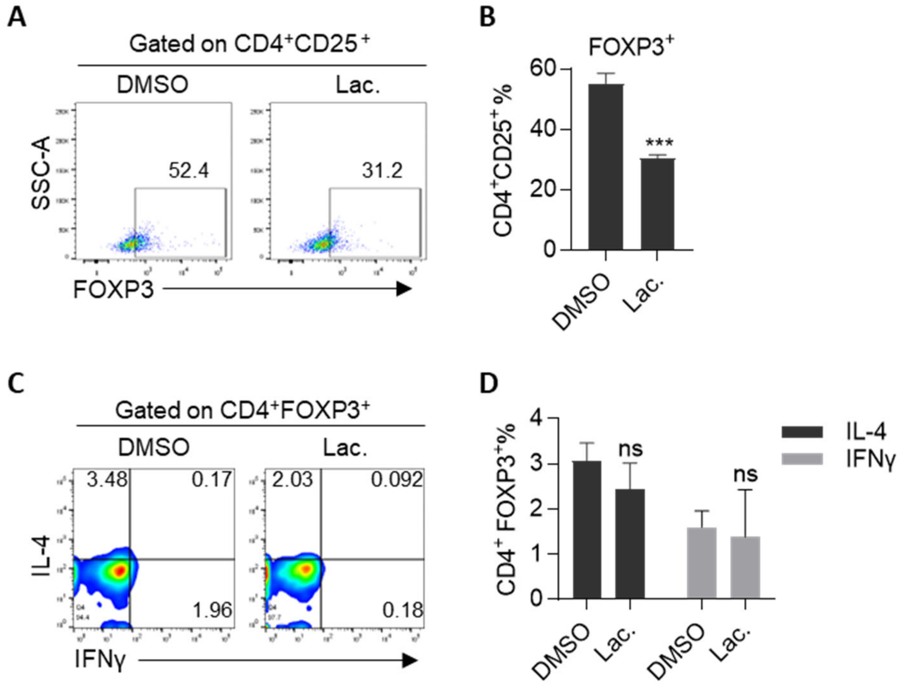

Appendix Fig. S2. Impact of Lac on differentiation of Tregs

A: Lac suppresses FOXP3 expression in Tregs. FOXP3 expression was detected by flow cytometry.

B: Statistics analysis FOXP3 expression of Appendix Fig. S2A. Data are representative of three independent experiments. $^{***}P = 0.0003$ by Student's *t*-test. Error bars denote mean ± SD.

C: Impact of Lac on the differentiation of Tregs to TH1 or TH2.

D: Statistics of IL-4 or IFNγ expression of Appendix Fig. S2C. Data are representative of three independent experiments and were analyzed by Student's *t*-test. Error bars denote mean ± SD. *P* value: $FOXP3^{+}IL\text{-}4^{+}$, $P = 0.2002$; $FOXP3^{+}IFN\gamma^{+}$, $P = 0.7587$.

Data are representative of three independent experiments and were analyzed by unpaired t-test. Error bars denote SD. $^{***}P < 0.001$.

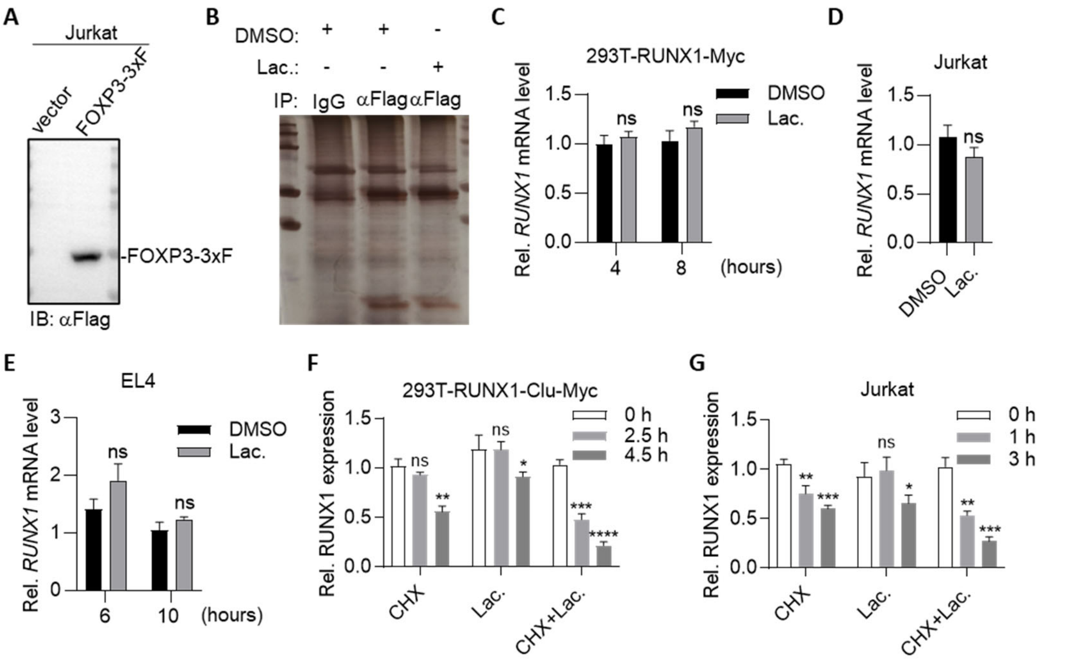

Appendix Fig. S3. Lac does not affect transcription of RUNX1

A: Generation of Jurkat-FOXP3-3×FLAG stable cell line.

B: Silver staining of SDS-PAGE separated immunoprecipitants enriched from Jurkat-FOXP3-3×FLAG cells with indicated antibodies.

C-E: Lac has no impact on transcription of *RUNX1*. 293T-RUNX1-Myc (Appendix Fig. S3C), Jurkat (Appendix Fig. S3D) or EL4 (Appendix Fig. S3E) cells were treated with DMSO or Lac for indicated time points before qPCR analysis. Data are representative of three independent experiments and were analyzed by Student's *t*-test. Error bars denote mean ± SD. *P* value: 4h, $P = 0.2832$, 8h, $P = 0.1091$ for 293T-RUNX1-Myc; $P = 0.2587$ for Jurkat; 6h, $P = 0.0631$, 10h, $P = 0.0841$ for EL4.

F,G: Relative changes in the half-life of RUNX1. RUNX1 protein level was normalized against β-Actin and quantified using ImageJ software. Statistics analysis of relative RUNX1 expression of Fig. 3G (Appendix Fig. S3F) and Fig.3H (Appendix Fig. S3G). Data are representative of three independent experiments and were

analyzed by Student's *t*-test. Error bars denote mean ± SD. *P* value: CHX (0h vs 2.5h), $P = 0.1471$, CHX (0h vs 4.5h), $^{**}P = 0.0012$, Lac. (0h vs 2.5h), >0.9999, Lac. (0h vs 4.5h), $^{*}P = 0.0374$, CHX+Lac. (0h vs 2.5h), $^{***}P = 0.0002$, CHX+Lac. (0h vs 4.5h), $^{****}P < 0.0001$ for 293T-RUNX1-Clu-Myc; CHX (0h vs1h), $^{**}P = 0.0059$, CHX (0h vs 3h), $^{***}P = 0.0002$, Lac. (0h vs 1h), $P = 0.6177$, Lac. (0h vs 3h), $^{*}P = 0.0422$, CHX+Lac. (0h vs 1h), $^{**}P = 0.0012$, CHX+Lac. (0h vs 3h), $^{***}P = 0.0002$ for Jurkat.

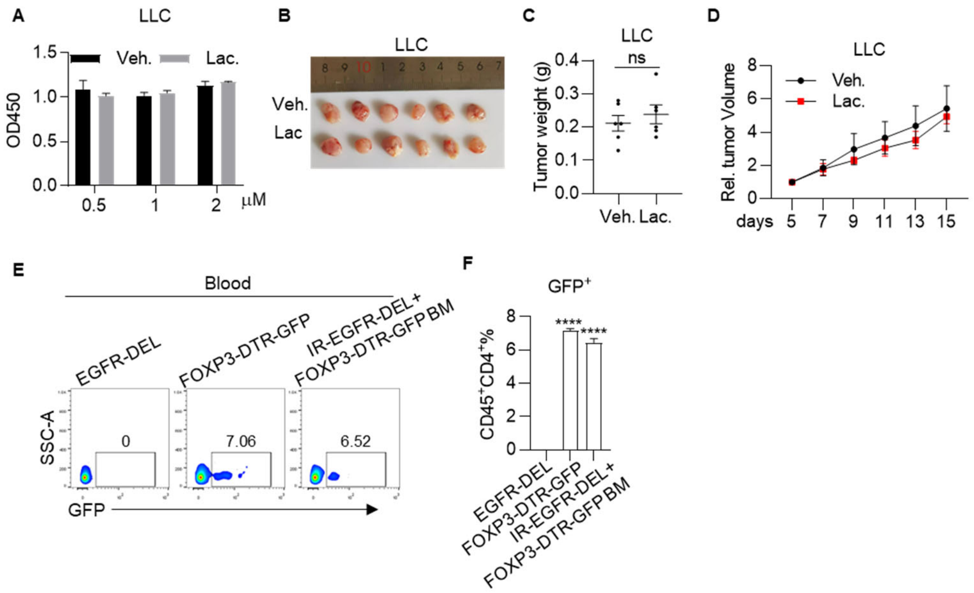

Appendix Fig. S4. Lac rewires tumor microenvironment by inhibiting function of Tregs

A: Lac is not toxic to LLC cell *in vitro*. Data are representative of three independent experiments and were analyzed by Student's *t*-test. Error bars denote mean ± SD. *P* value: 0.5 μM, P = 0.1894; 1 μM, P = 0.1697; 2 μM, P = 0.1178.

B-D: Lac exhibits no treatment effect on LLC allograft tumor *in vivo*. Mice were then randomized for treatment (n=6) by PBS or Lac for two weeks. The allografts (n=6) were dissected to photograph (B), weigh (C) by the end of experiments. Tumor growth (D) was recorded every 2 days. Values represent the mean ± SD by Student's *t*-test. *P* value: tumor weight, P=0.4839; tumor volume, P=0.4258.

E: Flow cytometry analysis GFP^+ population in blood from indicated mice. Representative flow plots shown for $CD45^+CD4^+$ population.

F: Statistics of GFP^+ population of Appendix Fig. S4E. Data are representative of three independent experiments and were analyzed by Student's *t*-test. Error bars denote mean ± SD. $^{****}P < 0.0001$ by Student's *t*-test.

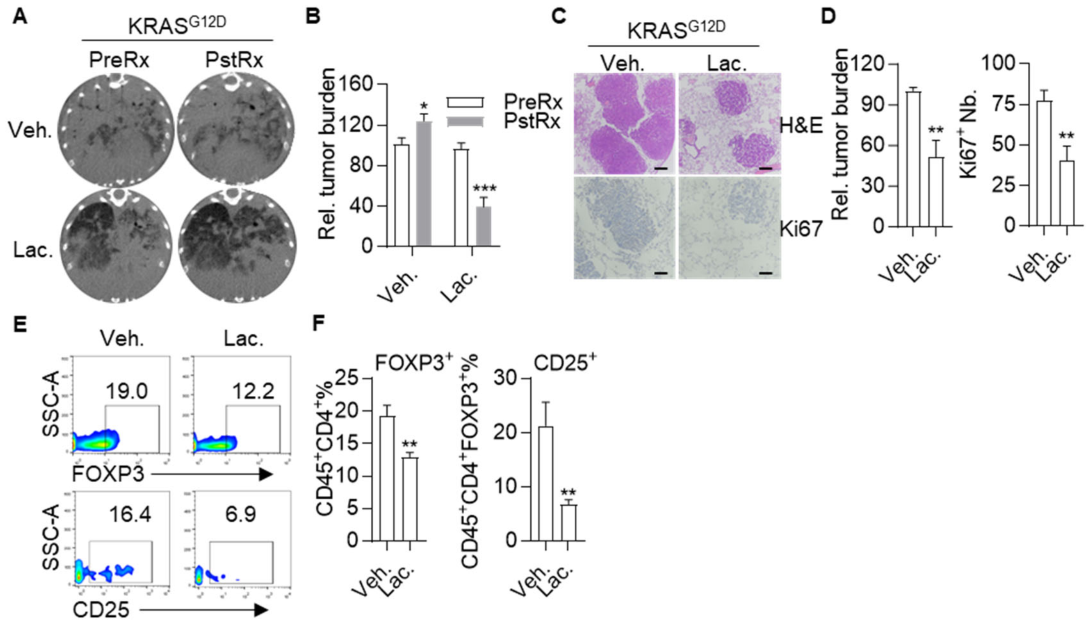

Appendix Fig. S5. Lac synergizes with PD-1 inhibitor to treat mutant KRAS driven lung cancer

A: Lac effectively shrinks lung tumors in $KRAS^{G12D}$ mice. Tumor burdens (n=3) were monitored through CT scanning before and two-week after treatment.

B: Statistics of relative tumor burden of Appendix Fig. S5A. Data are representative of three independent experiments and were analyzed by Student's *t*-test. Error bars denote mean ± SD. *P* value: Veh, $^{*}P$=0.0124; Lac, $^{***}P$=0.0009.

C: Representative images of H&E (upper panel) or Ki67 (lower panel) staining of the lung tissues of treated mice. Scale bar: 200 μm.

D: Statistics of relative tumor burden (left panel) or Ki67 positive cells (right panel) of Appendix Fig. S5C. Data are representative of three independent experiments and were analyzed by Student's *t*-test. Error bars denote mean ± SD. *P* value: Rel.tumor burden, $^{**}P$=0.0028; $Ki67^{+}$, $^{**}P$=0.0044.

E: Lac suppresses expression of FOXP3 and CD25 of tumor infiltrated Tregs *in vivo*.

F: Statistics of the proportion of $FOXP3^+$ (left panel) and $CD25^+$ (right panel) of Tregs in tumor of Appendix Fig. S5E. Data are representative of three independent experiments and were analyzed by Student's *t*-test. Error bars denote mean ± SD. *P* value: $FOXP3^+$, $^{**}P$=0.0031; $CD25^+$, $^{**}P$=0.0046.

Data are representative of three independent experiments and were analyzed by unpaired t-test. Error bars denote SD. $^{*}P < 0.05$, $^{**}P < 0.01$, $^{***}P < 0.001$.
