# Supplementary figures and images for: Lanatoside C activates the E3 ligase STUB1 to inhibit FOXP3 transcriptional activity and promote antitumor immunity (part 1 of 2)
Source: EMBO Mol Med. 2025 Feb 20;17(3):563–88. doi: 10.1038/s44321-025-00200-y (PMC11904033; doi:10.1038/s44321-025-00200-y)

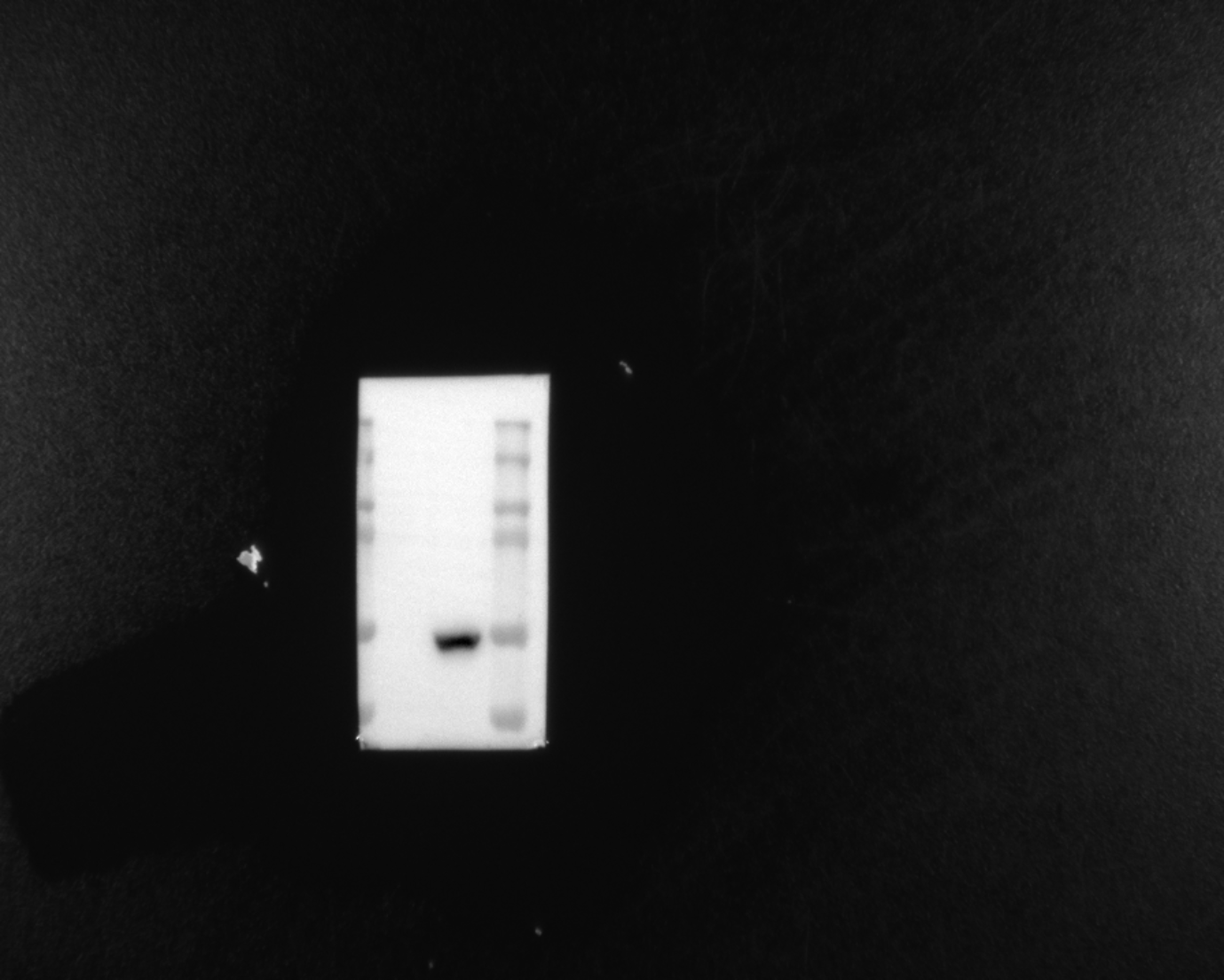

Supplement: Supplementary file 8 — EV Figure and Appendix Figure Source Data [file 44321_2025_200_MOESM8_ESM.zip › Appendix Fig. S3/Appendix Fig. S3A/Jurkat-3xFlag.tif]

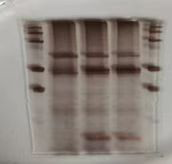

Supplement: Supplementary file 8 — EV Figure and Appendix Figure Source Data [file 44321_2025_200_MOESM8_ESM.zip › Appendix Fig. S3/Appendix Fig. S3B/IP-Flag.tif]

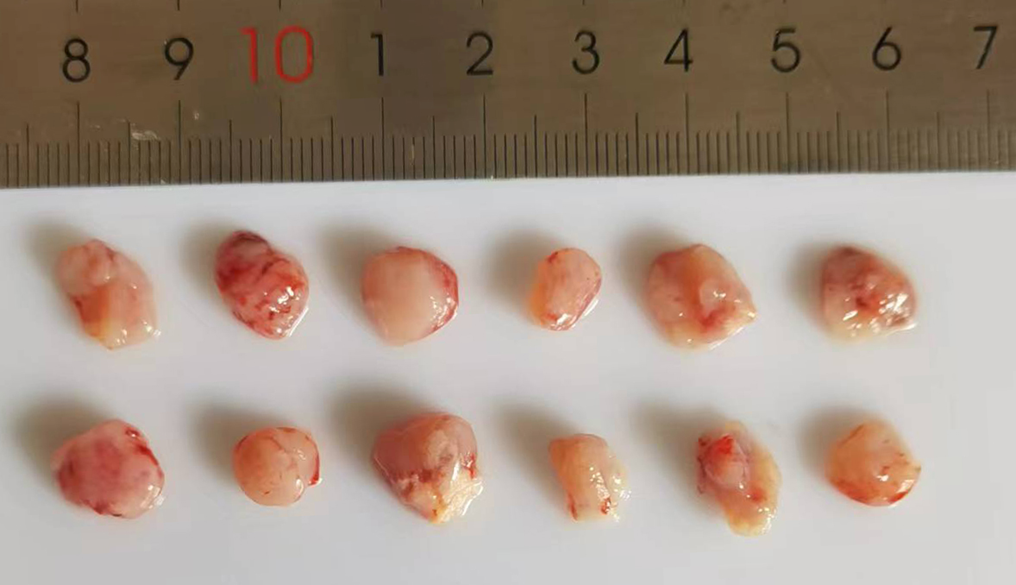

Supplement: Supplementary file 8 — EV Figure and Appendix Figure Source Data [file 44321_2025_200_MOESM8_ESM.zip › Appendix Fig. S4/Appendix Fig. S4B/Appendix Fig. S4B.png]

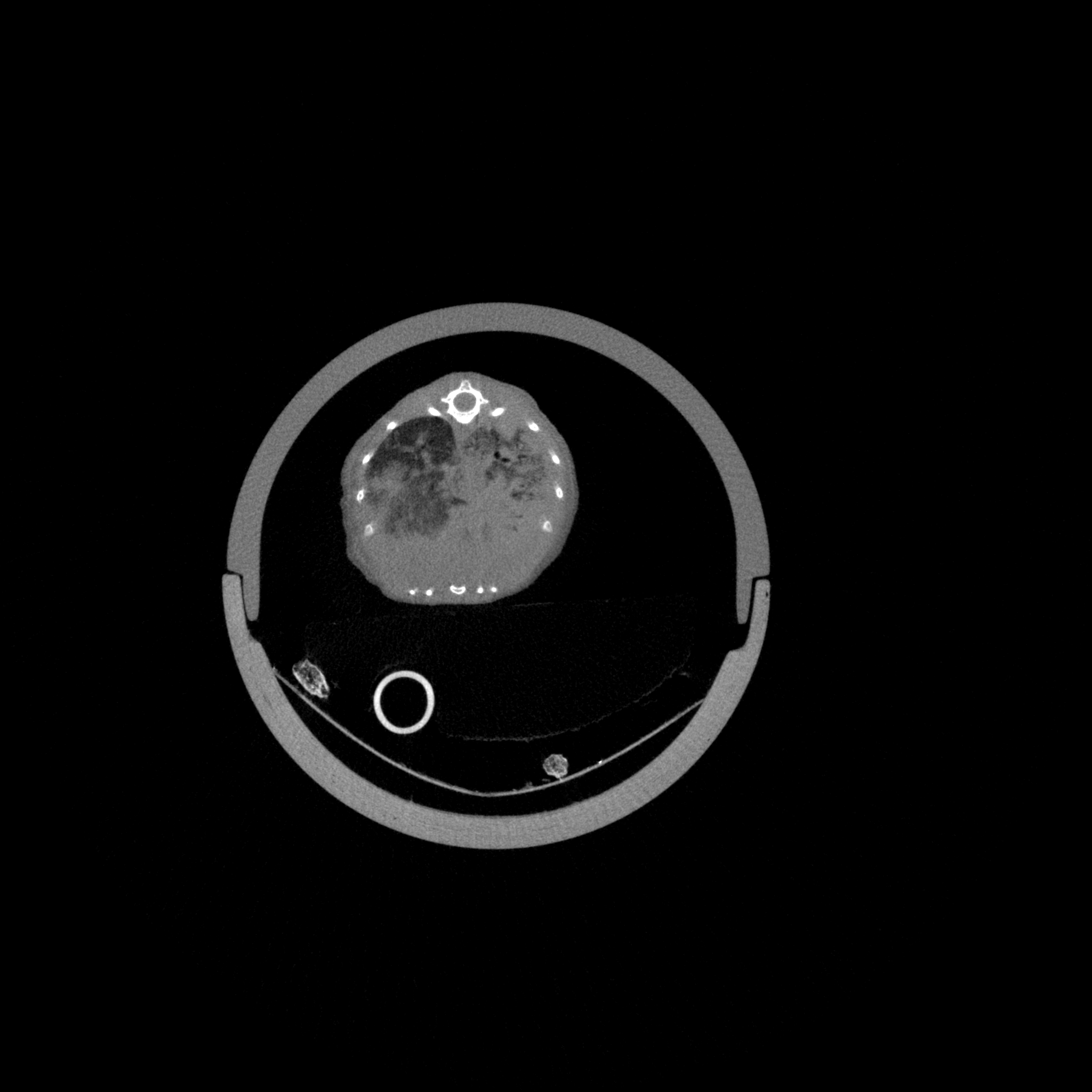

Supplement: Supplementary file 8 — EV Figure and Appendix Figure Source Data [file 44321_2025_200_MOESM8_ESM.zip › Appendix Fig. S5/Appendix Fig. S5A/PreRX Lac.tif]

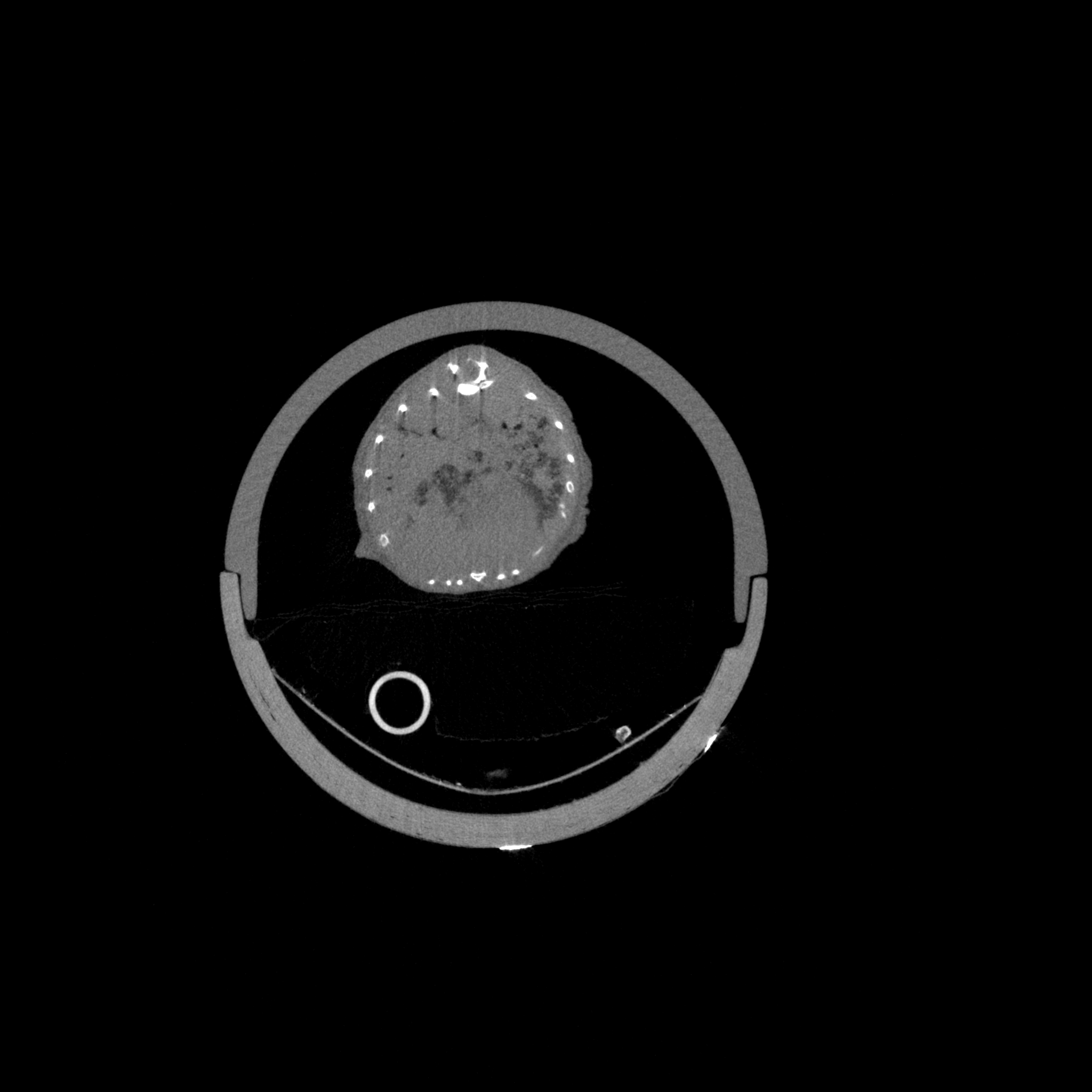

Supplement: Supplementary file 8 — EV Figure and Appendix Figure Source Data [file 44321_2025_200_MOESM8_ESM.zip › Appendix Fig. S5/Appendix Fig. S5A/PreRX Veh.tif]

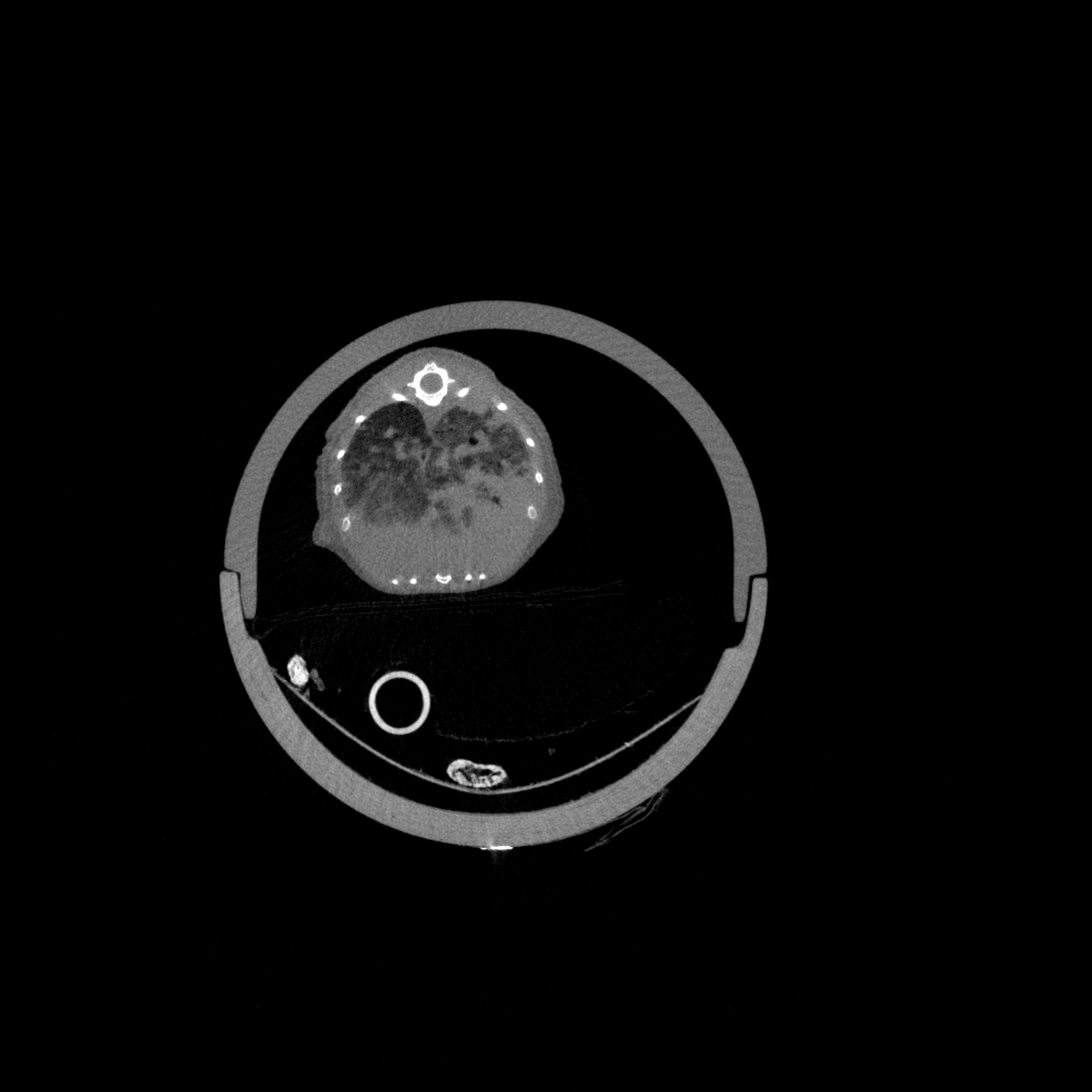

Supplement: Supplementary file 8 — EV Figure and Appendix Figure Source Data [file 44321_2025_200_MOESM8_ESM.zip › Appendix Fig. S5/Appendix Fig. S5A/PstRX Lac.tif]

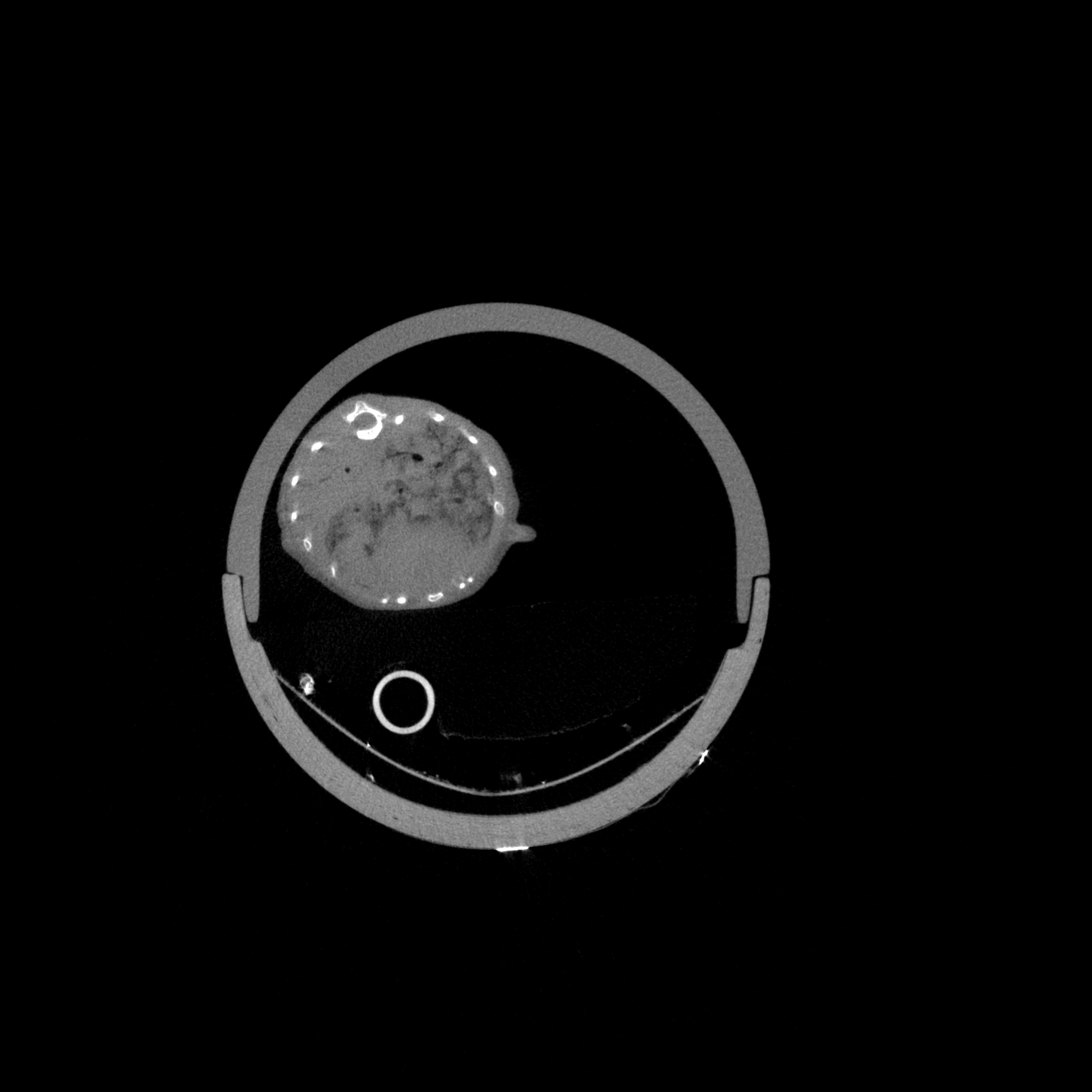

Supplement: Supplementary file 8 — EV Figure and Appendix Figure Source Data [file 44321_2025_200_MOESM8_ESM.zip › Appendix Fig. S5/Appendix Fig. S5A/PstRX Veh.tif]

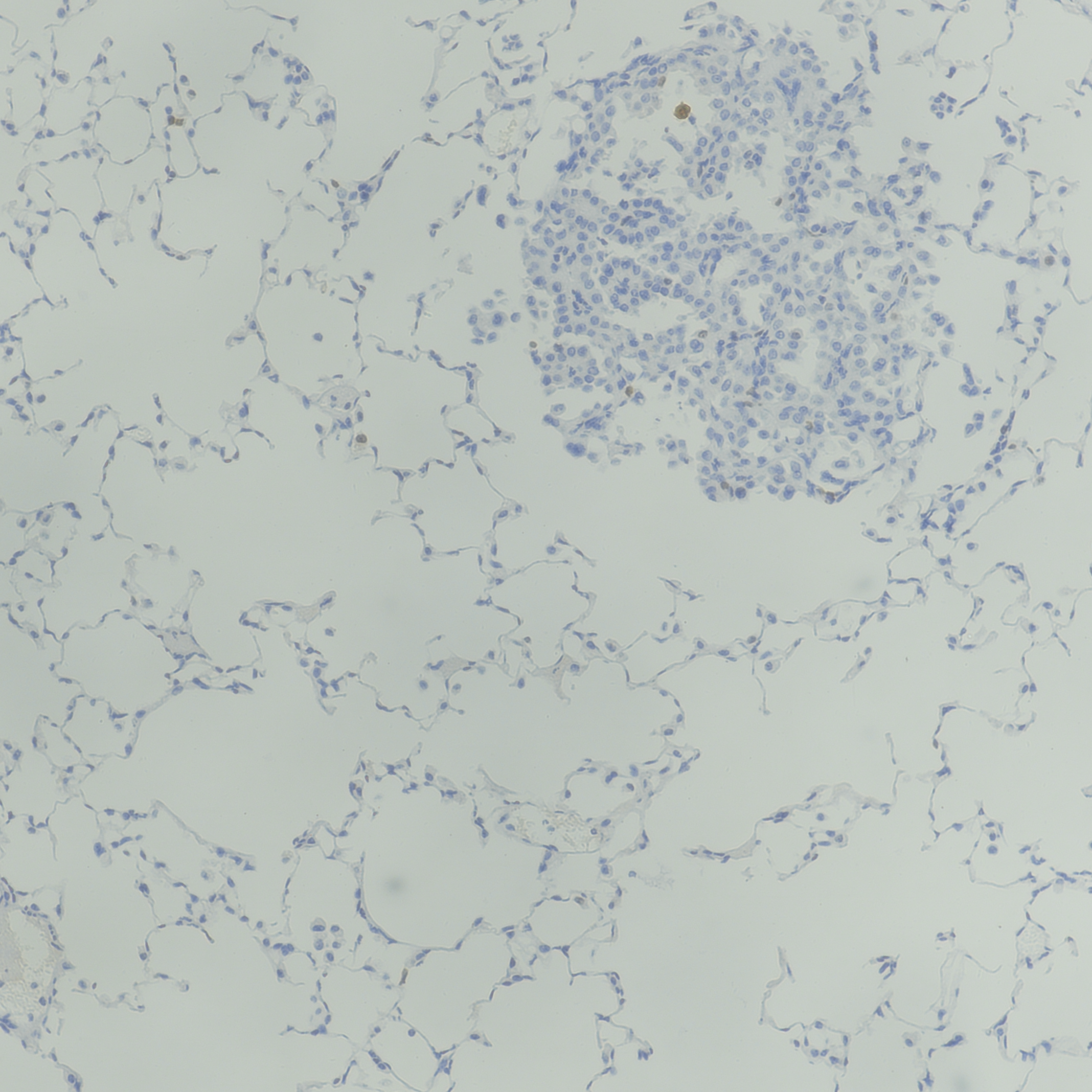

Supplement: Supplementary file 8 — EV Figure and Appendix Figure Source Data [file 44321_2025_200_MOESM8_ESM.zip › Appendix Fig. S5/Appendix Fig. S5C/KrasG12D Lac. ki67.tif]

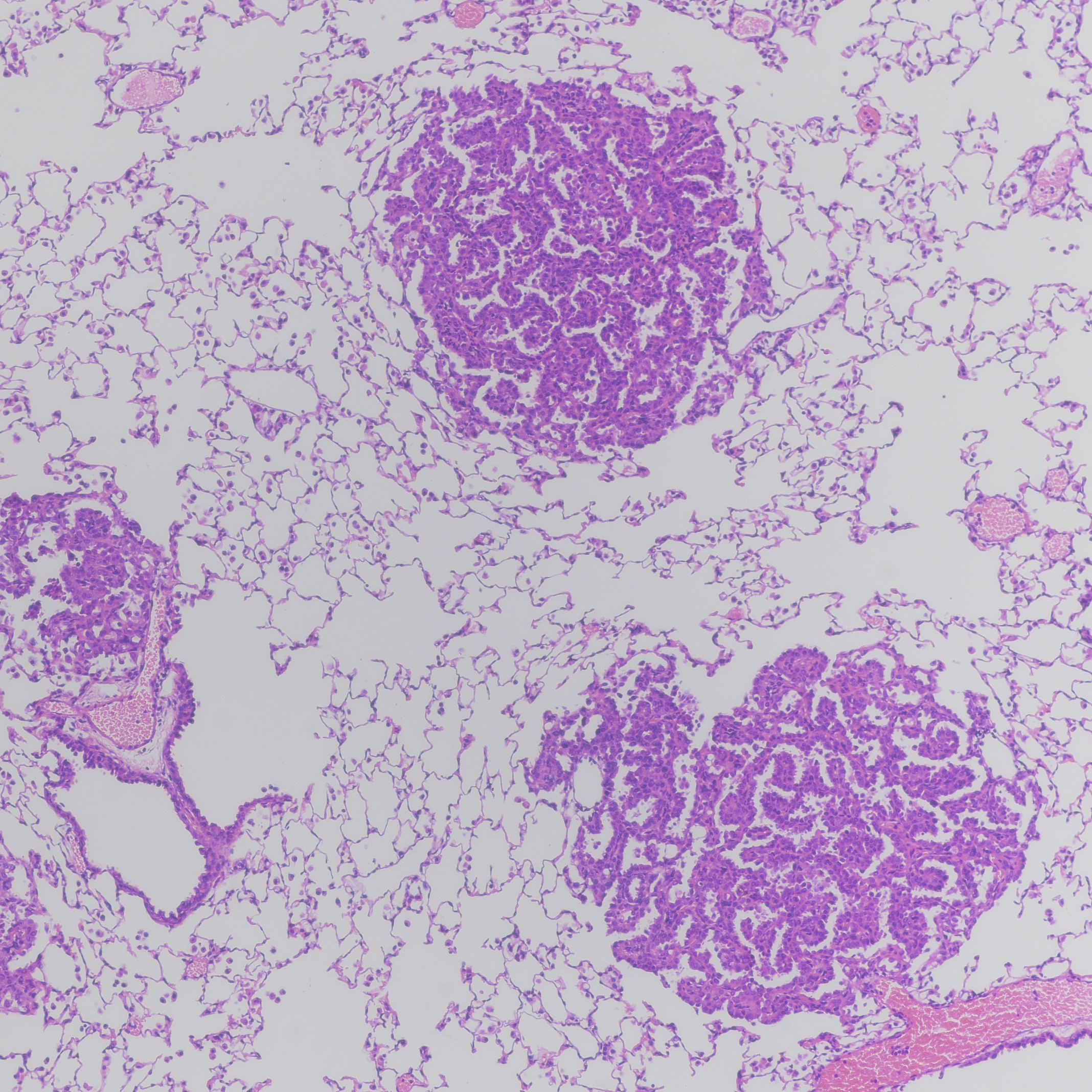

Supplement: Supplementary file 8 — EV Figure and Appendix Figure Source Data [file 44321_2025_200_MOESM8_ESM.zip › Appendix Fig. S5/Appendix Fig. S5C/KrasG12D Lac.HE.tif]

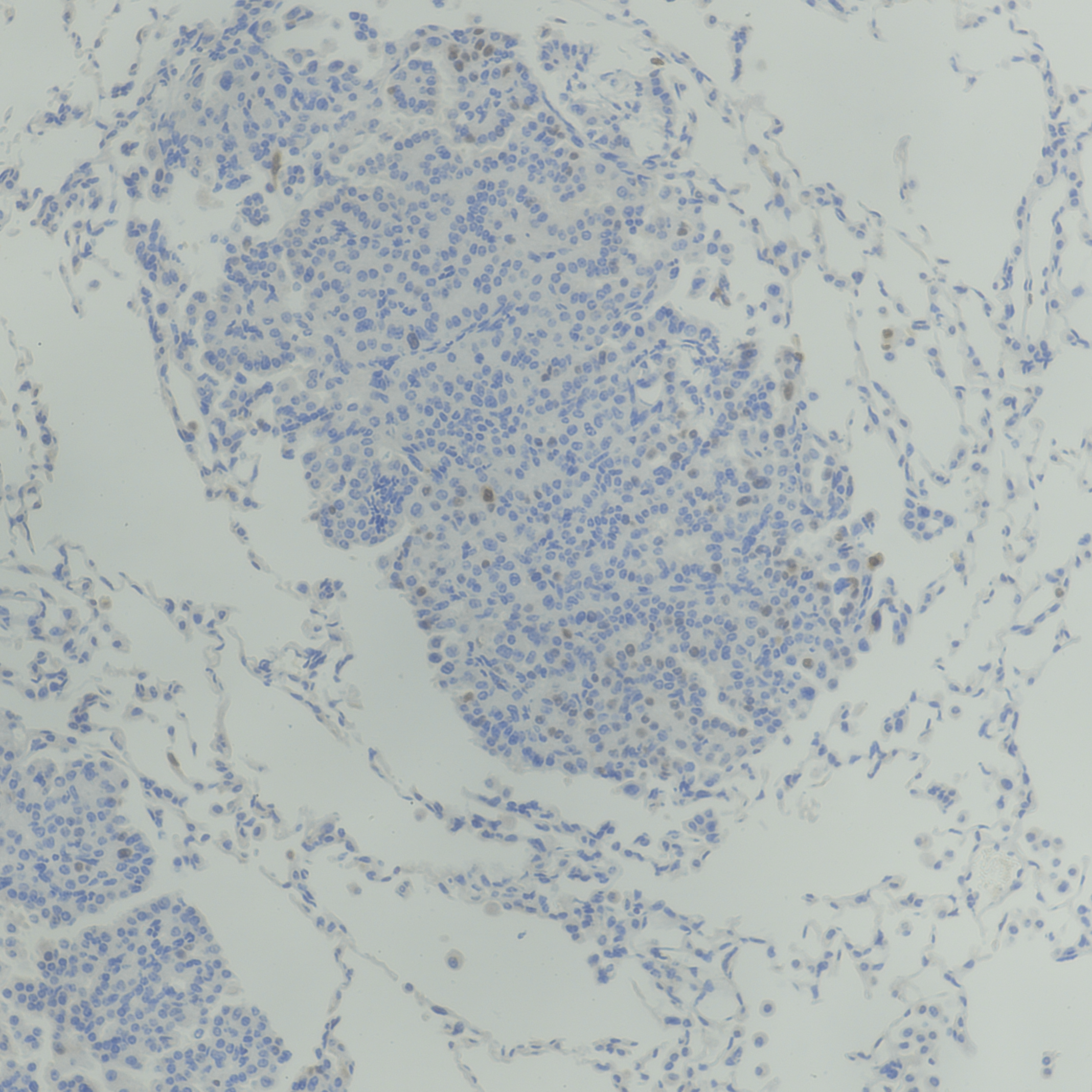

Supplement: Supplementary file 8 — EV Figure and Appendix Figure Source Data [file 44321_2025_200_MOESM8_ESM.zip › Appendix Fig. S5/Appendix Fig. S5C/KrasG12D Veh. ki67.tif.tif]

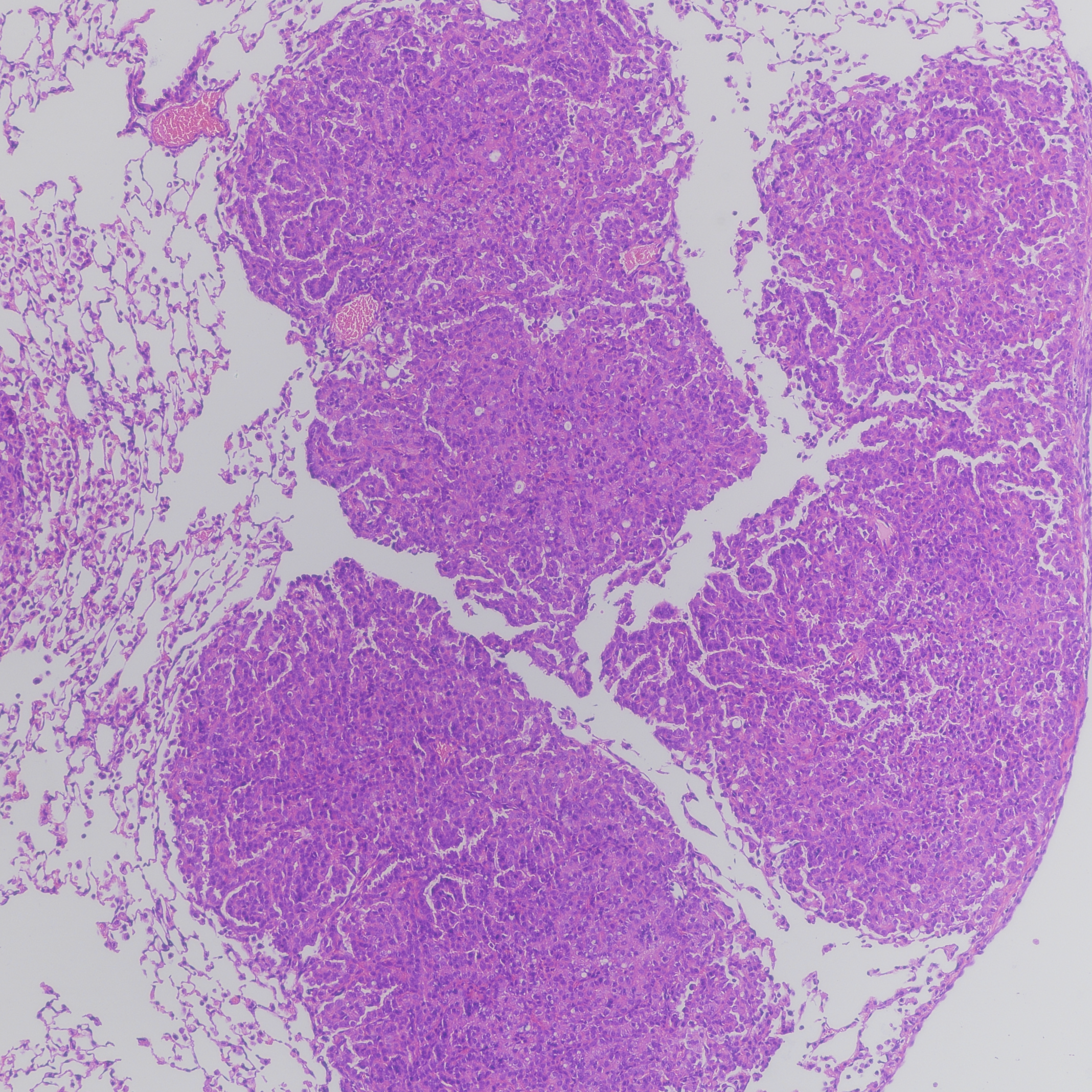

Supplement: Supplementary file 8 — EV Figure and Appendix Figure Source Data [file 44321_2025_200_MOESM8_ESM.zip › Appendix Fig. S5/Appendix Fig. S5C/KrasG12D Veh.HE.tif]

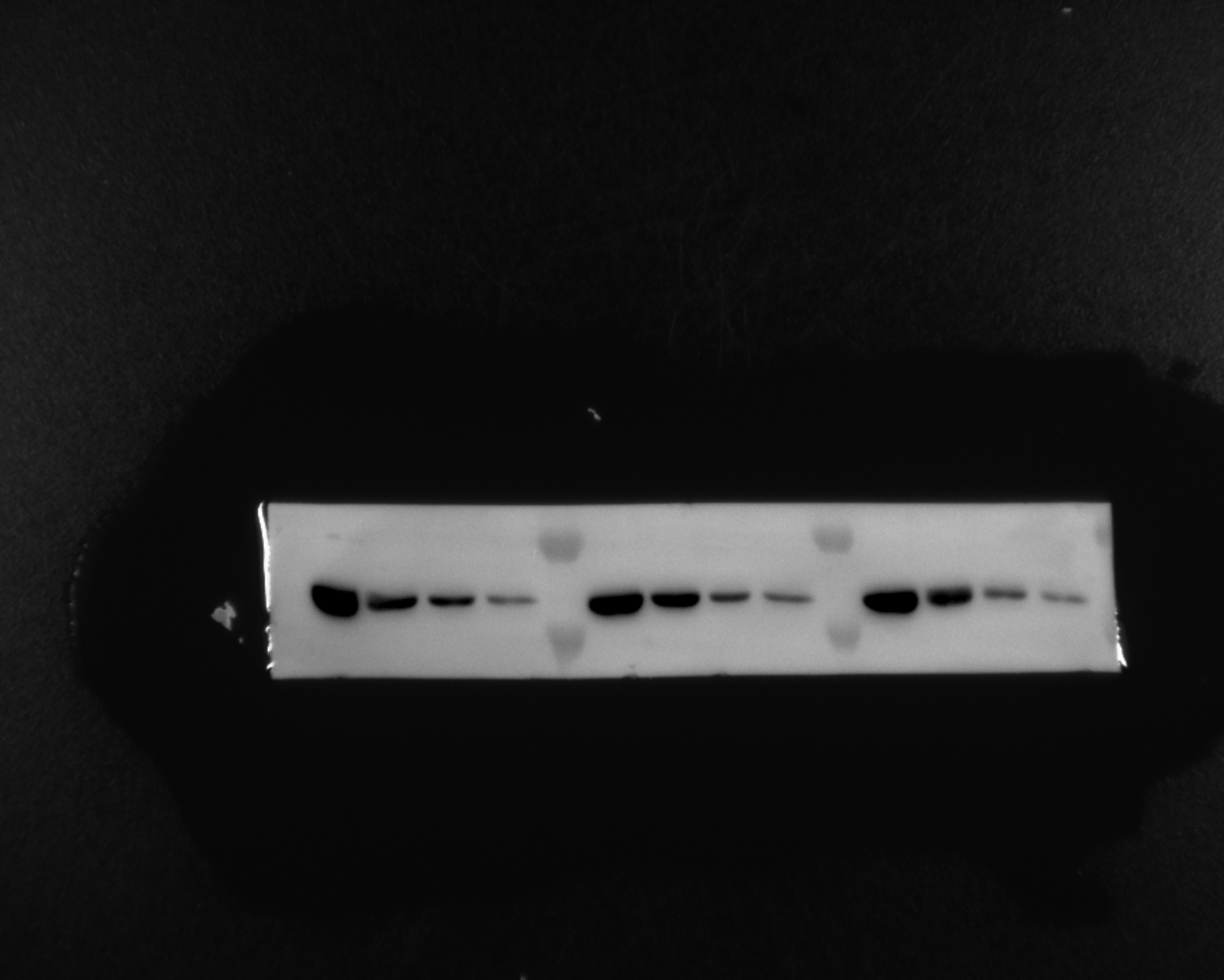

Supplement: Supplementary file 8 — EV Figure and Appendix Figure Source Data [file 44321_2025_200_MOESM8_ESM.zip › Fig. EV3/Fig. EV3A/ACTIN-1.tif]

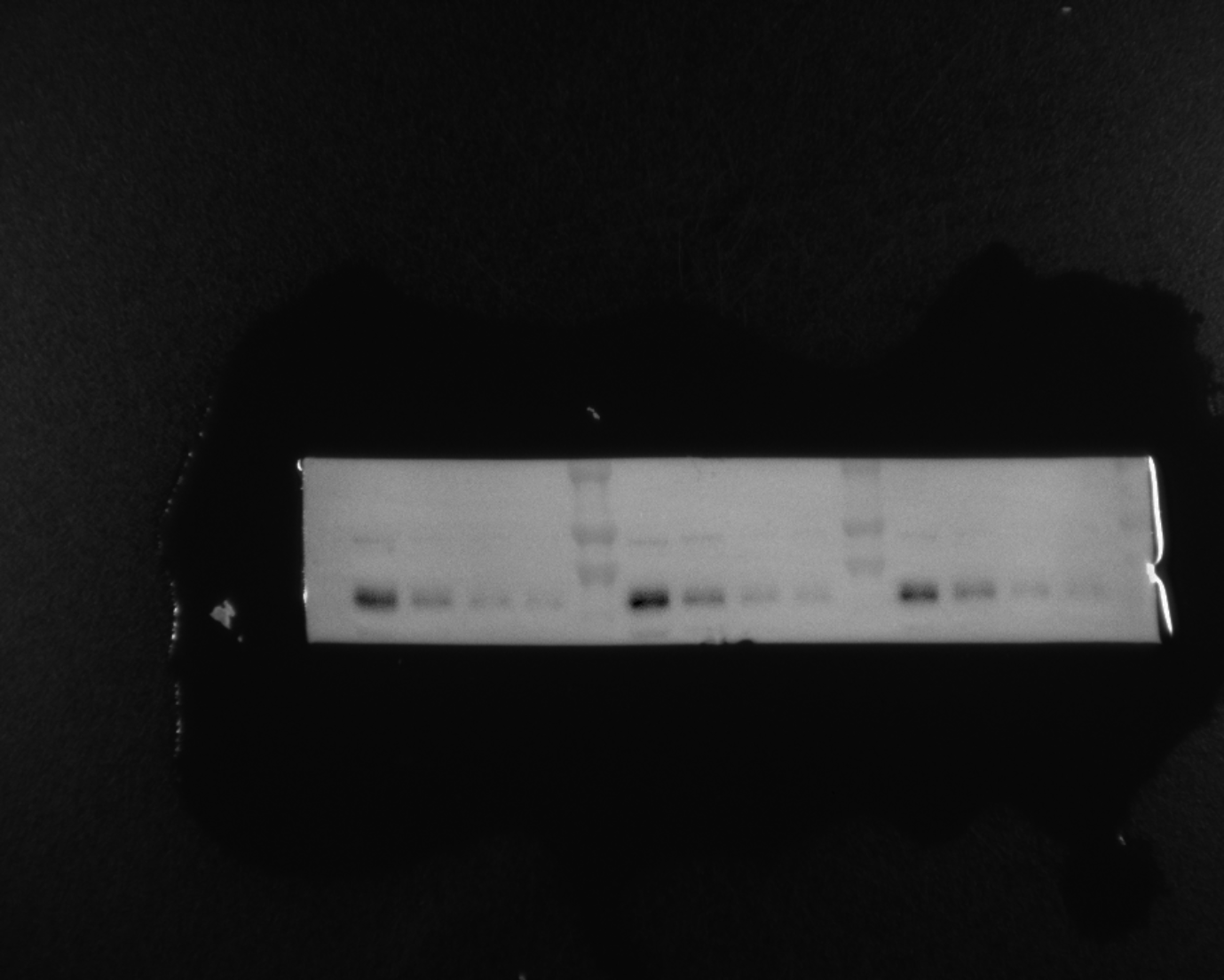

Supplement: Supplementary file 8 — EV Figure and Appendix Figure Source Data [file 44321_2025_200_MOESM8_ESM.zip › Fig. EV3/Fig. EV3A/RUNX1-1.tif]

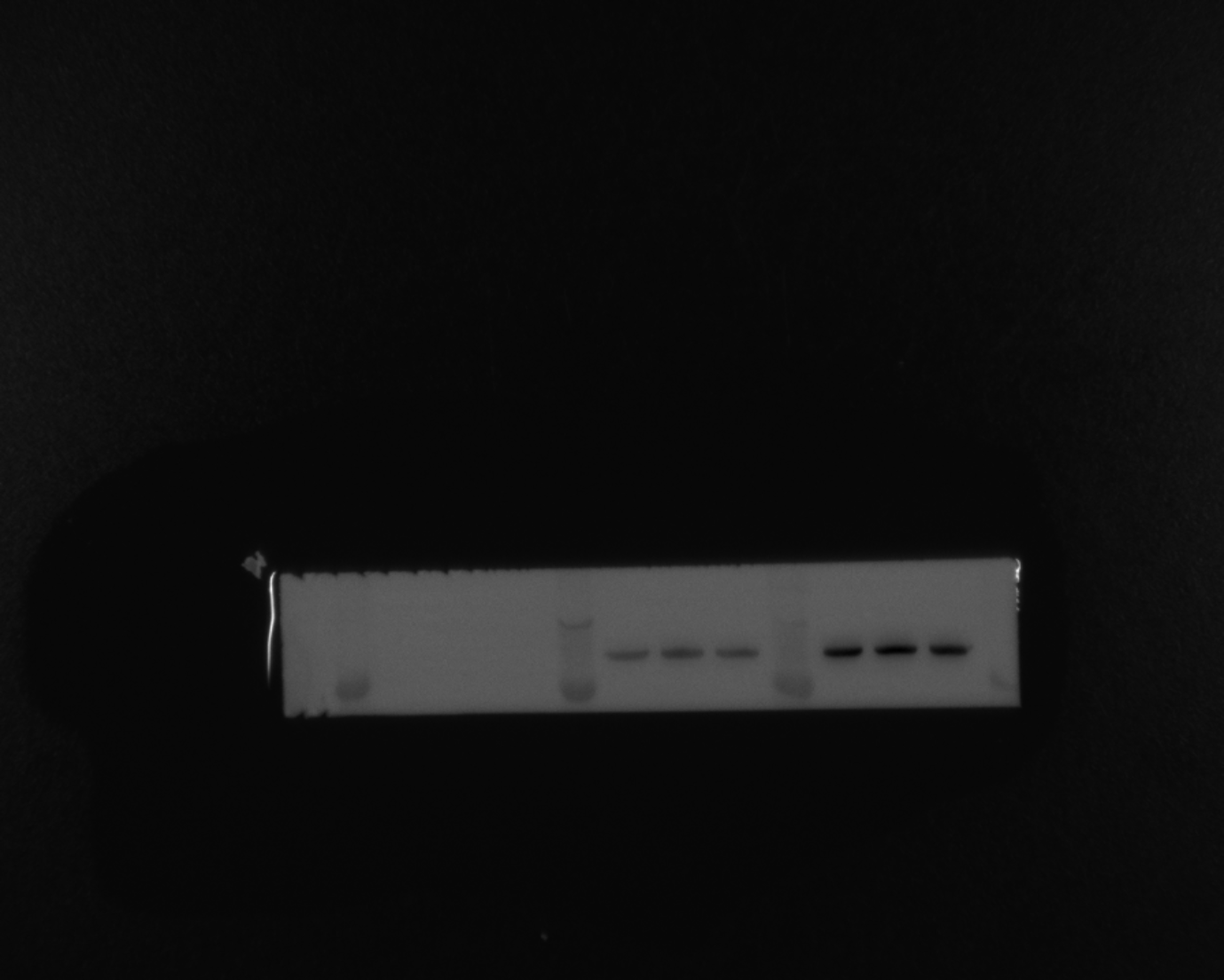

Supplement: Supplementary file 8 — EV Figure and Appendix Figure Source Data [file 44321_2025_200_MOESM8_ESM.zip › Fig. EV3/Fig. EV3C/ACTIN.tif]

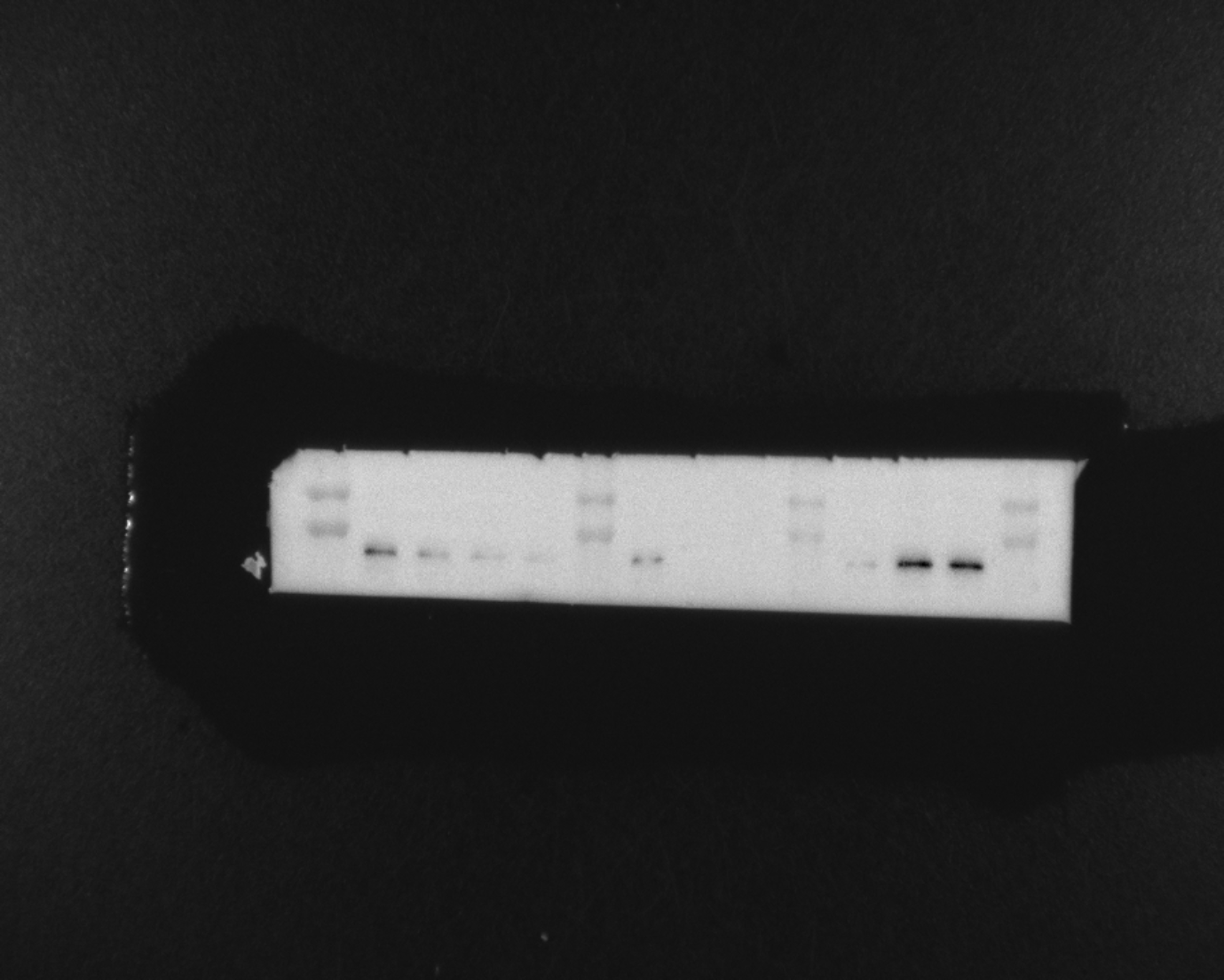

Supplement: Supplementary file 8 — EV Figure and Appendix Figure Source Data [file 44321_2025_200_MOESM8_ESM.zip › Fig. EV3/Fig. EV3C/IP-RUNX1-Clu.tif]

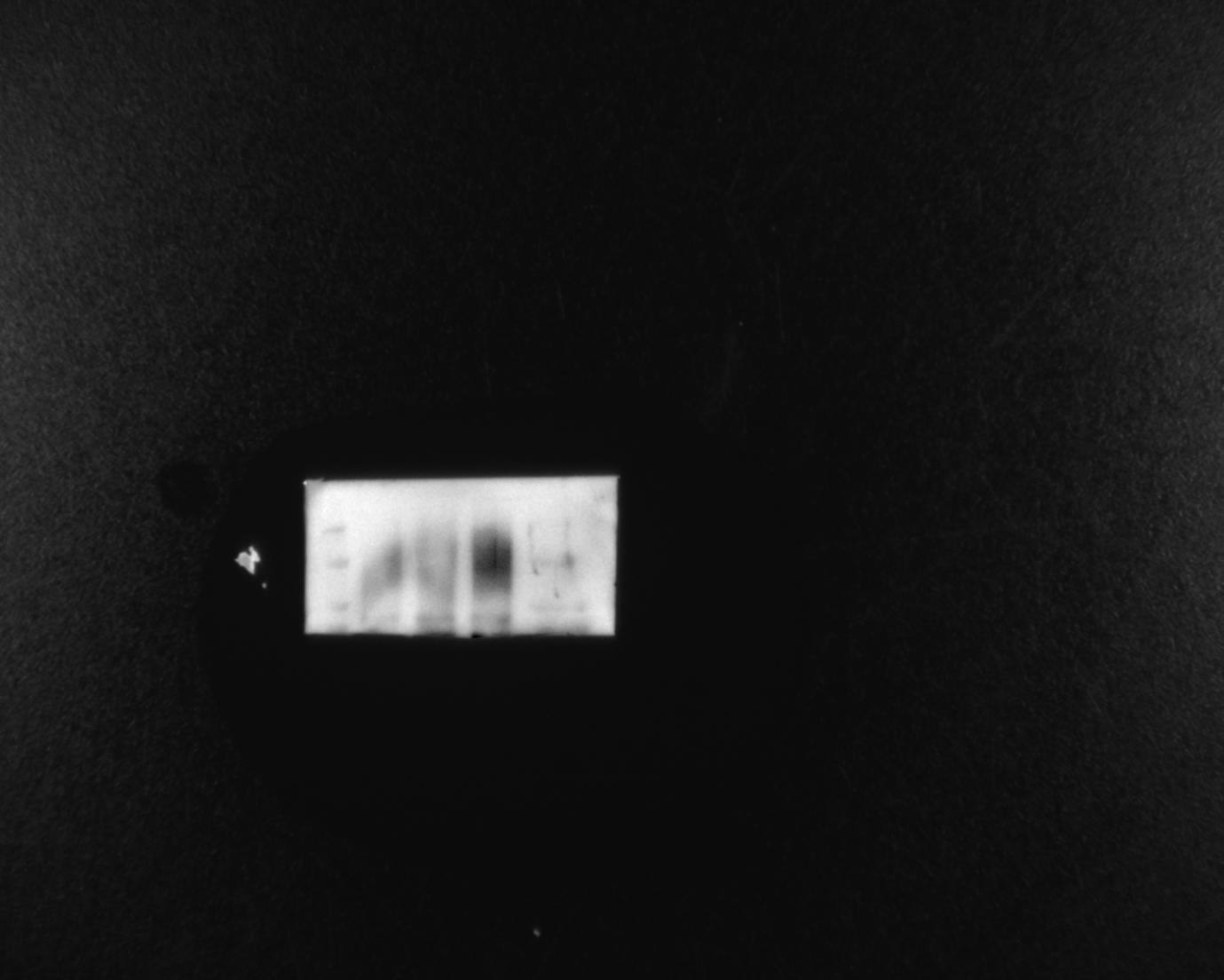

Supplement: Supplementary file 8 — EV Figure and Appendix Figure Source Data [file 44321_2025_200_MOESM8_ESM.zip › Fig. EV3/Fig. EV3C/IP-UB-Myc.tif]

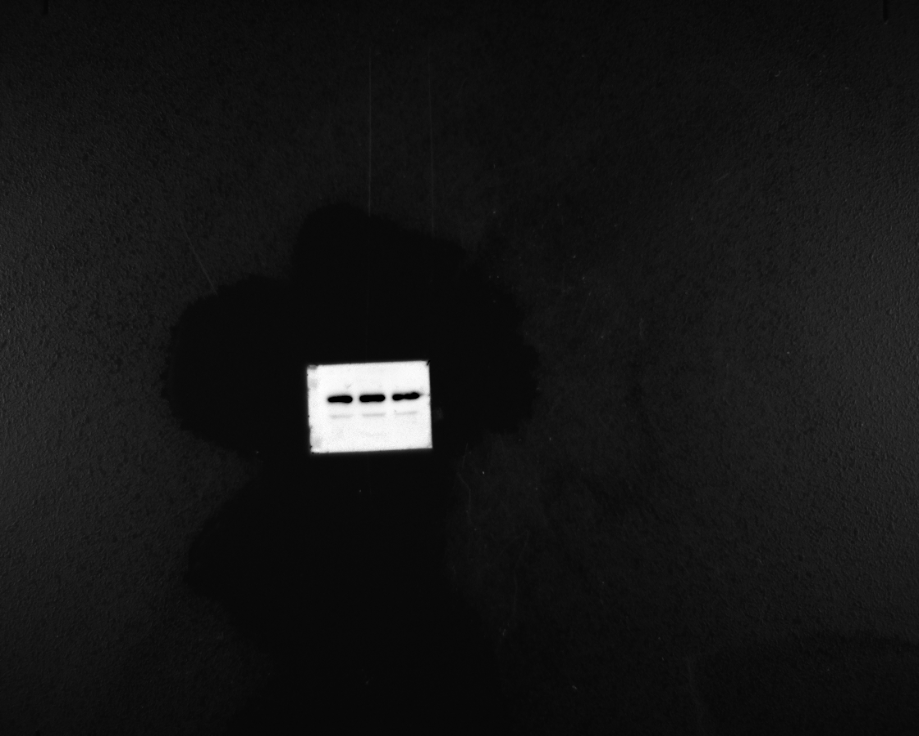

Supplement: Supplementary file 8 — EV Figure and Appendix Figure Source Data [file 44321_2025_200_MOESM8_ESM.zip › Fig. EV3/Fig. EV3C/WCL-RUNX1-Clu.tif]

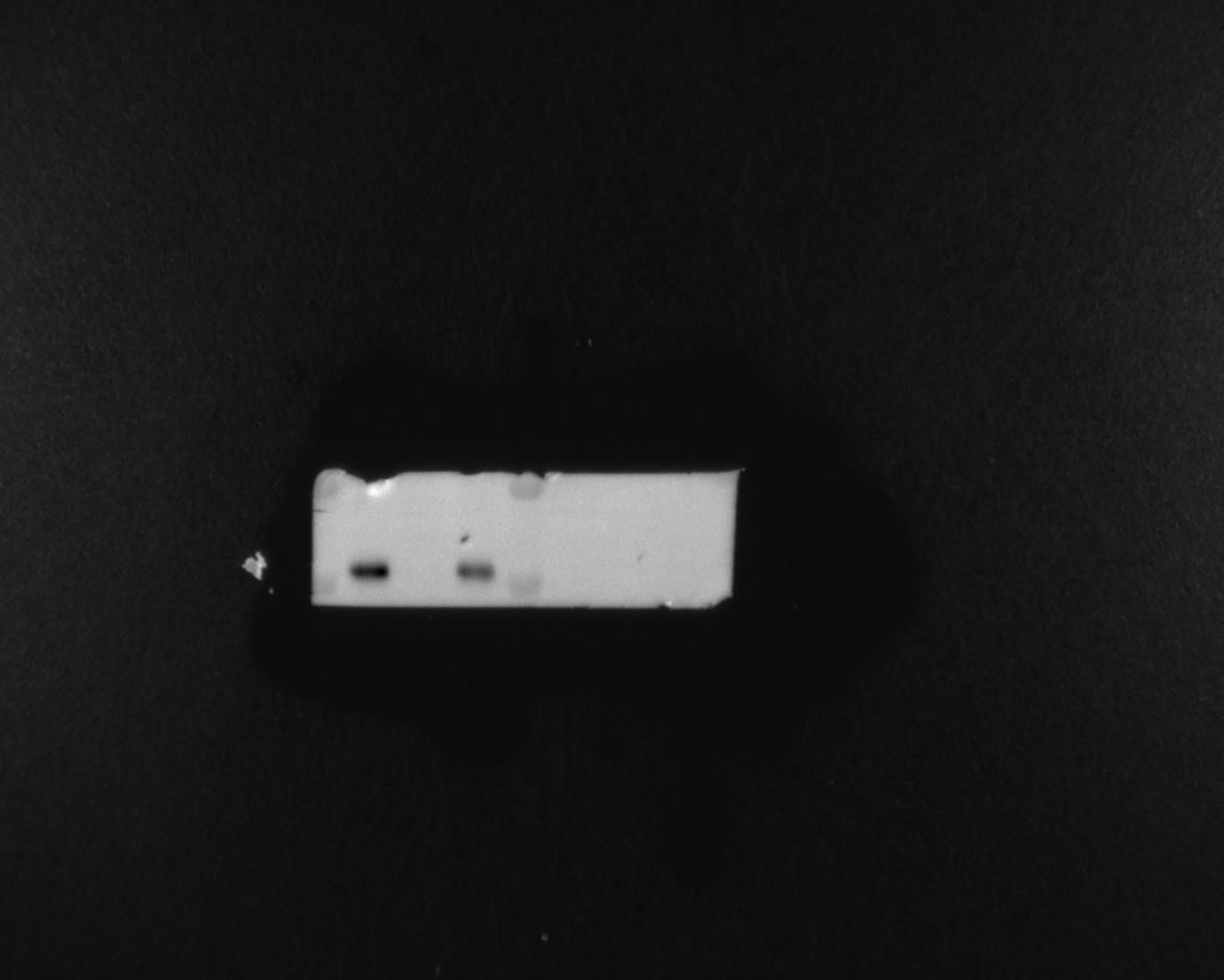

Supplement: Supplementary file 8 — EV Figure and Appendix Figure Source Data [file 44321_2025_200_MOESM8_ESM.zip › Fig. EV3/Fig. EV3C/WCL-STUB1-HA.tif]

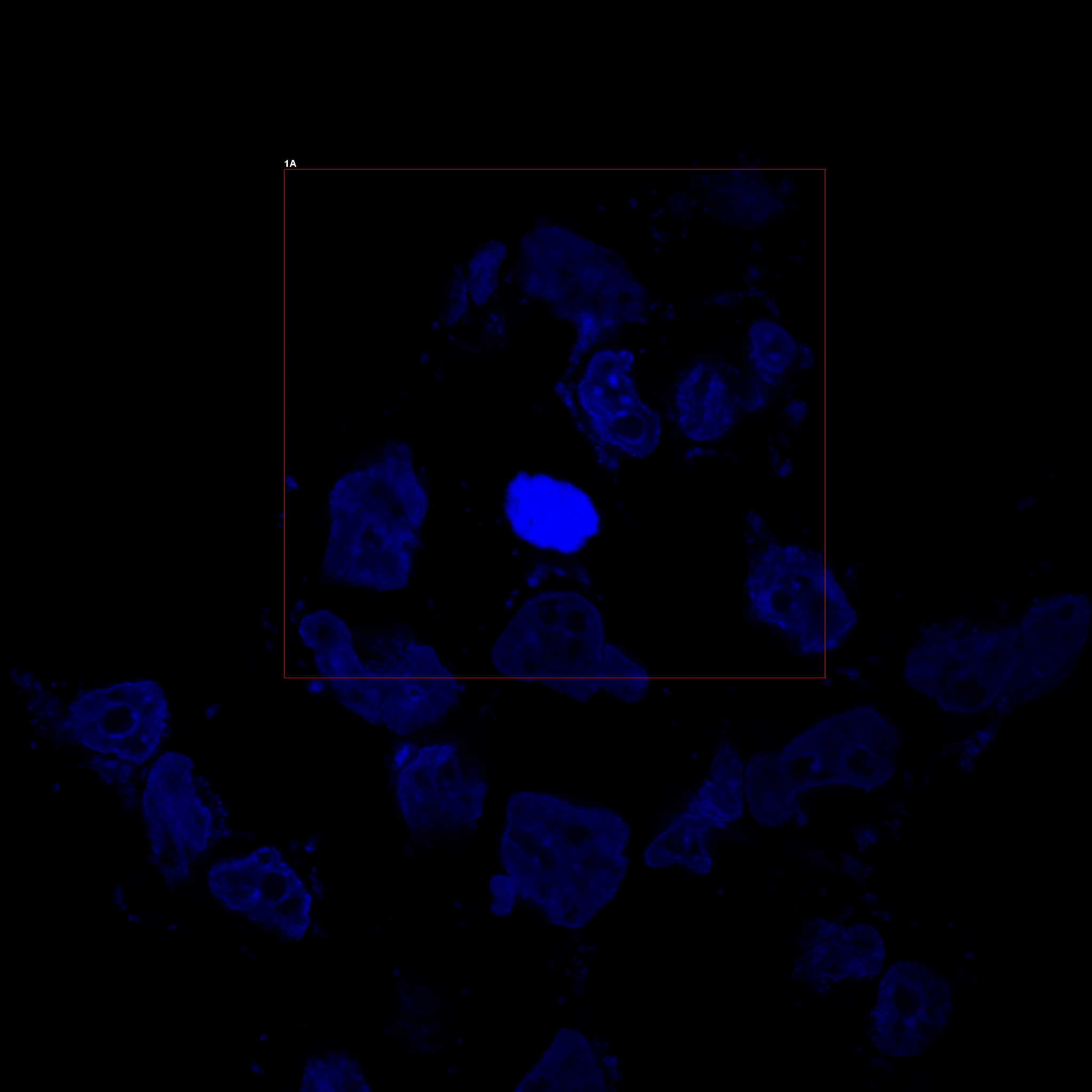

Supplement: Supplementary file 8 — EV Figure and Appendix Figure Source Data [file 44321_2025_200_MOESM8_ESM.zip › Fig. EV3/Fig. EV3D/DAPI.tif]

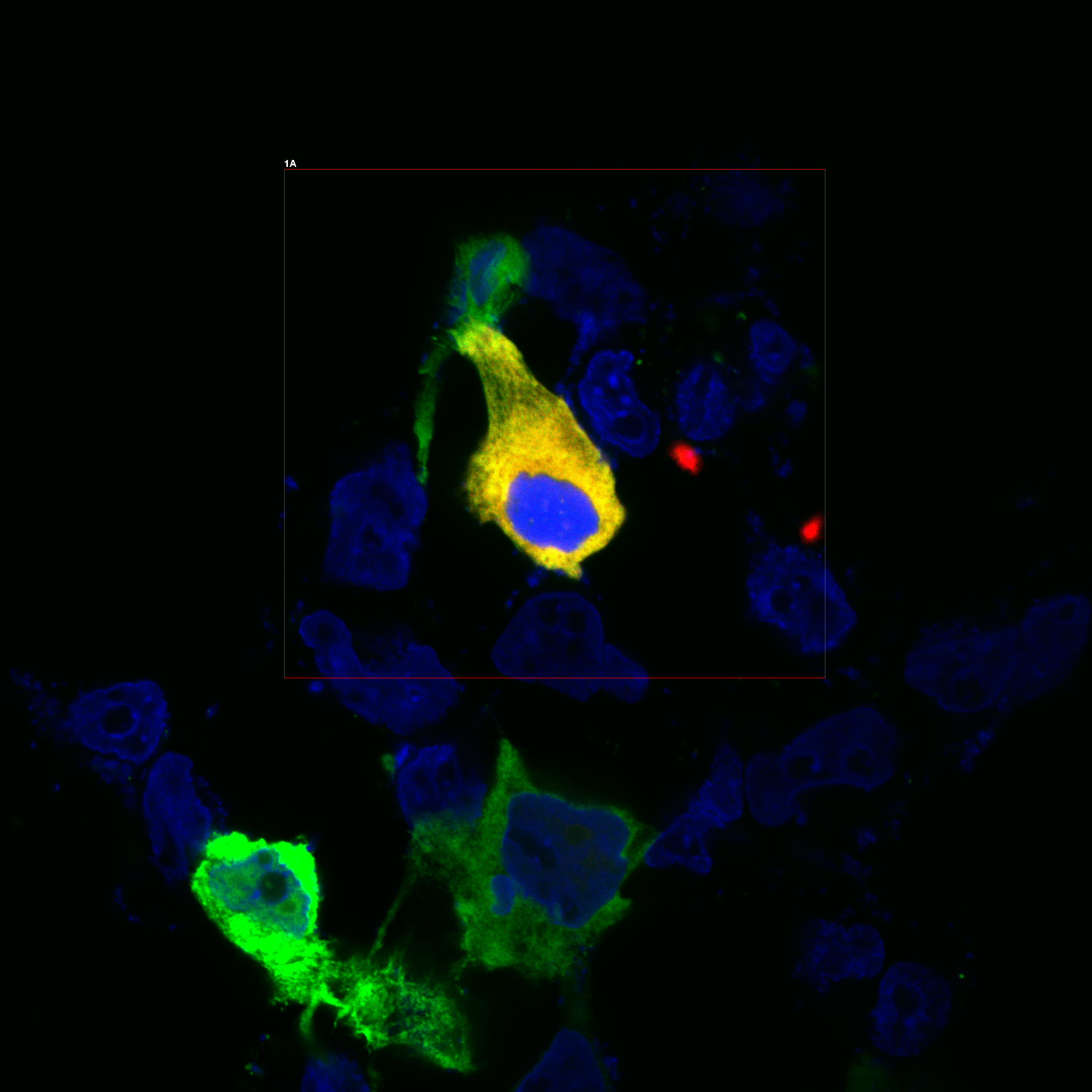

Supplement: Supplementary file 8 — EV Figure and Appendix Figure Source Data [file 44321_2025_200_MOESM8_ESM.zip › Fig. EV3/Fig. EV3D/Merge.tif]

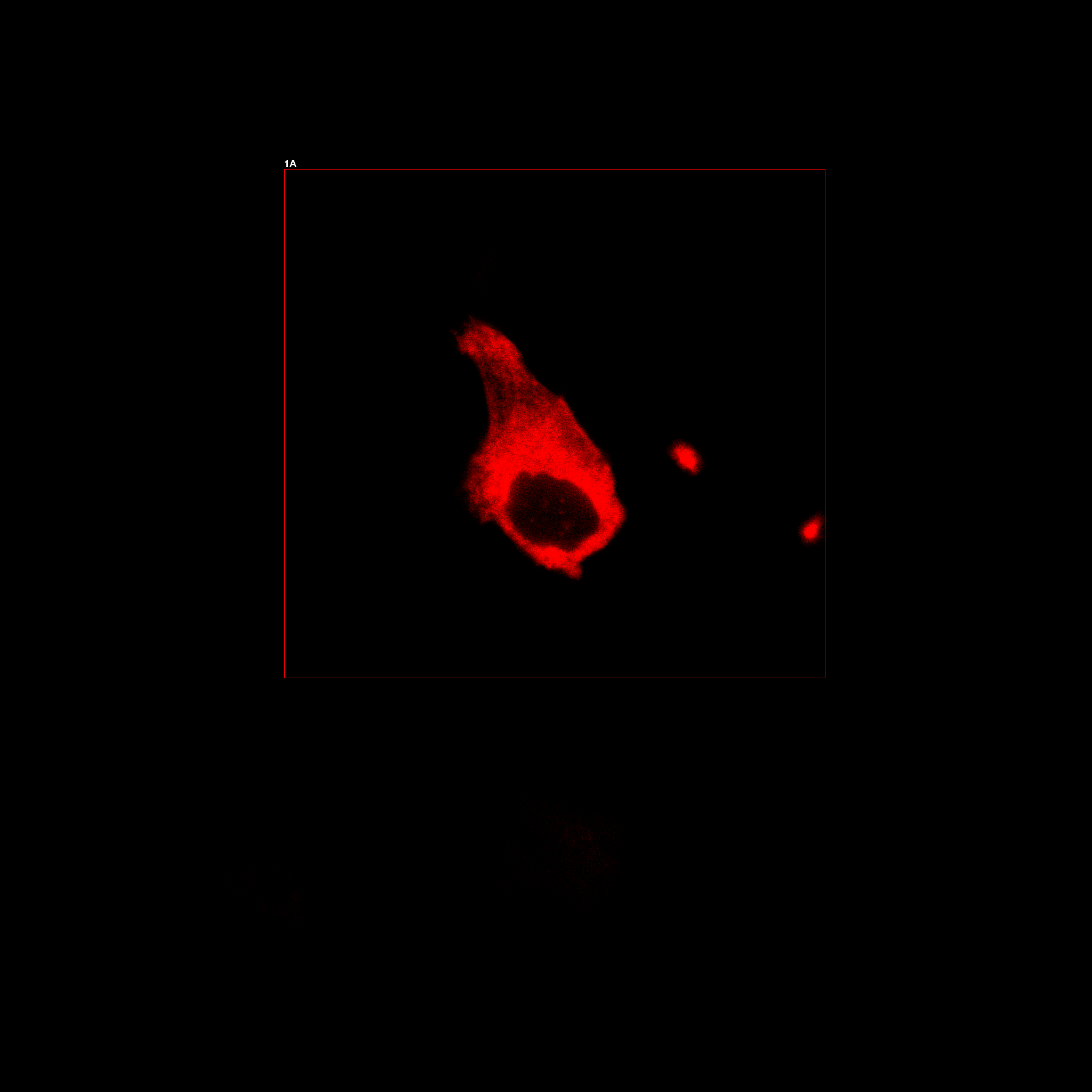

Supplement: Supplementary file 8 — EV Figure and Appendix Figure Source Data [file 44321_2025_200_MOESM8_ESM.zip › Fig. EV3/Fig. EV3D/RUNX1-tdTomato.tif]

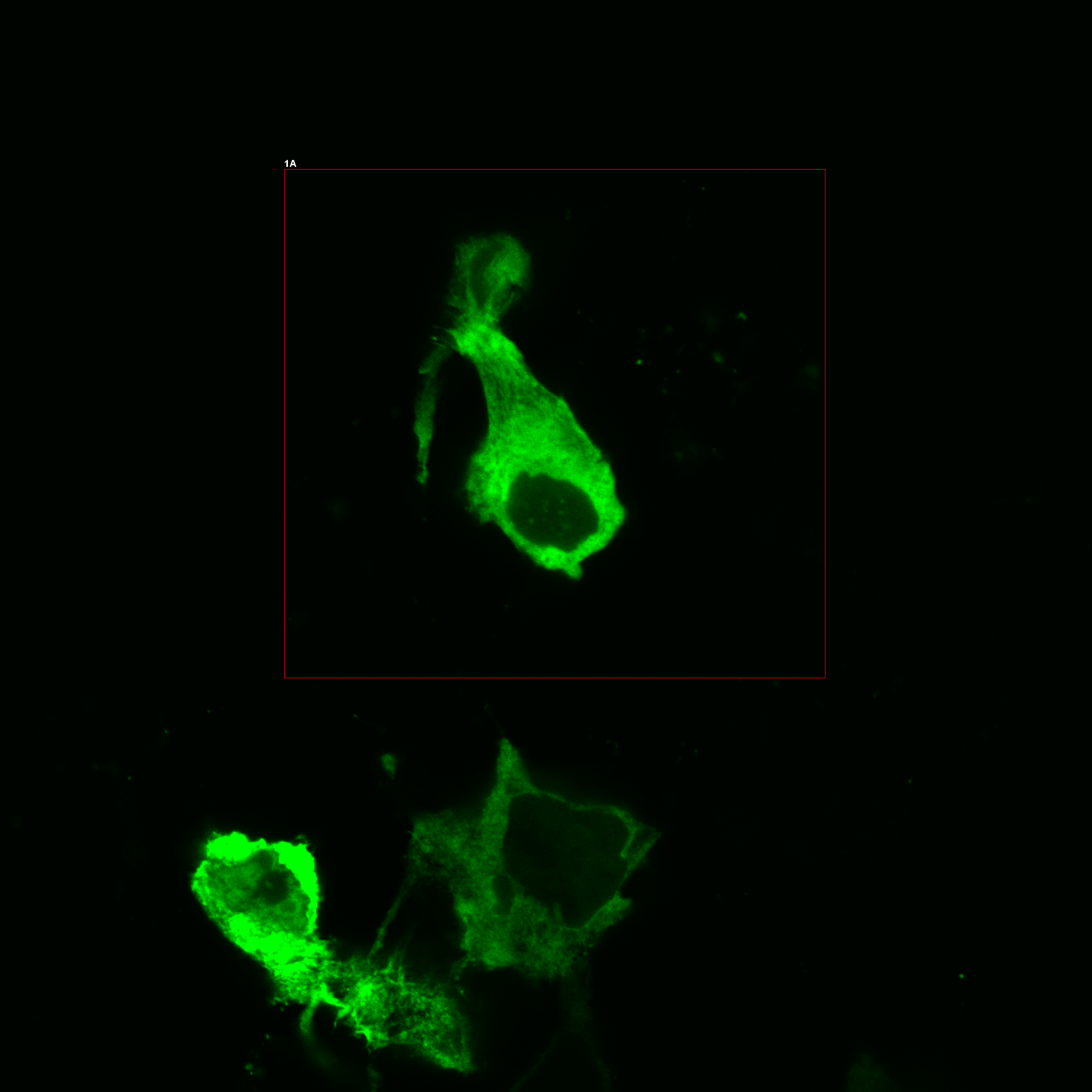

Supplement: Supplementary file 8 — EV Figure and Appendix Figure Source Data [file 44321_2025_200_MOESM8_ESM.zip › Fig. EV3/Fig. EV3D/STUB1-GFP.tif]

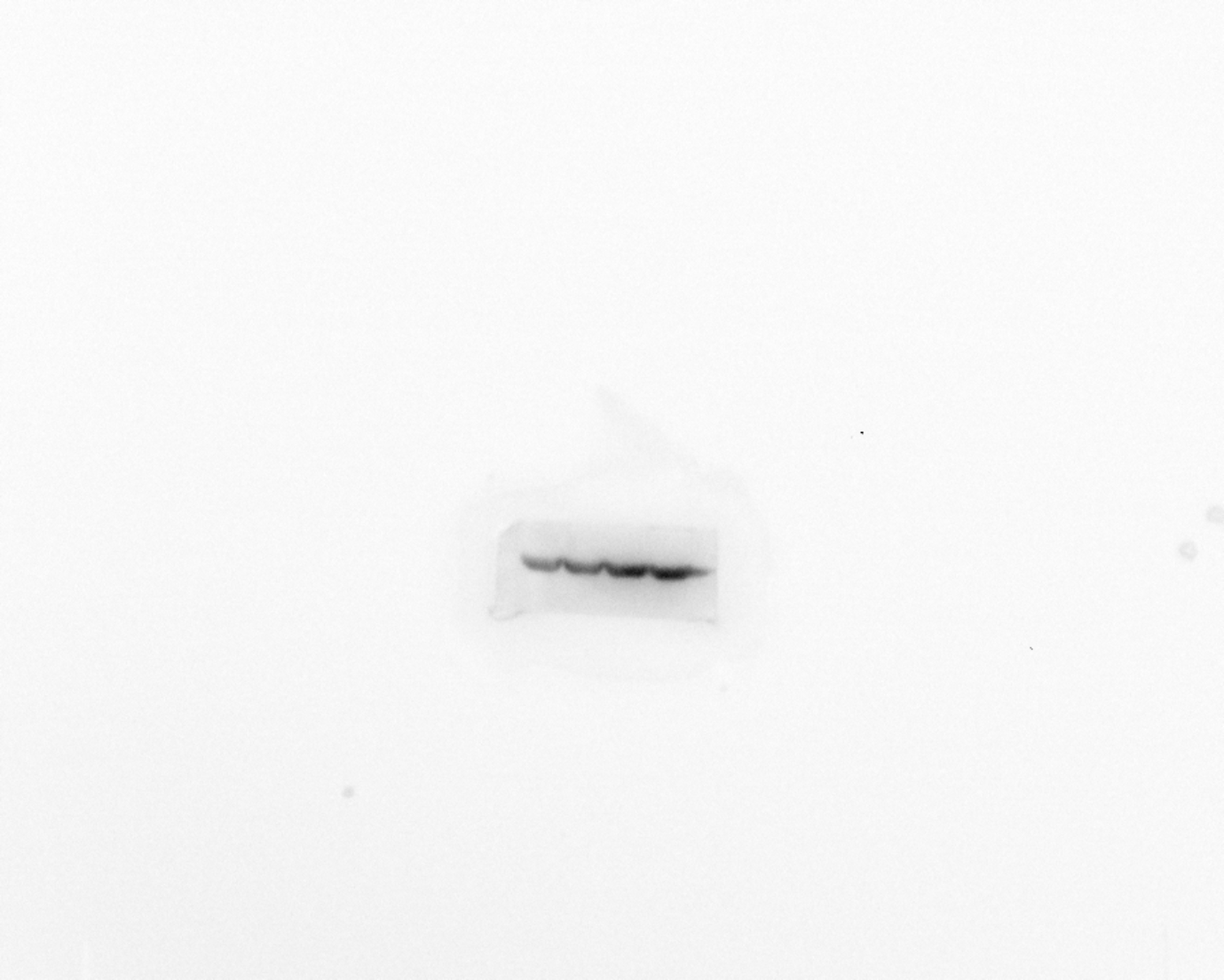

Supplement: Supplementary file 8 — EV Figure and Appendix Figure Source Data [file 44321_2025_200_MOESM8_ESM.zip › Fig. EV3/Fig. EV3G/ACTIN.tif]

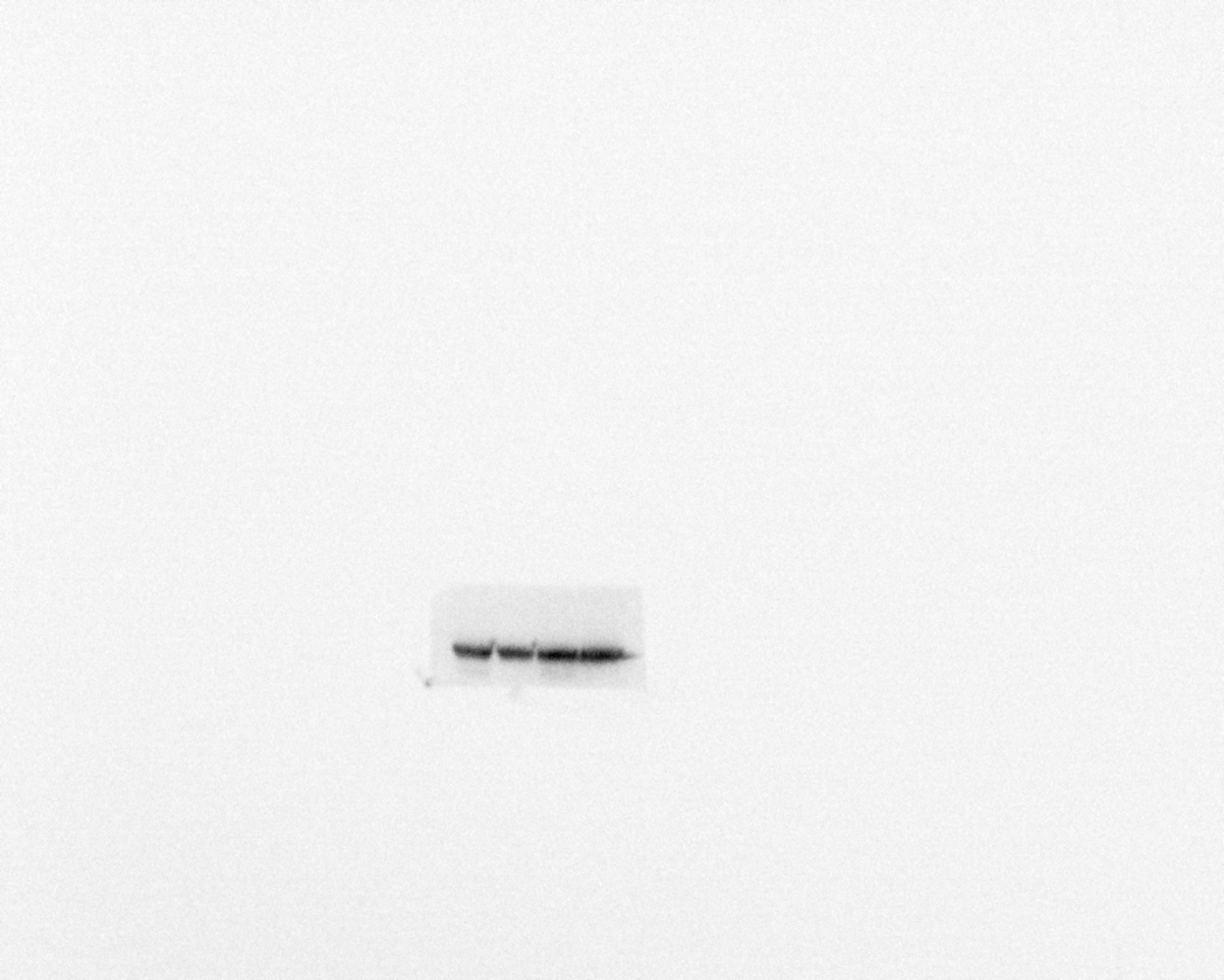

Supplement: Supplementary file 8 — EV Figure and Appendix Figure Source Data [file 44321_2025_200_MOESM8_ESM.zip › Fig. EV3/Fig. EV3G/FOXP3-Flag.tif]

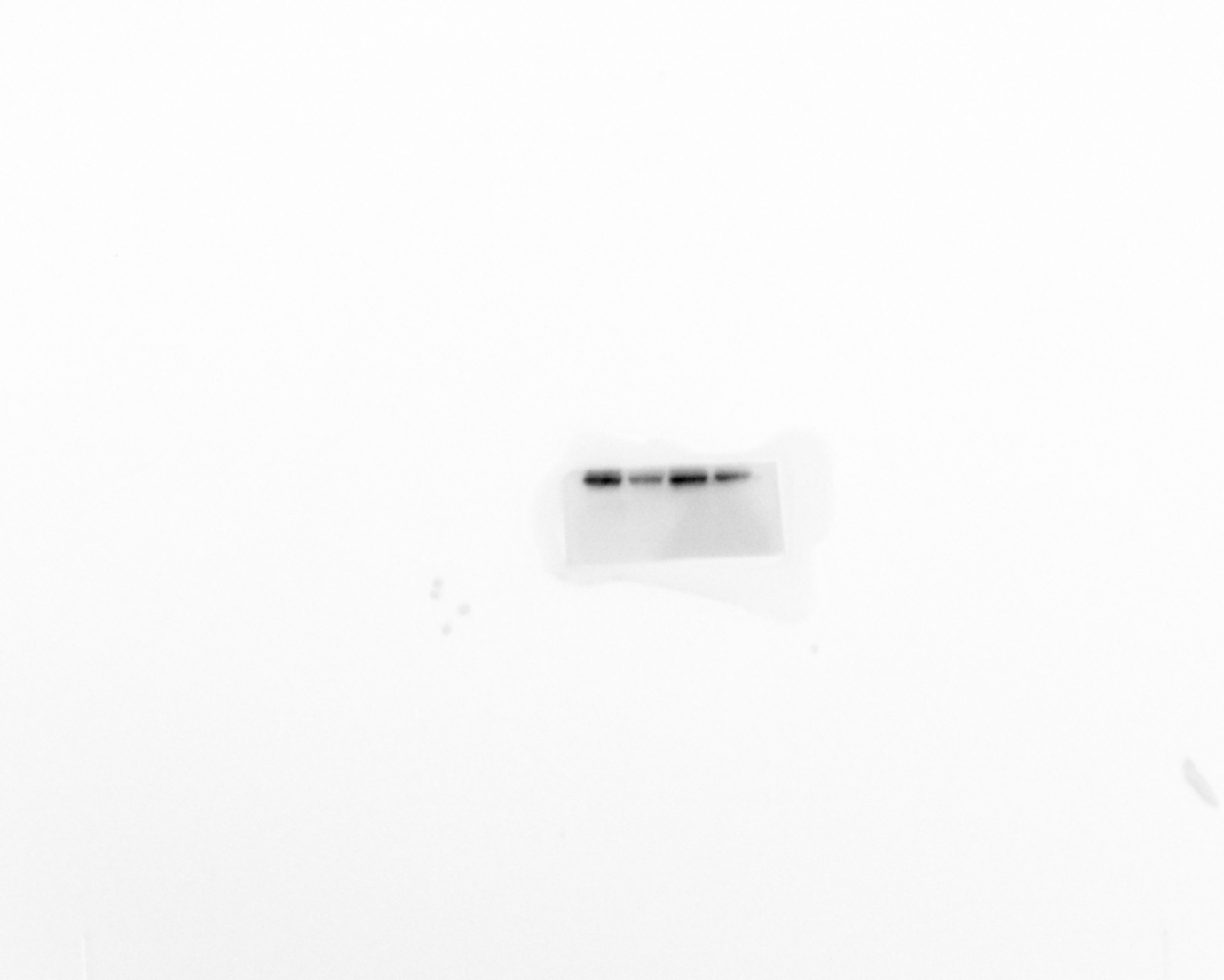

Supplement: Supplementary file 8 — EV Figure and Appendix Figure Source Data [file 44321_2025_200_MOESM8_ESM.zip › Fig. EV3/Fig. EV3G/RUNX1-Myc.tif]

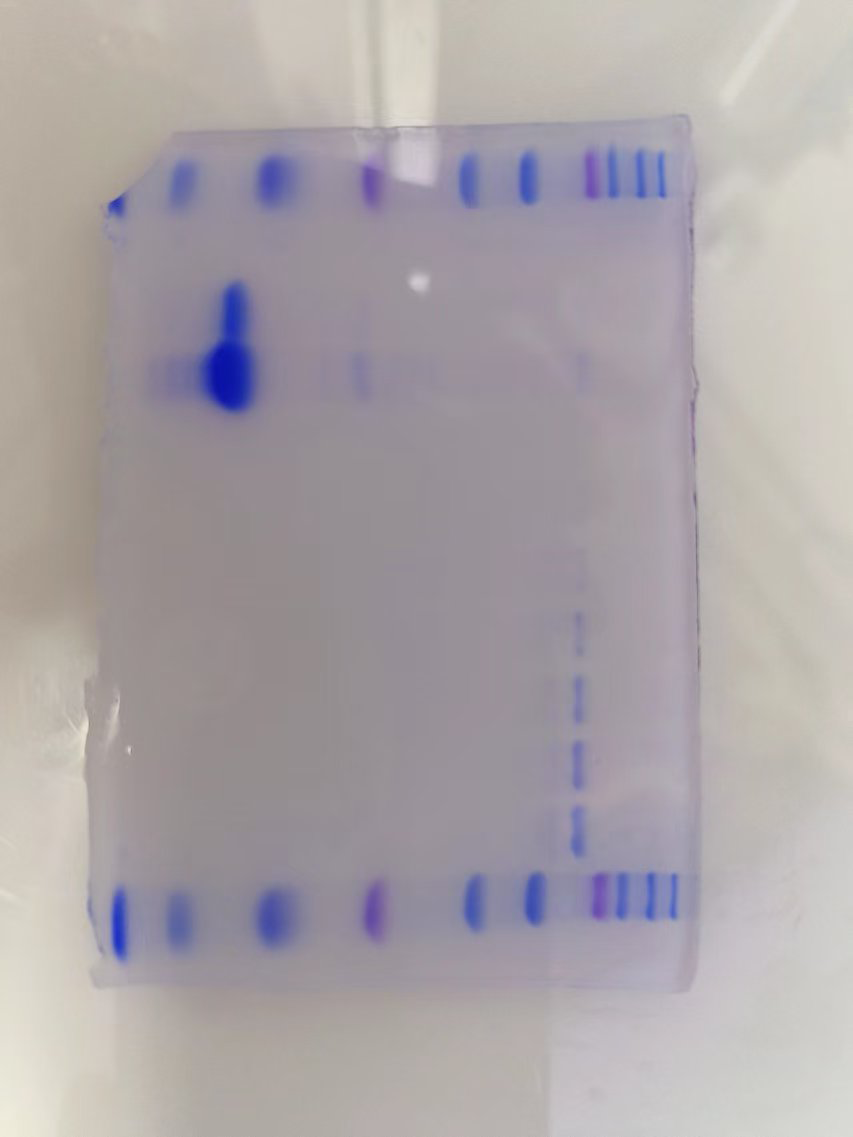

Supplement: Supplementary file 8 — EV Figure and Appendix Figure Source Data [file 44321_2025_200_MOESM8_ESM.zip › Fig. EV4/Fig. EV4A/purified STUB1-1-130-Brilliant Blue.tif]

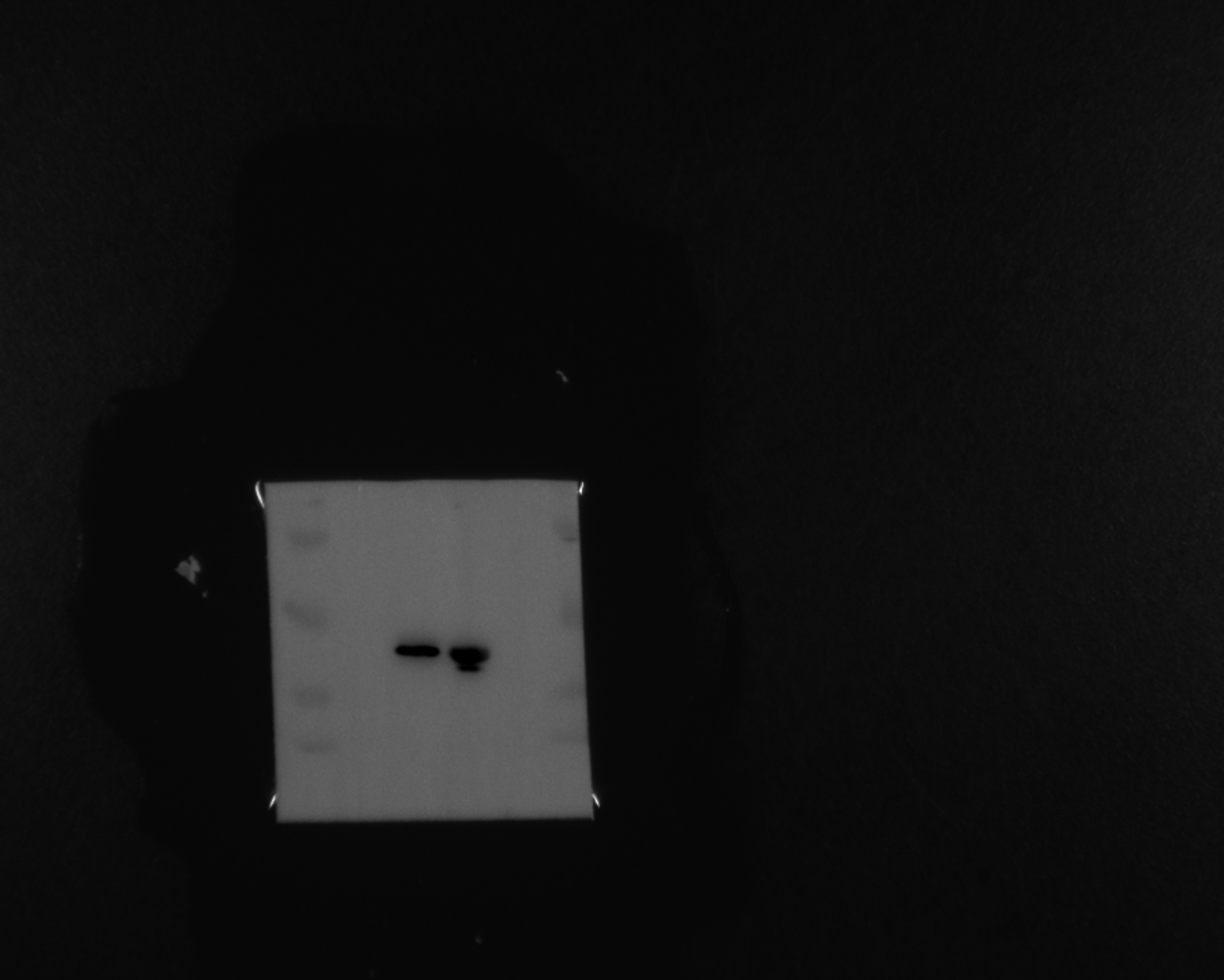

Supplement: Supplementary file 8 — EV Figure and Appendix Figure Source Data [file 44321_2025_200_MOESM8_ESM.zip › Fig. EV4/Fig. EV4A/purified STUB1-1-130-Western Blot.tif]

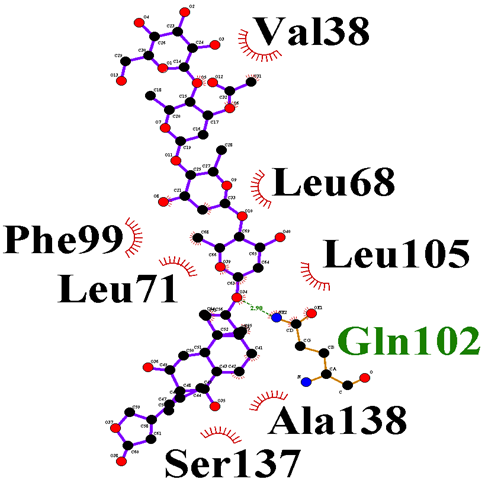

Supplement: Supplementary file 8 — EV Figure and Appendix Figure Source Data [file 44321_2025_200_MOESM8_ESM.zip › Fig. EV4/Fig. EV4C/EV4C-left.png]

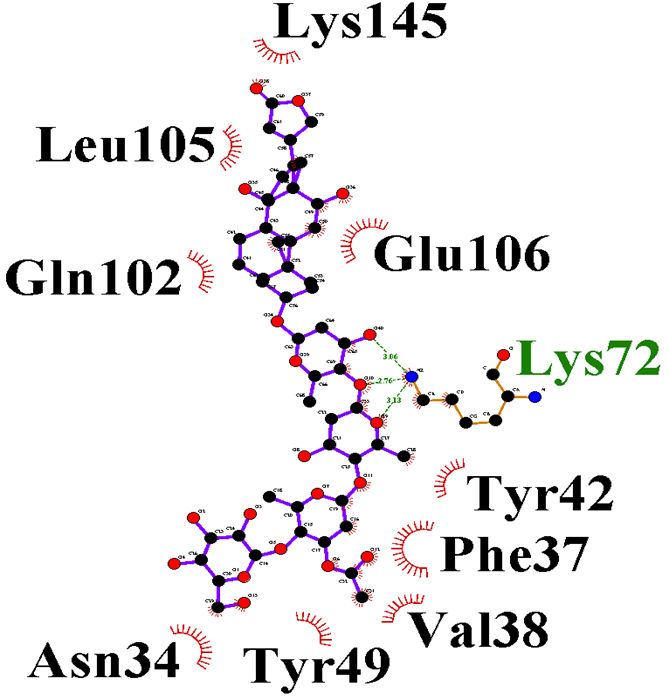

Supplement: Supplementary file 8 — EV Figure and Appendix Figure Source Data [file 44321_2025_200_MOESM8_ESM.zip › Fig. EV4/Fig. EV4C/EV4C-right.png]

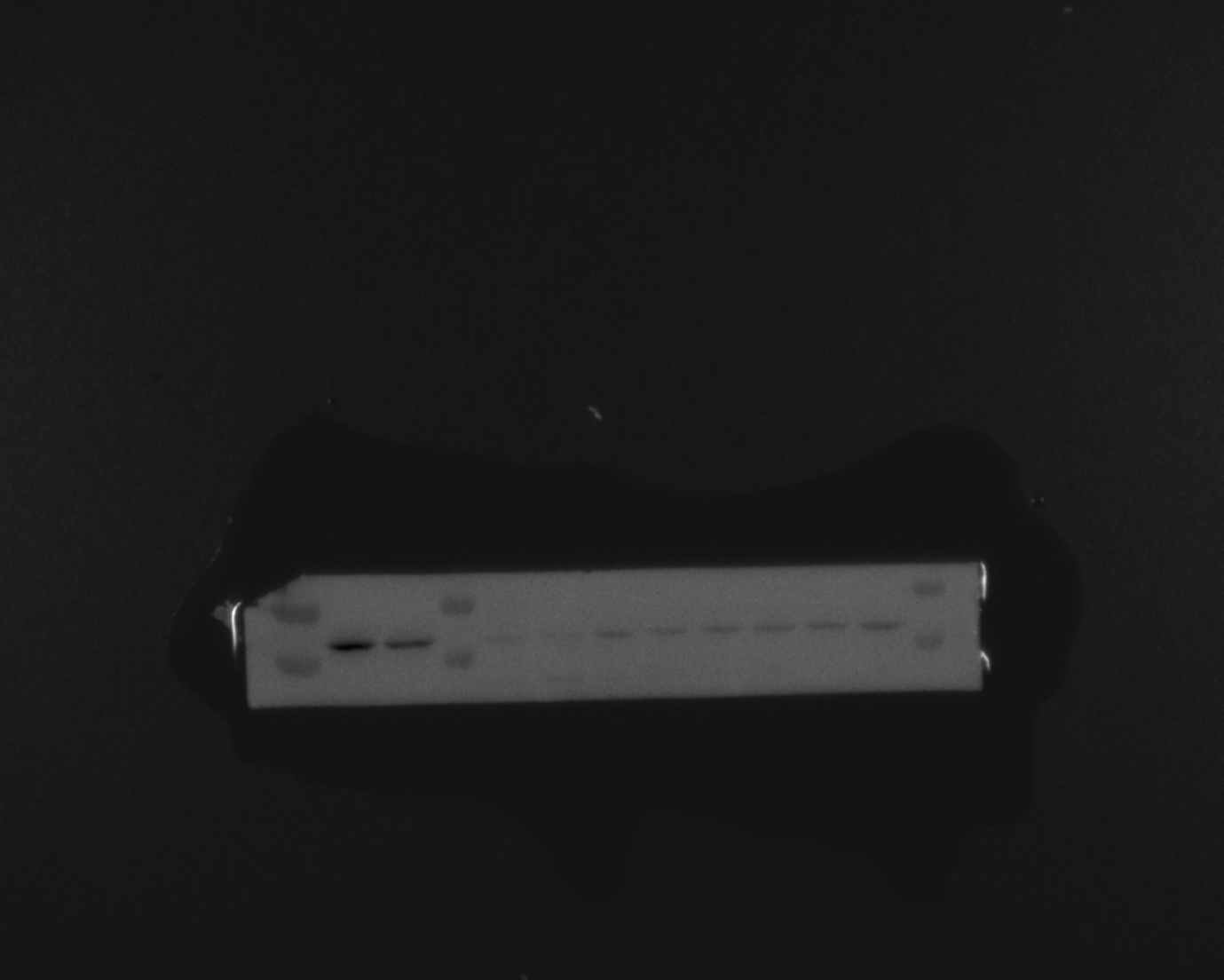

Supplement: Supplementary file 8 — EV Figure and Appendix Figure Source Data [file 44321_2025_200_MOESM8_ESM.zip › Fig. EV4/Fig. EV4F/STUB1-1-130-Actin.tif]

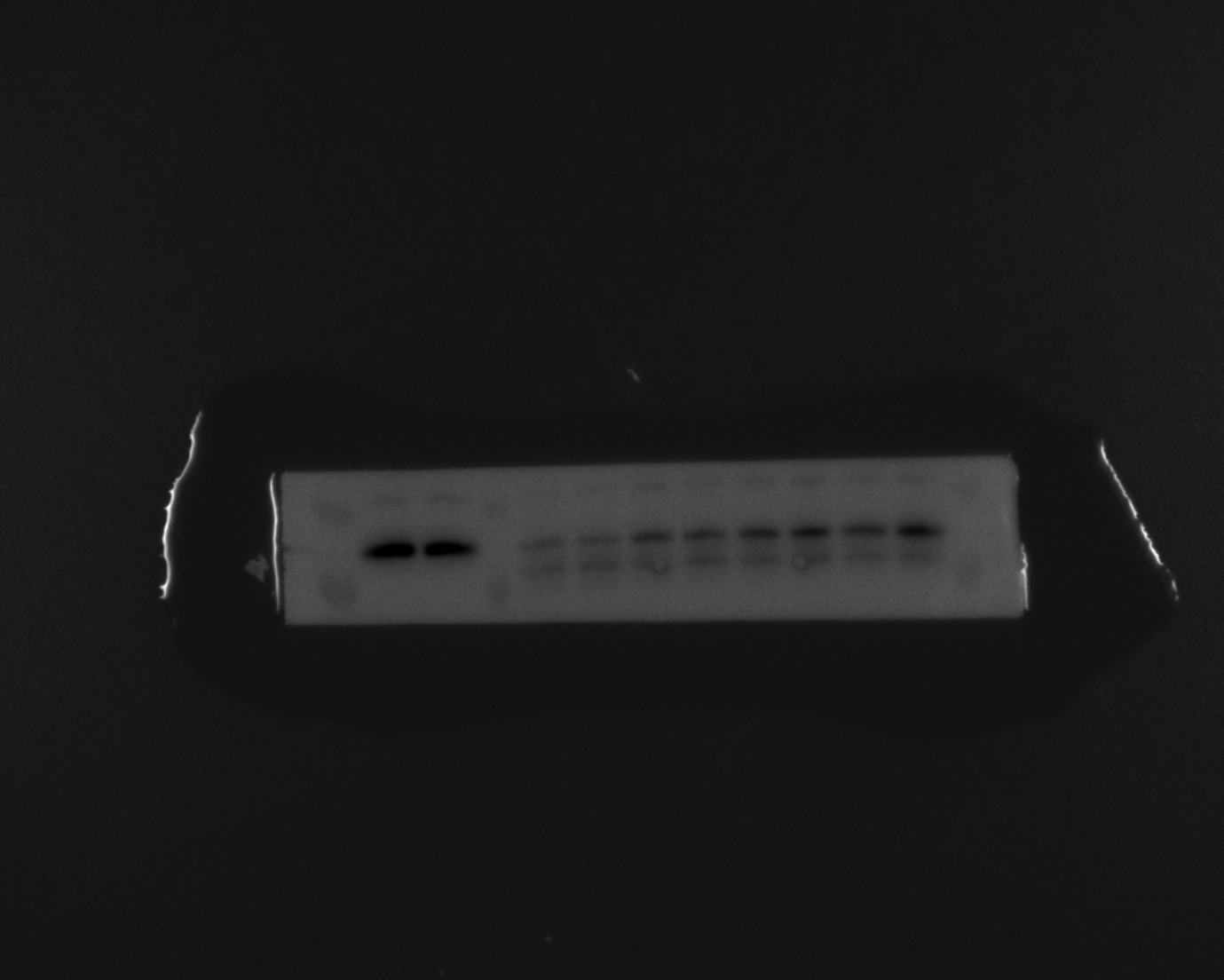

Supplement: Supplementary file 8 — EV Figure and Appendix Figure Source Data [file 44321_2025_200_MOESM8_ESM.zip › Fig. EV4/Fig. EV4F/STUB1-1-130-Flag.tif]

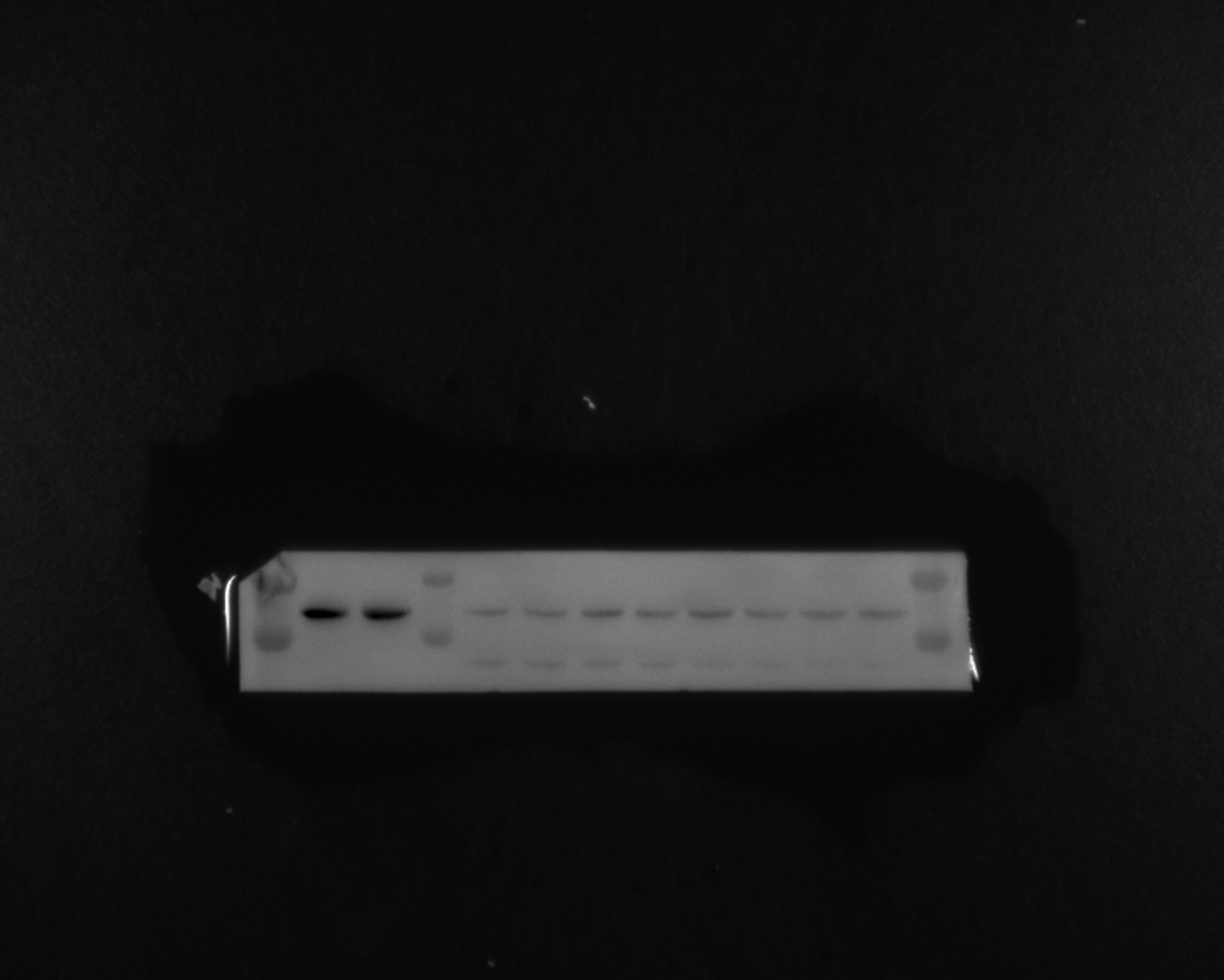

Supplement: Supplementary file 8 — EV Figure and Appendix Figure Source Data [file 44321_2025_200_MOESM8_ESM.zip › Fig. EV4/Fig. EV4F/STUB1-1-130-K72A-Actin.tif]

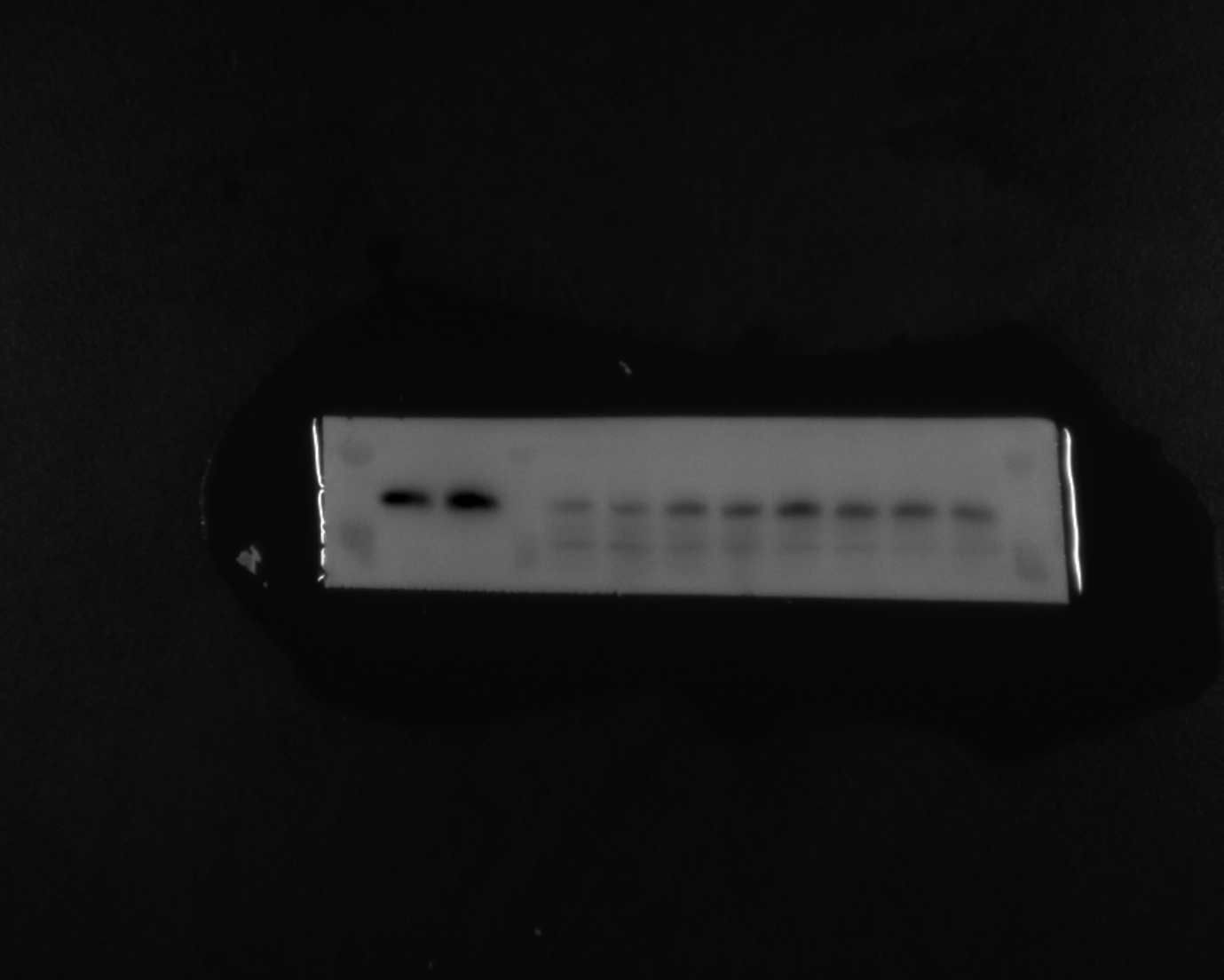

Supplement: Supplementary file 8 — EV Figure and Appendix Figure Source Data [file 44321_2025_200_MOESM8_ESM.zip › Fig. EV4/Fig. EV4F/STUB1-1-130-K72A-Flag.tif]

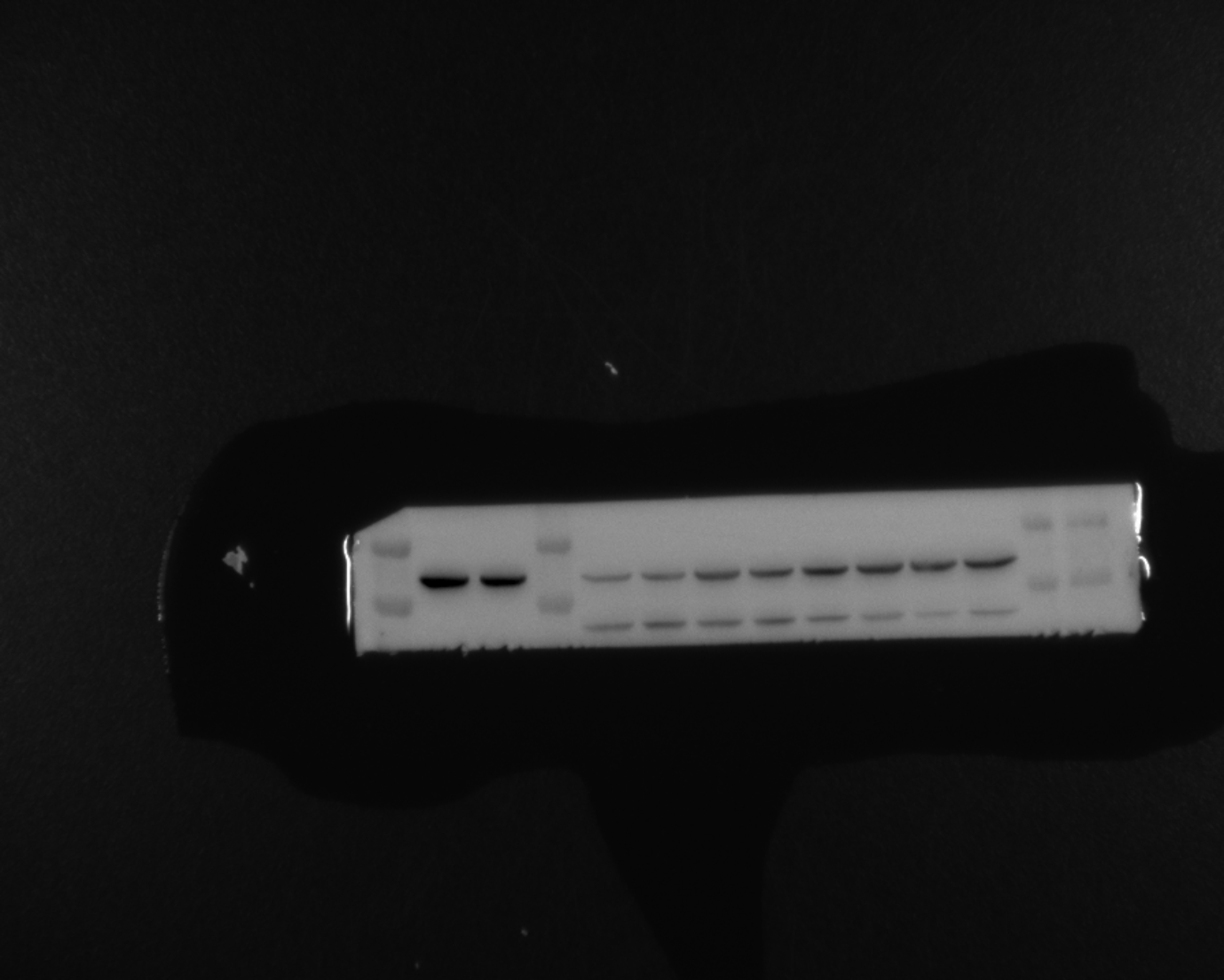

Supplement: Supplementary file 8 — EV Figure and Appendix Figure Source Data [file 44321_2025_200_MOESM8_ESM.zip › Fig. EV4/Fig. EV4F/STUB1-1-130-Q102A-Actin.tif]

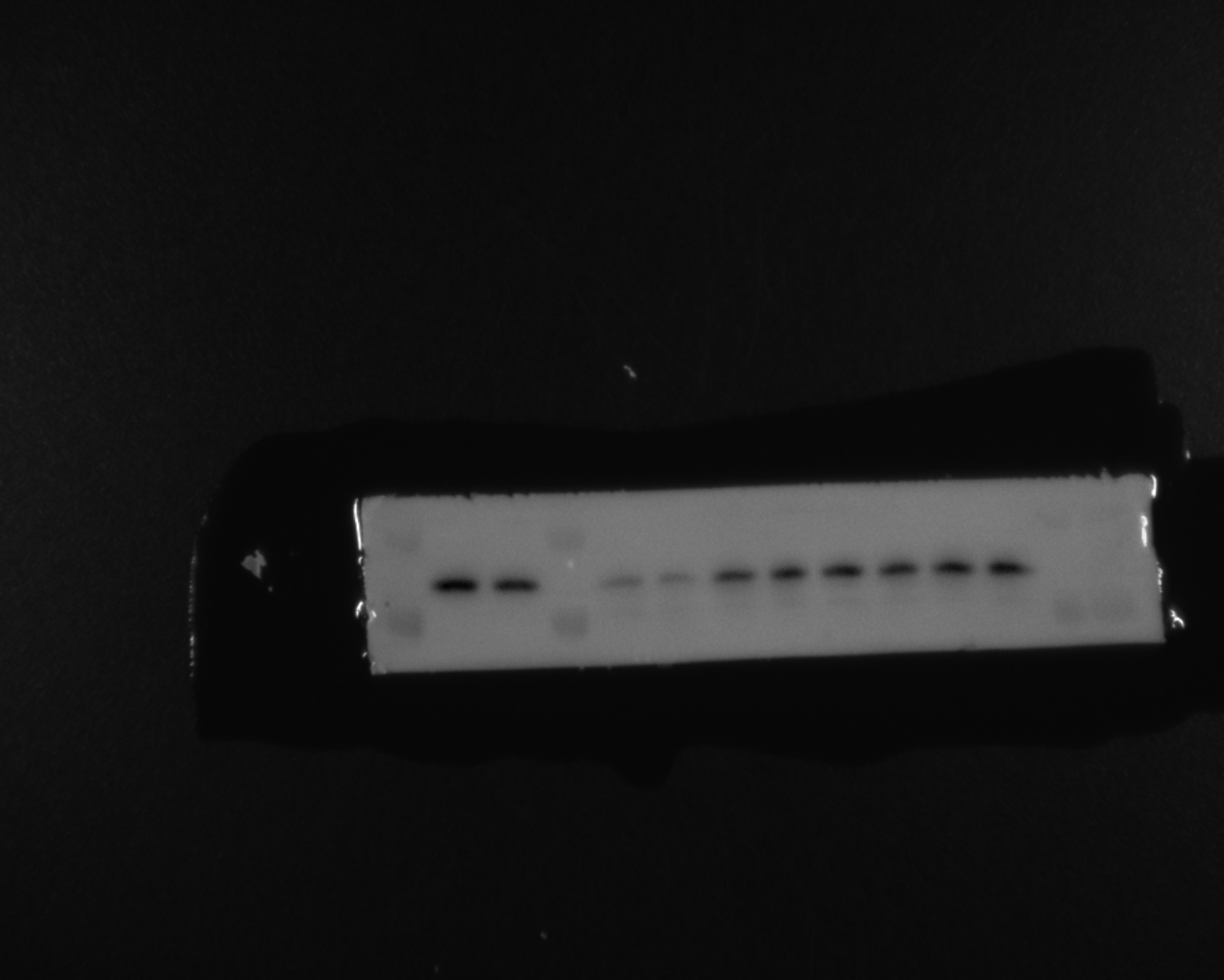

Supplement: Supplementary file 8 — EV Figure and Appendix Figure Source Data [file 44321_2025_200_MOESM8_ESM.zip › Fig. EV4/Fig. EV4F/STUB1-1-130-Q102A-Flag.tif]

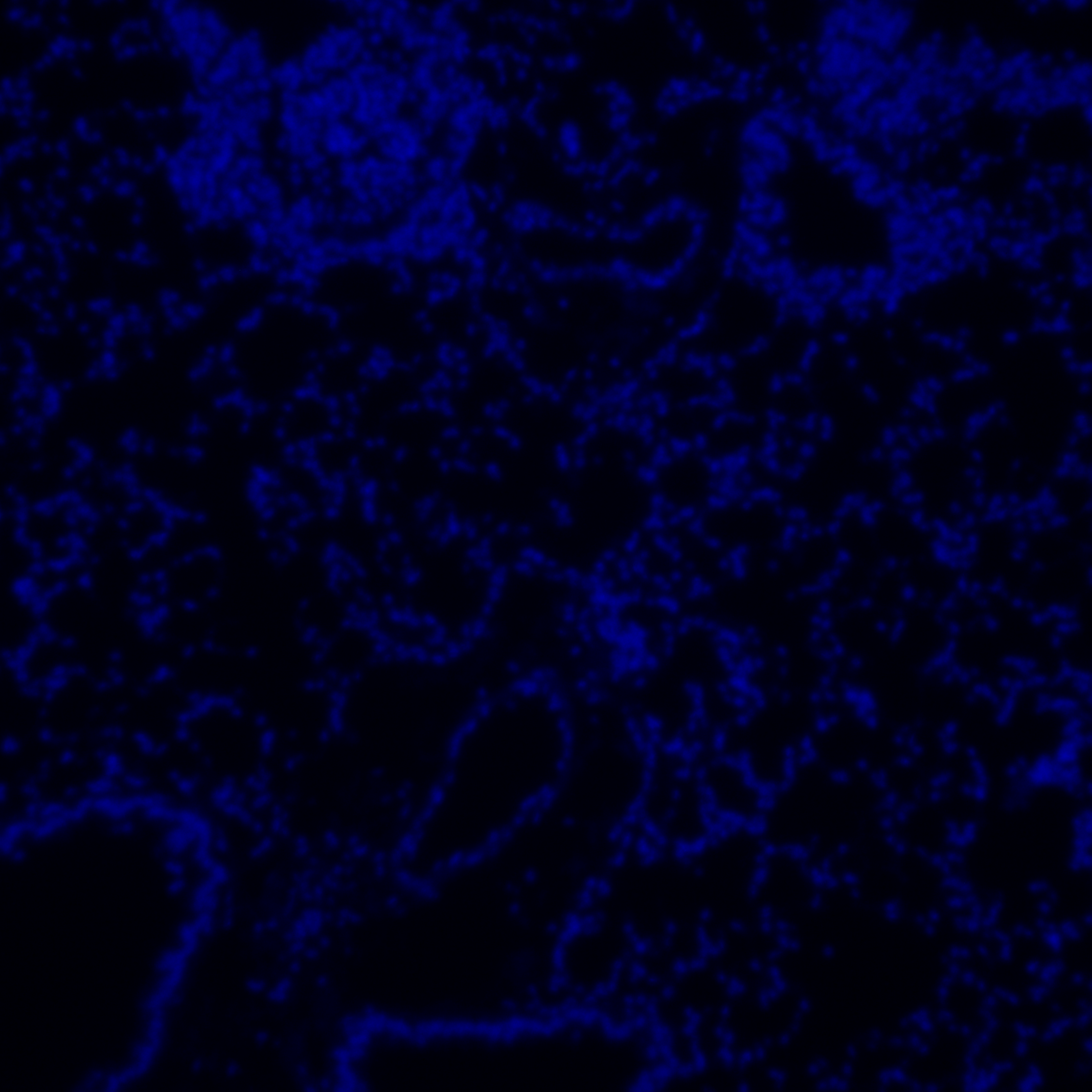

Supplement: Supplementary file 8 — EV Figure and Appendix Figure Source Data [file 44321_2025_200_MOESM8_ESM.zip › Fig. EV5/Fig. EV5B/Combo--DAPI.tif]

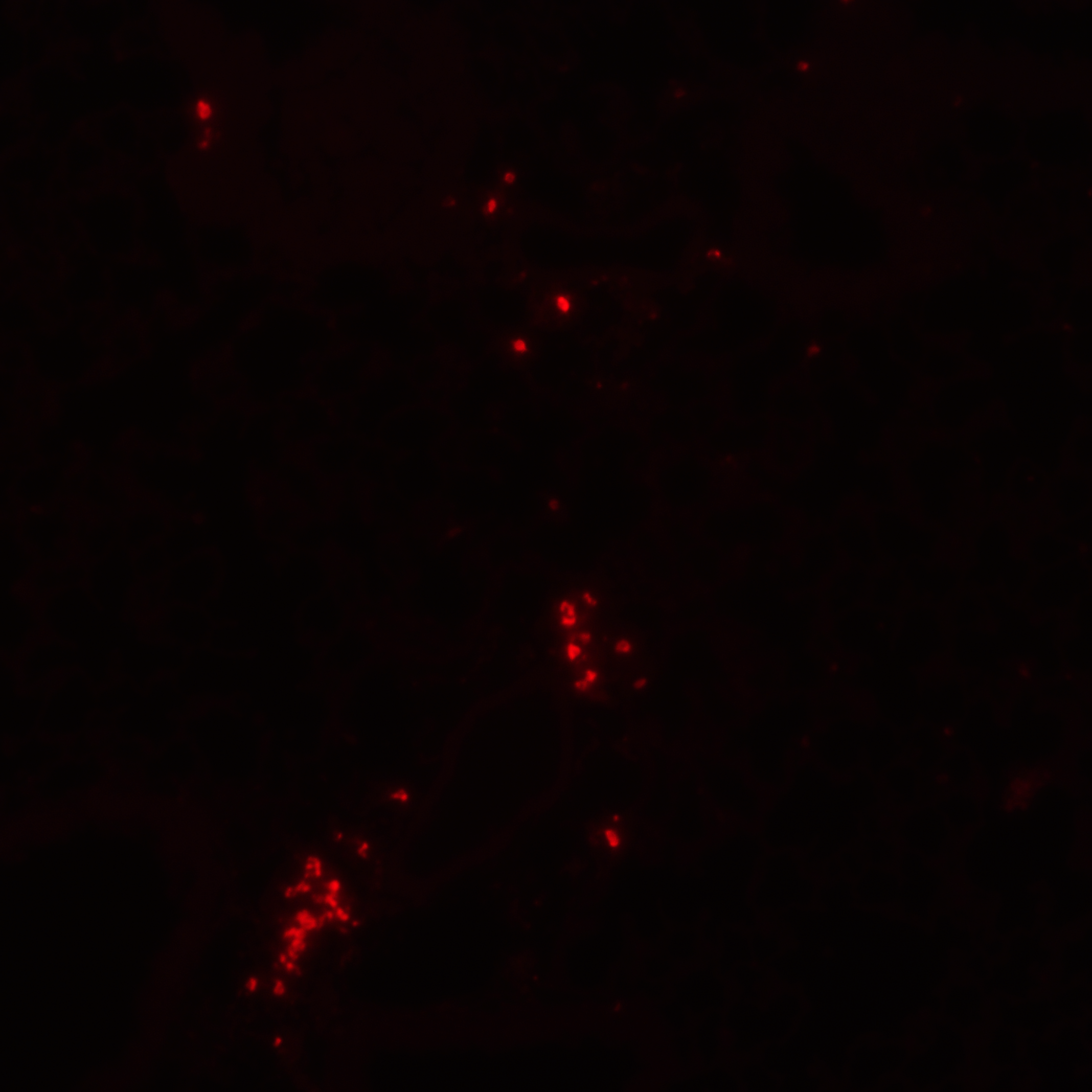

Supplement: Supplementary file 8 — EV Figure and Appendix Figure Source Data [file 44321_2025_200_MOESM8_ESM.zip › Fig. EV5/Fig. EV5B/Combo-CD4.tif]

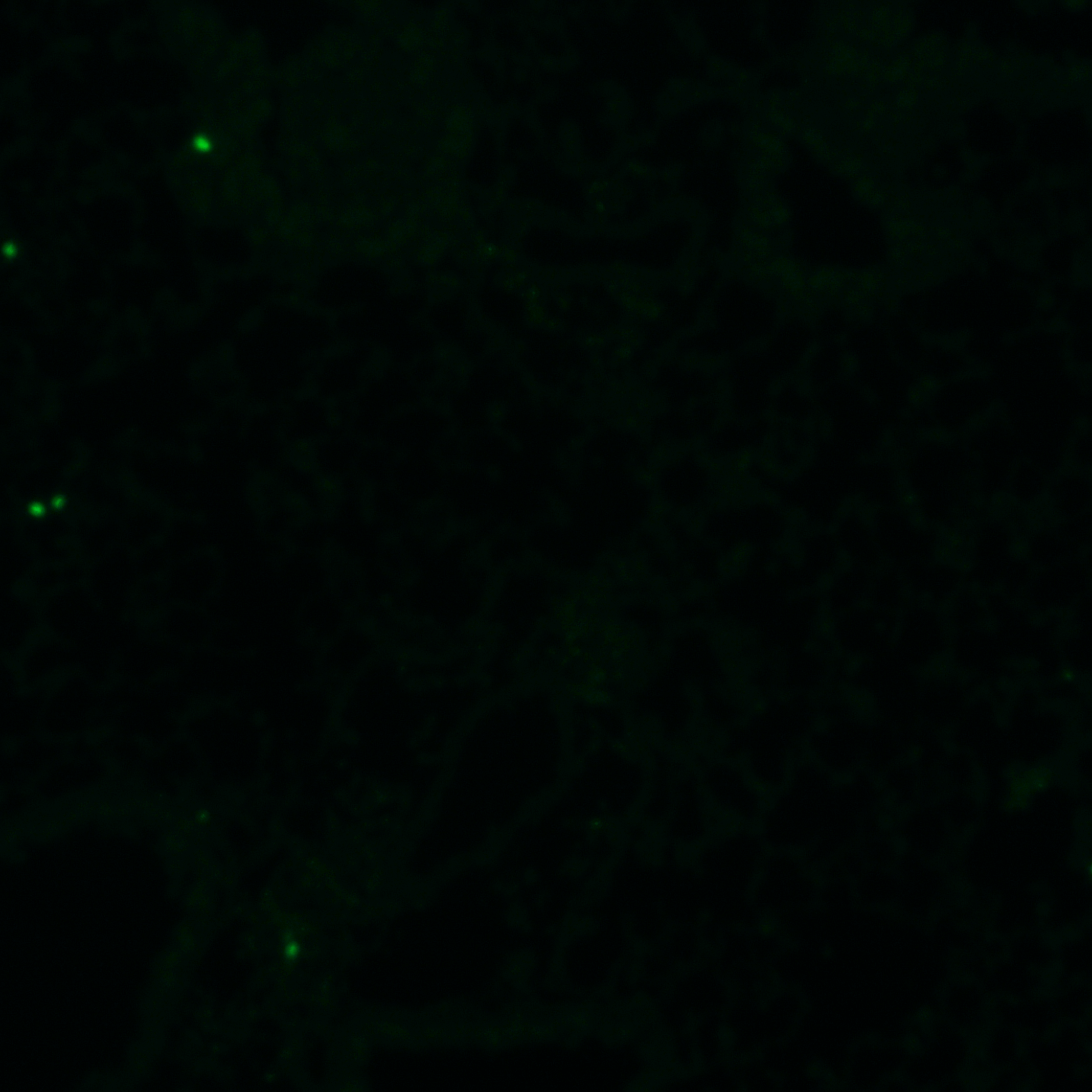

Supplement: Supplementary file 8 — EV Figure and Appendix Figure Source Data [file 44321_2025_200_MOESM8_ESM.zip › Fig. EV5/Fig. EV5B/Combo-FOXP3.tif]

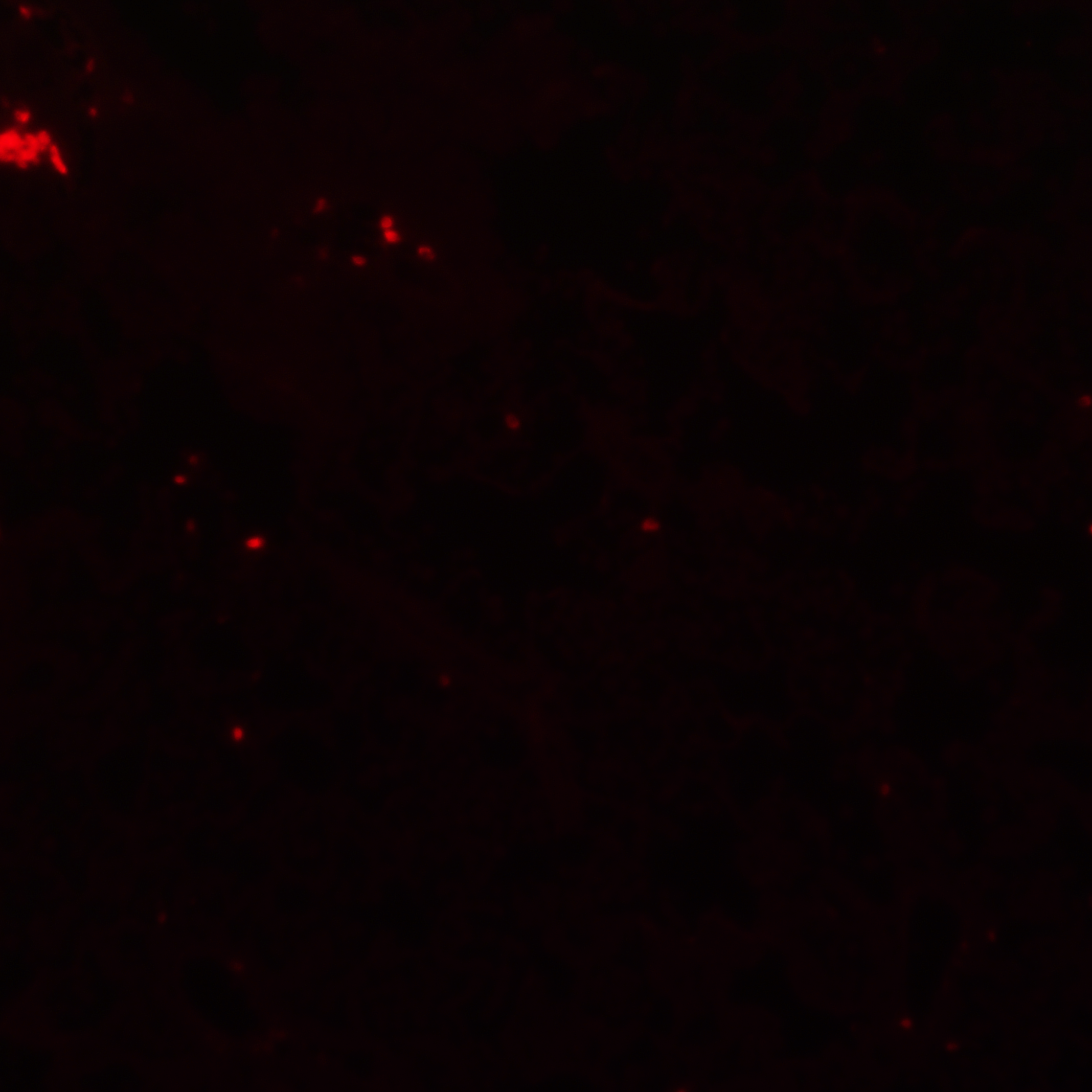

Supplement: Supplementary file 8 — EV Figure and Appendix Figure Source Data [file 44321_2025_200_MOESM8_ESM.zip › Fig. EV5/Fig. EV5B/LAC-CD4.tif]

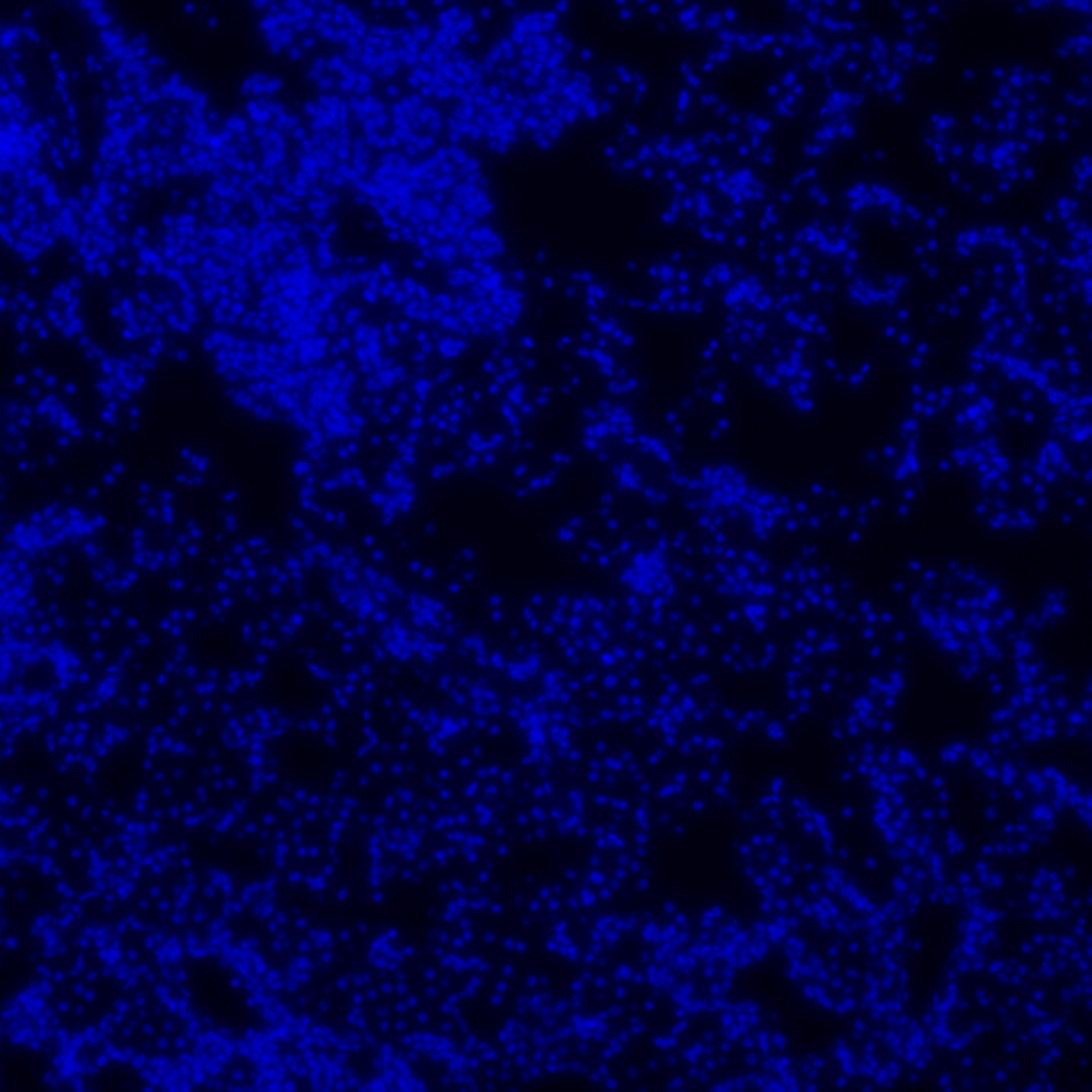

Supplement: Supplementary file 8 — EV Figure and Appendix Figure Source Data [file 44321_2025_200_MOESM8_ESM.zip › Fig. EV5/Fig. EV5B/LAC-DAPI.tif]

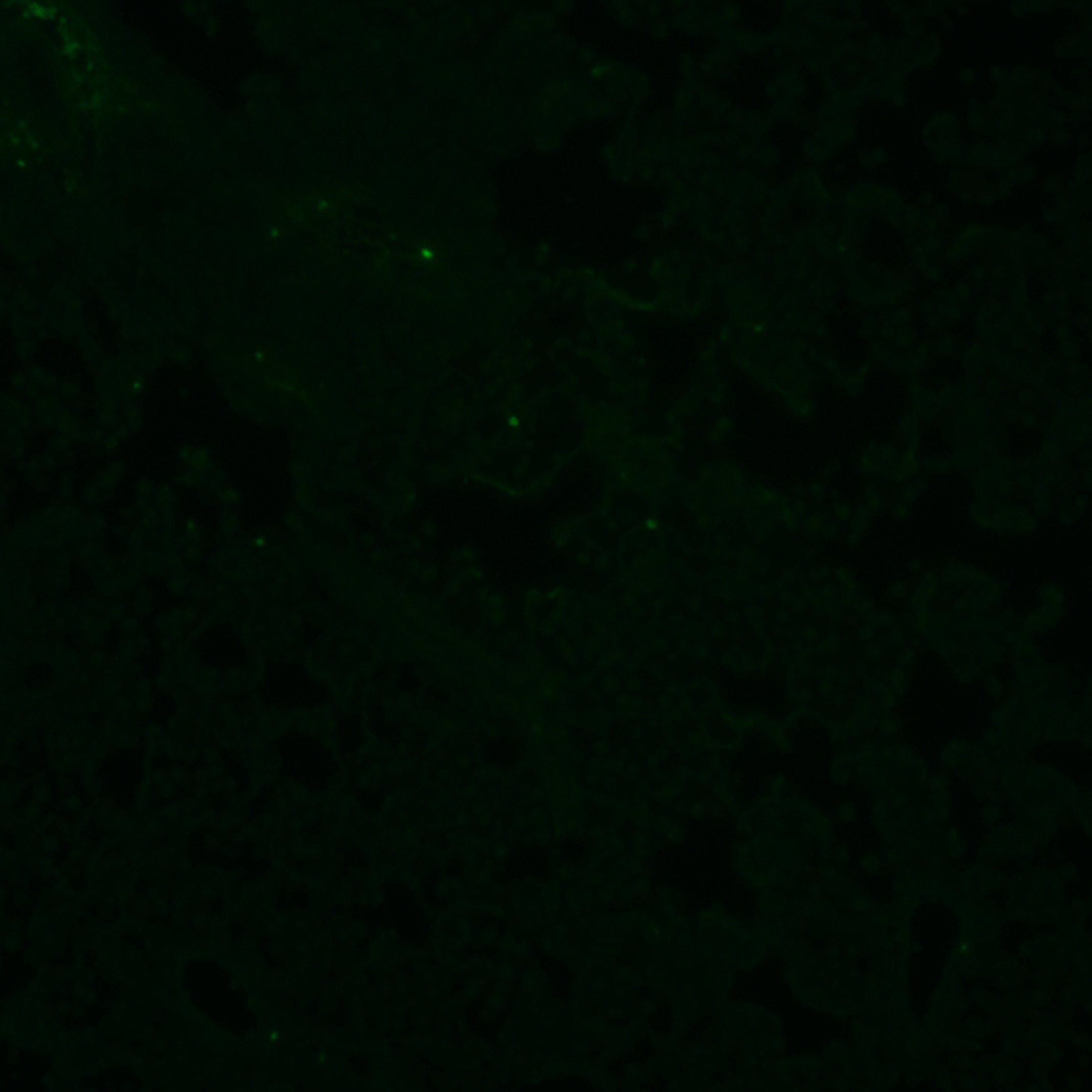

Supplement: Supplementary file 8 — EV Figure and Appendix Figure Source Data [file 44321_2025_200_MOESM8_ESM.zip › Fig. EV5/Fig. EV5B/LAC-FOXP3.tif]

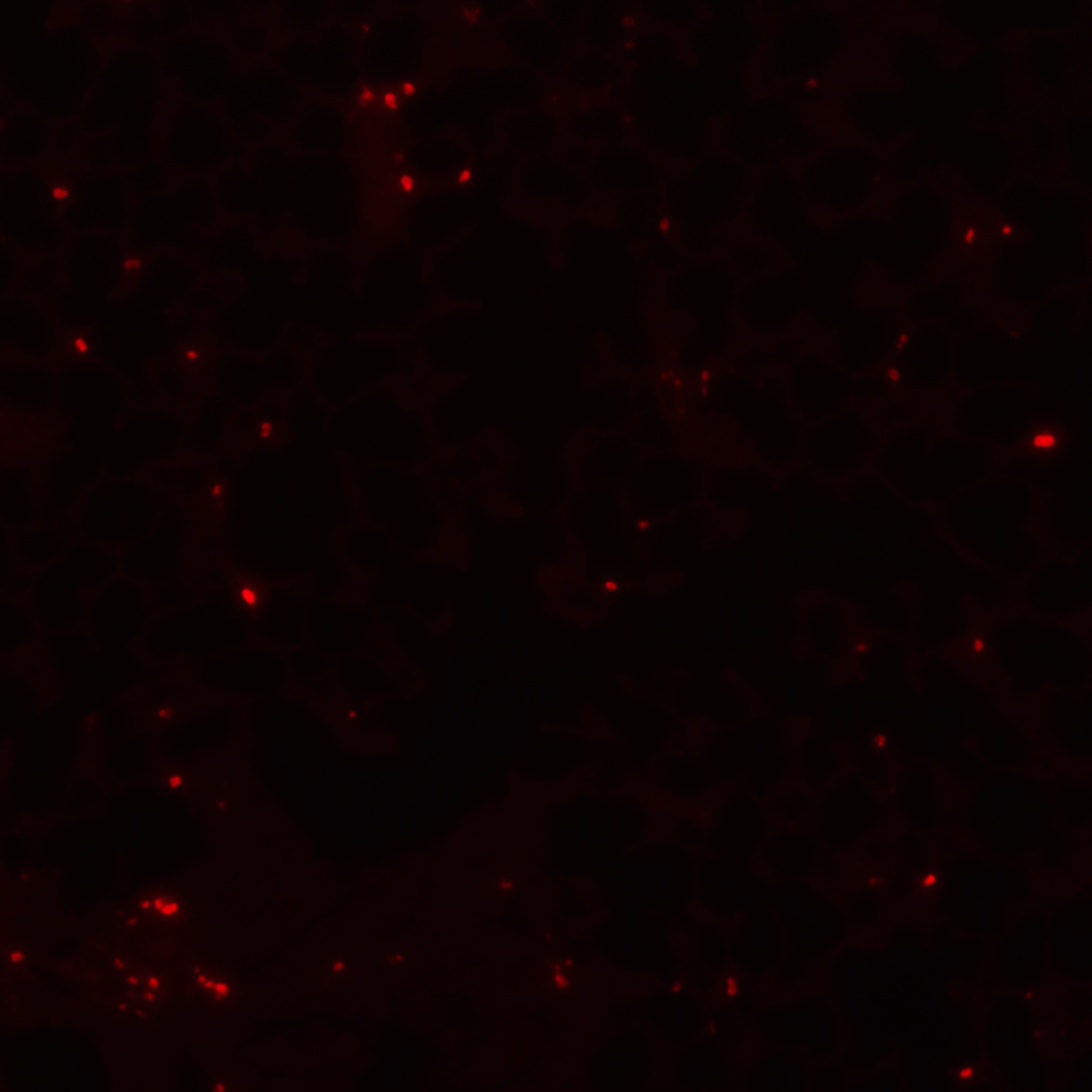

Supplement: Supplementary file 8 — EV Figure and Appendix Figure Source Data [file 44321_2025_200_MOESM8_ESM.zip › Fig. EV5/Fig. EV5B/PD1 Ab-CD4.tif]

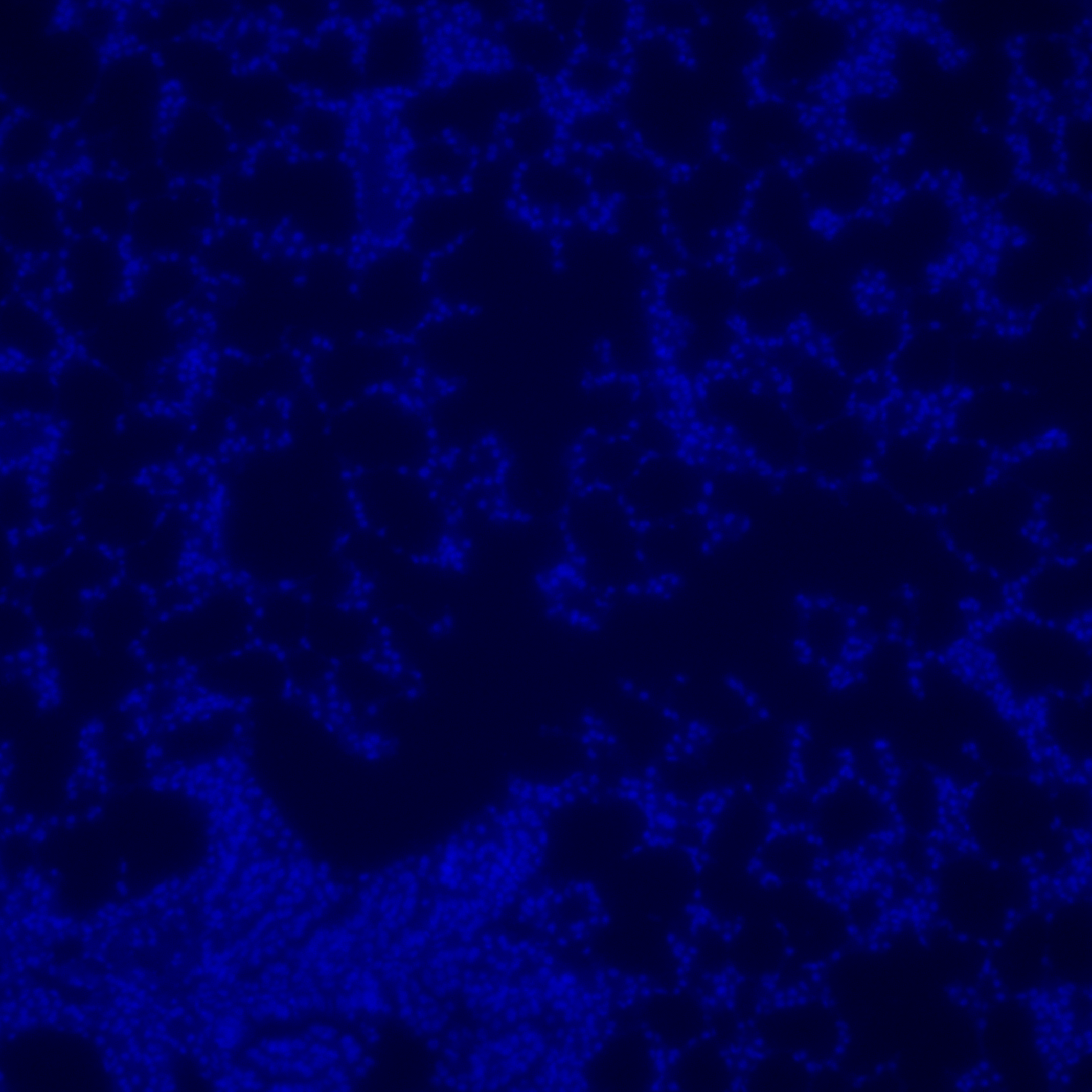

Supplement: Supplementary file 8 — EV Figure and Appendix Figure Source Data [file 44321_2025_200_MOESM8_ESM.zip › Fig. EV5/Fig. EV5B/PD1 Ab-DAPI.tif]

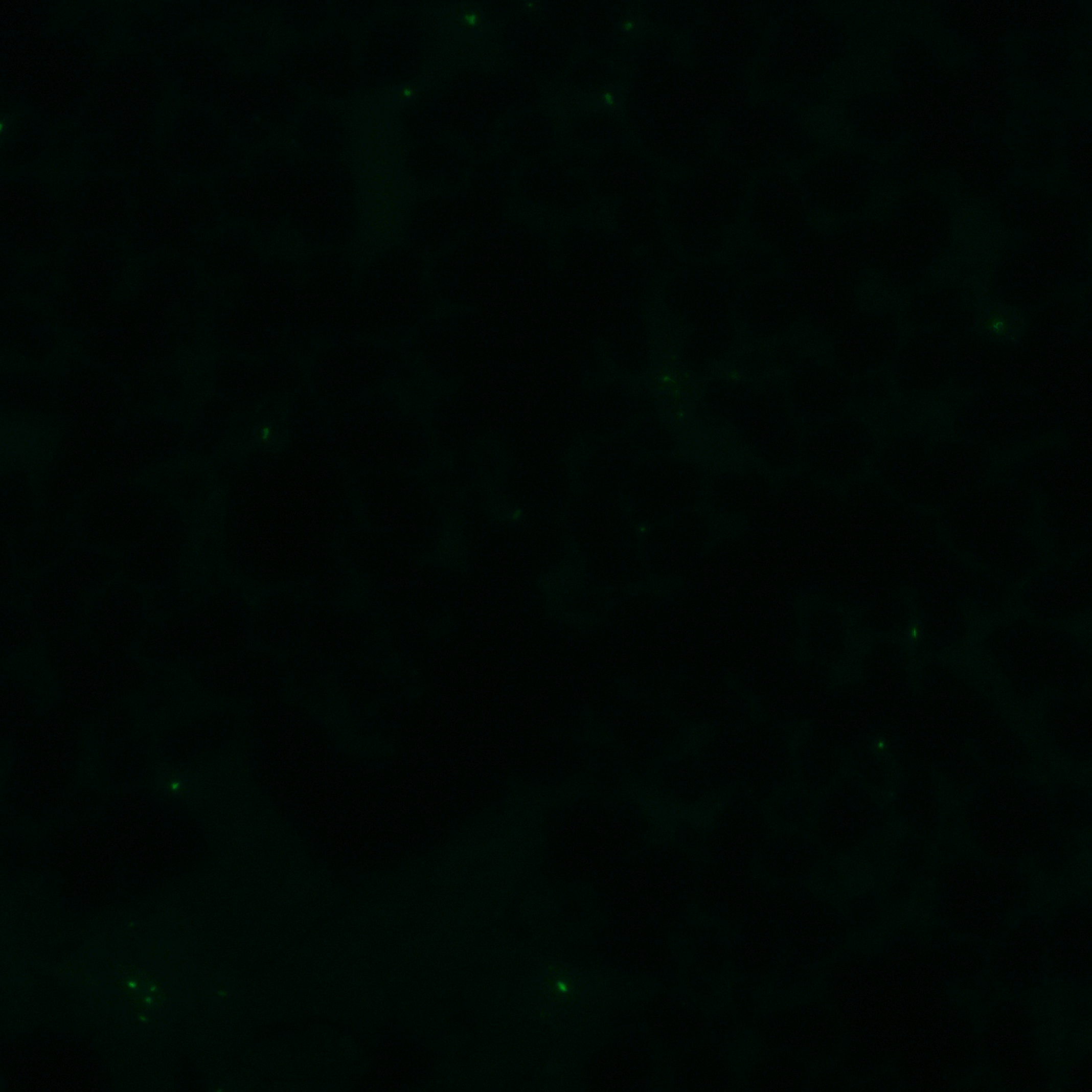

Supplement: Supplementary file 8 — EV Figure and Appendix Figure Source Data [file 44321_2025_200_MOESM8_ESM.zip › Fig. EV5/Fig. EV5B/PD1 Ab-FOXP3.tif]

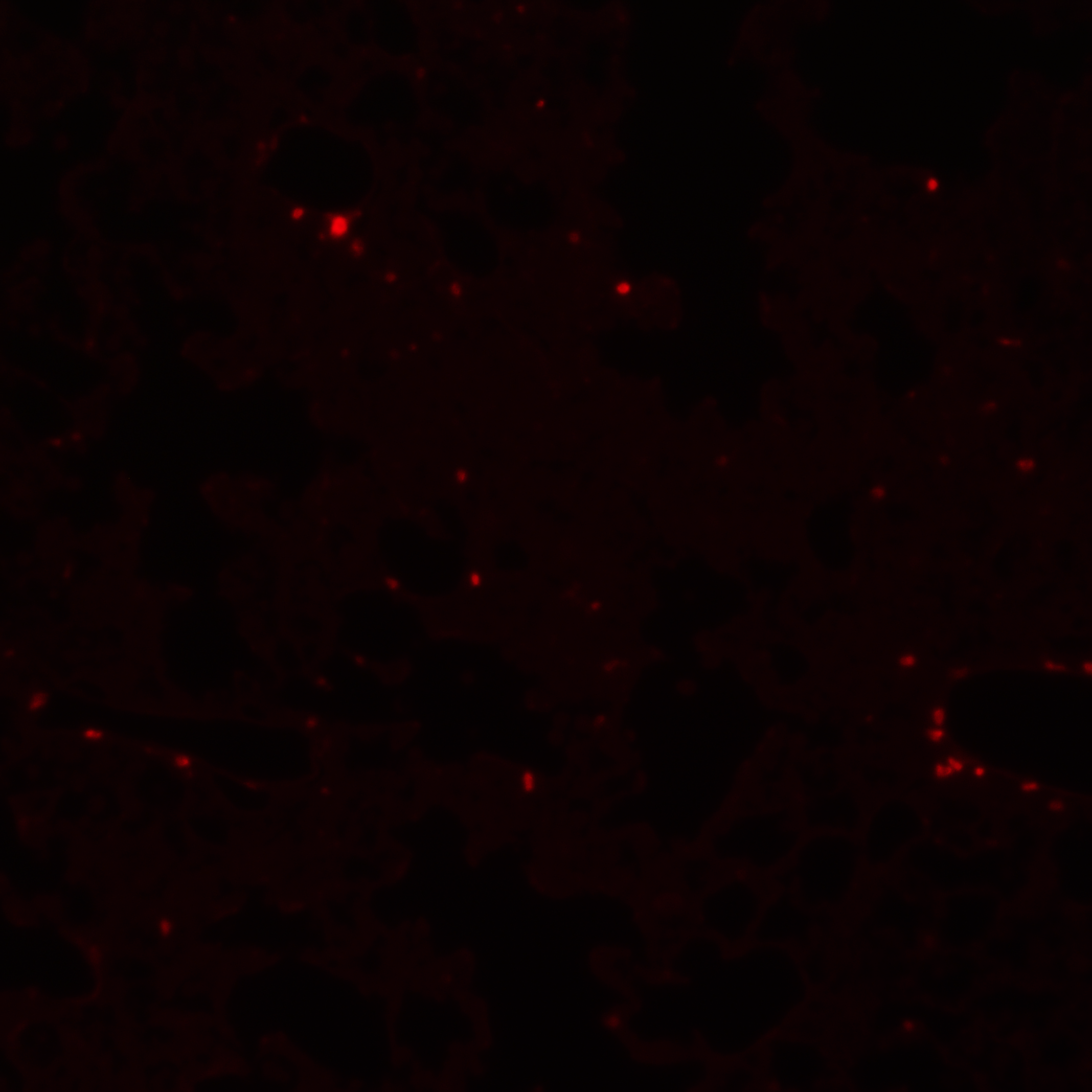

Supplement: Supplementary file 8 — EV Figure and Appendix Figure Source Data [file 44321_2025_200_MOESM8_ESM.zip › Fig. EV5/Fig. EV5B/Veh.-CD4.tif]

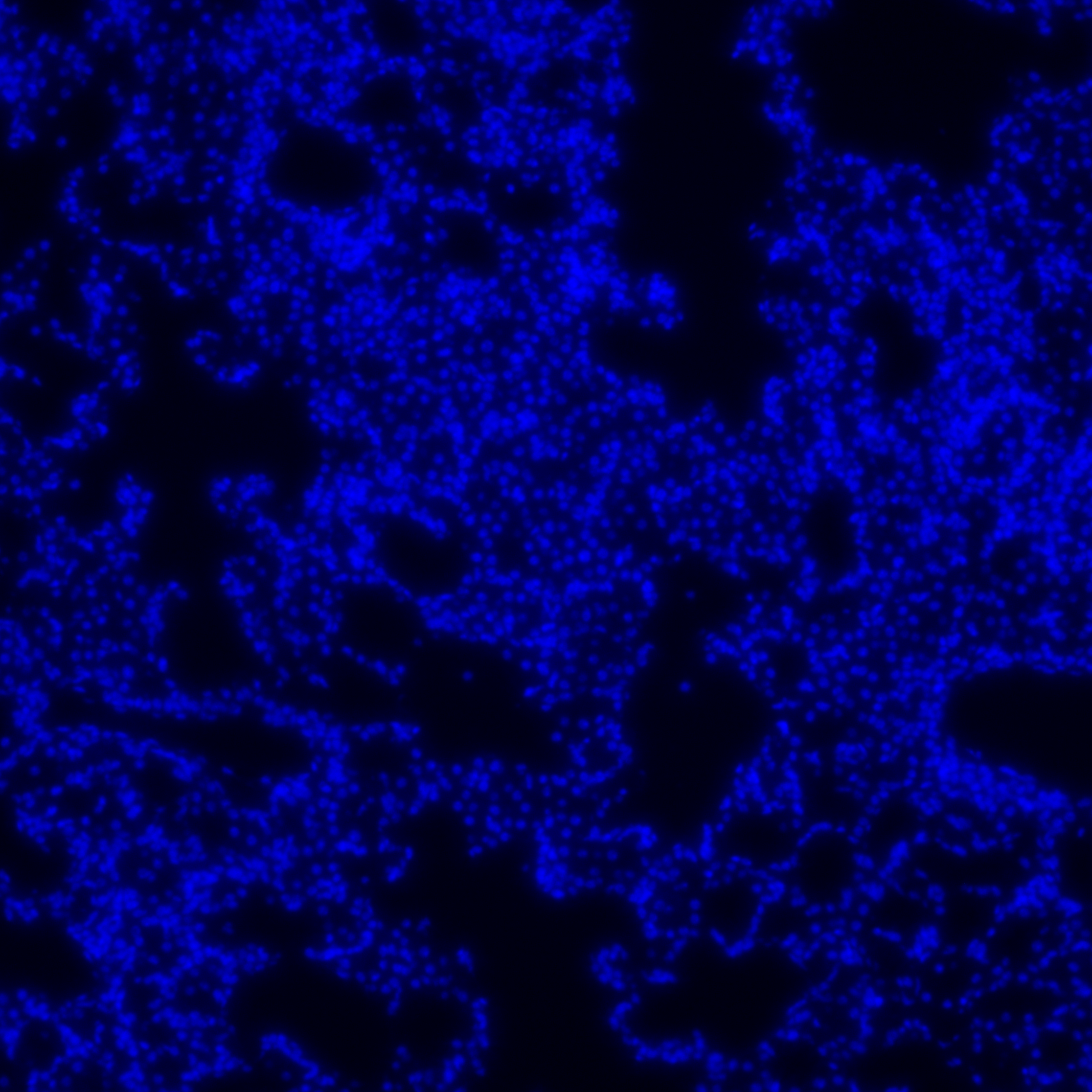

Supplement: Supplementary file 8 — EV Figure and Appendix Figure Source Data [file 44321_2025_200_MOESM8_ESM.zip › Fig. EV5/Fig. EV5B/Veh.-DAPI.tif]

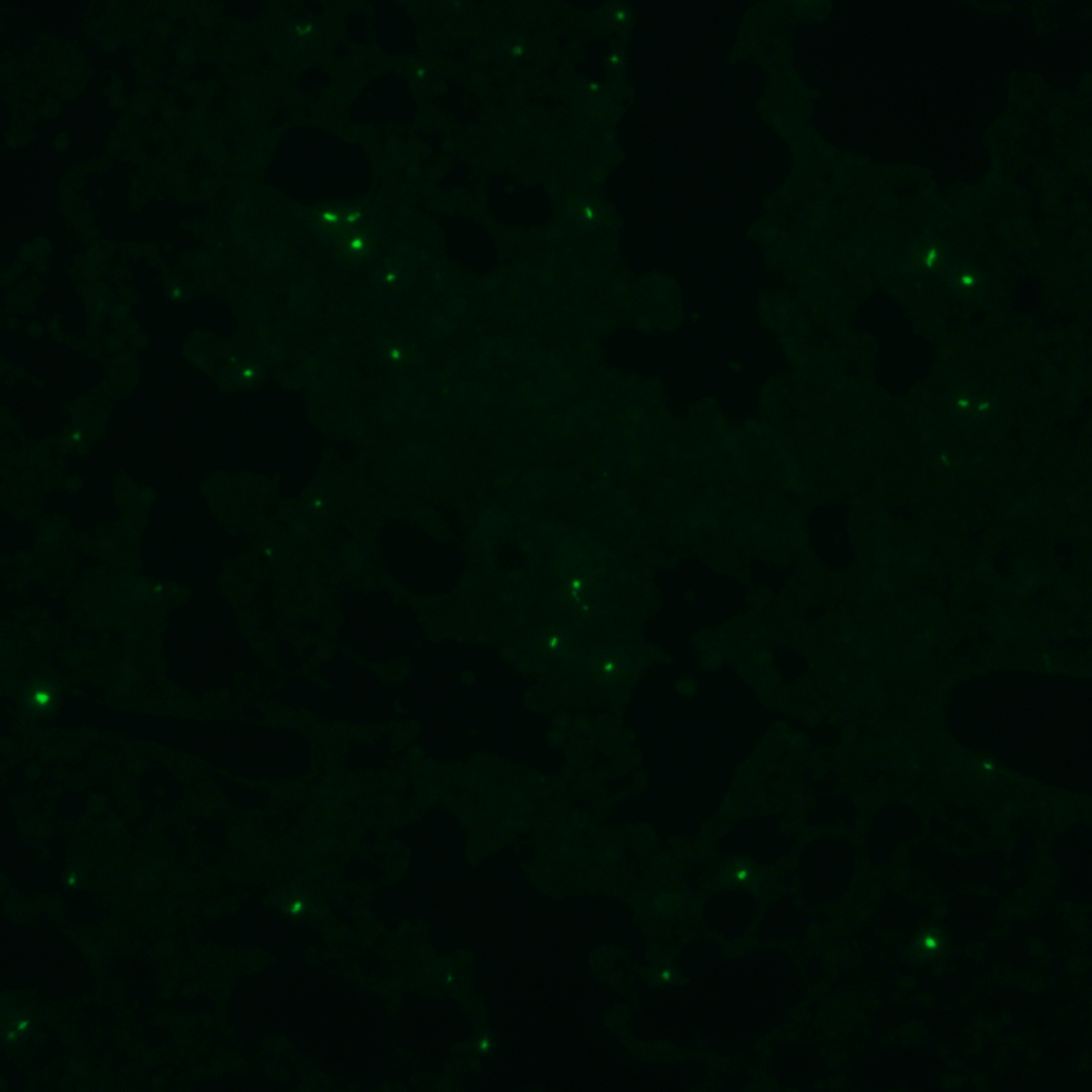

Supplement: Supplementary file 8 — EV Figure and Appendix Figure Source Data [file 44321_2025_200_MOESM8_ESM.zip › Fig. EV5/Fig. EV5B/Veh.-FOXP3.tif]

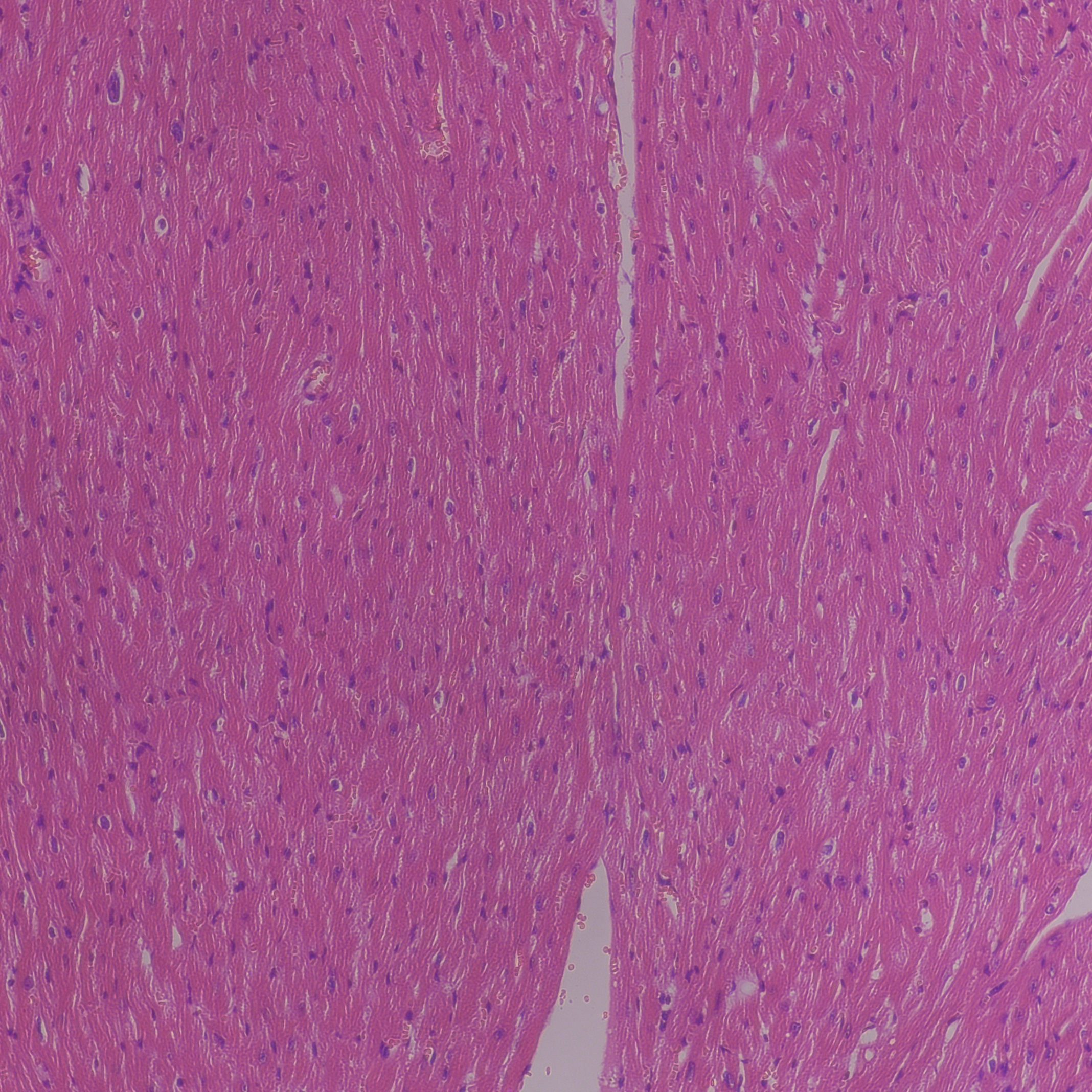

Supplement: Supplementary file 8 — EV Figure and Appendix Figure Source Data [file 44321_2025_200_MOESM8_ESM.zip › Fig. EV5/Fig. EV5H/heart-Combo.tif]

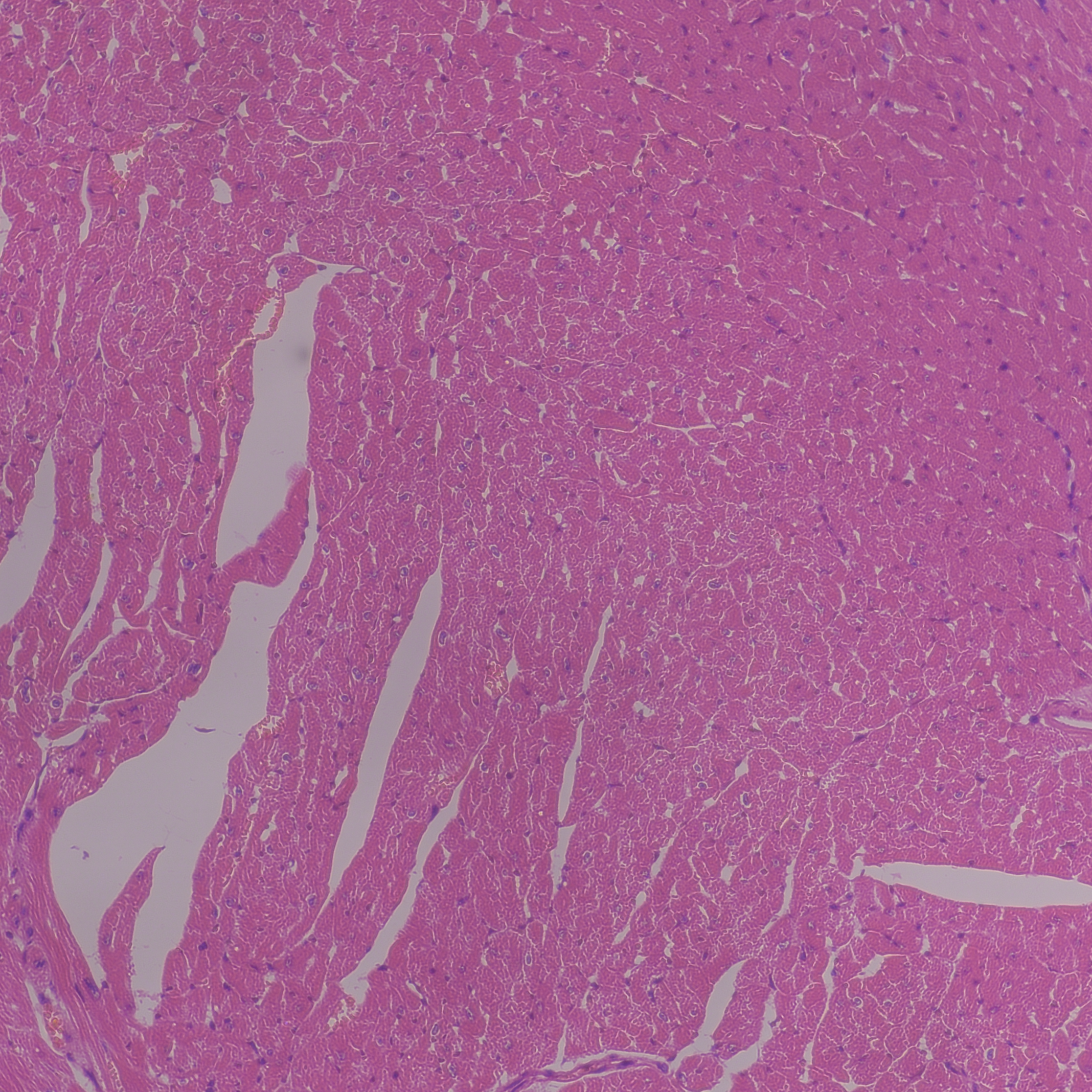

Supplement: Supplementary file 8 — EV Figure and Appendix Figure Source Data [file 44321_2025_200_MOESM8_ESM.zip › Fig. EV5/Fig. EV5H/heart-Lac.tif]

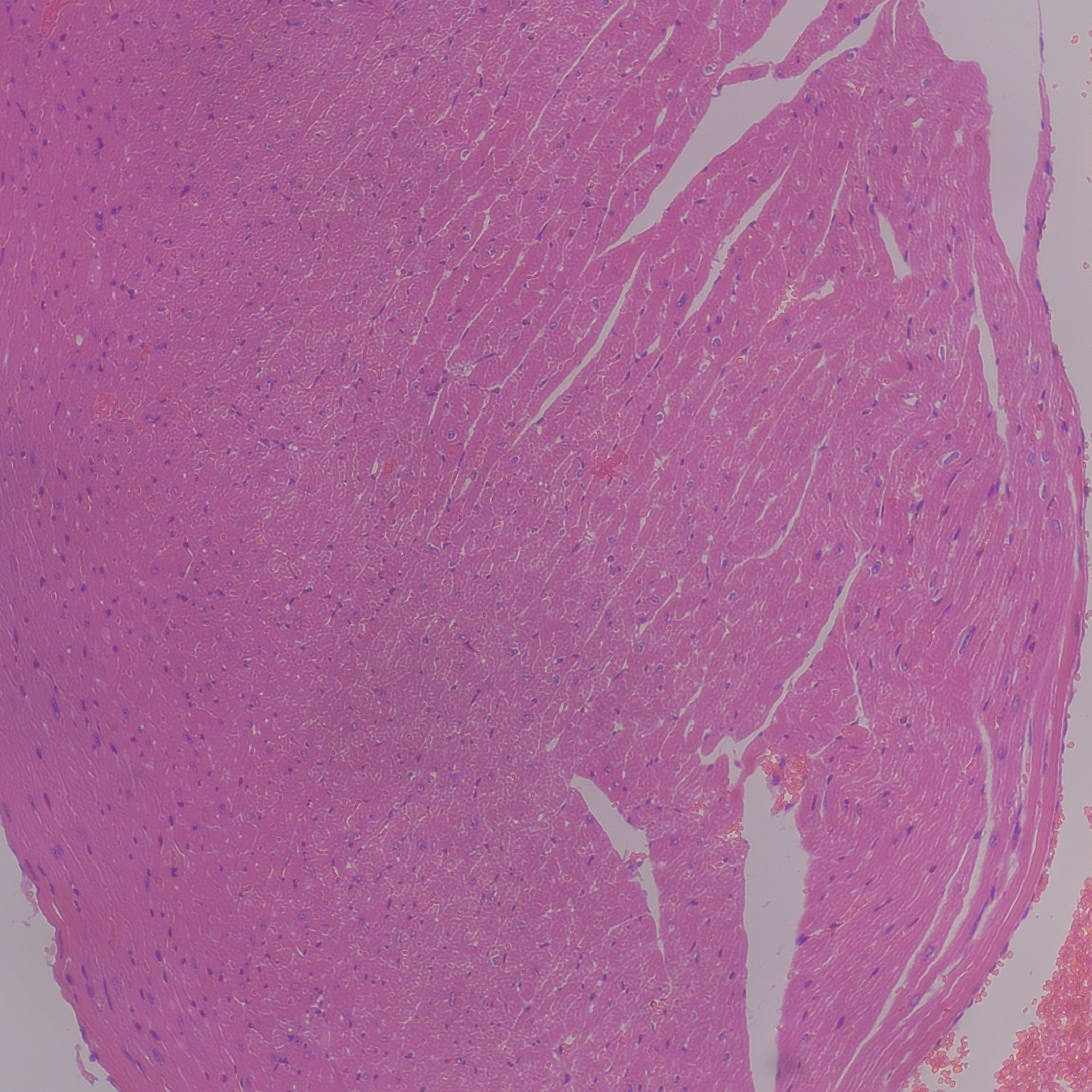

Supplement: Supplementary file 8 — EV Figure and Appendix Figure Source Data [file 44321_2025_200_MOESM8_ESM.zip › Fig. EV5/Fig. EV5H/heart-PD1 Ab.tif]

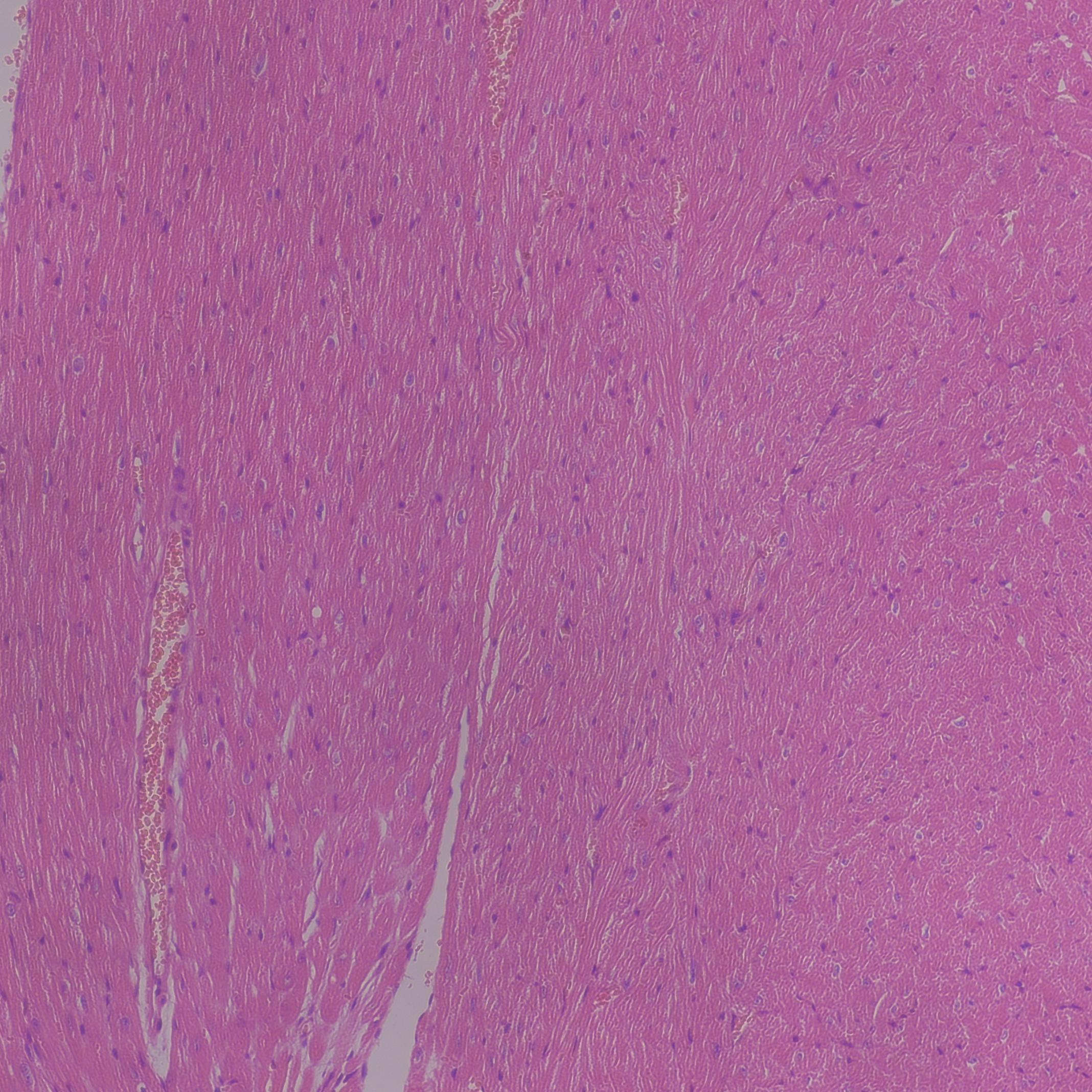

Supplement: Supplementary file 8 — EV Figure and Appendix Figure Source Data [file 44321_2025_200_MOESM8_ESM.zip › Fig. EV5/Fig. EV5H/heart-Veh..tif]

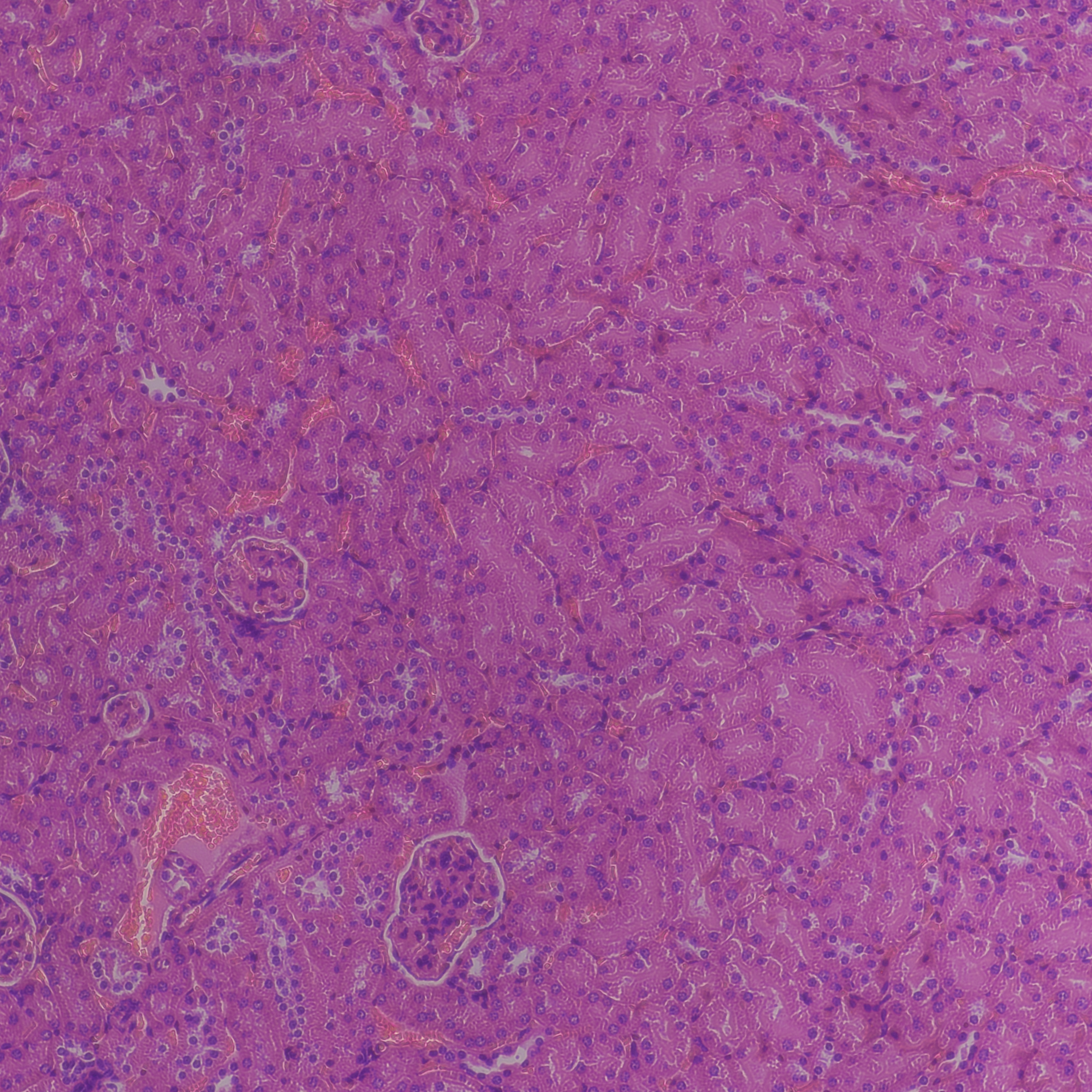

Supplement: Supplementary file 8 — EV Figure and Appendix Figure Source Data [file 44321_2025_200_MOESM8_ESM.zip › Fig. EV5/Fig. EV5H/Kidney-Combo.tif]

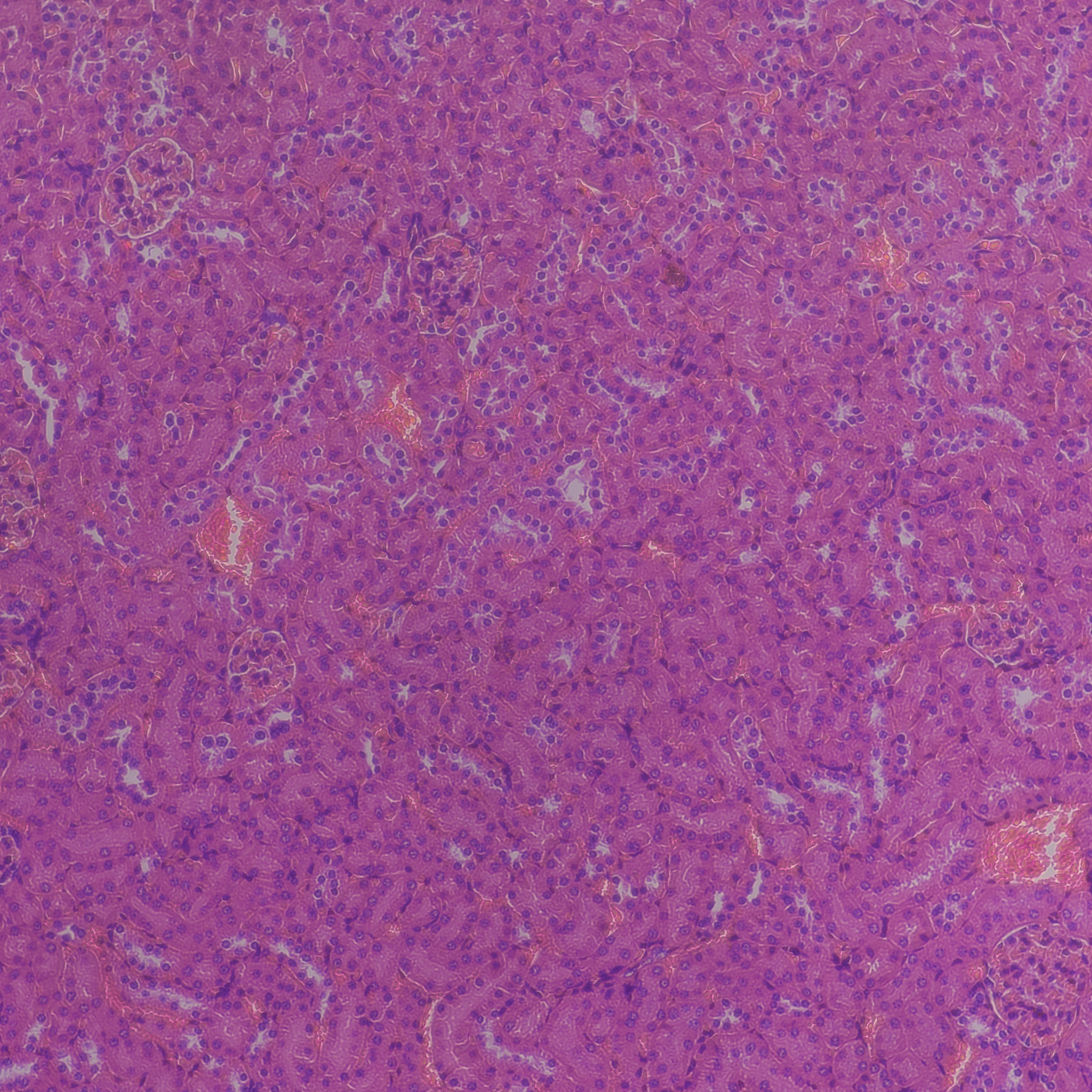

Supplement: Supplementary file 8 — EV Figure and Appendix Figure Source Data [file 44321_2025_200_MOESM8_ESM.zip › Fig. EV5/Fig. EV5H/Kidney-Lac.tif]

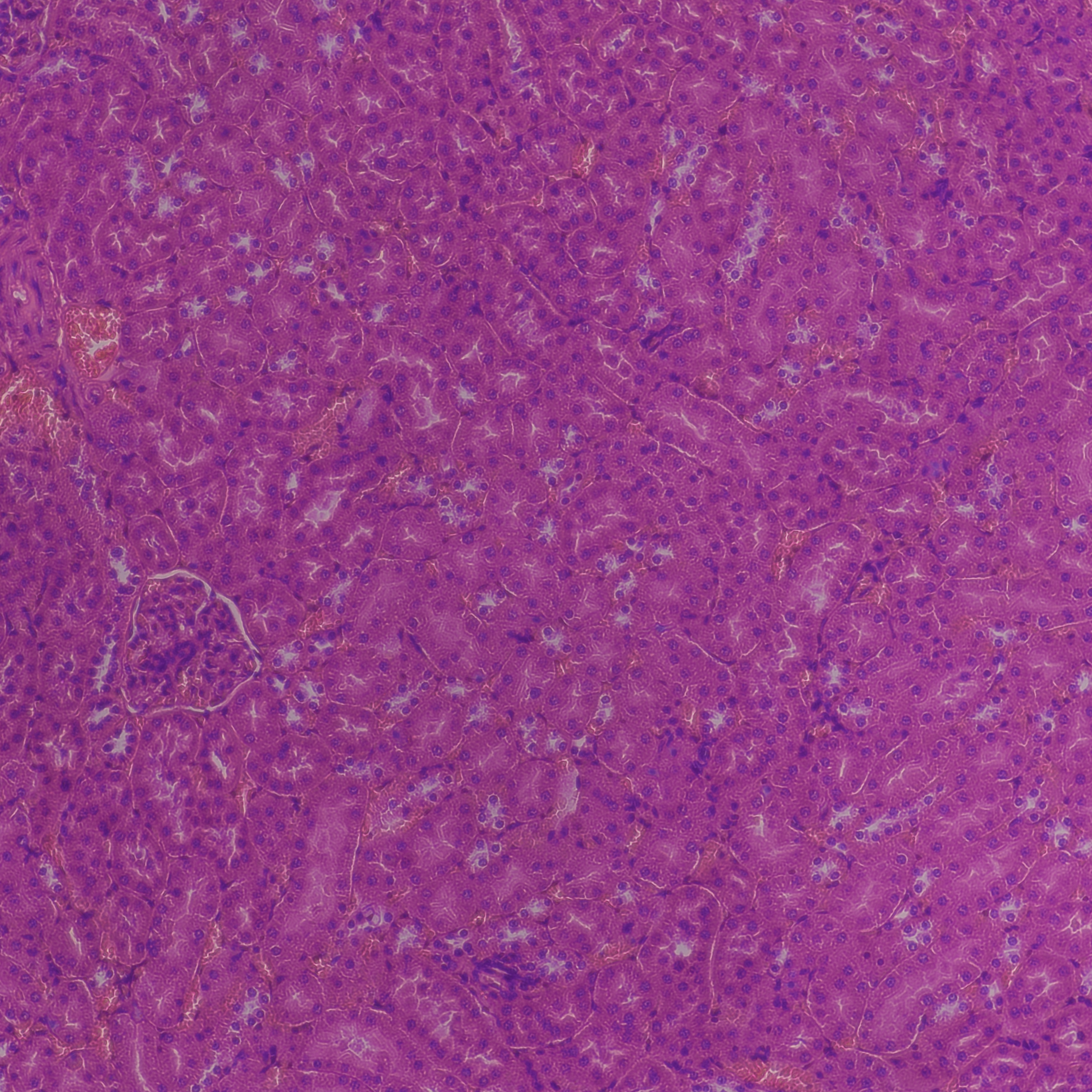

Supplement: Supplementary file 8 — EV Figure and Appendix Figure Source Data [file 44321_2025_200_MOESM8_ESM.zip › Fig. EV5/Fig. EV5H/Kidney-PD1 Ab.tif]

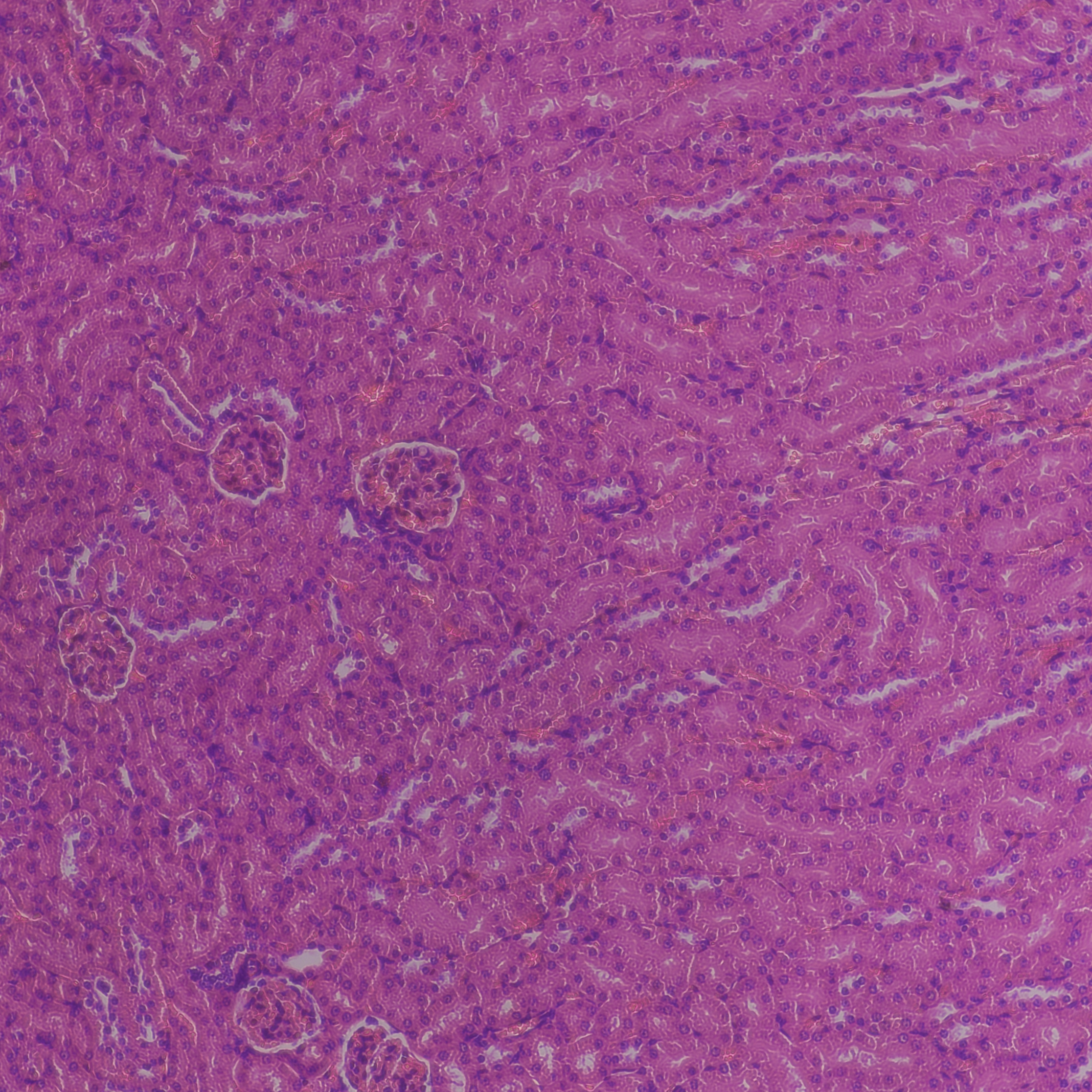

Supplement: Supplementary file 8 — EV Figure and Appendix Figure Source Data [file 44321_2025_200_MOESM8_ESM.zip › Fig. EV5/Fig. EV5H/Kidney-Veh.tif]

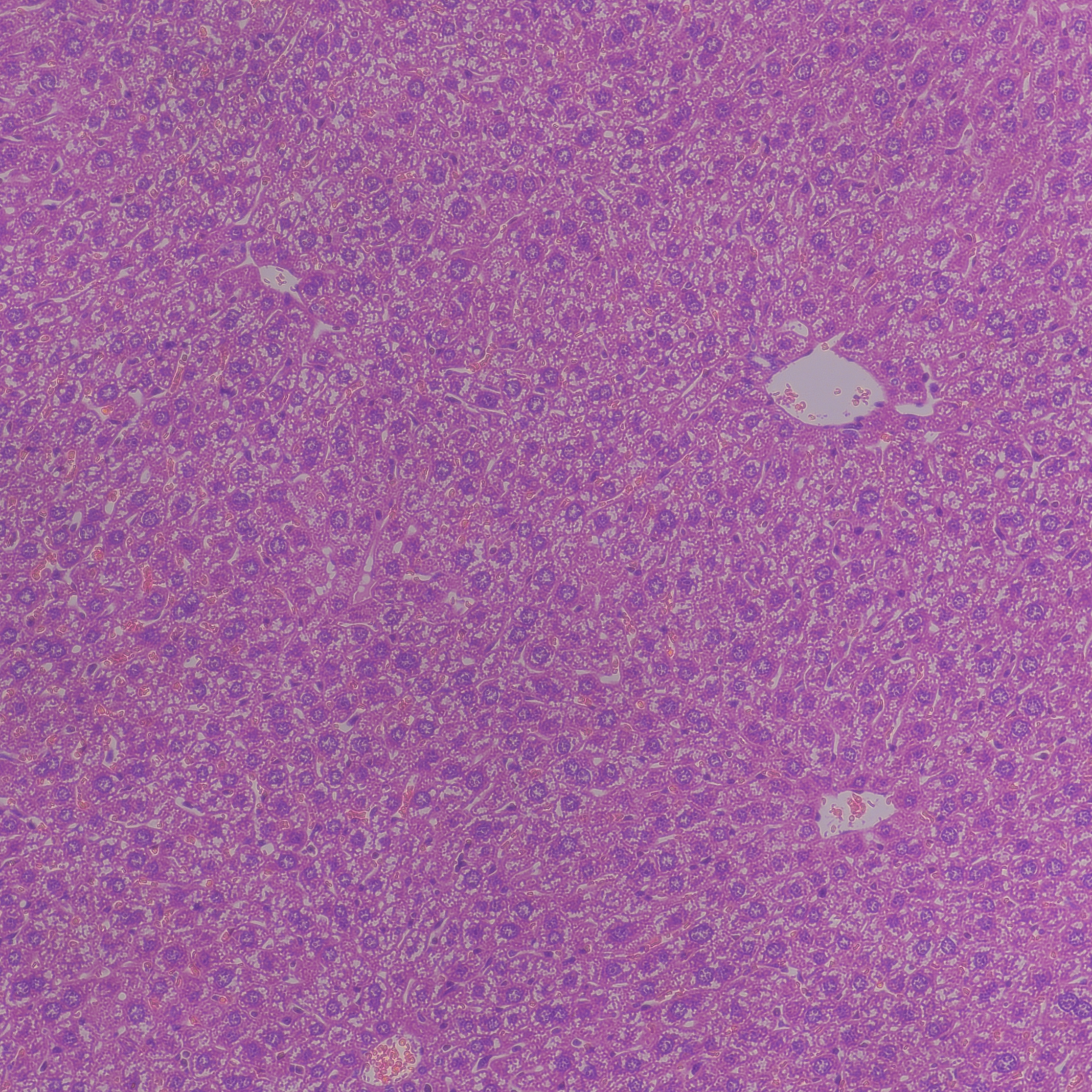

Supplement: Supplementary file 8 — EV Figure and Appendix Figure Source Data [file 44321_2025_200_MOESM8_ESM.zip › Fig. EV5/Fig. EV5H/Liver-Combo.tif]

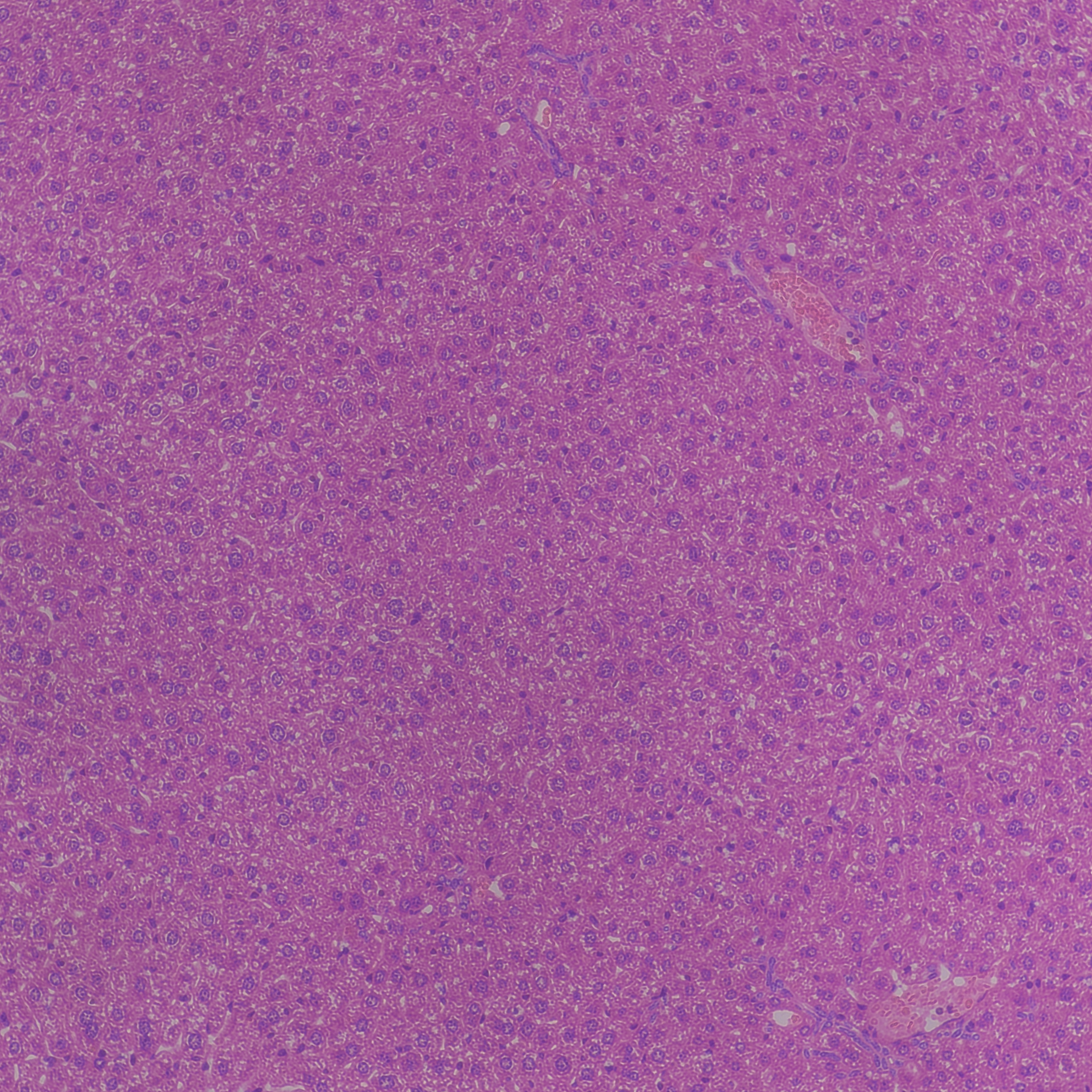

Supplement: Supplementary file 8 — EV Figure and Appendix Figure Source Data [file 44321_2025_200_MOESM8_ESM.zip › Fig. EV5/Fig. EV5H/Liver-Lac.tif]

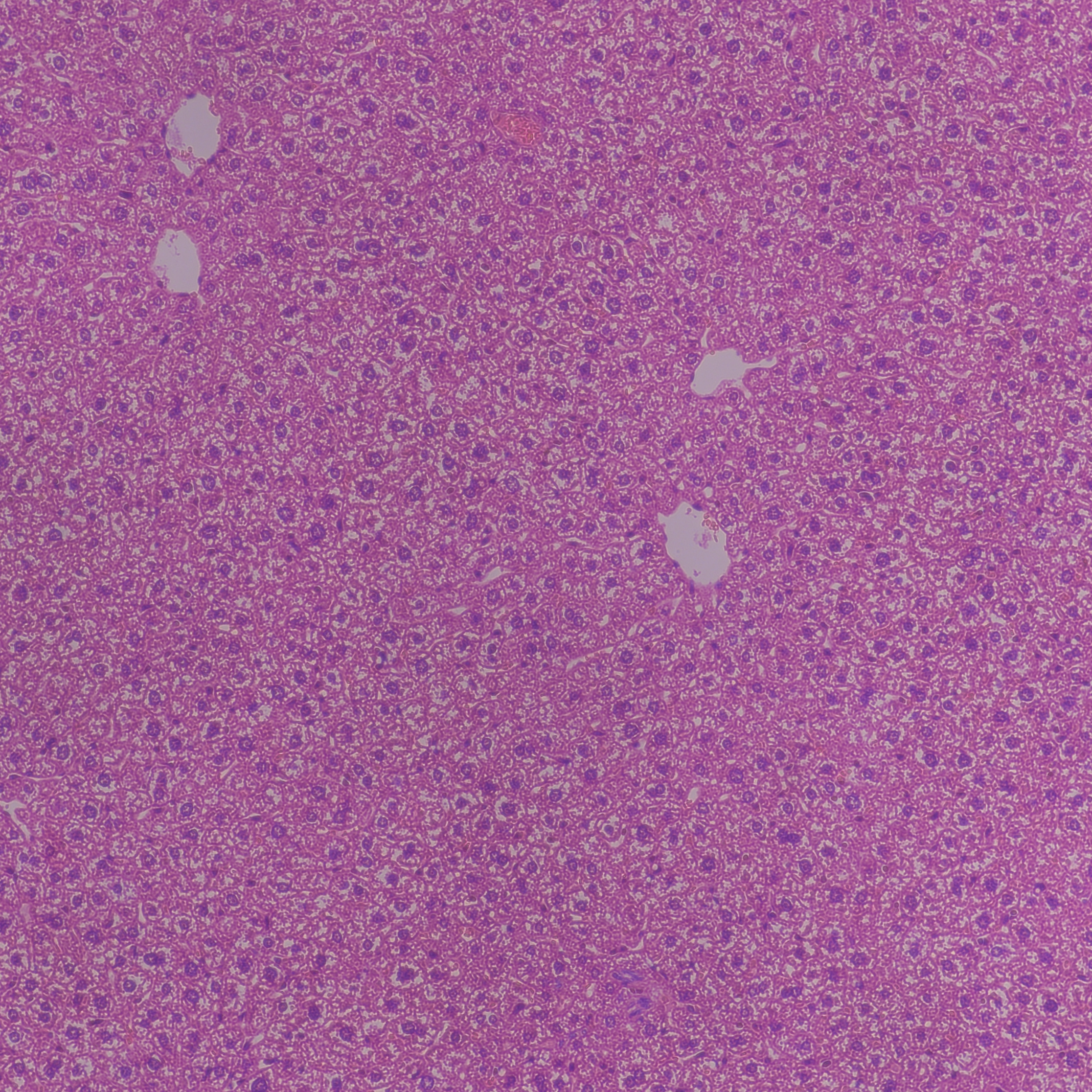

Supplement: Supplementary file 8 — EV Figure and Appendix Figure Source Data [file 44321_2025_200_MOESM8_ESM.zip › Fig. EV5/Fig. EV5H/Liver-PD1 Ab.tif]

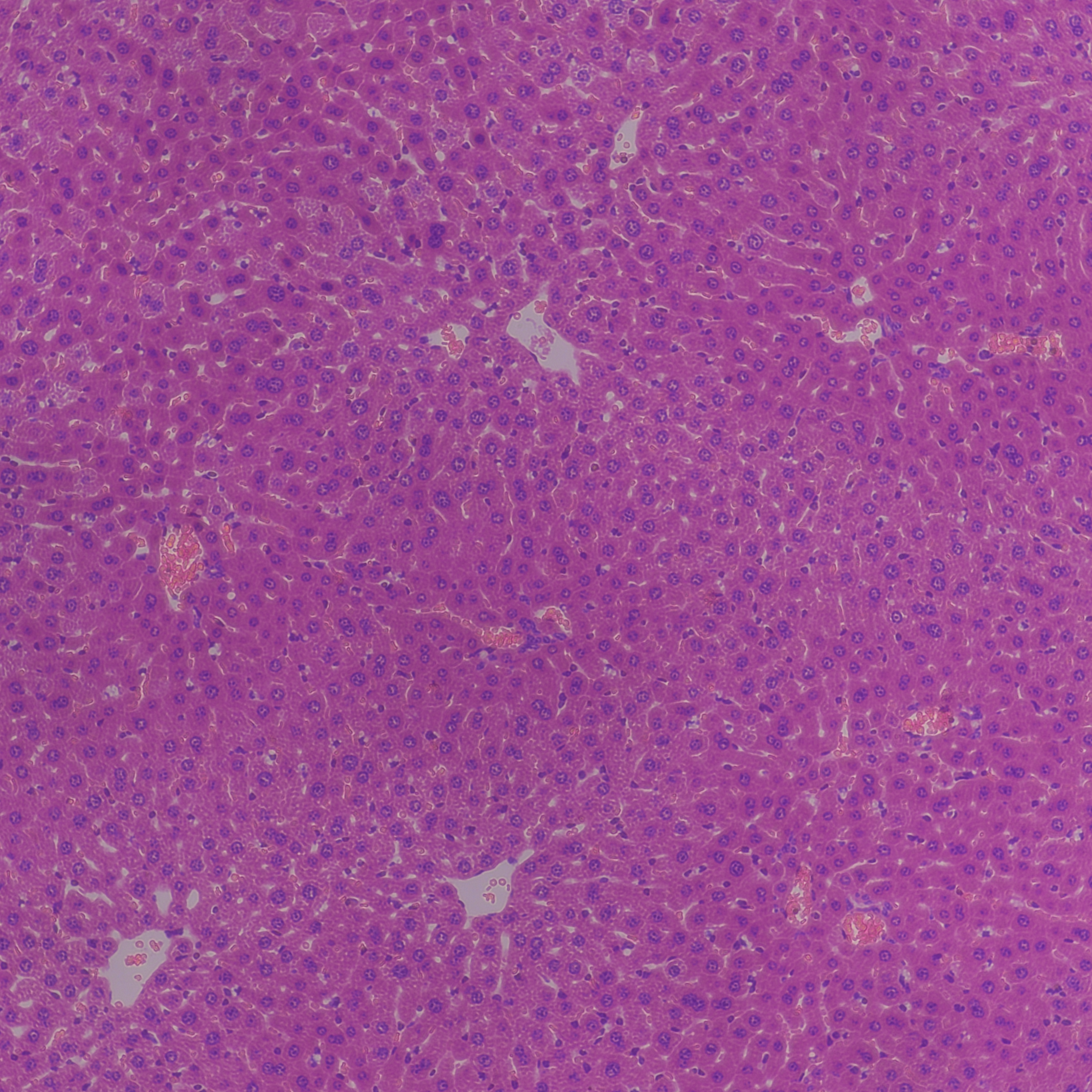

Supplement: Supplementary file 8 — EV Figure and Appendix Figure Source Data [file 44321_2025_200_MOESM8_ESM.zip › Fig. EV5/Fig. EV5H/Liver-Veh.tif]

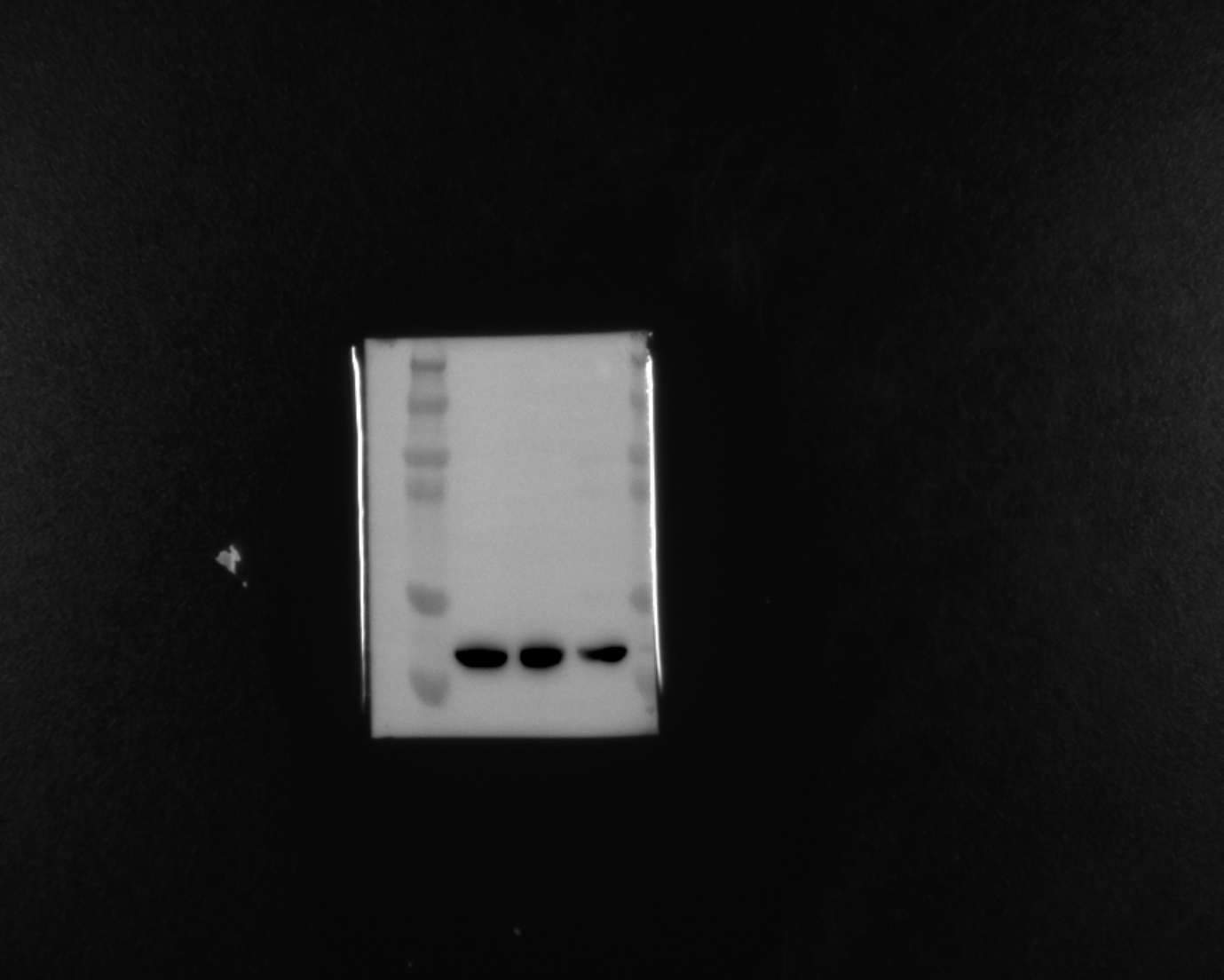

Supplement: Supplementary file 8 — EV Figure and Appendix Figure Source Data [file 44321_2025_200_MOESM8_ESM.zip › Appendix Fig. S1/Appendix Fig. S1A-right panel/Actin.tif]

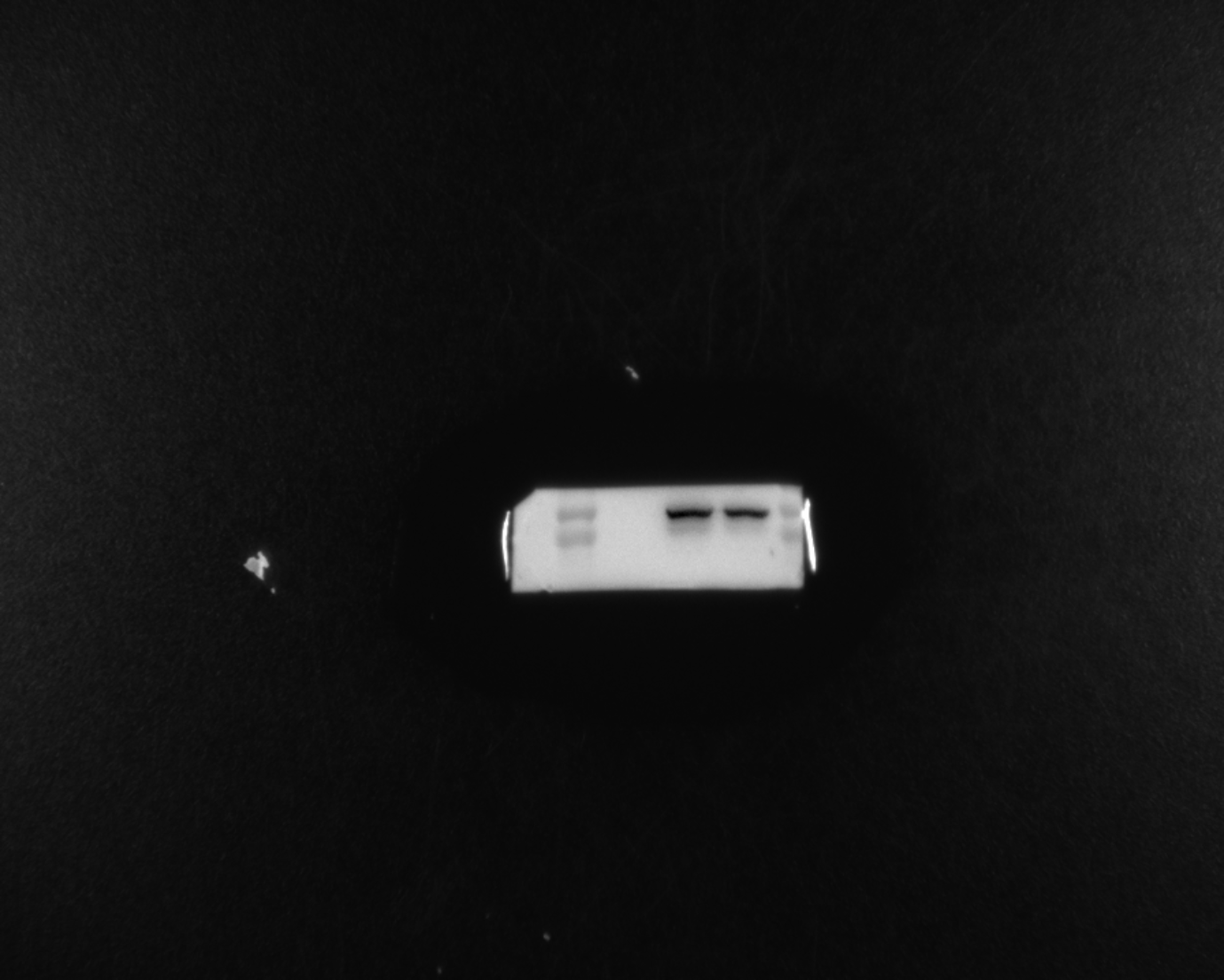

Supplement: Supplementary file 8 — EV Figure and Appendix Figure Source Data [file 44321_2025_200_MOESM8_ESM.zip › Appendix Fig. S1/Appendix Fig. S1A-right panel/Foxp3-Nluc-Flag.tif]

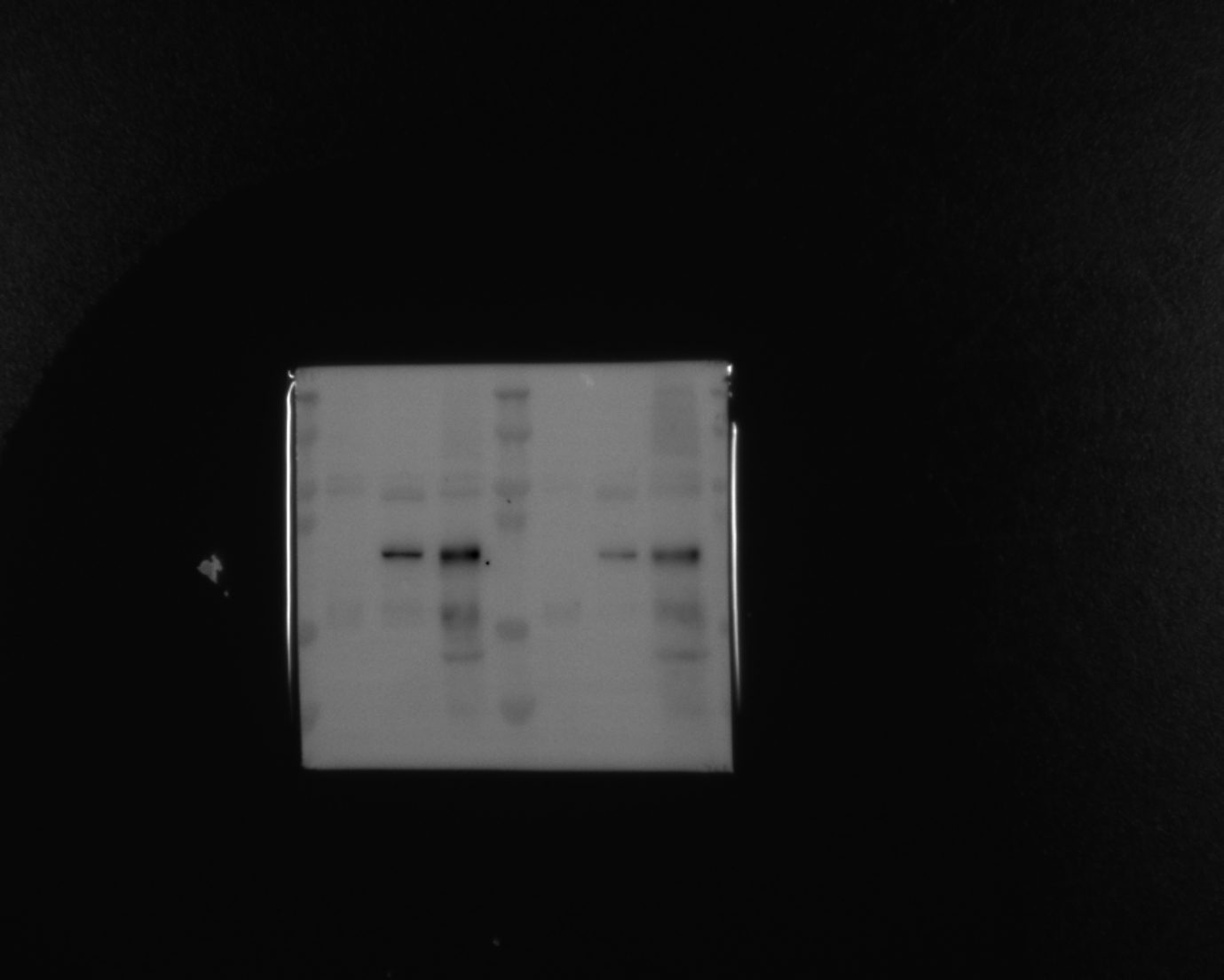

Supplement: Supplementary file 8 — EV Figure and Appendix Figure Source Data [file 44321_2025_200_MOESM8_ESM.zip › Appendix Fig. S1/Appendix Fig. S1A-right panel/RUNX1,CD27-Clu-Myc.tif]

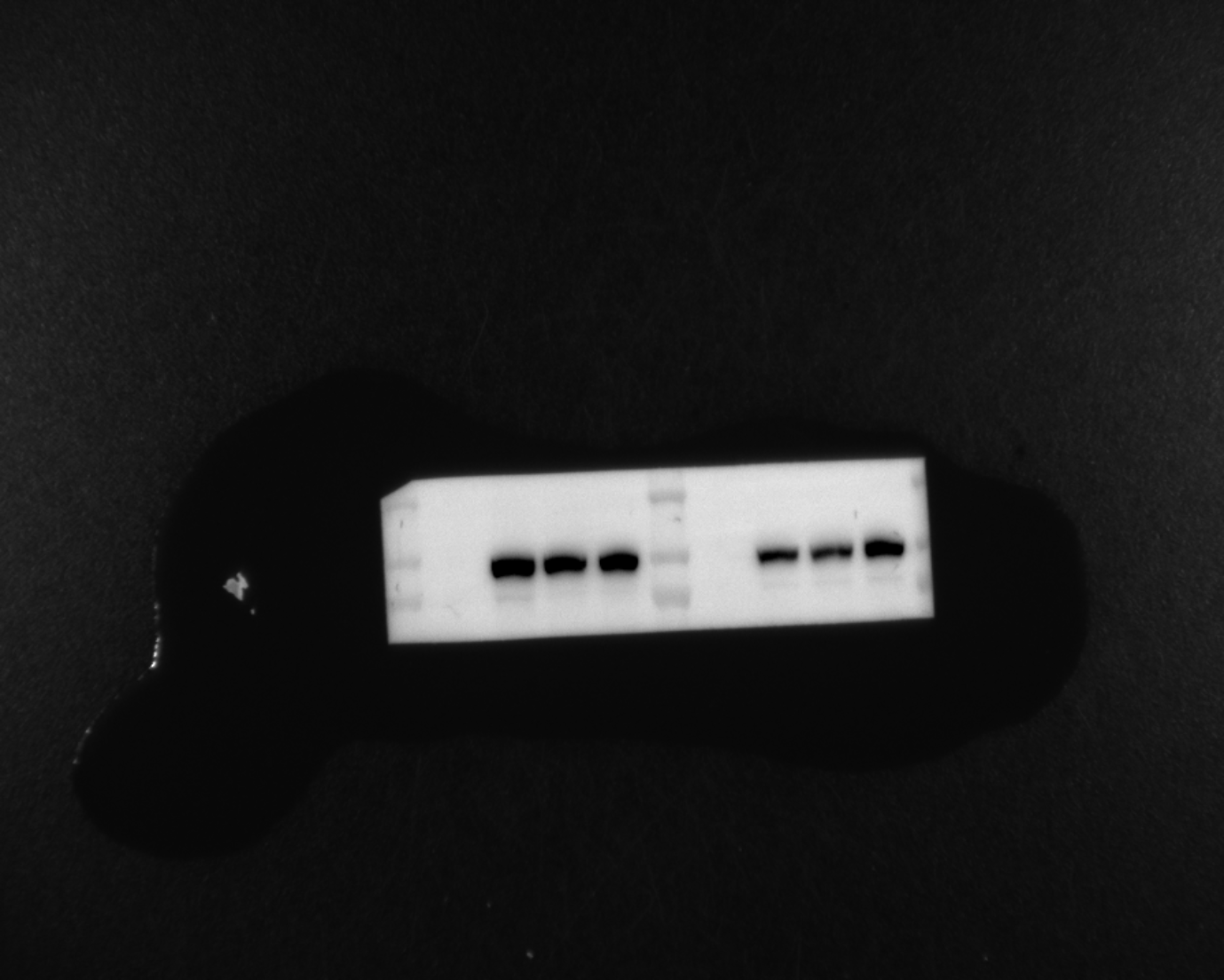

Supplement: Supplementary file 11 — Source data Fig. 3 [file 44321_2025_200_MOESM11_ESM.zip › EMM-2024-20400_SourceDataForFigure 3/Figure 3A/IP-FOXP3-Flag.tif]

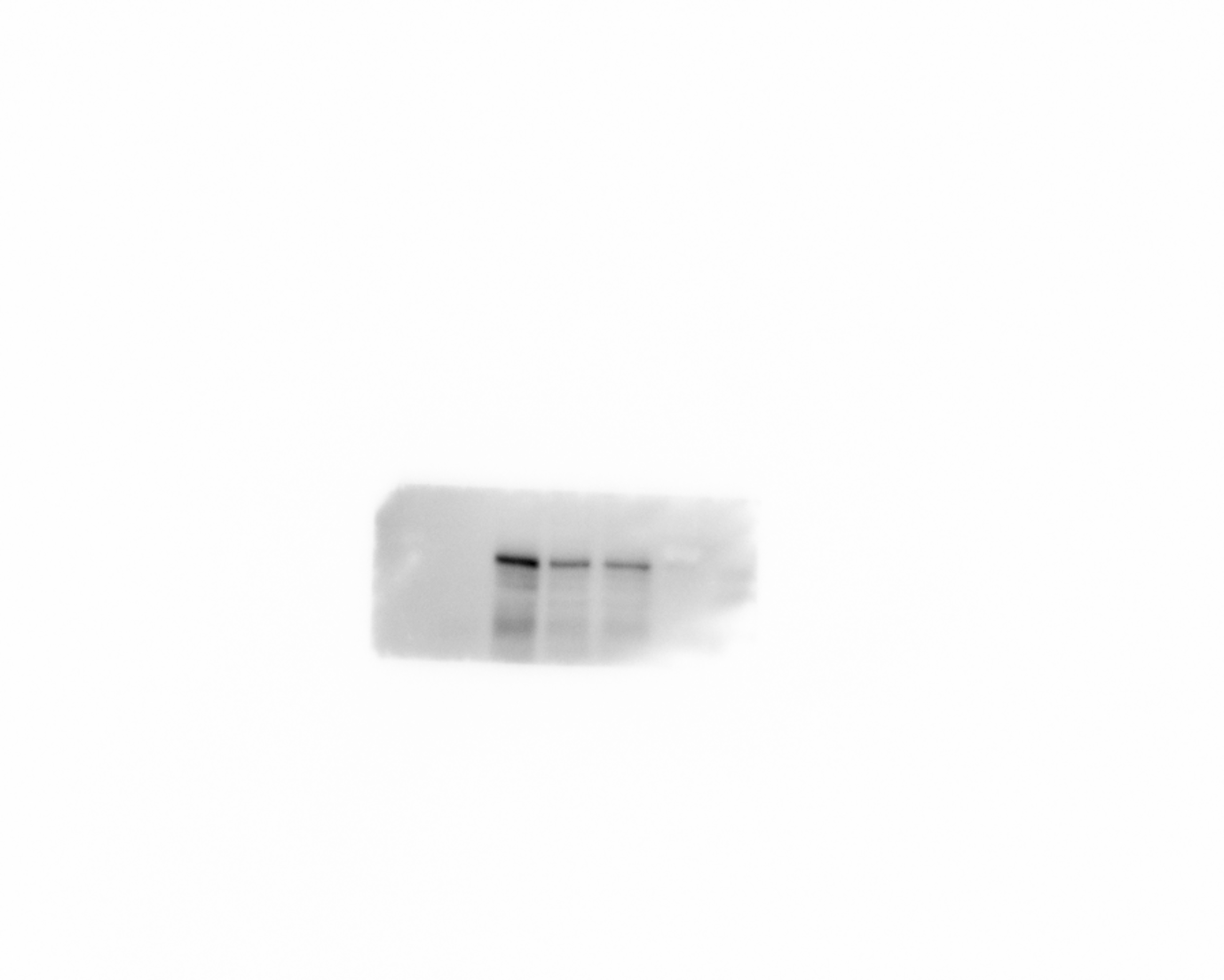

Supplement: Supplementary file 11 — Source data Fig. 3 [file 44321_2025_200_MOESM11_ESM.zip › EMM-2024-20400_SourceDataForFigure 3/Figure 3A/IP-RUNX1-Myc.tif]

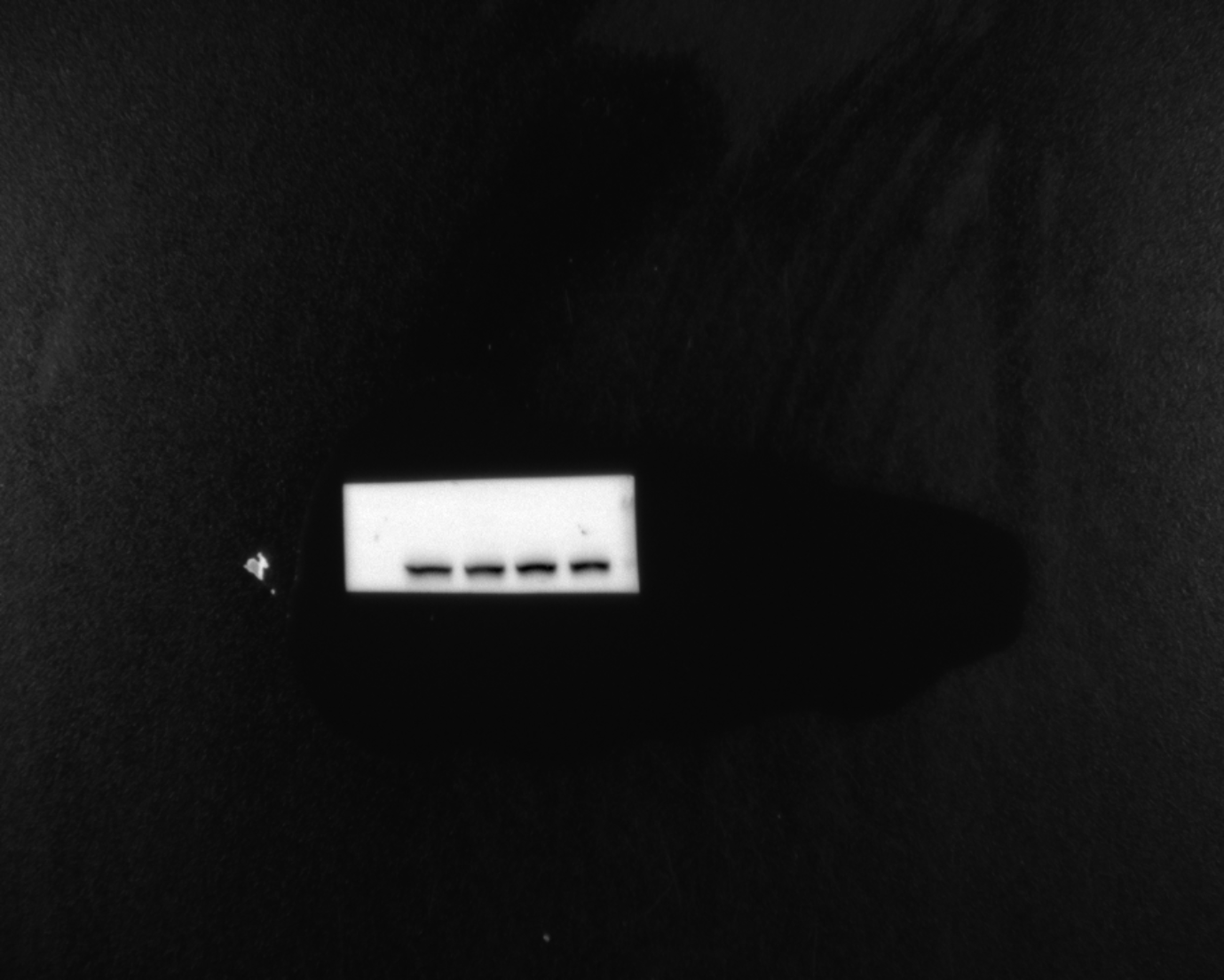

Supplement: Supplementary file 11 — Source data Fig. 3 [file 44321_2025_200_MOESM11_ESM.zip › EMM-2024-20400_SourceDataForFigure 3/Figure 3A/WCL-FOXP3-FLAG.tif]

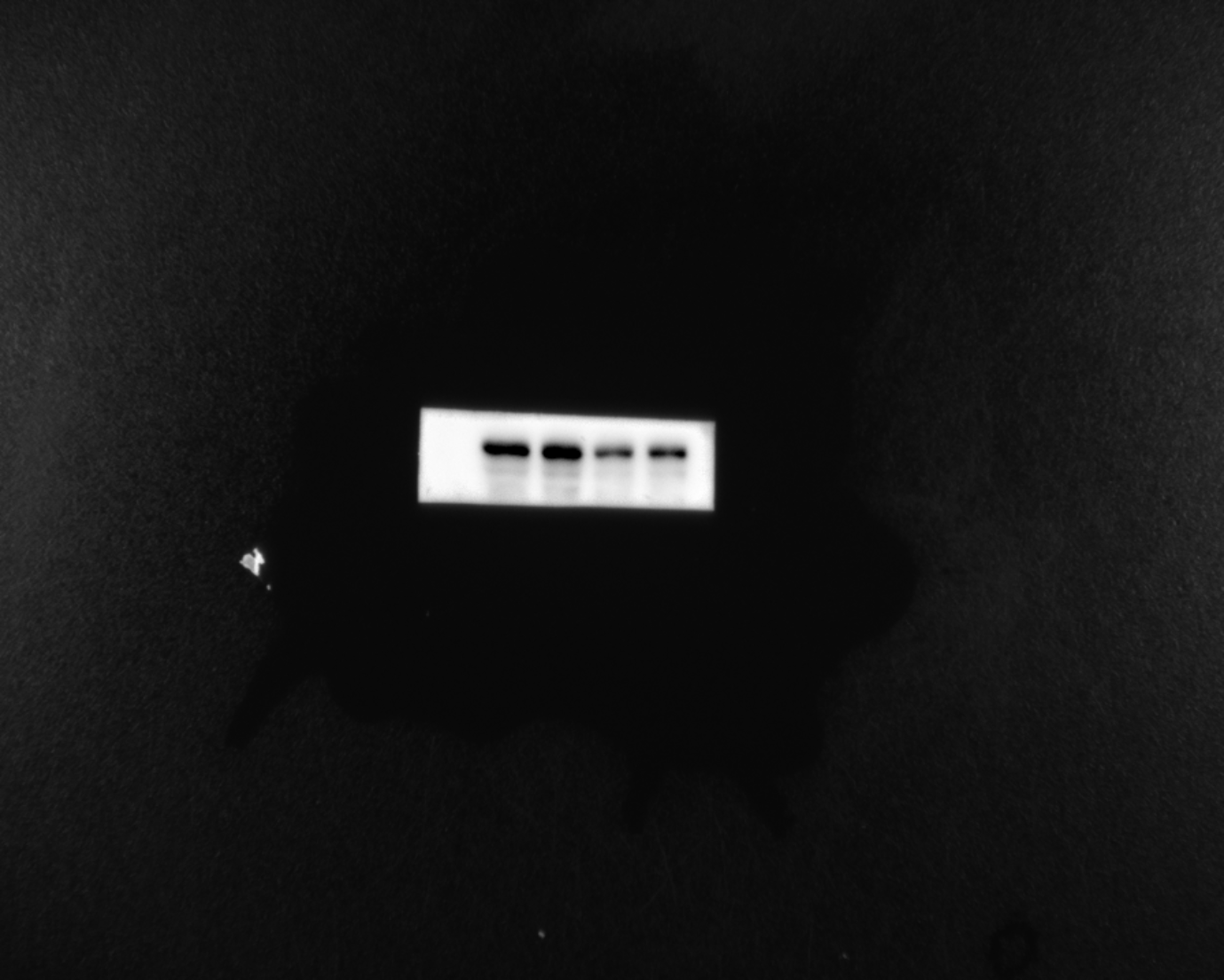

Supplement: Supplementary file 11 — Source data Fig. 3 [file 44321_2025_200_MOESM11_ESM.zip › EMM-2024-20400_SourceDataForFigure 3/Figure 3A/WCL-RUNX1-Myc.tif]

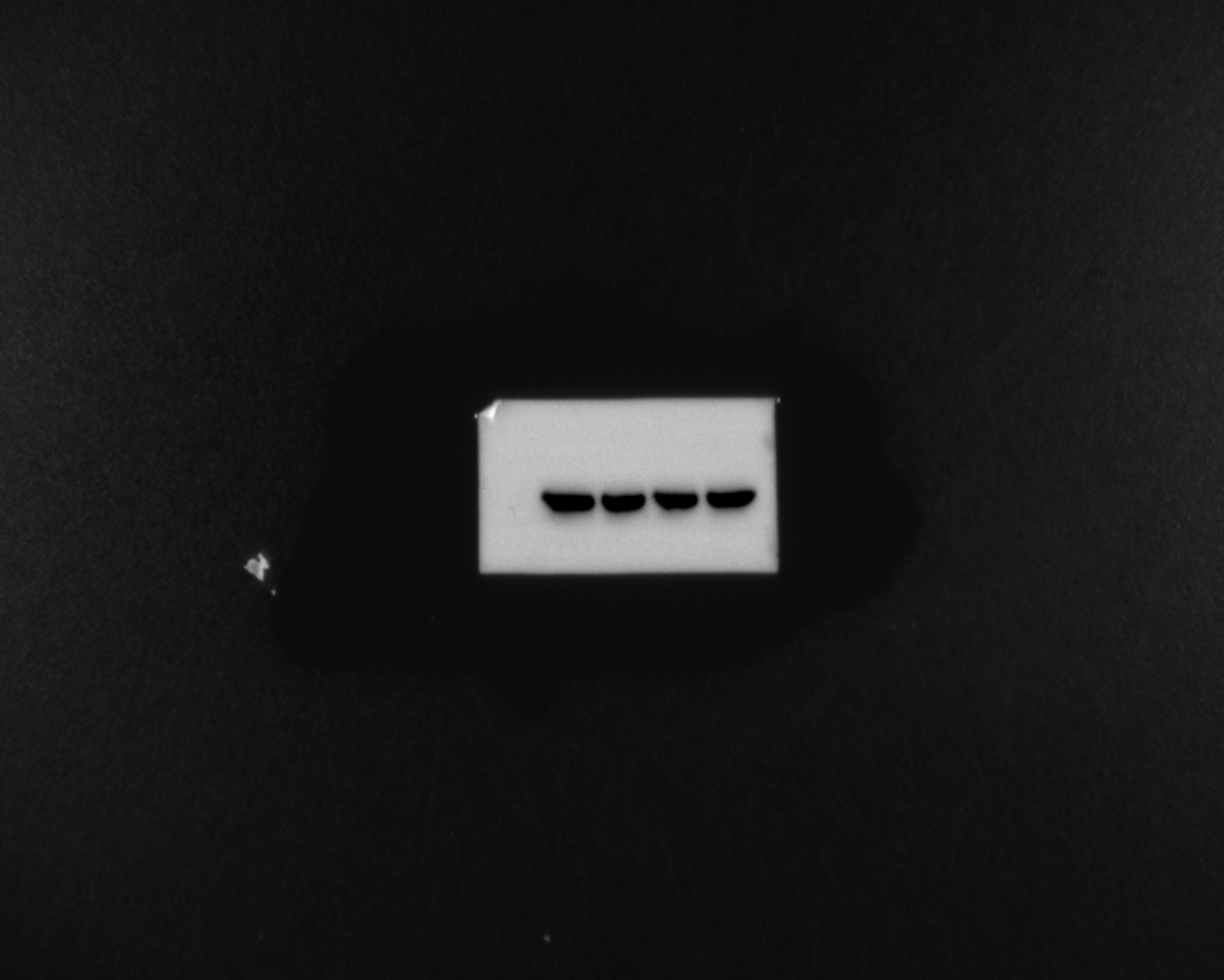

Supplement: Supplementary file 11 — Source data Fig. 3 [file 44321_2025_200_MOESM11_ESM.zip › EMM-2024-20400_SourceDataForFigure 3/Figure 3A/β-Actin.tif]

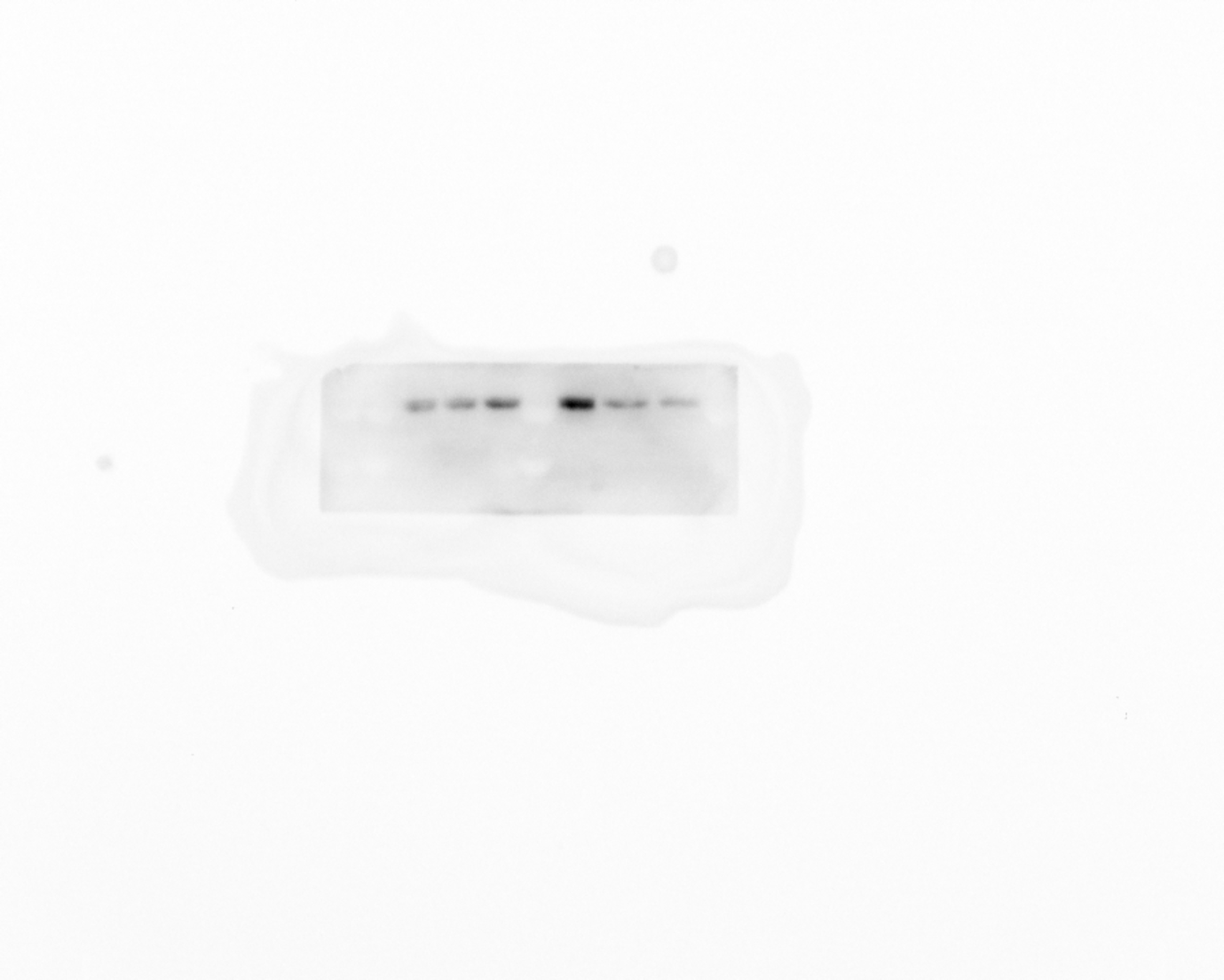

Supplement: Supplementary file 11 — Source data Fig. 3 [file 44321_2025_200_MOESM11_ESM.zip › EMM-2024-20400_SourceDataForFigure 3/Figure 3B/RUNX1-Myc.tif]

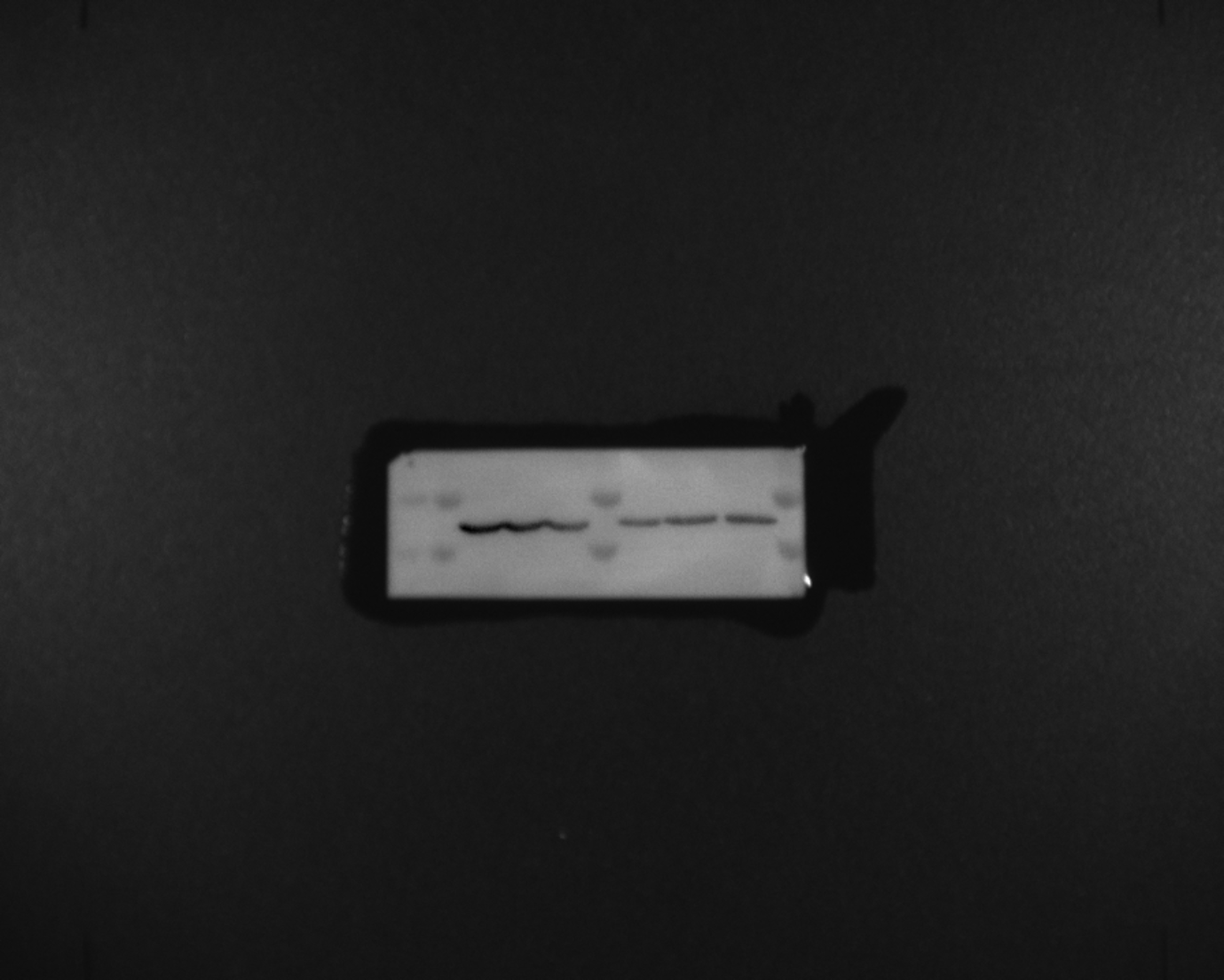

Supplement: Supplementary file 11 — Source data Fig. 3 [file 44321_2025_200_MOESM11_ESM.zip › EMM-2024-20400_SourceDataForFigure 3/Figure 3B/β-Actin.tif]

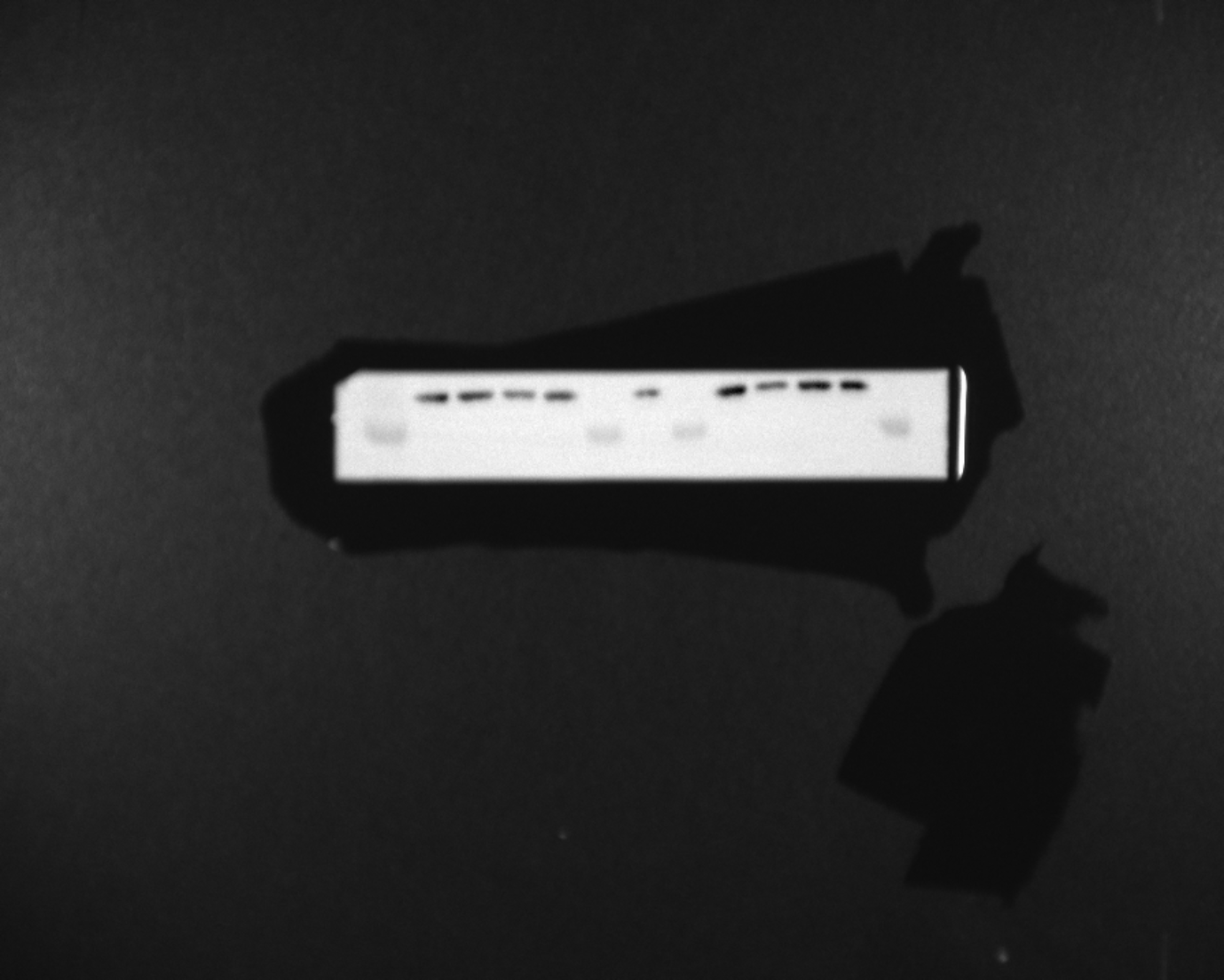

Supplement: Supplementary file 11 — Source data Fig. 3 [file 44321_2025_200_MOESM11_ESM.zip › EMM-2024-20400_SourceDataForFigure 3/Figure 3C/JURKAT-actin-2.tif]

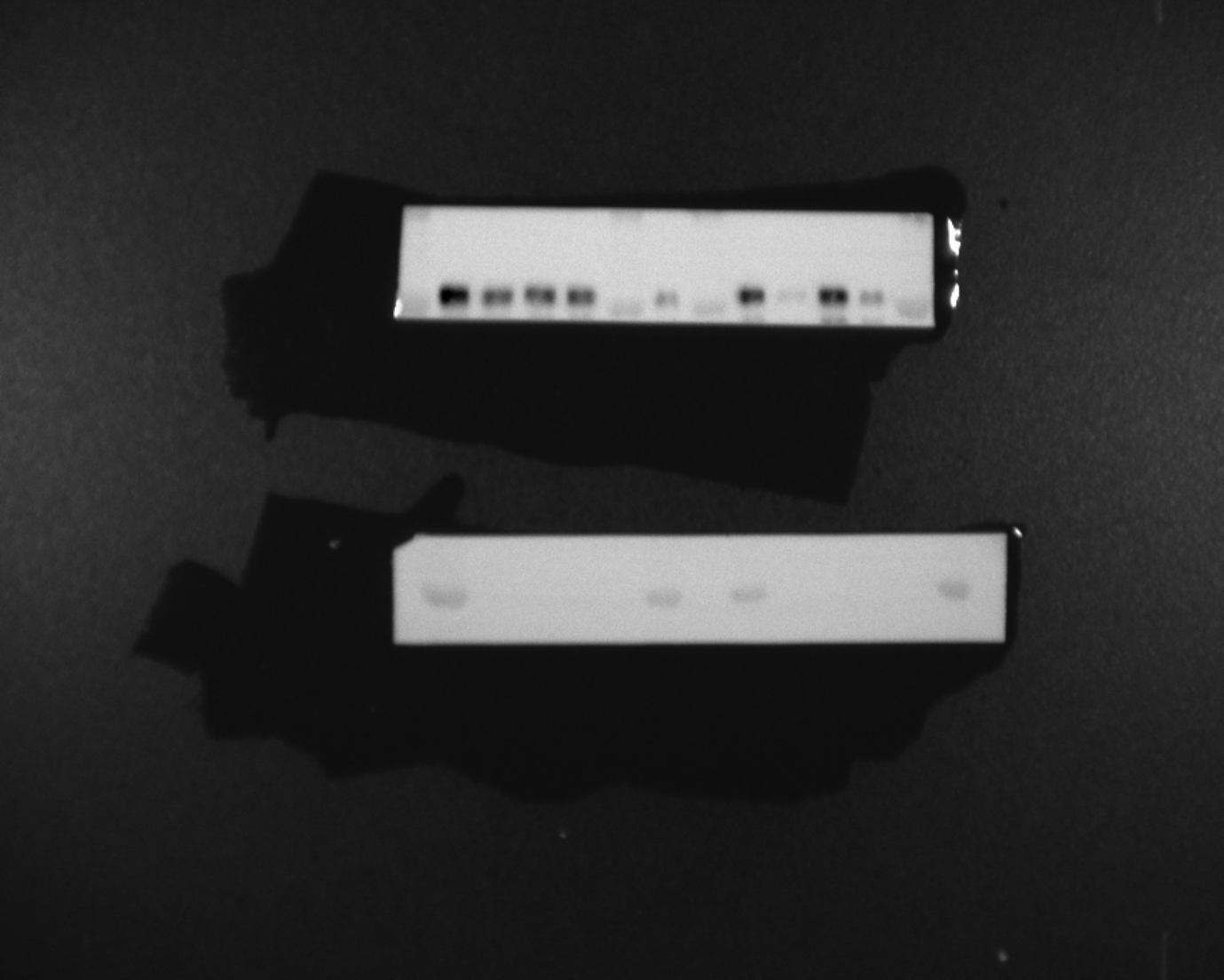

Supplement: Supplementary file 11 — Source data Fig. 3 [file 44321_2025_200_MOESM11_ESM.zip › EMM-2024-20400_SourceDataForFigure 3/Figure 3C/JURKAT-runx1-3.tif]

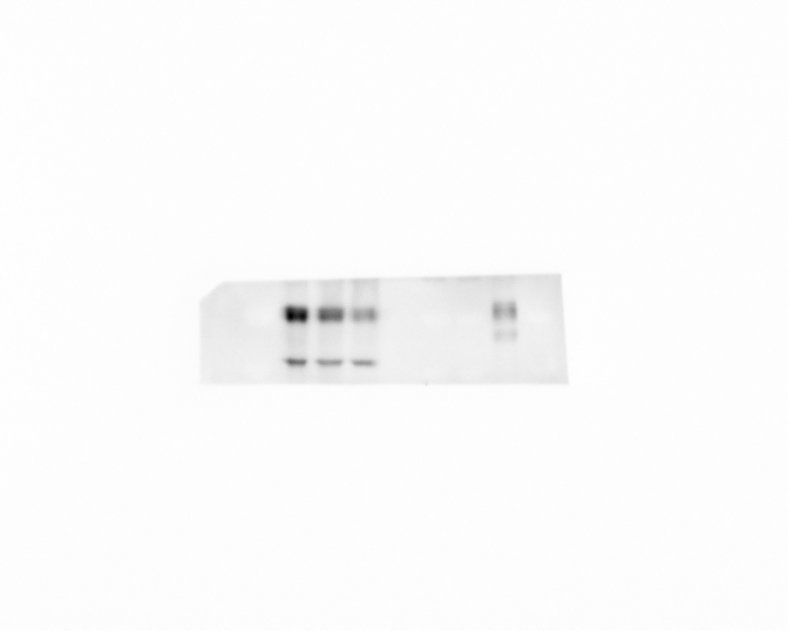

Supplement: Supplementary file 11 — Source data Fig. 3 [file 44321_2025_200_MOESM11_ESM.zip › EMM-2024-20400_SourceDataForFigure 3/Figure 3F/RUNX1.tif]

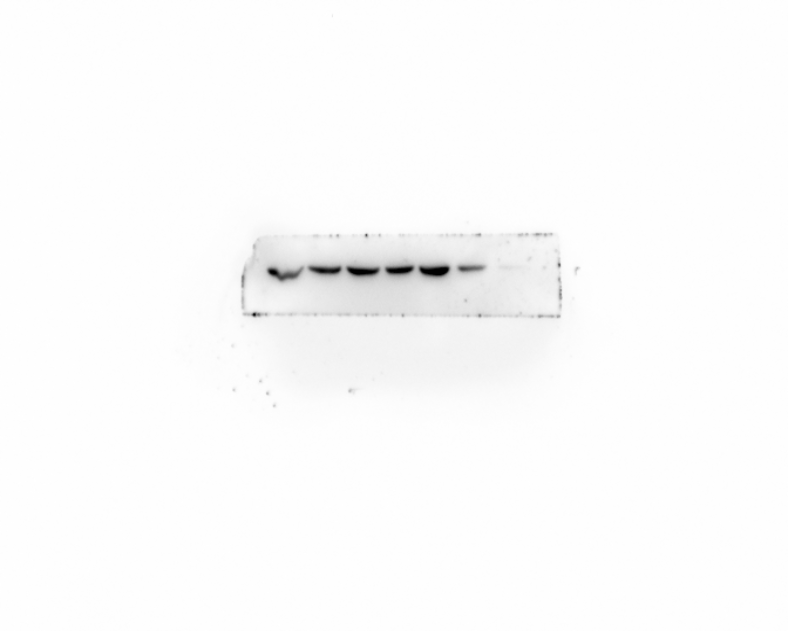

Supplement: Supplementary file 11 — Source data Fig. 3 [file 44321_2025_200_MOESM11_ESM.zip › EMM-2024-20400_SourceDataForFigure 3/Figure 3F/β-Actin.tif]

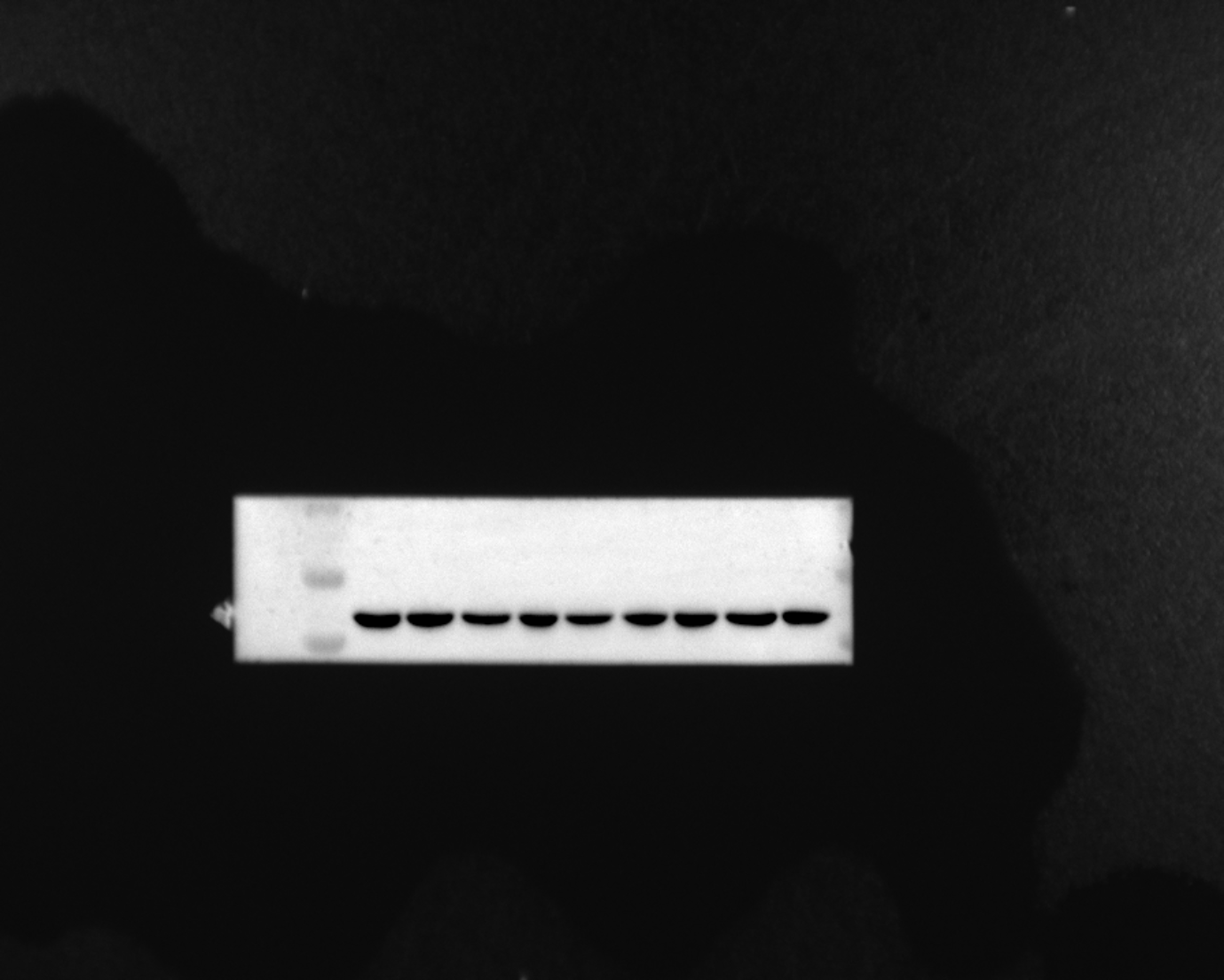

Supplement: Supplementary file 11 — Source data Fig. 3 [file 44321_2025_200_MOESM11_ESM.zip › EMM-2024-20400_SourceDataForFigure 3/Figure 3G/ACTIN.tif]

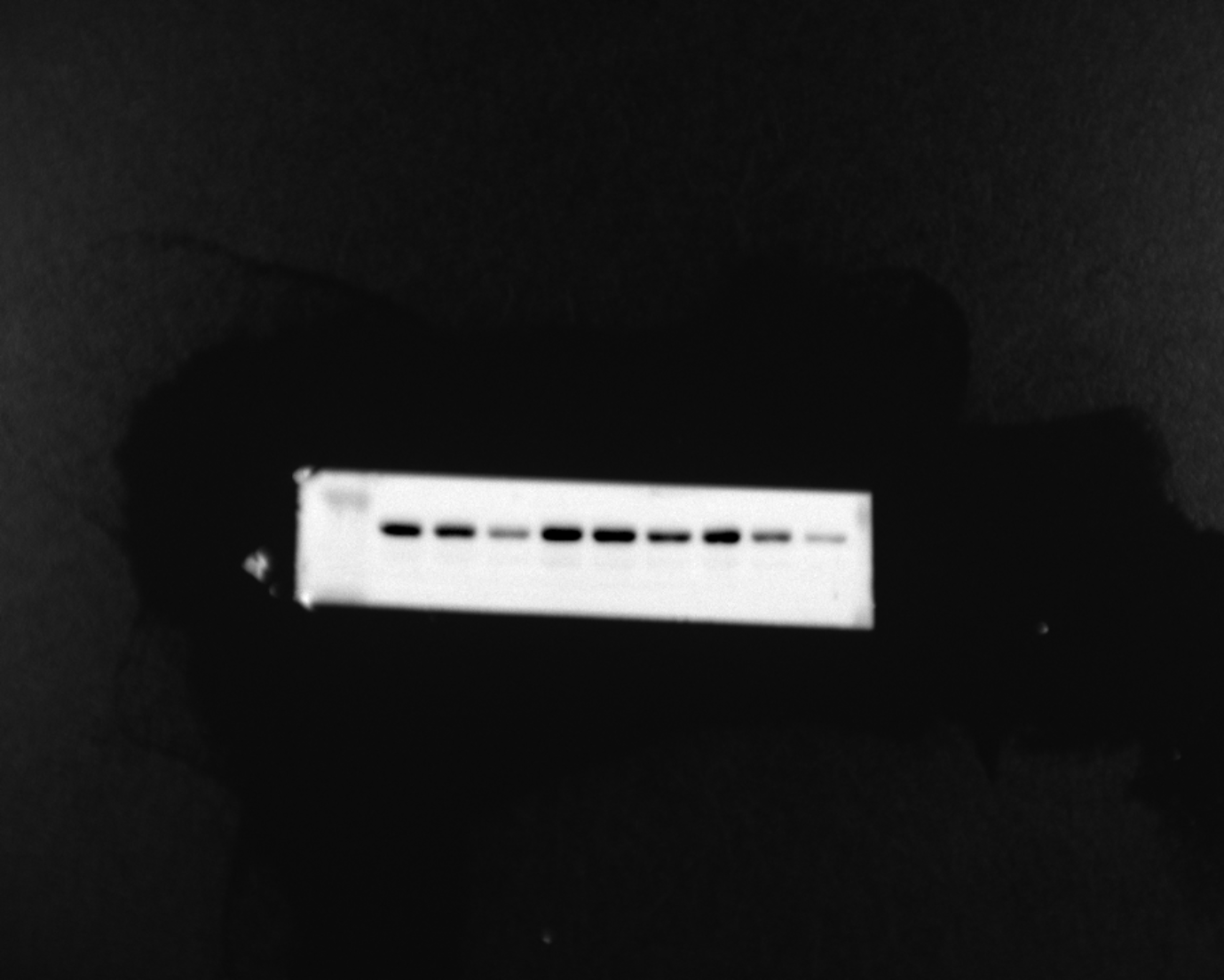

Supplement: Supplementary file 11 — Source data Fig. 3 [file 44321_2025_200_MOESM11_ESM.zip › EMM-2024-20400_SourceDataForFigure 3/Figure 3G/RUNX1-Clu.tif]

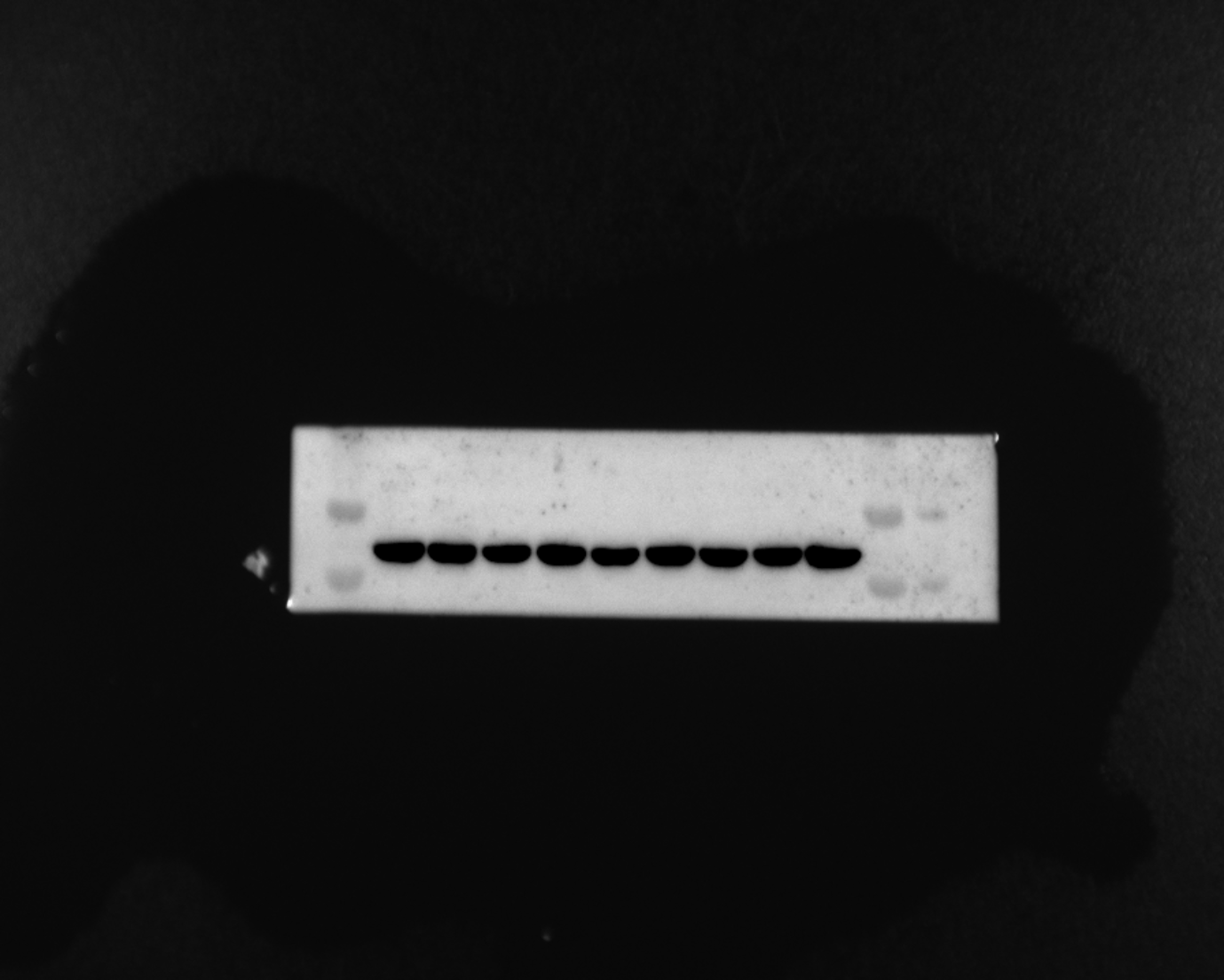

Supplement: Supplementary file 11 — Source data Fig. 3 [file 44321_2025_200_MOESM11_ESM.zip › EMM-2024-20400_SourceDataForFigure 3/Figure 3H/Actin.tif]

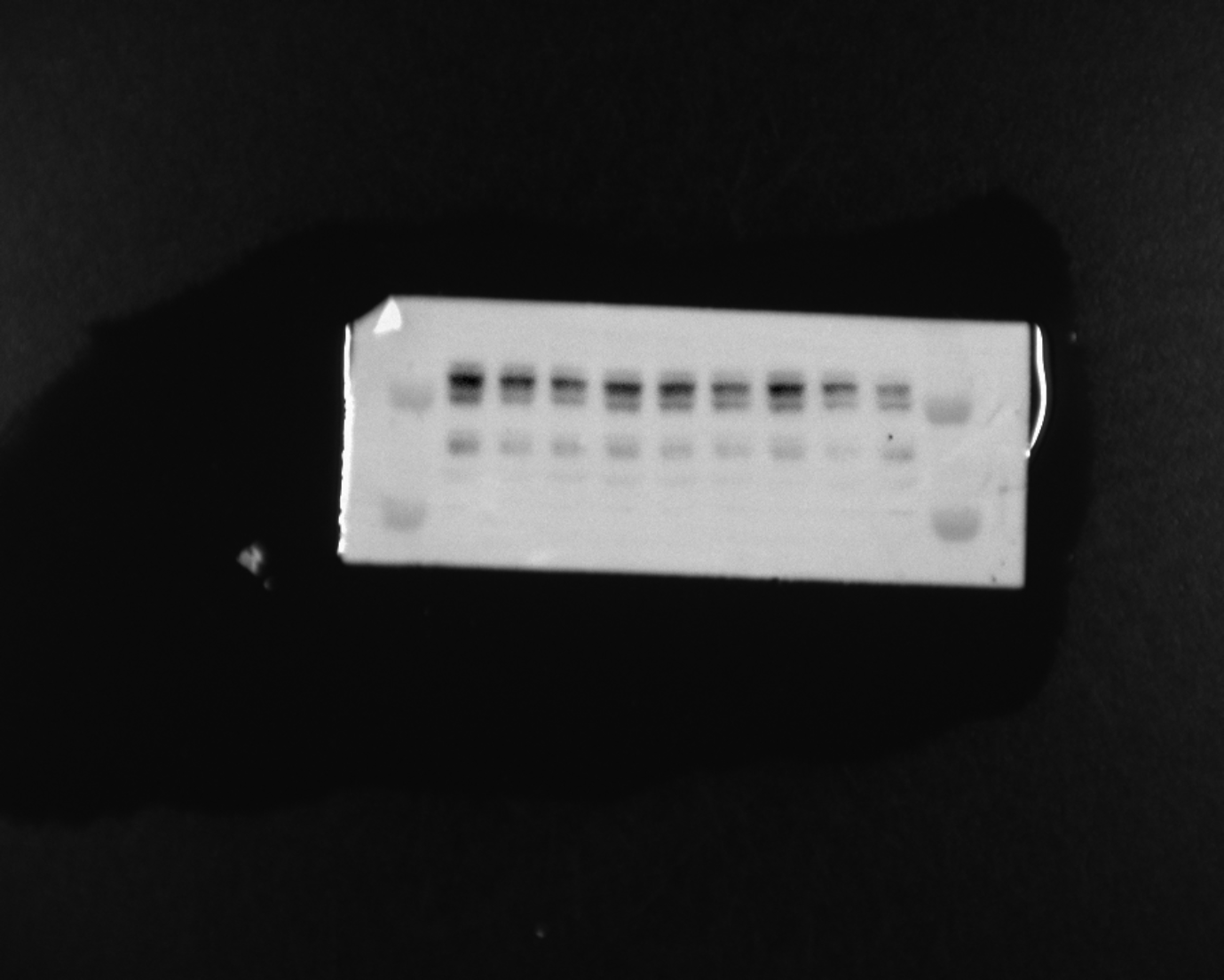

Supplement: Supplementary file 11 — Source data Fig. 3 [file 44321_2025_200_MOESM11_ESM.zip › EMM-2024-20400_SourceDataForFigure 3/Figure 3H/RUNX1.tif]

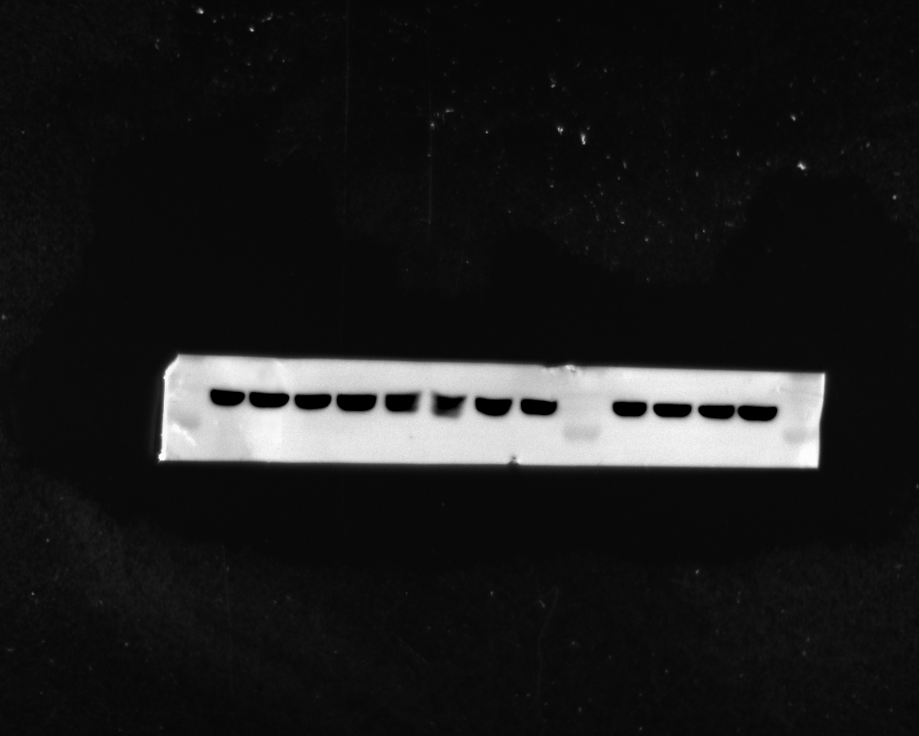

Supplement: Supplementary file 12 — Source data Fig. 4 [file 44321_2025_200_MOESM12_ESM.zip › EMM-2024-20400_SourceDataForFigure 4/Figure 4A/ACTIN.tif]

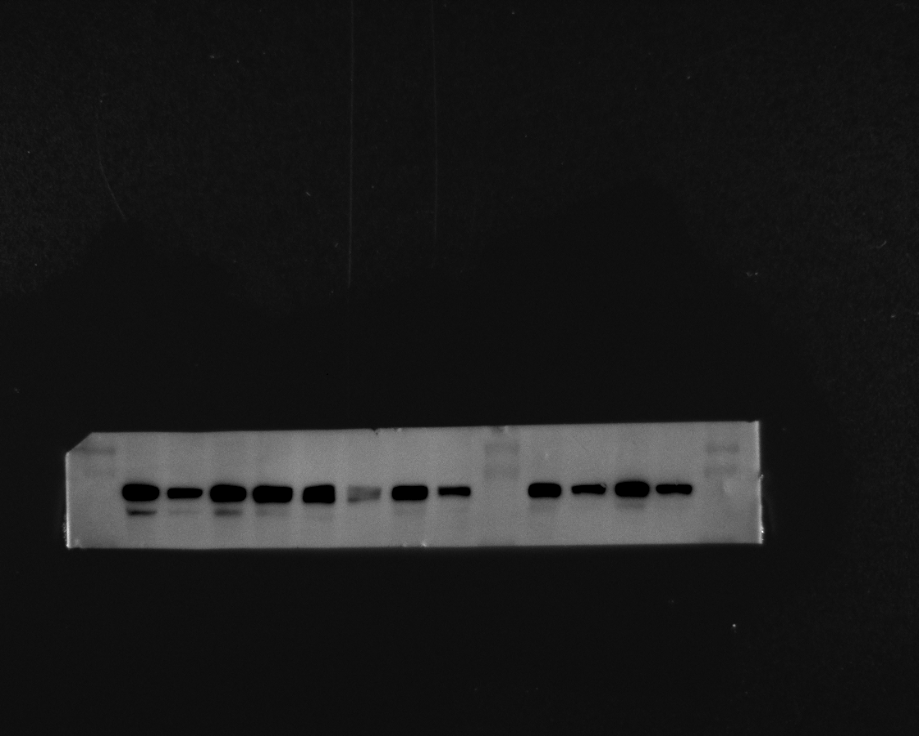

Supplement: Supplementary file 12 — Source data Fig. 4 [file 44321_2025_200_MOESM12_ESM.zip › EMM-2024-20400_SourceDataForFigure 4/Figure 4A/RUNX1-Clu.tif]

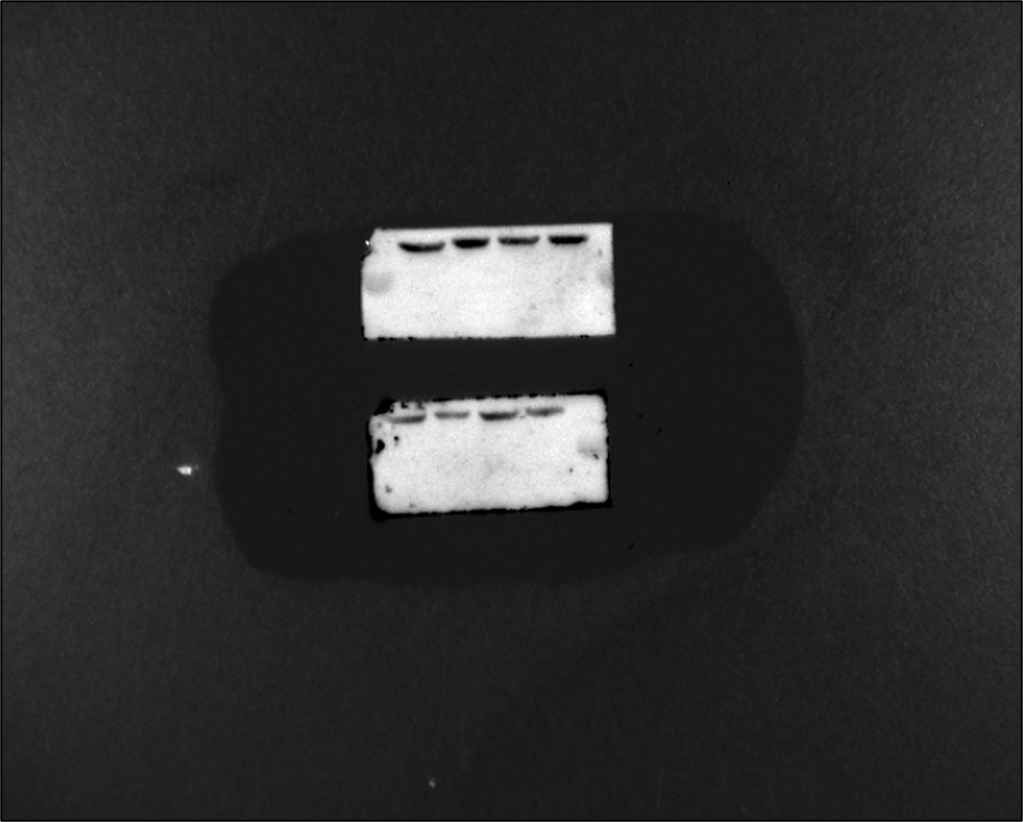

Supplement: Supplementary file 12 — Source data Fig. 4 [file 44321_2025_200_MOESM12_ESM.zip › EMM-2024-20400_SourceDataForFigure 4/Figure 4B/ACTIN.tif]

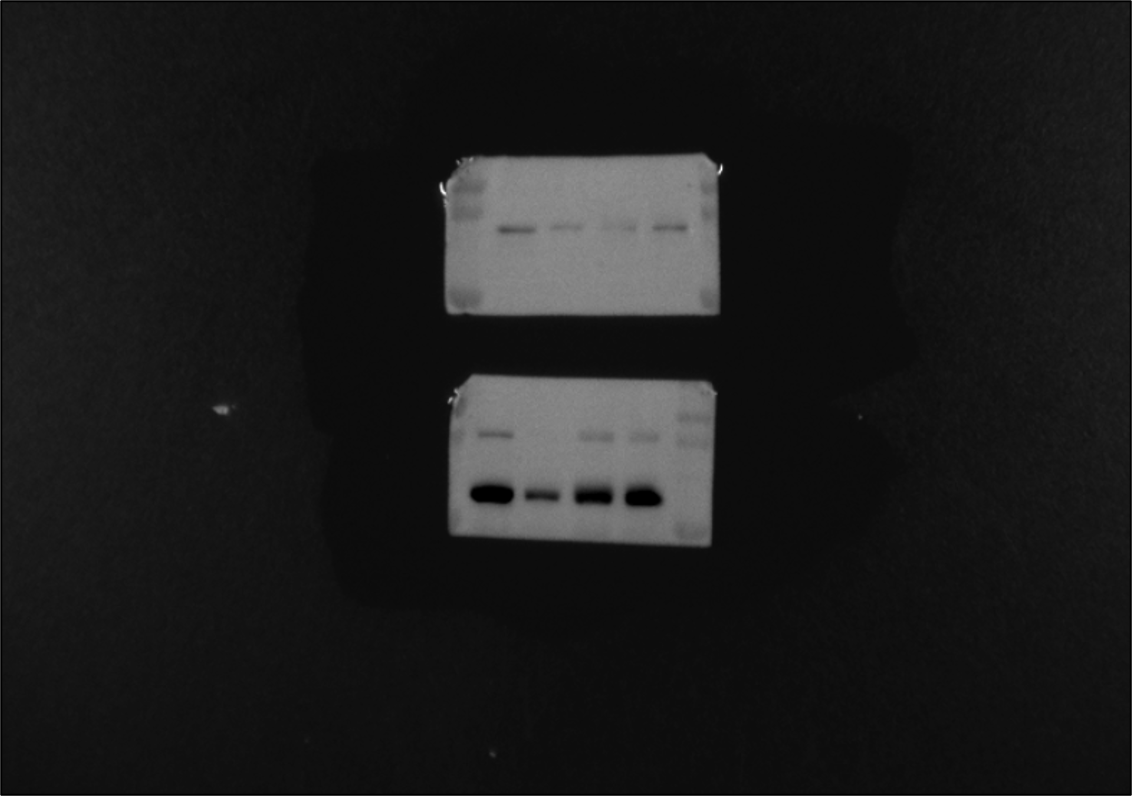

Supplement: Supplementary file 12 — Source data Fig. 4 [file 44321_2025_200_MOESM12_ESM.zip › EMM-2024-20400_SourceDataForFigure 4/Figure 4B/RUNX1.tif]

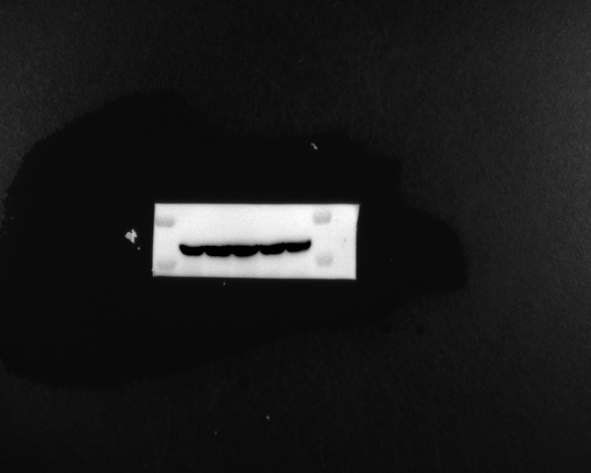

Supplement: Supplementary file 12 — Source data Fig. 4 [file 44321_2025_200_MOESM12_ESM.zip › EMM-2024-20400_SourceDataForFigure 4/Figure 4D/actin-1.tif]

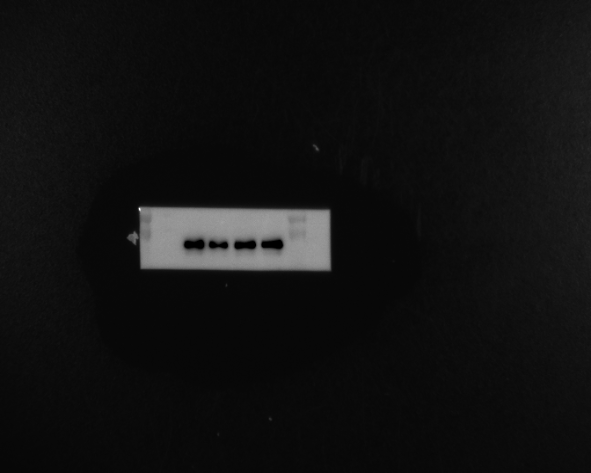

Supplement: Supplementary file 12 — Source data Fig. 4 [file 44321_2025_200_MOESM12_ESM.zip › EMM-2024-20400_SourceDataForFigure 4/Figure 4D/ip-RUNX1-1.tif]

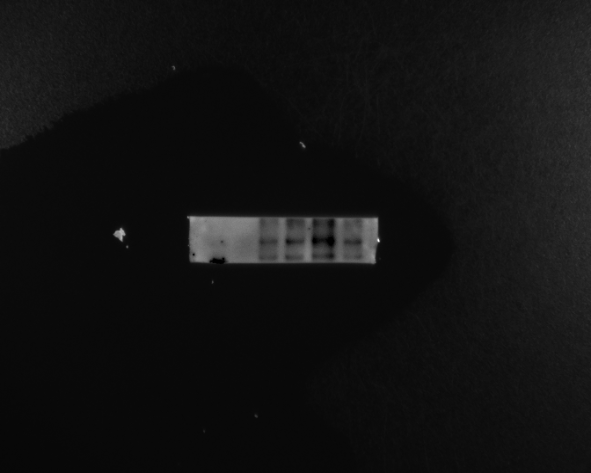

Supplement: Supplementary file 12 — Source data Fig. 4 [file 44321_2025_200_MOESM12_ESM.zip › EMM-2024-20400_SourceDataForFigure 4/Figure 4D/IP-STUB1(Intense exposure).tif]

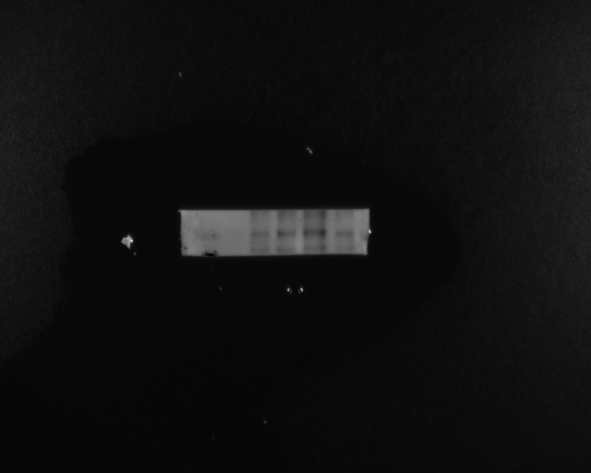

Supplement: Supplementary file 12 — Source data Fig. 4 [file 44321_2025_200_MOESM12_ESM.zip › EMM-2024-20400_SourceDataForFigure 4/Figure 4D/IP-STUB1(Weak exposure).tif]

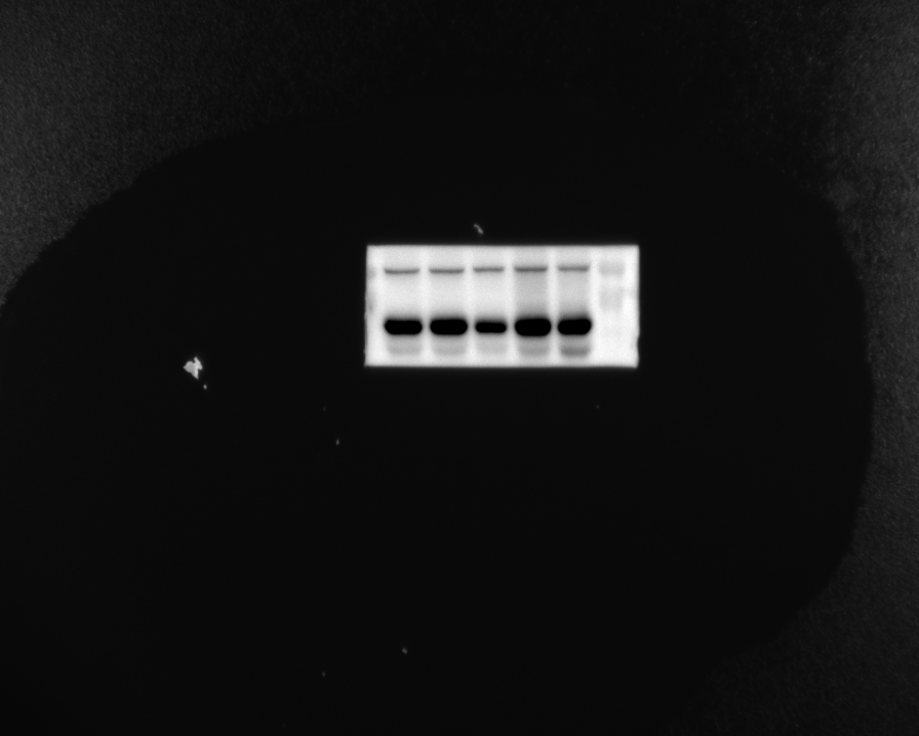

Supplement: Supplementary file 12 — Source data Fig. 4 [file 44321_2025_200_MOESM12_ESM.zip › EMM-2024-20400_SourceDataForFigure 4/Figure 4D/WCL-RUNX1-4.tif]

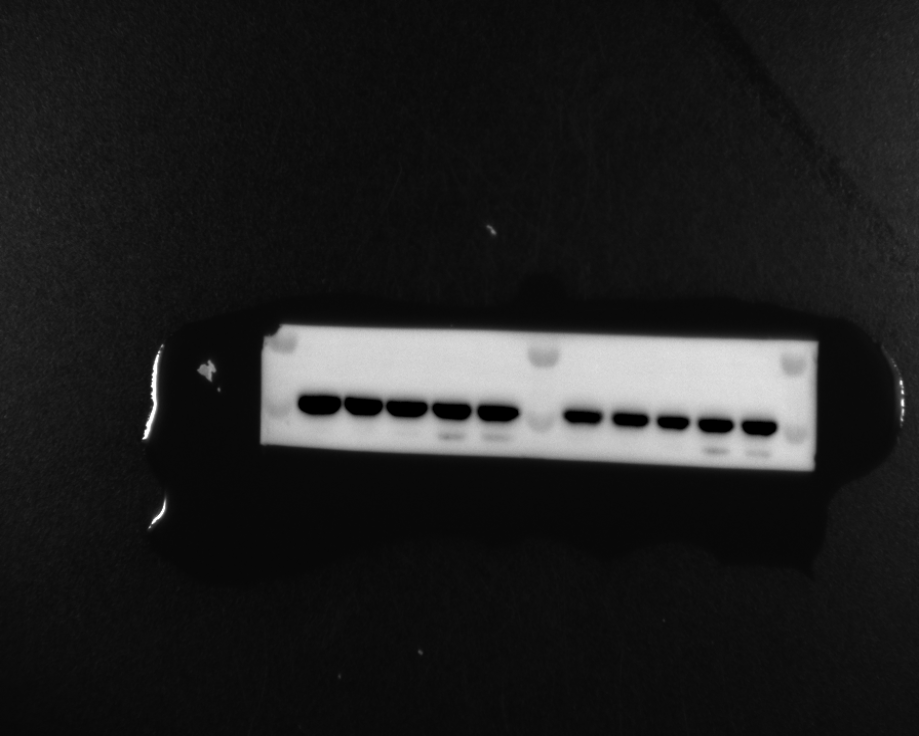

Supplement: Supplementary file 12 — Source data Fig. 4 [file 44321_2025_200_MOESM12_ESM.zip › EMM-2024-20400_SourceDataForFigure 4/Figure 4D/WCL-STUB1-1.tif]

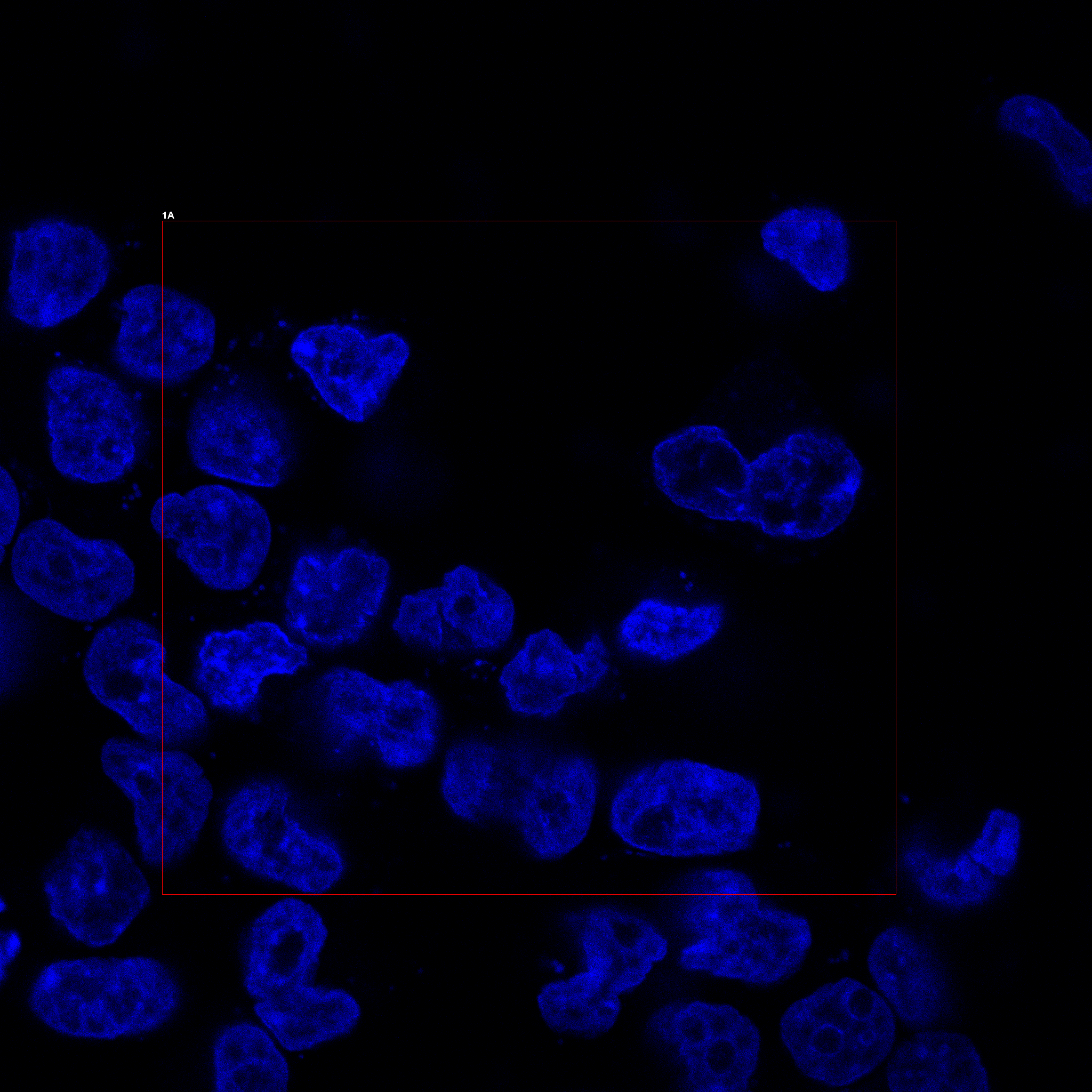

Supplement: Supplementary file 12 — Source data Fig. 4 [file 44321_2025_200_MOESM12_ESM.zip › EMM-2024-20400_SourceDataForFigure 4/Figure 4E/DMSO/DAPI.tif]

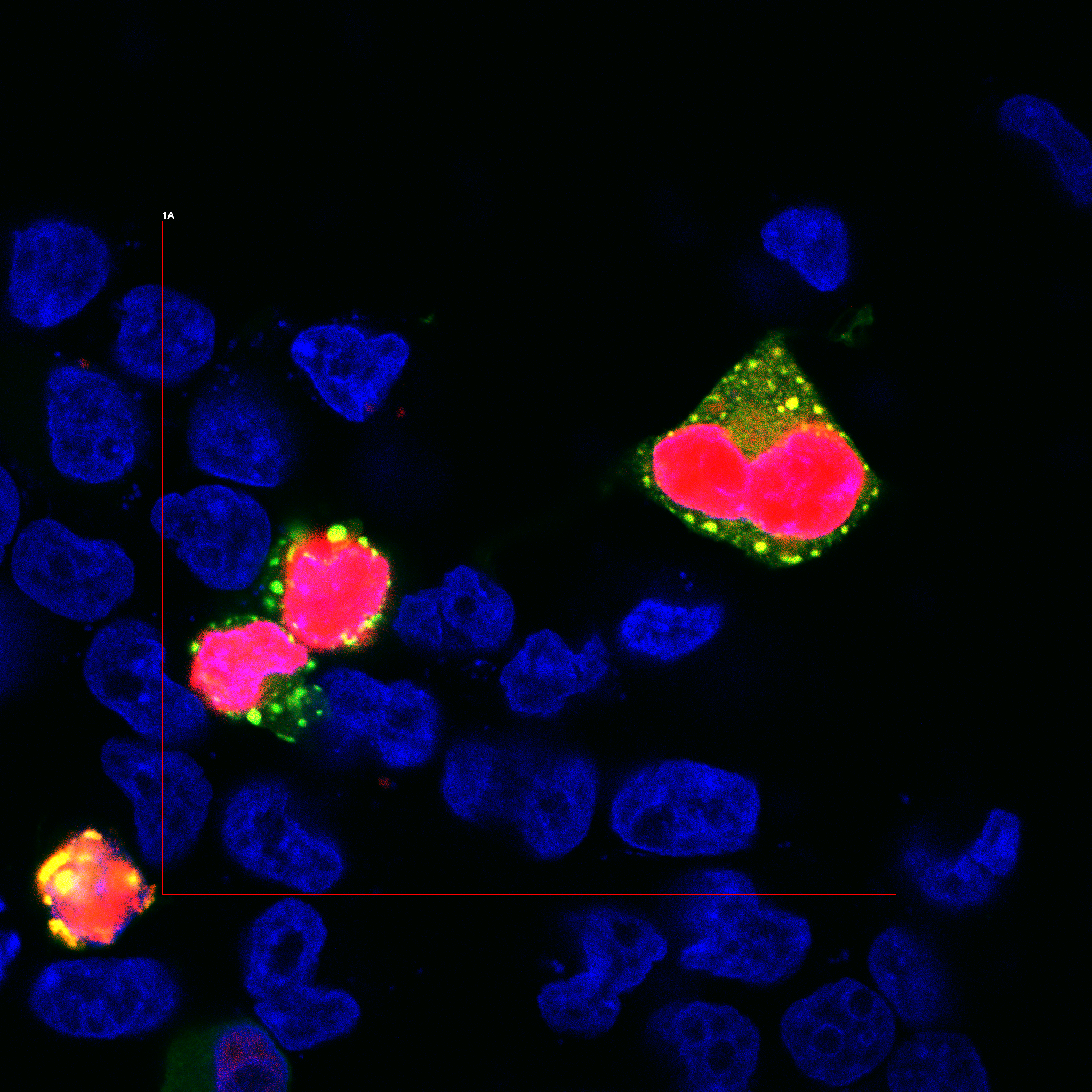

Supplement: Supplementary file 12 — Source data Fig. 4 [file 44321_2025_200_MOESM12_ESM.zip › EMM-2024-20400_SourceDataForFigure 4/Figure 4E/DMSO/Merge.tif]

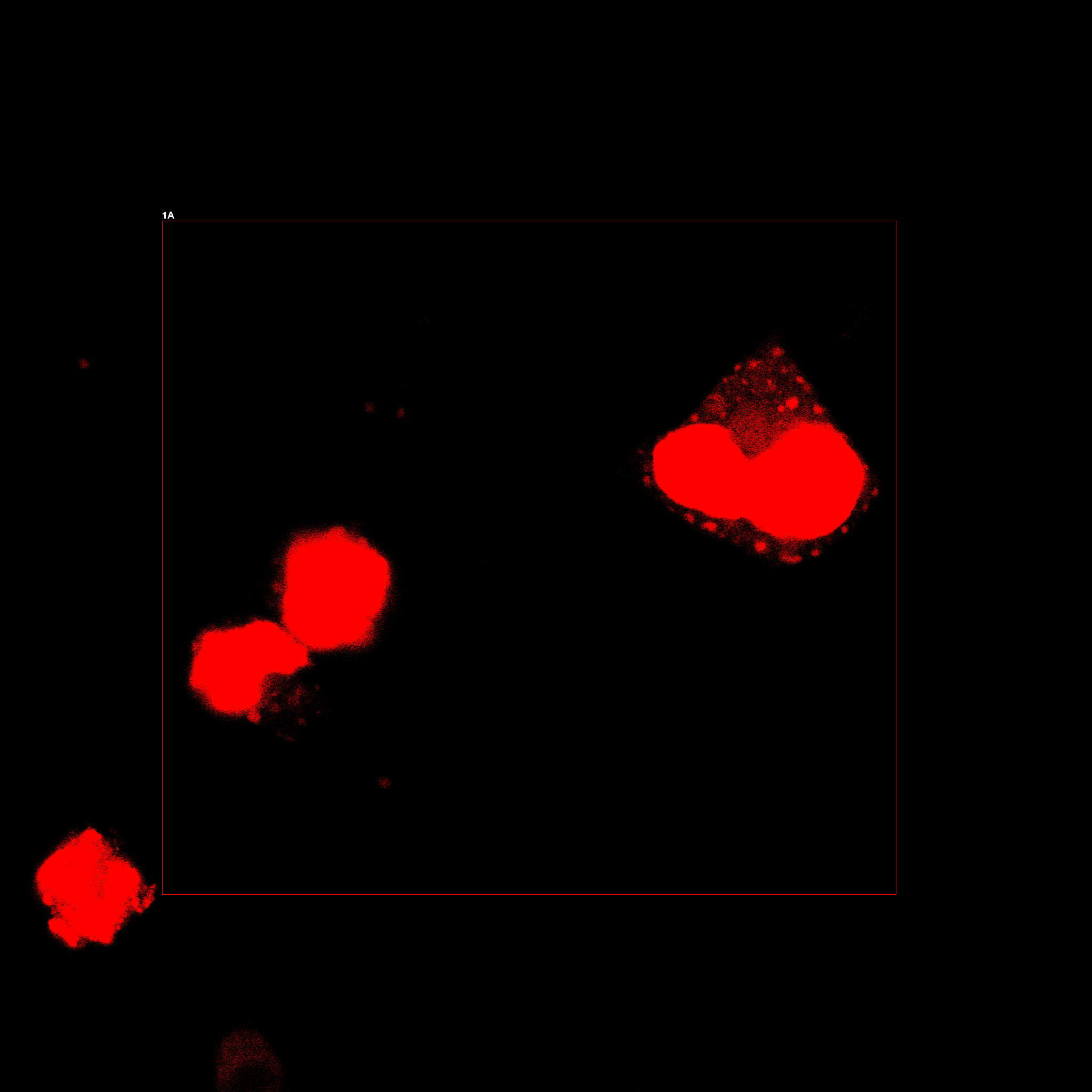

Supplement: Supplementary file 12 — Source data Fig. 4 [file 44321_2025_200_MOESM12_ESM.zip › EMM-2024-20400_SourceDataForFigure 4/Figure 4E/DMSO/RUNX1-tdTomato.tif]

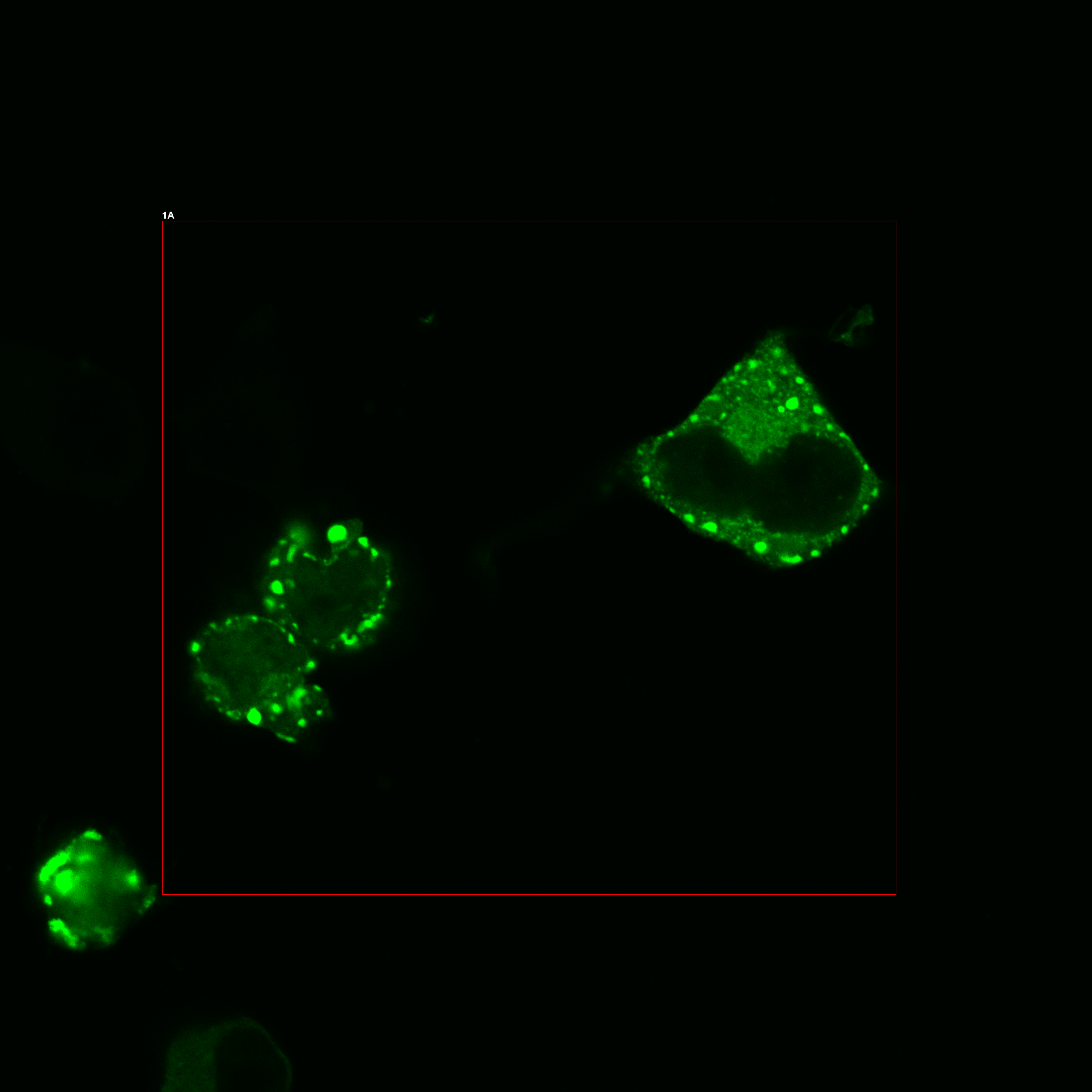

Supplement: Supplementary file 12 — Source data Fig. 4 [file 44321_2025_200_MOESM12_ESM.zip › EMM-2024-20400_SourceDataForFigure 4/Figure 4E/DMSO/STUB1-GFP.tif]

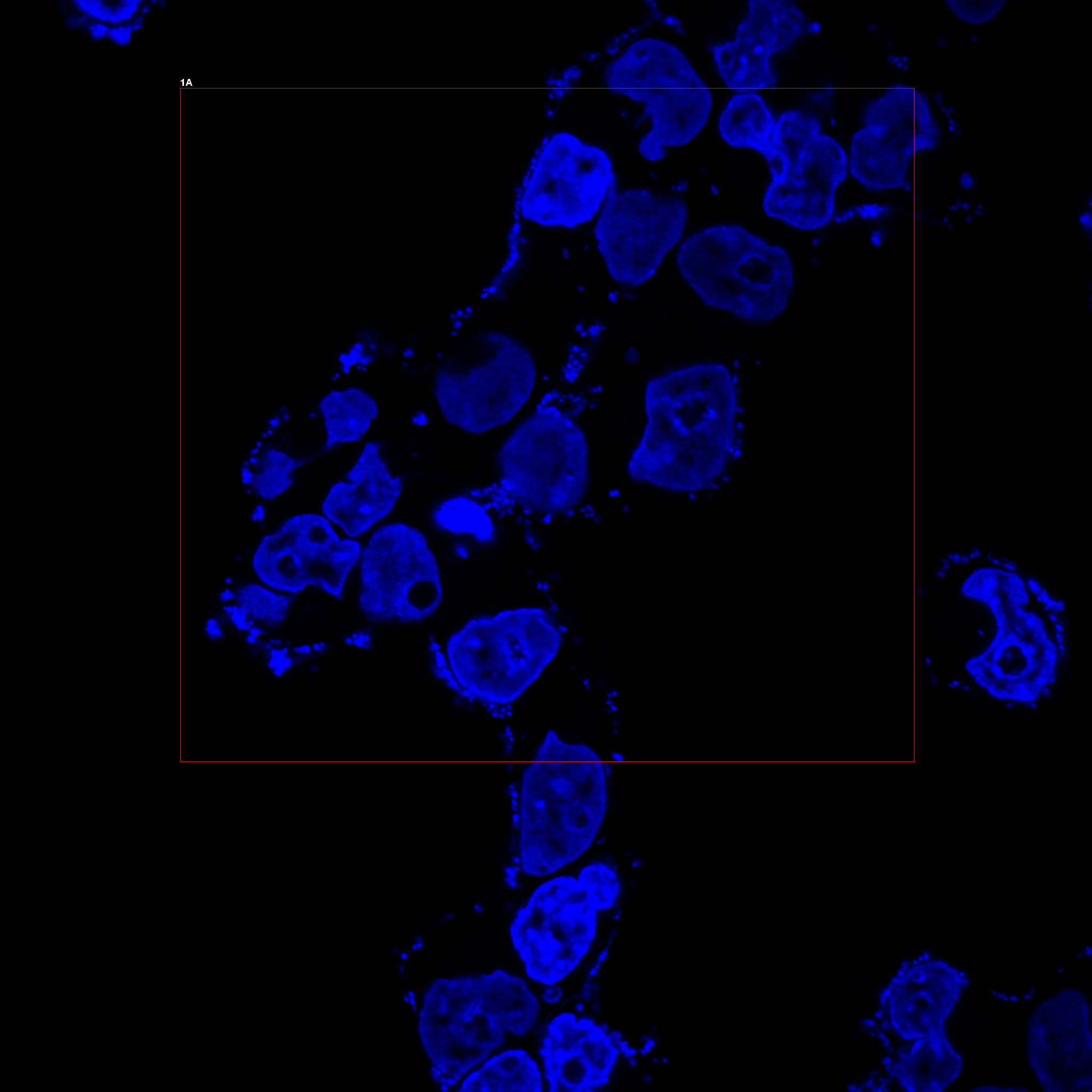

Supplement: Supplementary file 12 — Source data Fig. 4 [file 44321_2025_200_MOESM12_ESM.zip › EMM-2024-20400_SourceDataForFigure 4/Figure 4E/Lac/DAPI.tif]

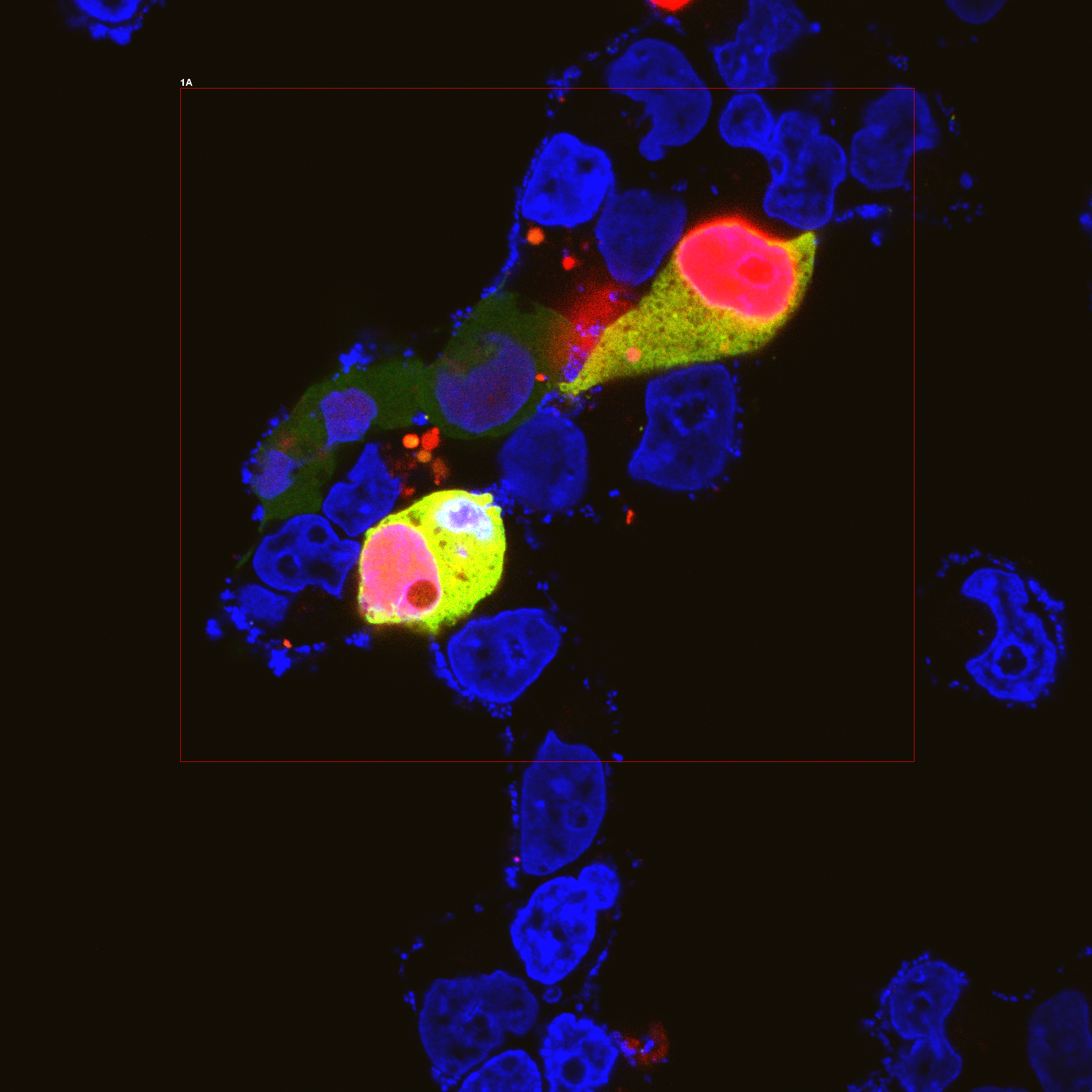

Supplement: Supplementary file 12 — Source data Fig. 4 [file 44321_2025_200_MOESM12_ESM.zip › EMM-2024-20400_SourceDataForFigure 4/Figure 4E/Lac/Merge.tif]

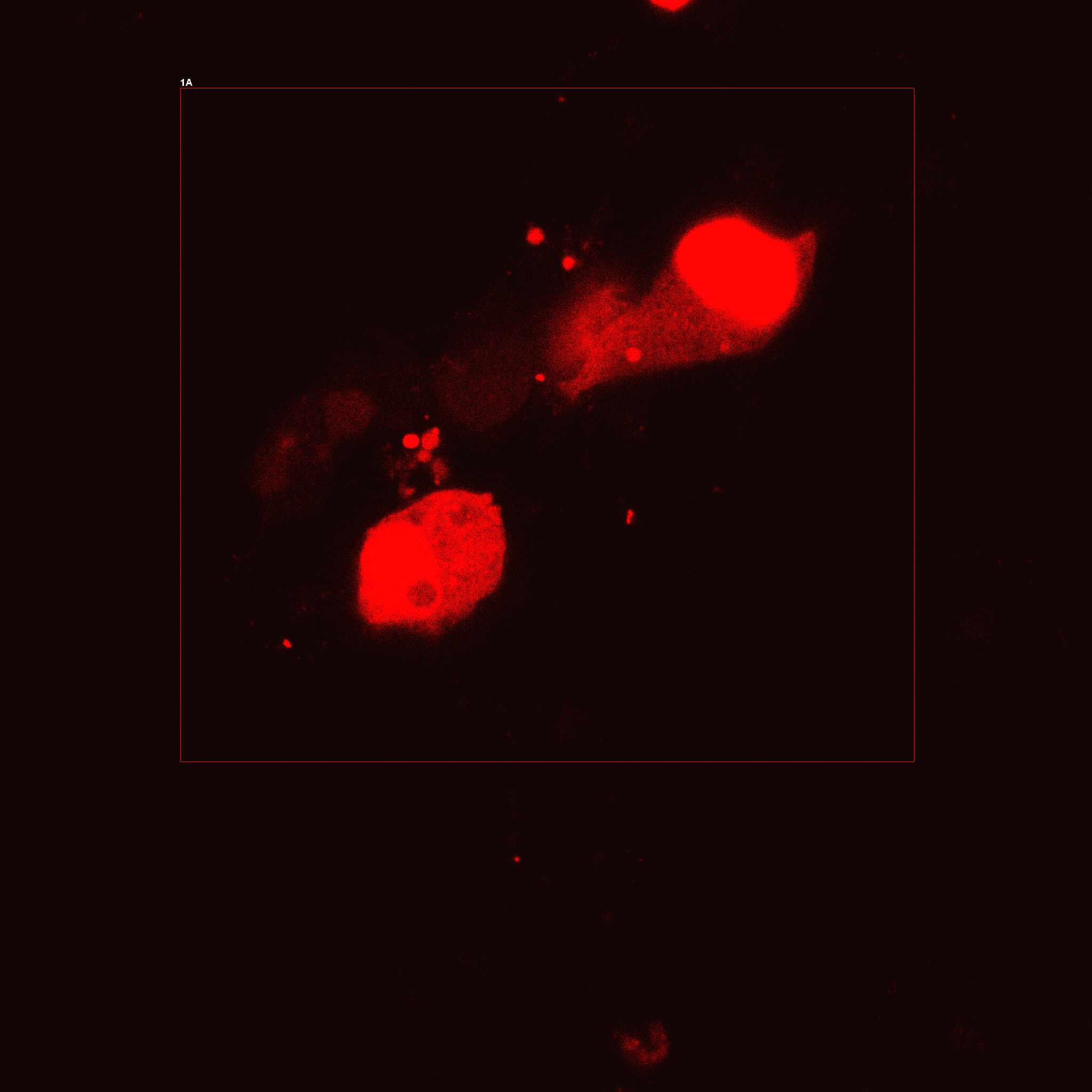

Supplement: Supplementary file 12 — Source data Fig. 4 [file 44321_2025_200_MOESM12_ESM.zip › EMM-2024-20400_SourceDataForFigure 4/Figure 4E/Lac/RUNX1-tdTomato.tif]

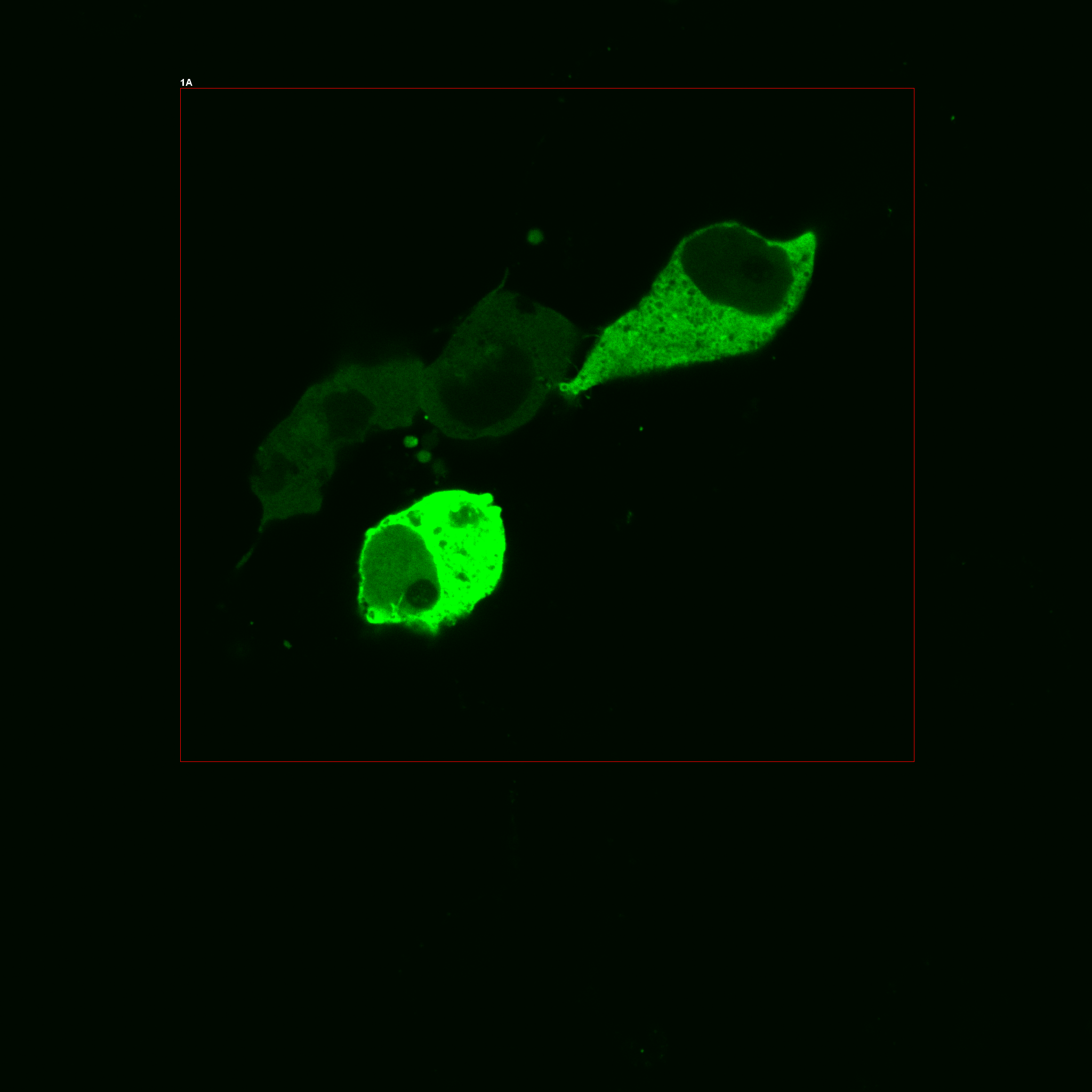

Supplement: Supplementary file 12 — Source data Fig. 4 [file 44321_2025_200_MOESM12_ESM.zip › EMM-2024-20400_SourceDataForFigure 4/Figure 4E/Lac/STUB1-GFP.tif]

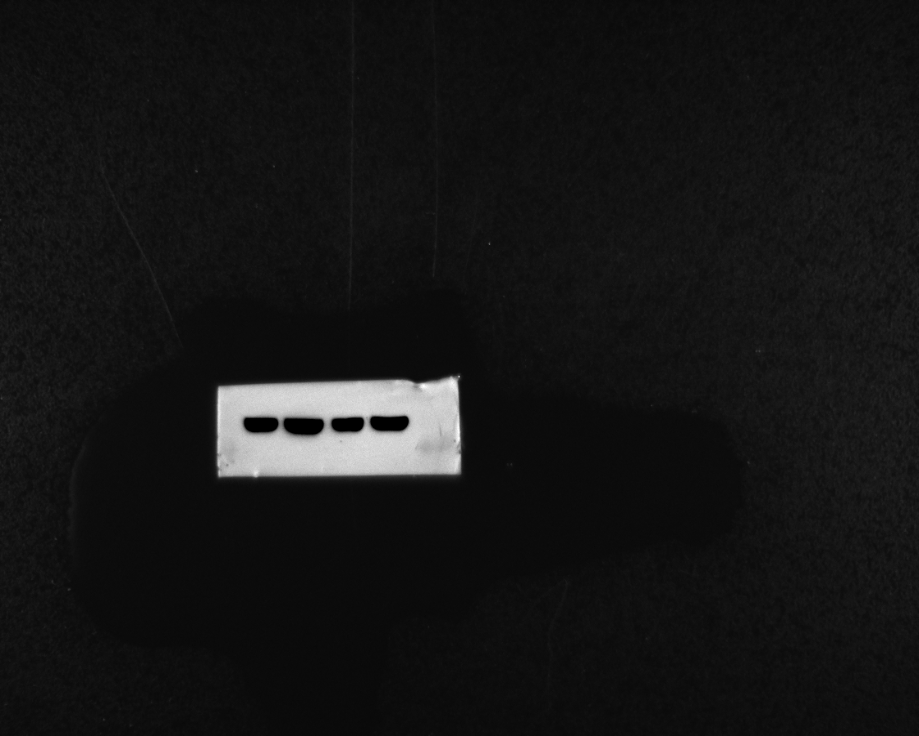

Supplement: Supplementary file 12 — Source data Fig. 4 [file 44321_2025_200_MOESM12_ESM.zip › EMM-2024-20400_SourceDataForFigure 4/Figure 4G/ACTIN-2.tif]

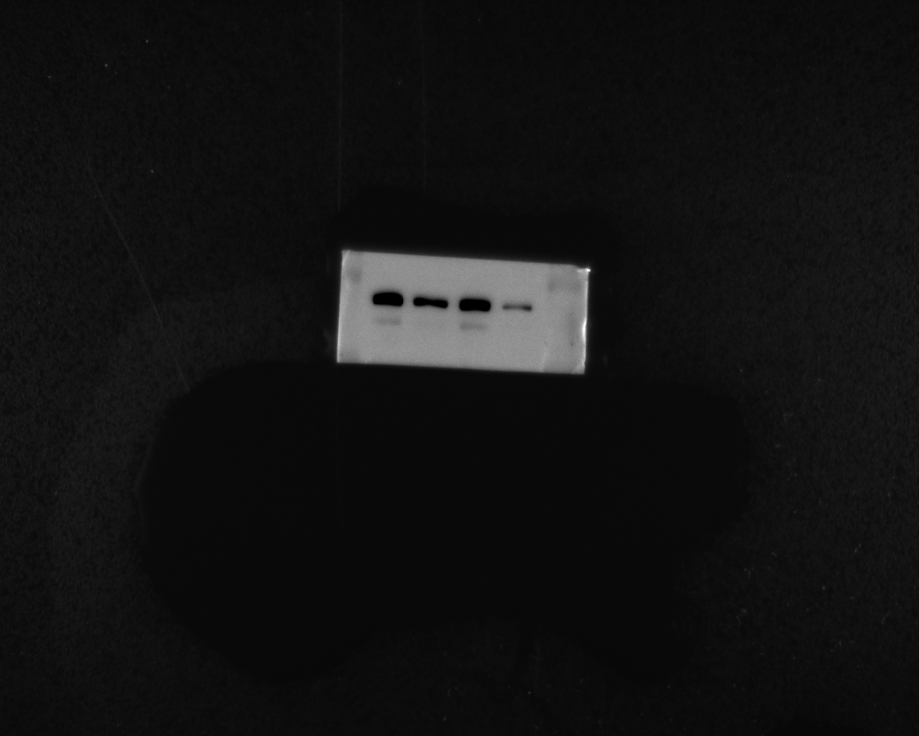

Supplement: Supplementary file 12 — Source data Fig. 4 [file 44321_2025_200_MOESM12_ESM.zip › EMM-2024-20400_SourceDataForFigure 4/Figure 4G/RUNX1-2.tif]

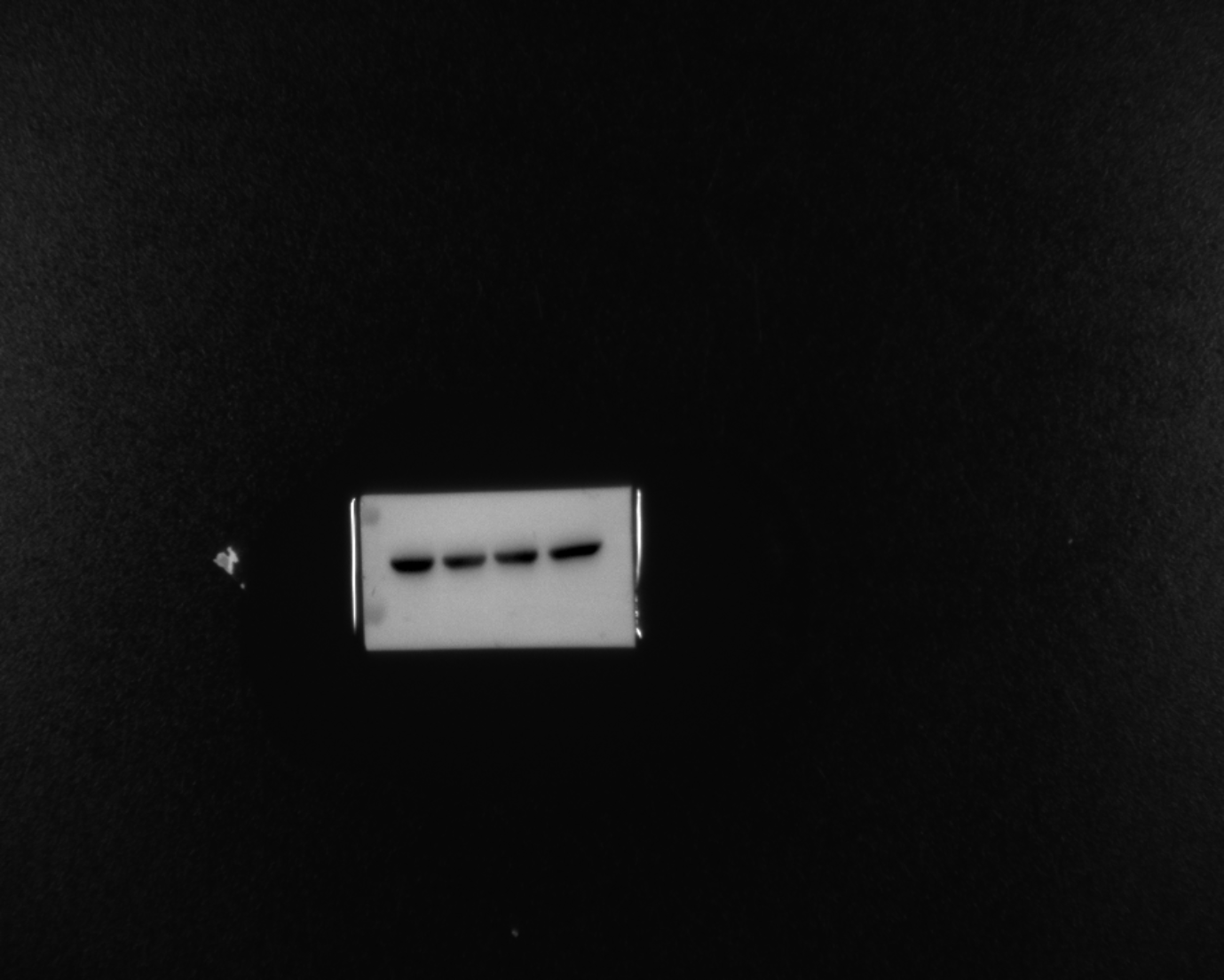

Supplement: Supplementary file 12 — Source data Fig. 4 [file 44321_2025_200_MOESM12_ESM.zip › EMM-2024-20400_SourceDataForFigure 4/Figure 4H/ACTIN.tif]

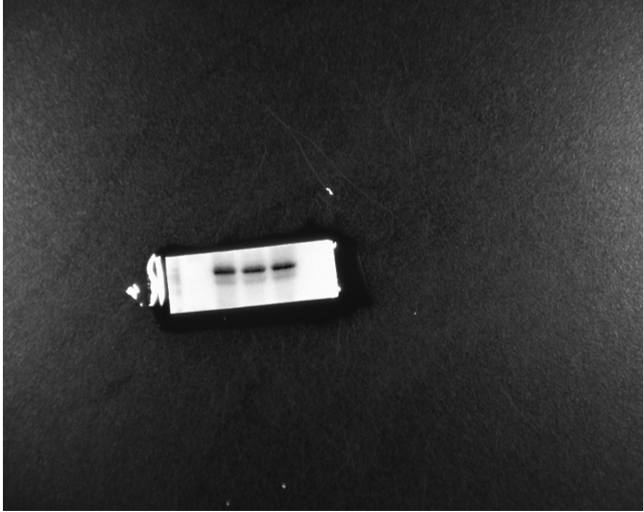

Supplement: Supplementary file 12 — Source data Fig. 4 [file 44321_2025_200_MOESM12_ESM.zip › EMM-2024-20400_SourceDataForFigure 4/Figure 4H/IP-RUNX1.tif]

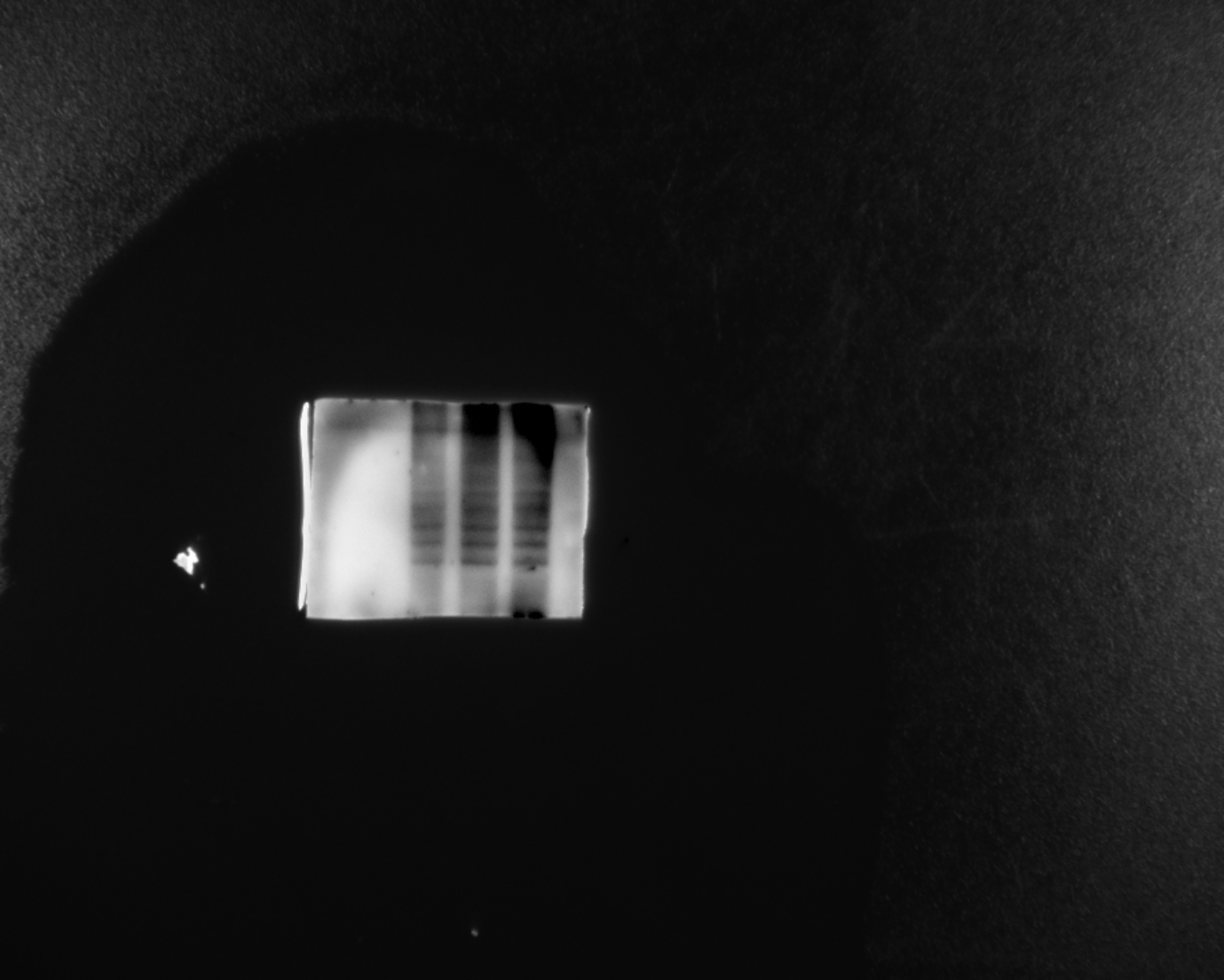

Supplement: Supplementary file 12 — Source data Fig. 4 [file 44321_2025_200_MOESM12_ESM.zip › EMM-2024-20400_SourceDataForFigure 4/Figure 4H/IP-UB-Myc.tif]
