# Supplementary figures and images for: Lanatoside C activates the E3 ligase STUB1 to inhibit FOXP3 transcriptional activity and promote antitumor immunity (part 2 of 2)
Source: EMBO Mol Med. 2025 Feb 20;17(3):563–88. doi: 10.1038/s44321-025-00200-y (PMC11904033; doi:10.1038/s44321-025-00200-y)

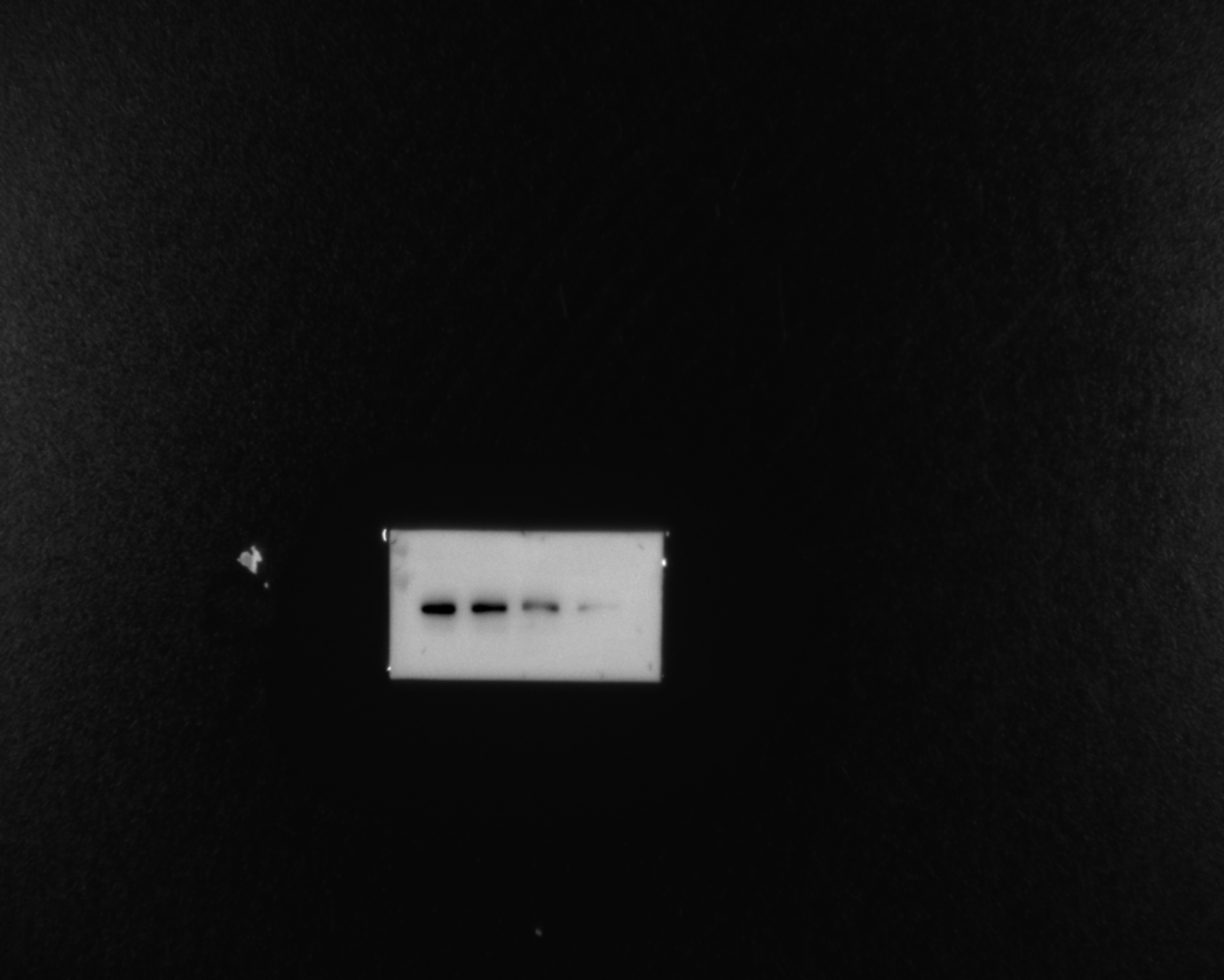

Supplement: Supplementary file 12 — Source data Fig. 4 [file 44321_2025_200_MOESM12_ESM.zip › EMM-2024-20400_SourceDataForFigure 4/Figure 4H/WCL-RUNX1.tif]

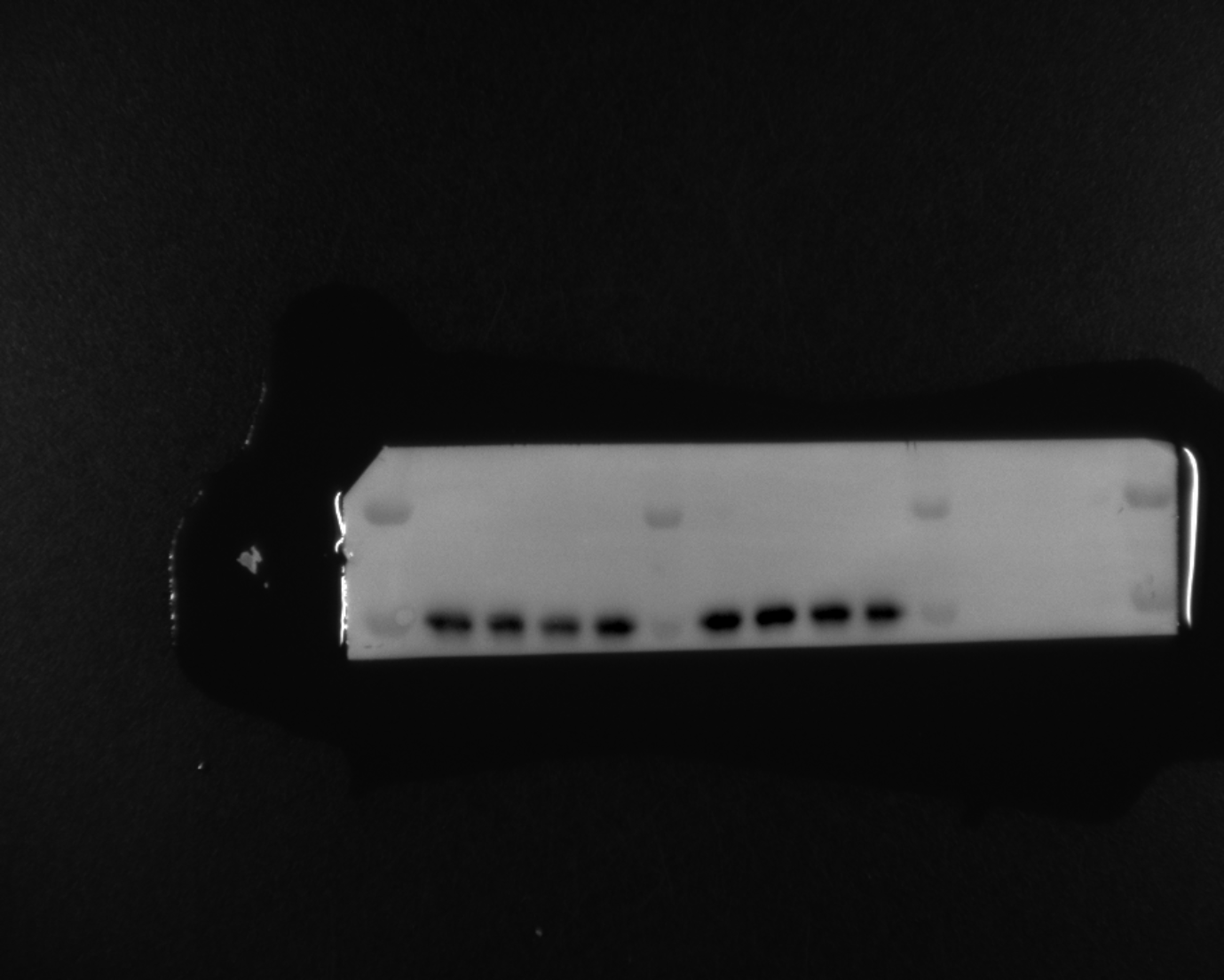

Supplement: Supplementary file 12 — Source data Fig. 4 [file 44321_2025_200_MOESM12_ESM.zip › EMM-2024-20400_SourceDataForFigure 4/Figure 4H/WCL-STUB1-HA.tif]

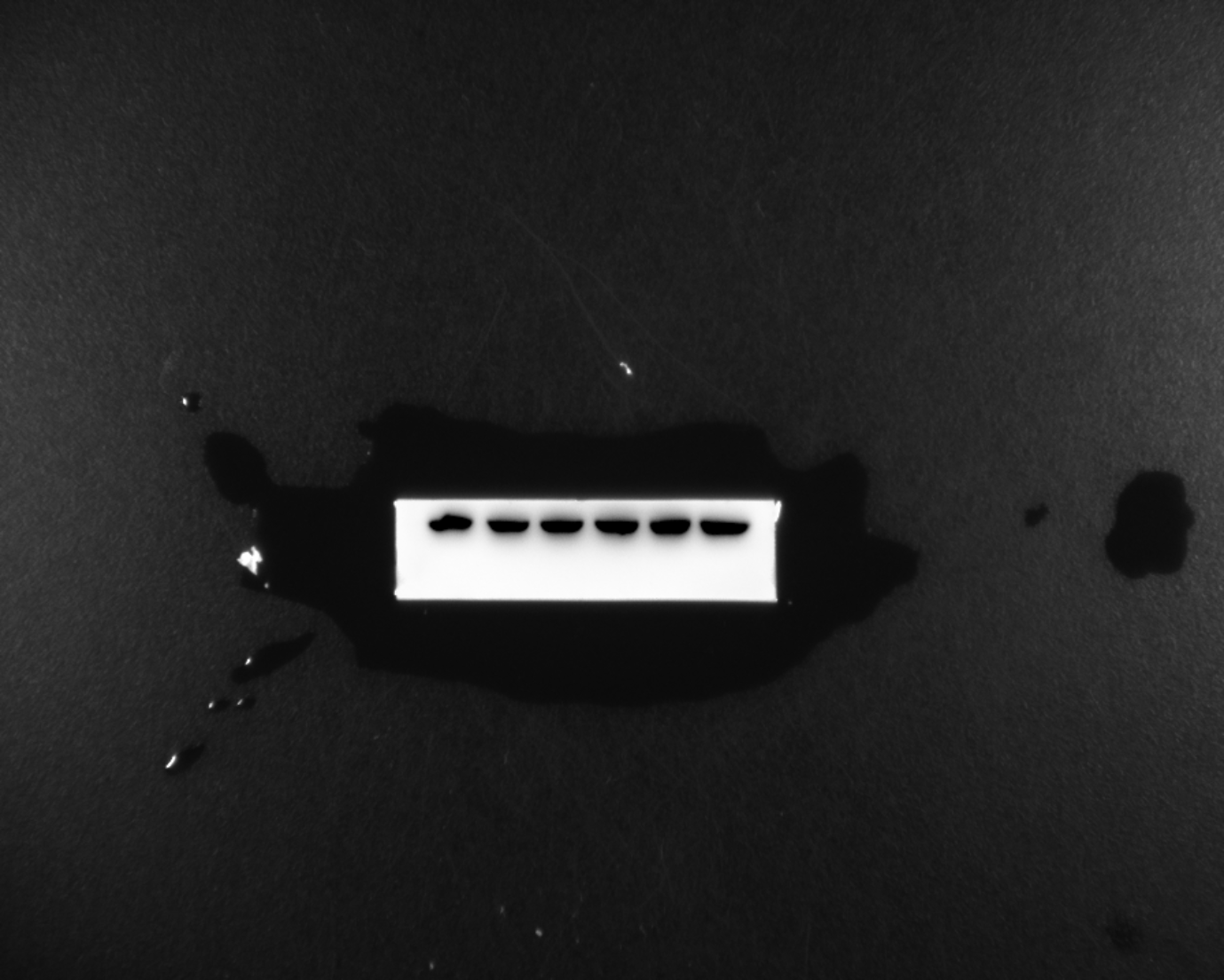

Supplement: Supplementary file 12 — Source data Fig. 4 [file 44321_2025_200_MOESM12_ESM.zip › EMM-2024-20400_SourceDataForFigure 4/Figure 4I/ACTIN.tif]

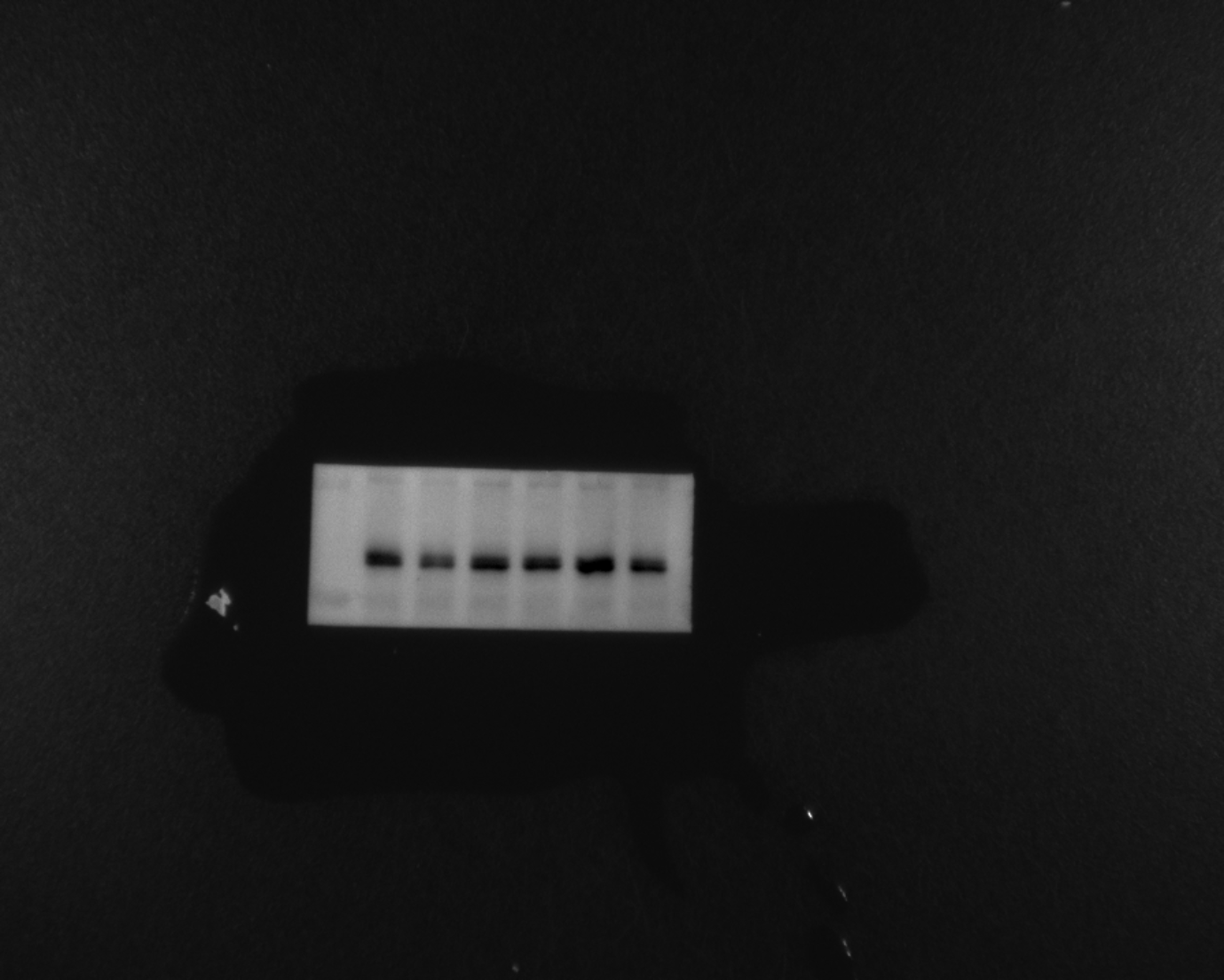

Supplement: Supplementary file 12 — Source data Fig. 4 [file 44321_2025_200_MOESM12_ESM.zip › EMM-2024-20400_SourceDataForFigure 4/Figure 4I/RUNX1.tif]

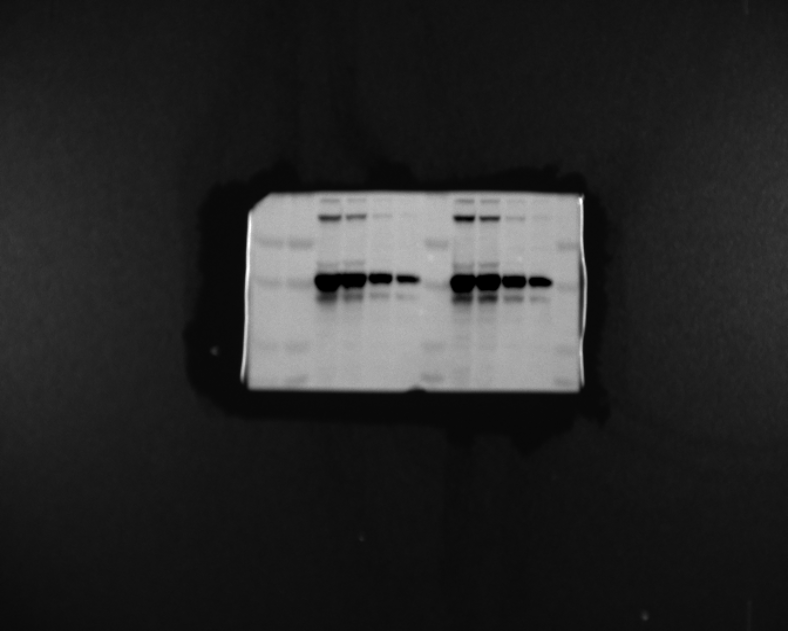

Supplement: Supplementary file 13 — Source data Fig. 5 [file 44321_2025_200_MOESM13_ESM.zip › EMM-2024-20400_SourceDataForFigure 5/Figure 5A/ACTIN.tif]

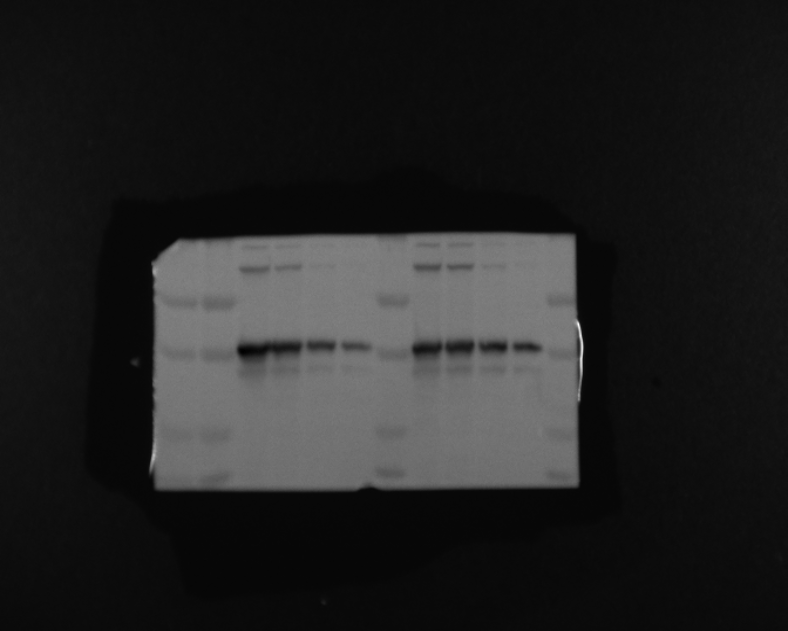

Supplement: Supplementary file 13 — Source data Fig. 5 [file 44321_2025_200_MOESM13_ESM.zip › EMM-2024-20400_SourceDataForFigure 5/Figure 5A/STUB1-HA.tif]

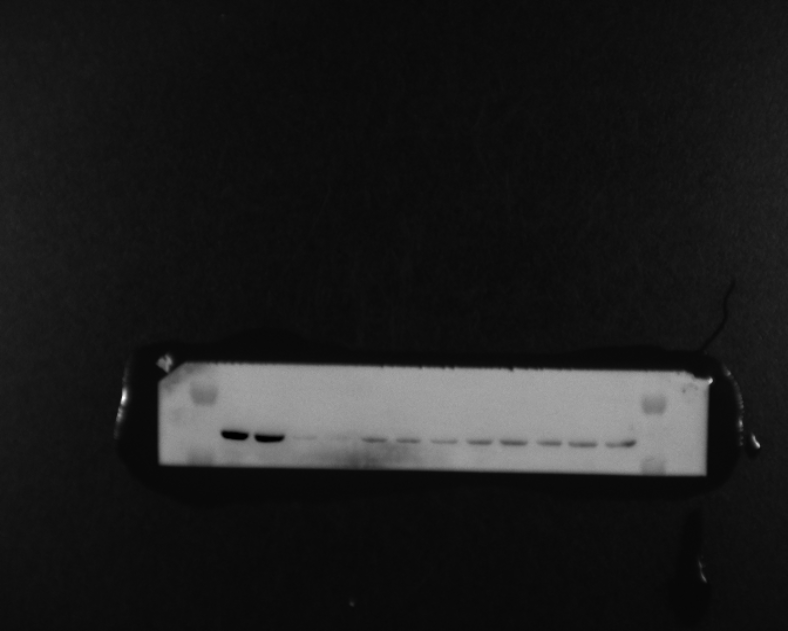

Supplement: Supplementary file 13 — Source data Fig. 5 [file 44321_2025_200_MOESM13_ESM.zip › EMM-2024-20400_SourceDataForFigure 5/Figure 5C/ACTIN.tif]

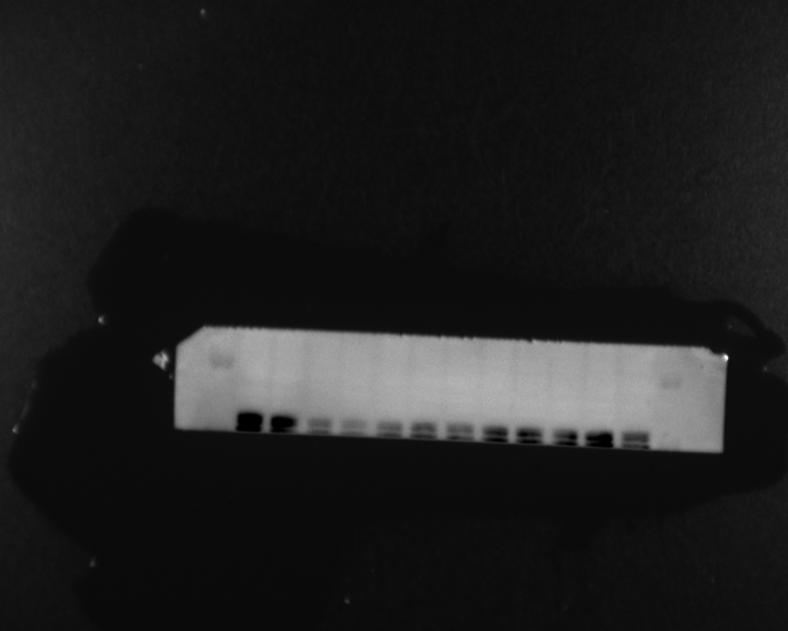

Supplement: Supplementary file 13 — Source data Fig. 5 [file 44321_2025_200_MOESM13_ESM.zip › EMM-2024-20400_SourceDataForFigure 5/Figure 5C/STUB1-HA.tif]

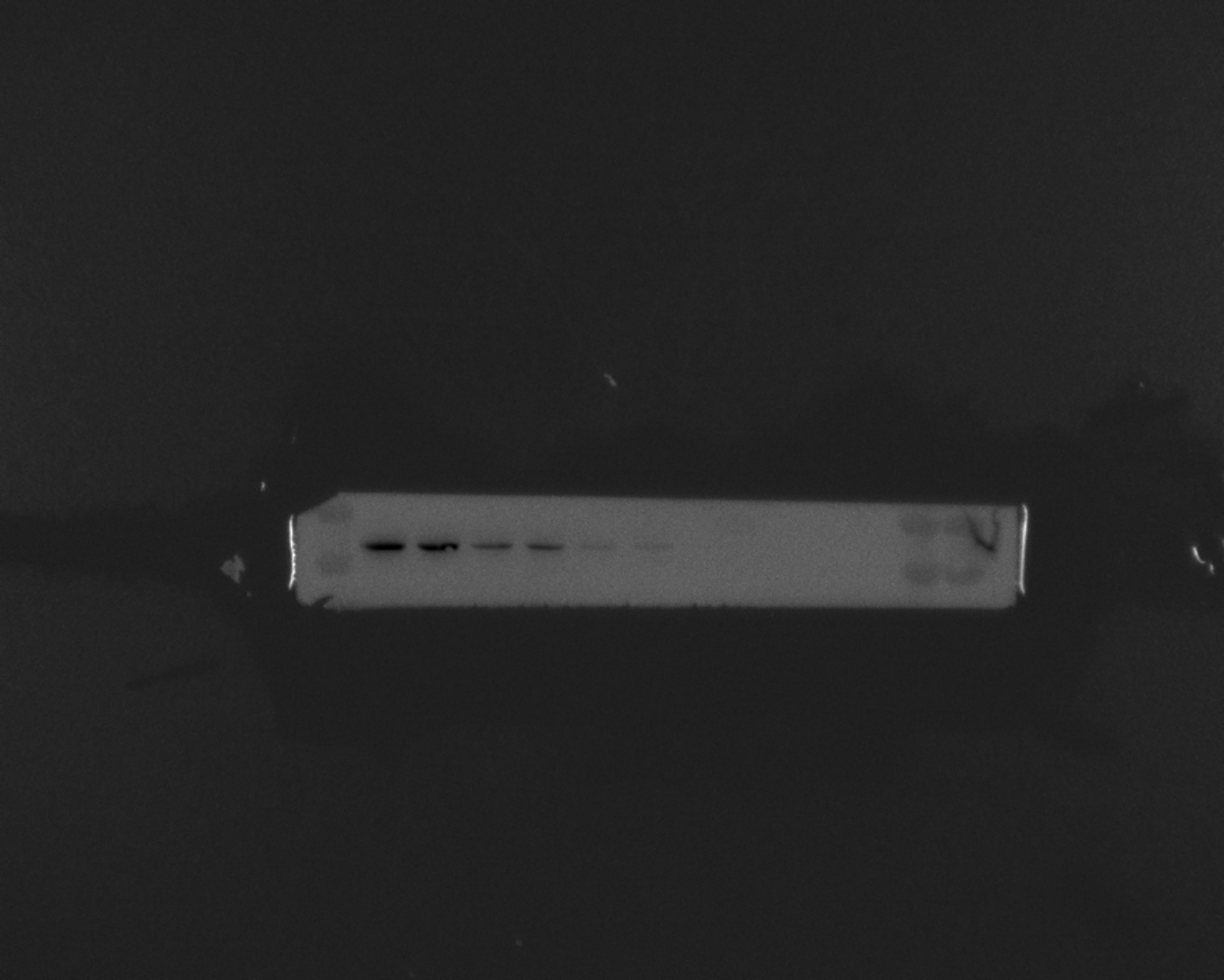

Supplement: Supplementary file 13 — Source data Fig. 5 [file 44321_2025_200_MOESM13_ESM.zip › EMM-2024-20400_SourceDataForFigure 5/Figure 5F/STUB1-1-130-ACTIN.tif]

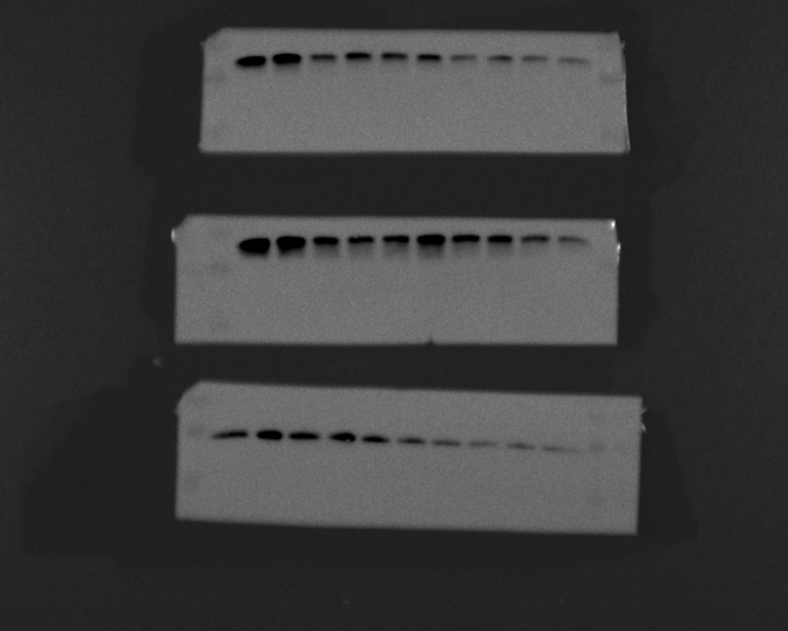

Supplement: Supplementary file 13 — Source data Fig. 5 [file 44321_2025_200_MOESM13_ESM.zip › EMM-2024-20400_SourceDataForFigure 5/Figure 5F/STUB1-1-130-Flag.tif]

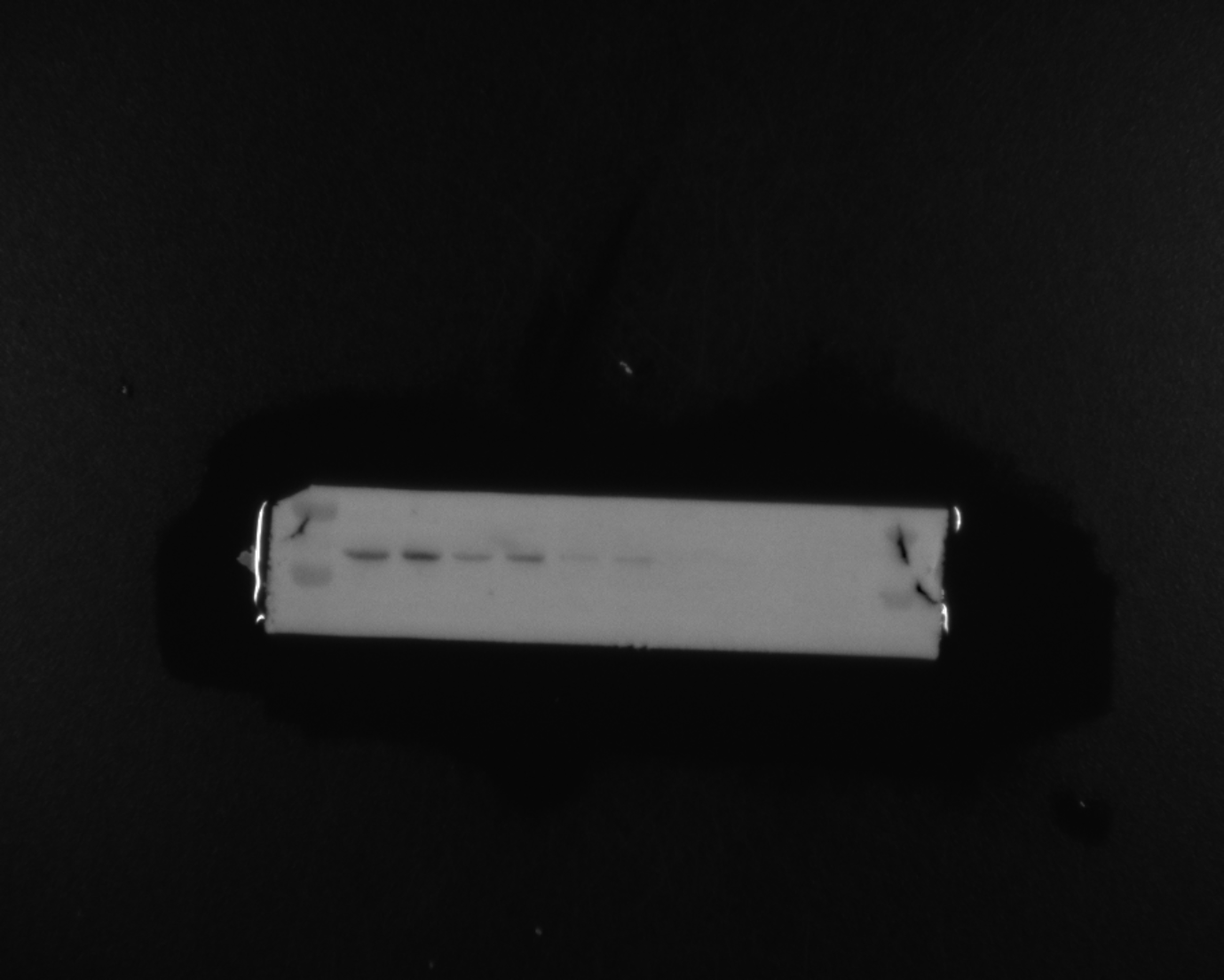

Supplement: Supplementary file 13 — Source data Fig. 5 [file 44321_2025_200_MOESM13_ESM.zip › EMM-2024-20400_SourceDataForFigure 5/Figure 5F/STUB1-127-226-ACTIN-.tif]

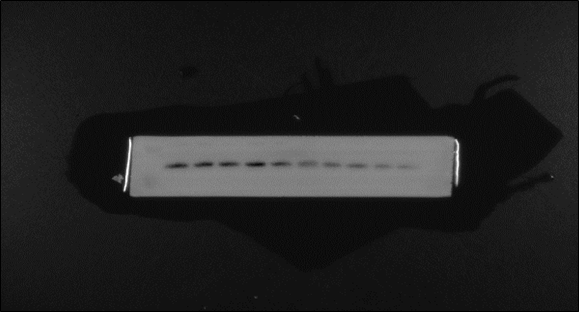

Supplement: Supplementary file 13 — Source data Fig. 5 [file 44321_2025_200_MOESM13_ESM.zip › EMM-2024-20400_SourceDataForFigure 5/Figure 5F/STUB1-127-226-Flag.tif]

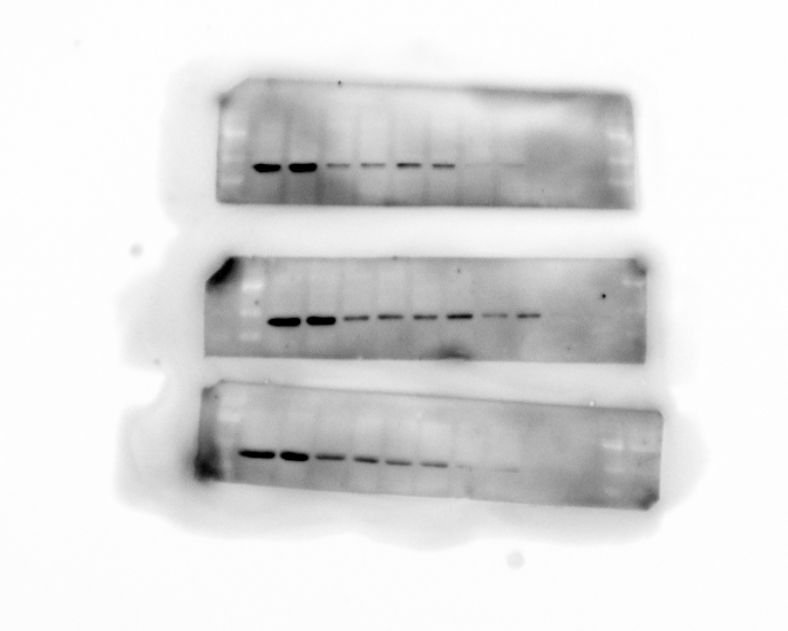

Supplement: Supplementary file 13 — Source data Fig. 5 [file 44321_2025_200_MOESM13_ESM.zip › EMM-2024-20400_SourceDataForFigure 5/Figure 5F/STUB1-223-303-ACTIN.tif]

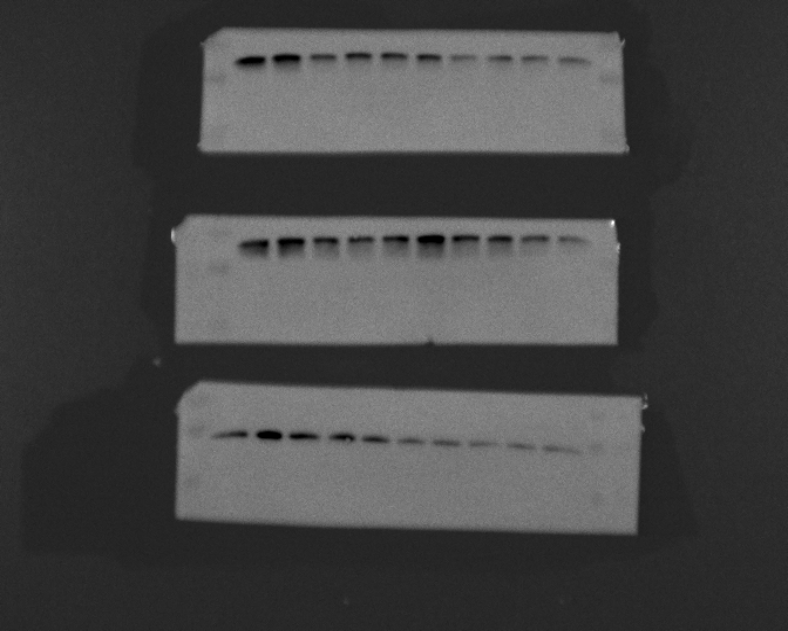

Supplement: Supplementary file 13 — Source data Fig. 5 [file 44321_2025_200_MOESM13_ESM.zip › EMM-2024-20400_SourceDataForFigure 5/Figure 5F/STUB1-223-303-Flag.tif]

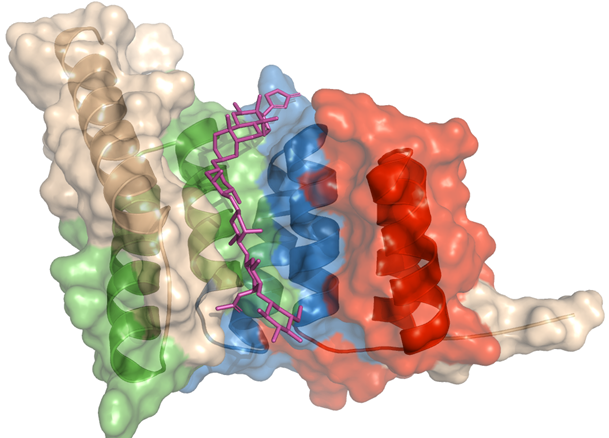

Supplement: Supplementary file 13 — Source data Fig. 5 [file 44321_2025_200_MOESM13_ESM.zip › EMM-2024-20400_SourceDataForFigure 5/Figure 5I/Figure 5I.png]

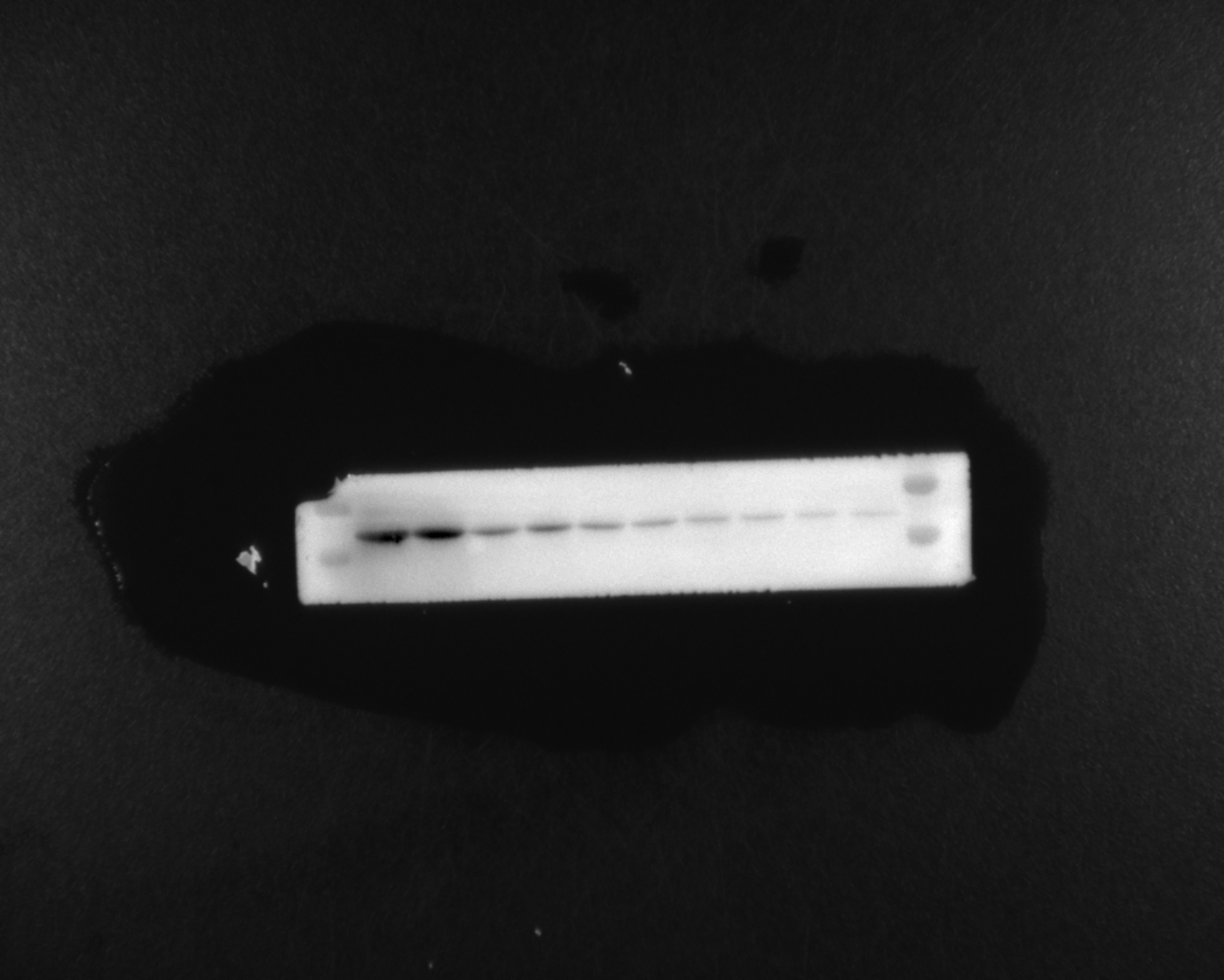

Supplement: Supplementary file 13 — Source data Fig. 5 [file 44321_2025_200_MOESM13_ESM.zip › EMM-2024-20400_SourceDataForFigure 5/Figure 5K/STUB1-K72A-Actin.tif]

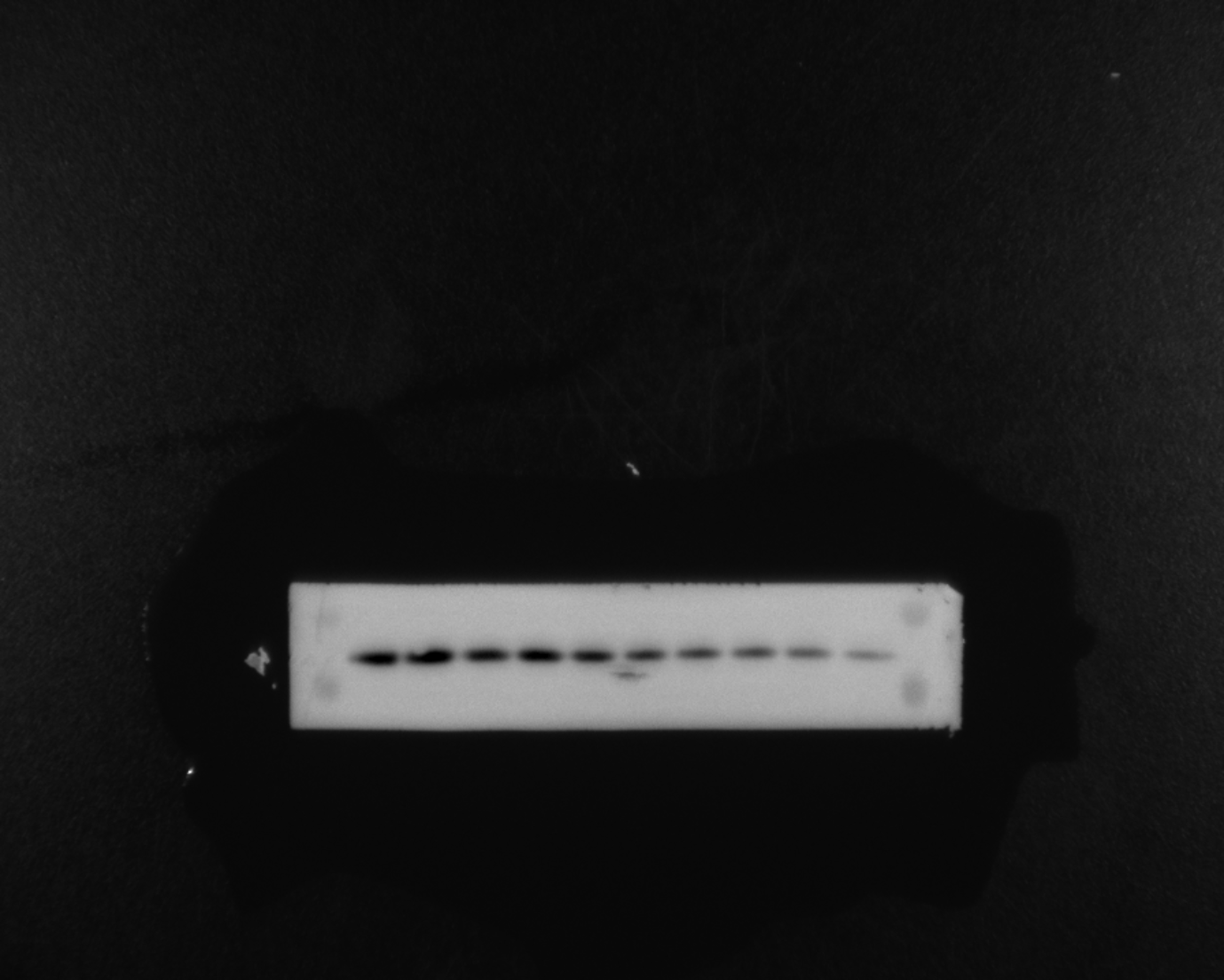

Supplement: Supplementary file 13 — Source data Fig. 5 [file 44321_2025_200_MOESM13_ESM.zip › EMM-2024-20400_SourceDataForFigure 5/Figure 5K/STUB1-K72A-Flag.tif]

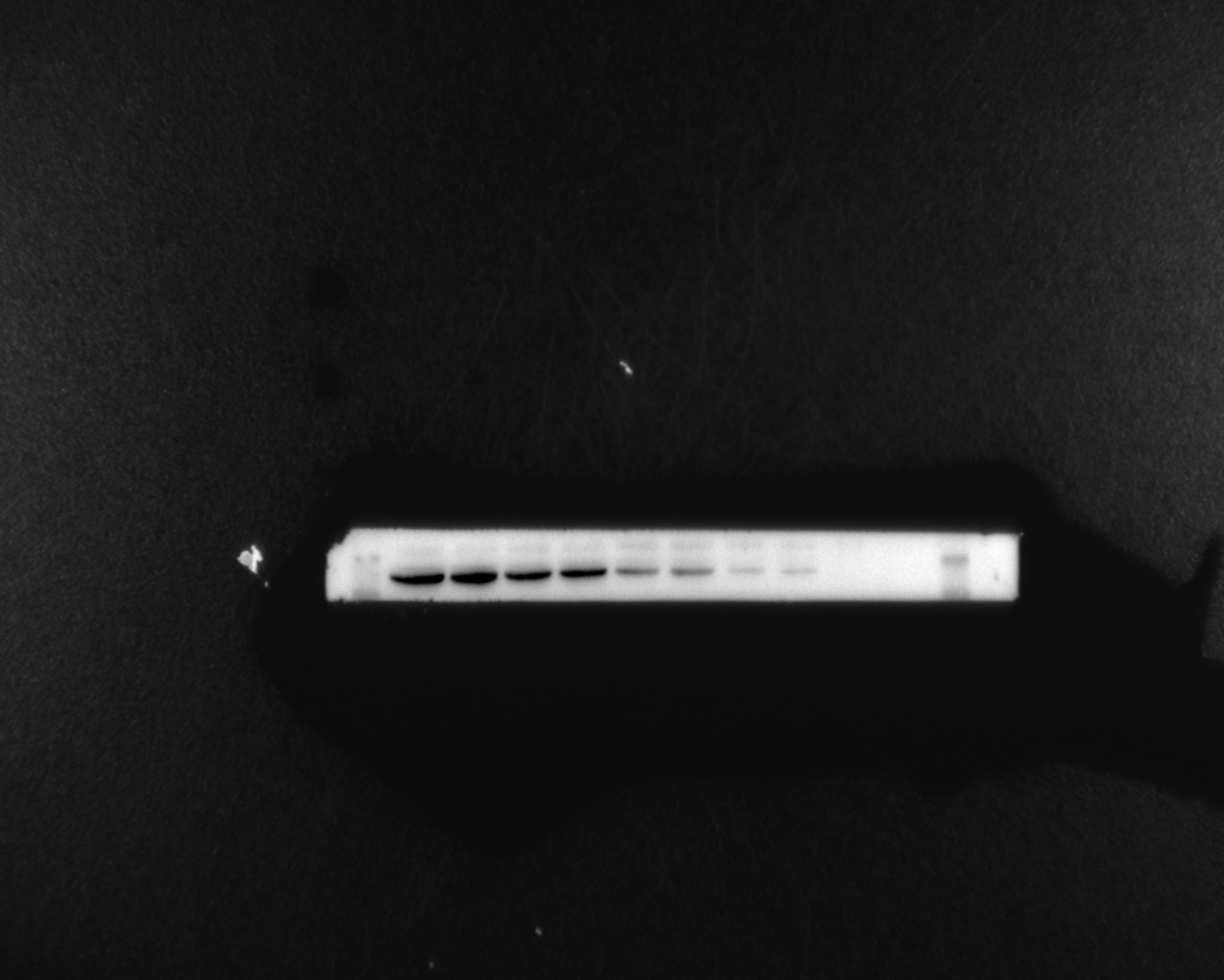

Supplement: Supplementary file 13 — Source data Fig. 5 [file 44321_2025_200_MOESM13_ESM.zip › EMM-2024-20400_SourceDataForFigure 5/Figure 5K/STUB1-Q102A-Actin.tif]

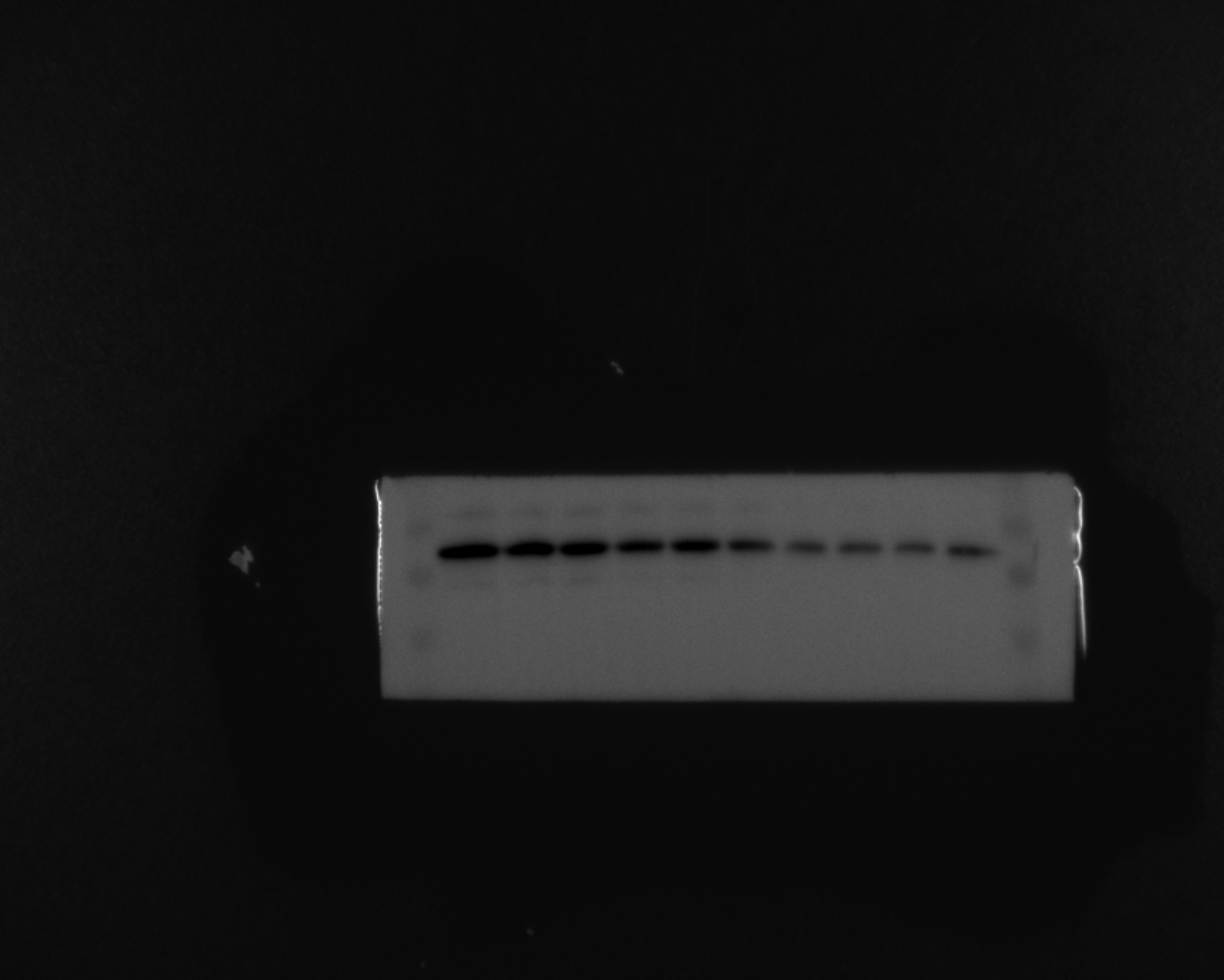

Supplement: Supplementary file 13 — Source data Fig. 5 [file 44321_2025_200_MOESM13_ESM.zip › EMM-2024-20400_SourceDataForFigure 5/Figure 5K/STUB1-Q102A-Flag.tif]

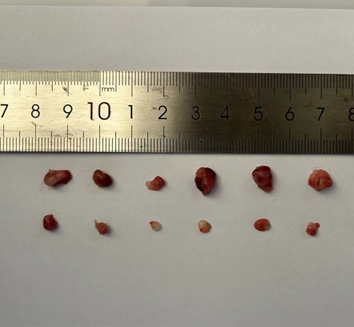

Supplement: Supplementary file 14 — Source data Fig. 6 [file 44321_2025_200_MOESM14_ESM.zip › EMM-2024-20400_SourceDataForFigure 6/Figure 6A/Tumor(LLC).png]

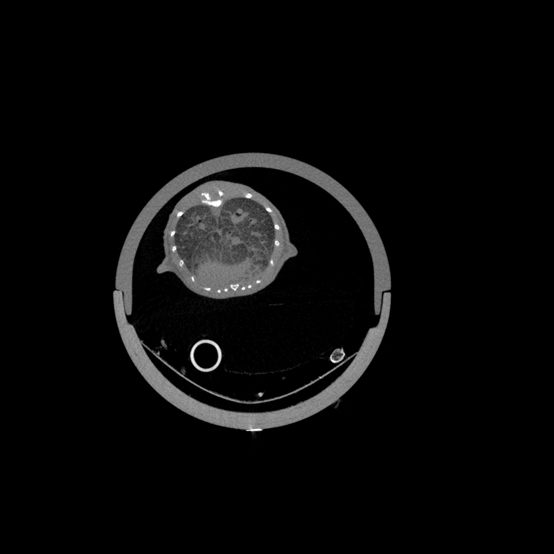

Supplement: Supplementary file 14 — Source data Fig. 6 [file 44321_2025_200_MOESM14_ESM.zip › EMM-2024-20400_SourceDataForFigure 6/Figure 6H/PreRX DT(EGFR-DEL).tif]

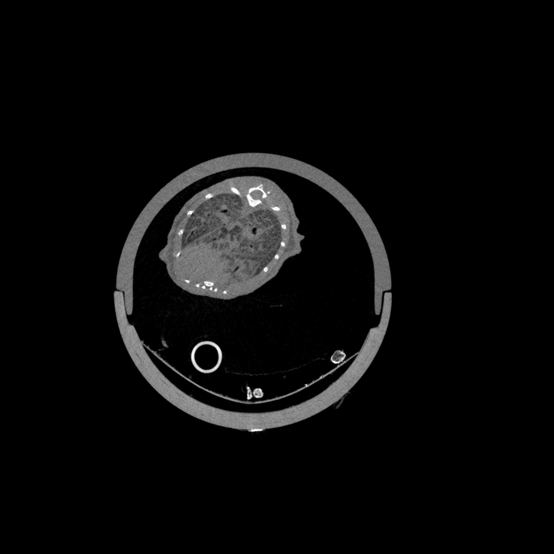

Supplement: Supplementary file 14 — Source data Fig. 6 [file 44321_2025_200_MOESM14_ESM.zip › EMM-2024-20400_SourceDataForFigure 6/Figure 6H/PreRX DT(IR-EGFR-DEL+FOXP3-DTR-GFP BM).tif]

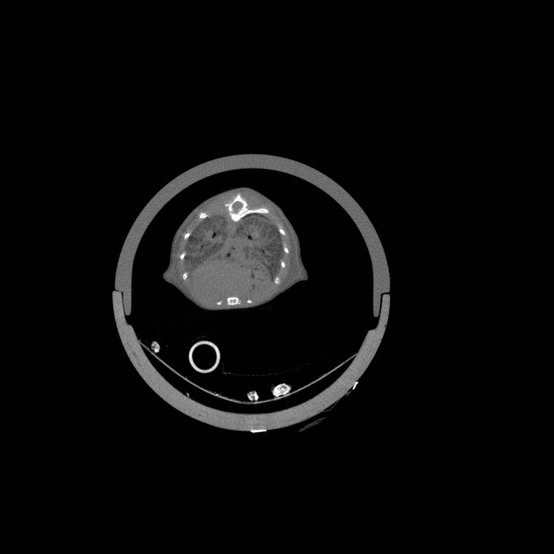

Supplement: Supplementary file 14 — Source data Fig. 6 [file 44321_2025_200_MOESM14_ESM.zip › EMM-2024-20400_SourceDataForFigure 6/Figure 6H/PreRX Lac(EGFR-DEL).tif]

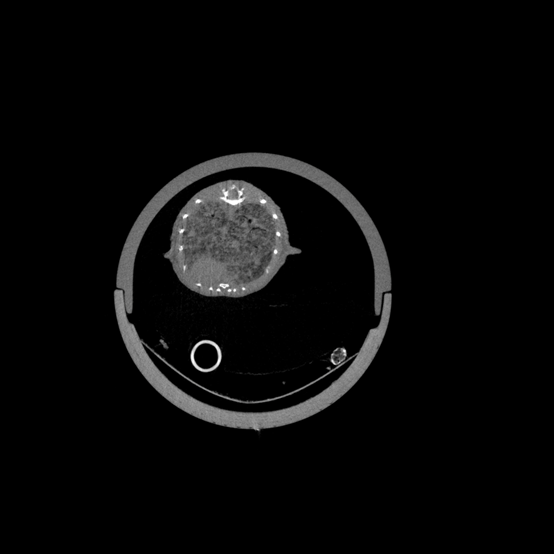

Supplement: Supplementary file 14 — Source data Fig. 6 [file 44321_2025_200_MOESM14_ESM.zip › EMM-2024-20400_SourceDataForFigure 6/Figure 6H/PreRX Lac(IR-EGFR-DEL+FOXP3-DTR-GFP BM).tif]

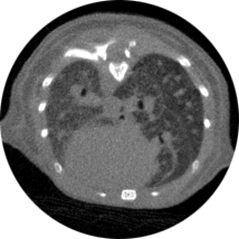

Supplement: Supplementary file 14 — Source data Fig. 6 [file 44321_2025_200_MOESM14_ESM.zip › EMM-2024-20400_SourceDataForFigure 6/Figure 6H/PreRX Veh (EGFR-DEL).tif]

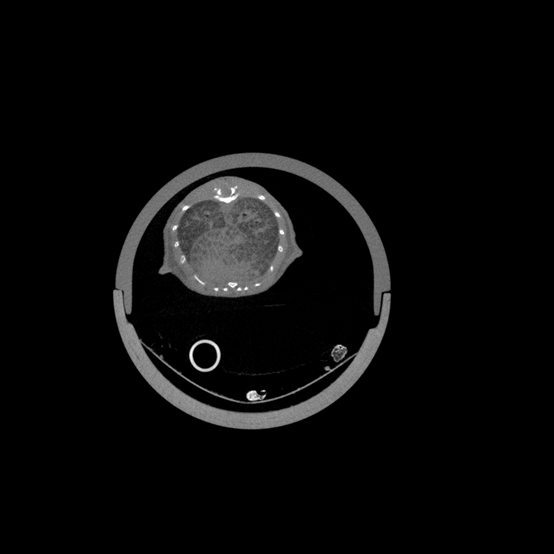

Supplement: Supplementary file 14 — Source data Fig. 6 [file 44321_2025_200_MOESM14_ESM.zip › EMM-2024-20400_SourceDataForFigure 6/Figure 6H/PreRX Veh(IR-EGFR-DEL+FOXP3-DTR-GFP BM).tif]

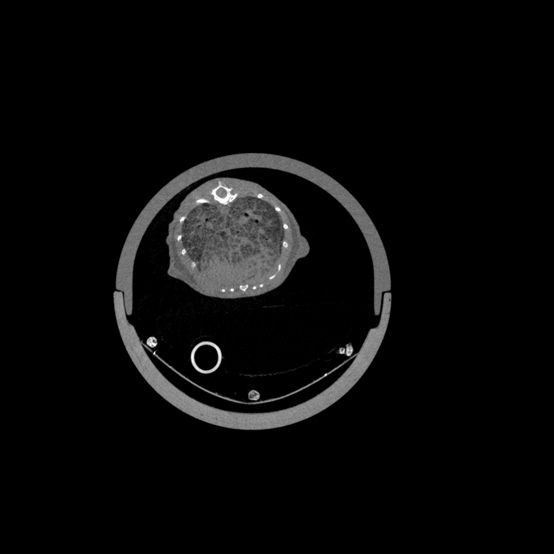

Supplement: Supplementary file 14 — Source data Fig. 6 [file 44321_2025_200_MOESM14_ESM.zip › EMM-2024-20400_SourceDataForFigure 6/Figure 6H/PstRX DT(EGFR-DEL).tif]

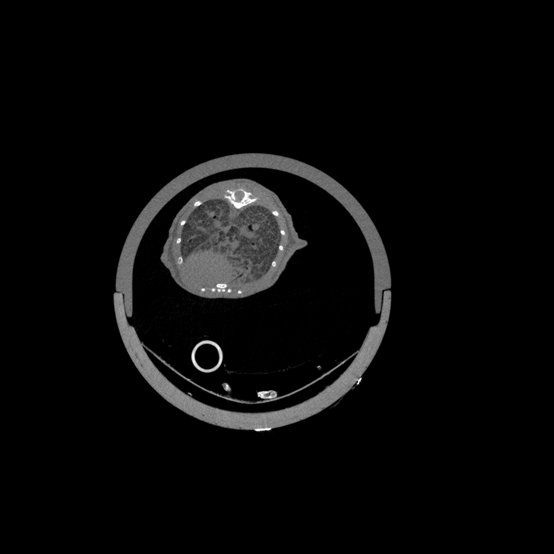

Supplement: Supplementary file 14 — Source data Fig. 6 [file 44321_2025_200_MOESM14_ESM.zip › EMM-2024-20400_SourceDataForFigure 6/Figure 6H/PstRX DT(IR-EGFR-DEL+FOXP3-DTR-GFP BM).tif]

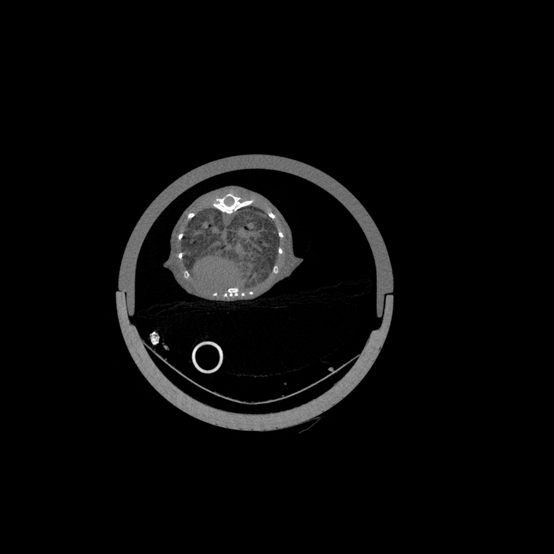

Supplement: Supplementary file 14 — Source data Fig. 6 [file 44321_2025_200_MOESM14_ESM.zip › EMM-2024-20400_SourceDataForFigure 6/Figure 6H/PstRX Lac(EGFR-DEL).tif]

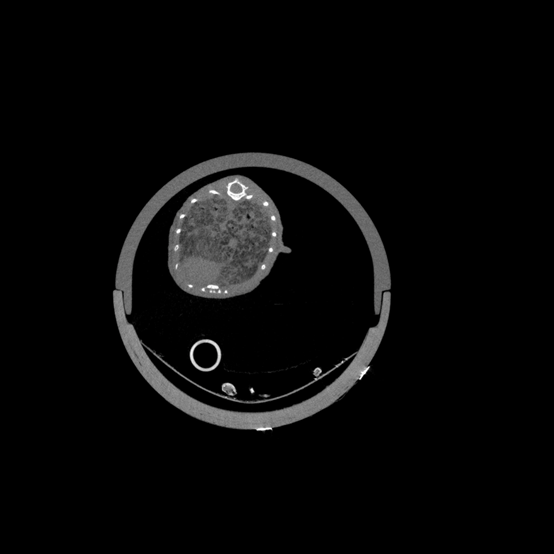

Supplement: Supplementary file 14 — Source data Fig. 6 [file 44321_2025_200_MOESM14_ESM.zip › EMM-2024-20400_SourceDataForFigure 6/Figure 6H/PstRX Lac(IR-EGFR-DEL+FOXP3-DTR-GFP BM).tif]

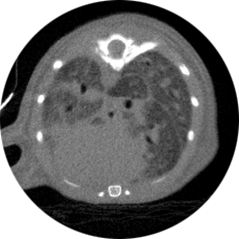

Supplement: Supplementary file 14 — Source data Fig. 6 [file 44321_2025_200_MOESM14_ESM.zip › EMM-2024-20400_SourceDataForFigure 6/Figure 6H/PstRX Veh (EGFR-DEL).tif]

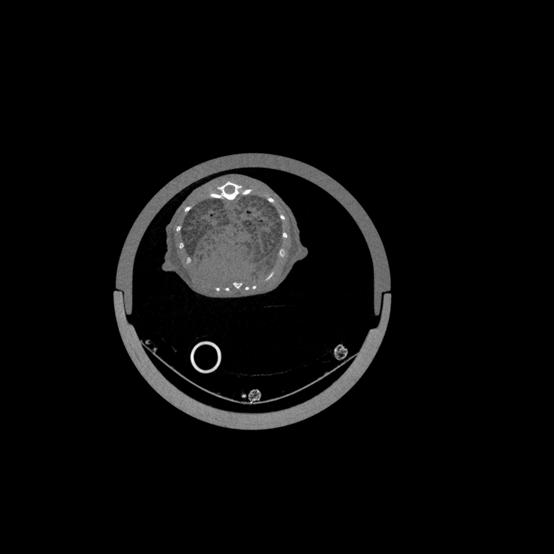

Supplement: Supplementary file 14 — Source data Fig. 6 [file 44321_2025_200_MOESM14_ESM.zip › EMM-2024-20400_SourceDataForFigure 6/Figure 6H/PstRX Veh(IR-EGFR-DEL+FOXP3-DTR-GFP BM).tif]

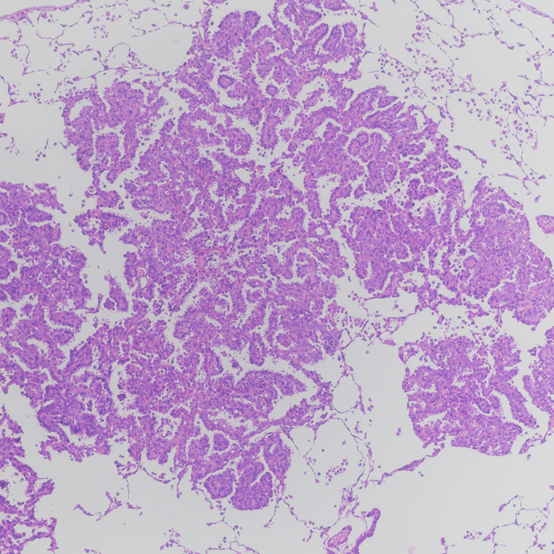

Supplement: Supplementary file 14 — Source data Fig. 6 [file 44321_2025_200_MOESM14_ESM.zip › EMM-2024-20400_SourceDataForFigure 6/Figure 6J/DT(EGFR-DEL).tif]

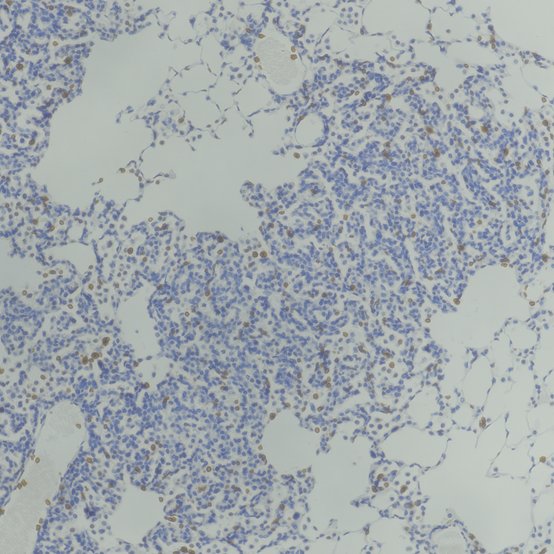

Supplement: Supplementary file 14 — Source data Fig. 6 [file 44321_2025_200_MOESM14_ESM.zip › EMM-2024-20400_SourceDataForFigure 6/Figure 6J/DT(EGFR-DEL)ki67.tif]

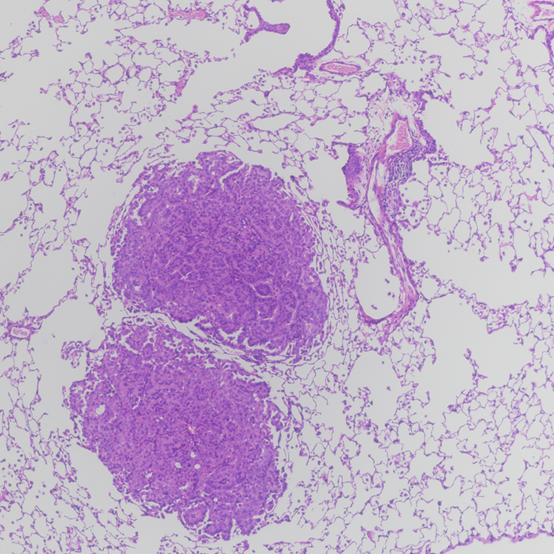

Supplement: Supplementary file 14 — Source data Fig. 6 [file 44321_2025_200_MOESM14_ESM.zip › EMM-2024-20400_SourceDataForFigure 6/Figure 6J/DT(IR-EGFR-DEL+FOXP3-DTR-GFP BM).tif]

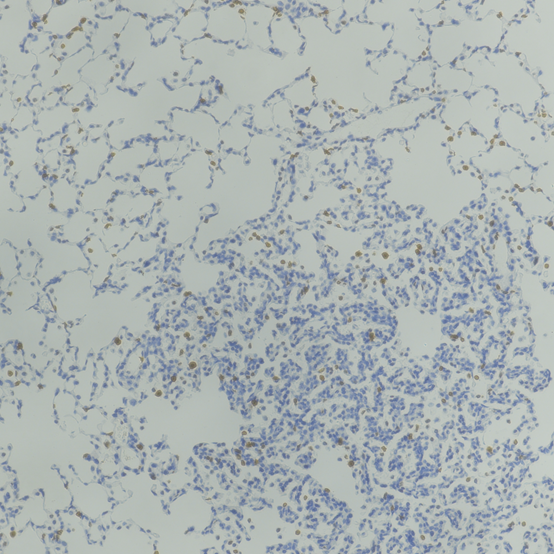

Supplement: Supplementary file 14 — Source data Fig. 6 [file 44321_2025_200_MOESM14_ESM.zip › EMM-2024-20400_SourceDataForFigure 6/Figure 6J/DT(IR-EGFR-DEL+FOXP3-DTR-GFP BM)ki67.tif]

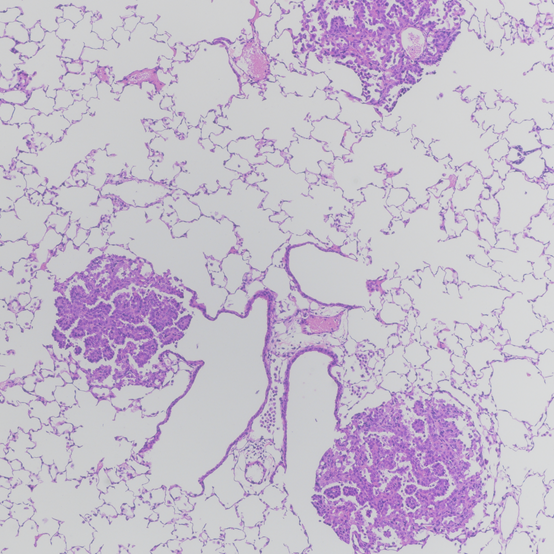

Supplement: Supplementary file 14 — Source data Fig. 6 [file 44321_2025_200_MOESM14_ESM.zip › EMM-2024-20400_SourceDataForFigure 6/Figure 6J/Lac(EGFR-DEL).tif]

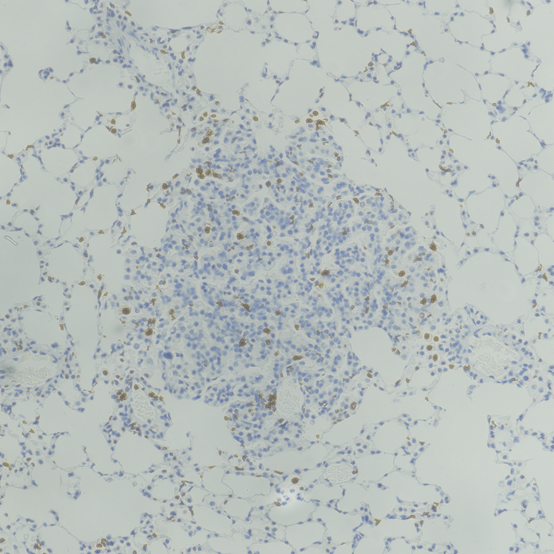

Supplement: Supplementary file 14 — Source data Fig. 6 [file 44321_2025_200_MOESM14_ESM.zip › EMM-2024-20400_SourceDataForFigure 6/Figure 6J/Lac(EGFR-DEL)ki67.tif]

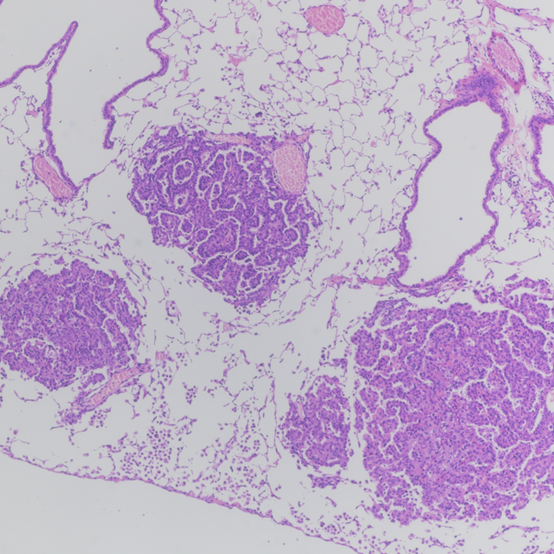

Supplement: Supplementary file 14 — Source data Fig. 6 [file 44321_2025_200_MOESM14_ESM.zip › EMM-2024-20400_SourceDataForFigure 6/Figure 6J/Lac(IR-EGFR-DEL+FOXP3-DTR-GFP BM).tif]

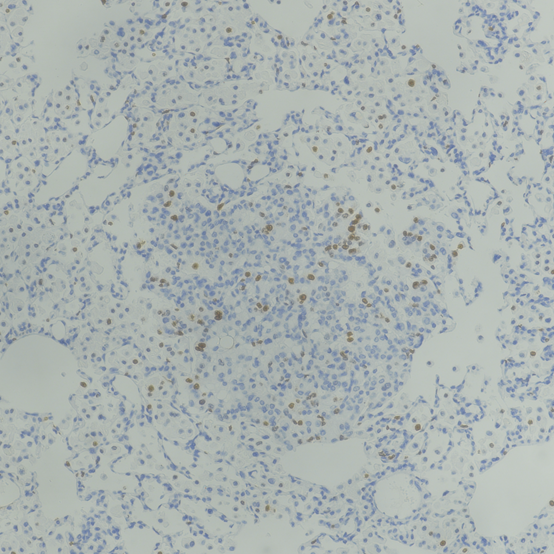

Supplement: Supplementary file 14 — Source data Fig. 6 [file 44321_2025_200_MOESM14_ESM.zip › EMM-2024-20400_SourceDataForFigure 6/Figure 6J/Lac(IR-EGFR-DEL+FOXP3-DTR-GFP BM)ki67.tif]

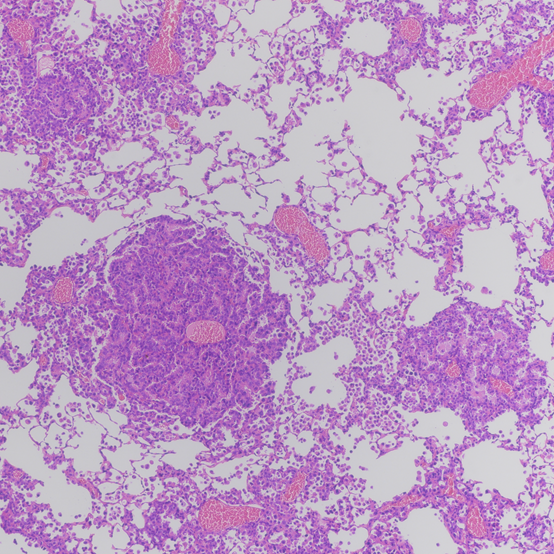

Supplement: Supplementary file 14 — Source data Fig. 6 [file 44321_2025_200_MOESM14_ESM.zip › EMM-2024-20400_SourceDataForFigure 6/Figure 6J/Veh(EGFR-DEL).tif]

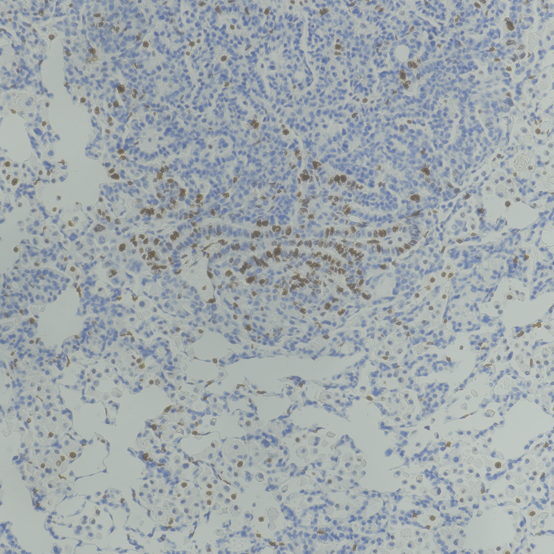

Supplement: Supplementary file 14 — Source data Fig. 6 [file 44321_2025_200_MOESM14_ESM.zip › EMM-2024-20400_SourceDataForFigure 6/Figure 6J/Veh(EGFR-DEL)ki67.tif]

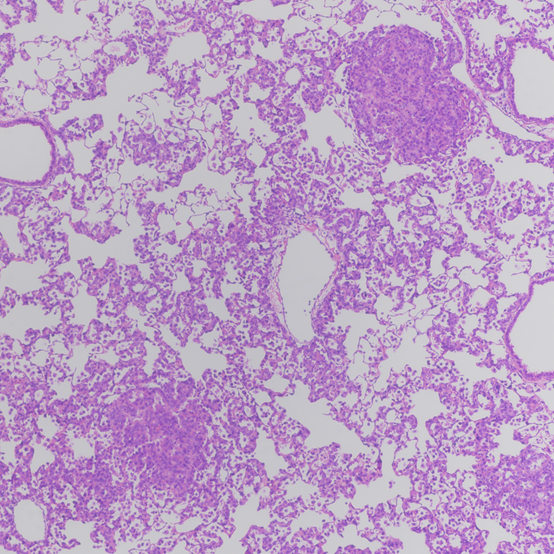

Supplement: Supplementary file 14 — Source data Fig. 6 [file 44321_2025_200_MOESM14_ESM.zip › EMM-2024-20400_SourceDataForFigure 6/Figure 6J/Veh(IR-EGFR-DEL+FOXP3-DTR-GFP BM).tif]

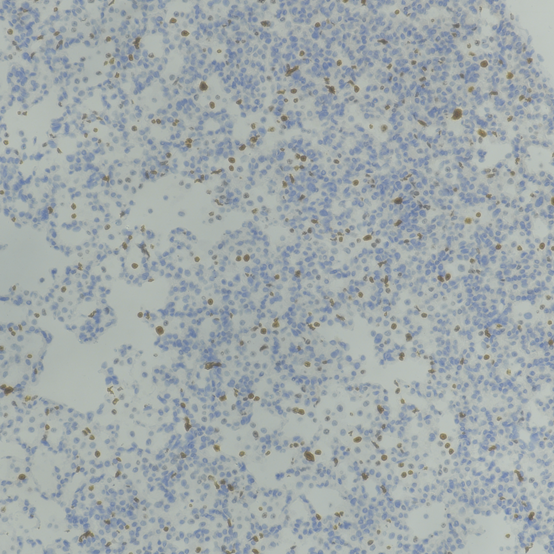

Supplement: Supplementary file 14 — Source data Fig. 6 [file 44321_2025_200_MOESM14_ESM.zip › EMM-2024-20400_SourceDataForFigure 6/Figure 6J/Veh(IR-EGFR-DEL+FOXP3-DTR-GFP BM)ki67.tif]
