# Supplementary material for: Lanatoside C activates the E3 ligase STUB1 to inhibit FOXP3 transcriptional activity and promote antitumor immunity
Source: EMBO Mol Med. 2025 Feb 20;17(3):563–88. doi: 10.1038/s44321-025-00200-y (PMC11904033; doi:10.1038/s44321-025-00200-y)
Supplement: Supplementary file 16 — Expanded View Figures [file 44321_2025_200_MOESM16_ESM.pdf]

Expanded View Figures

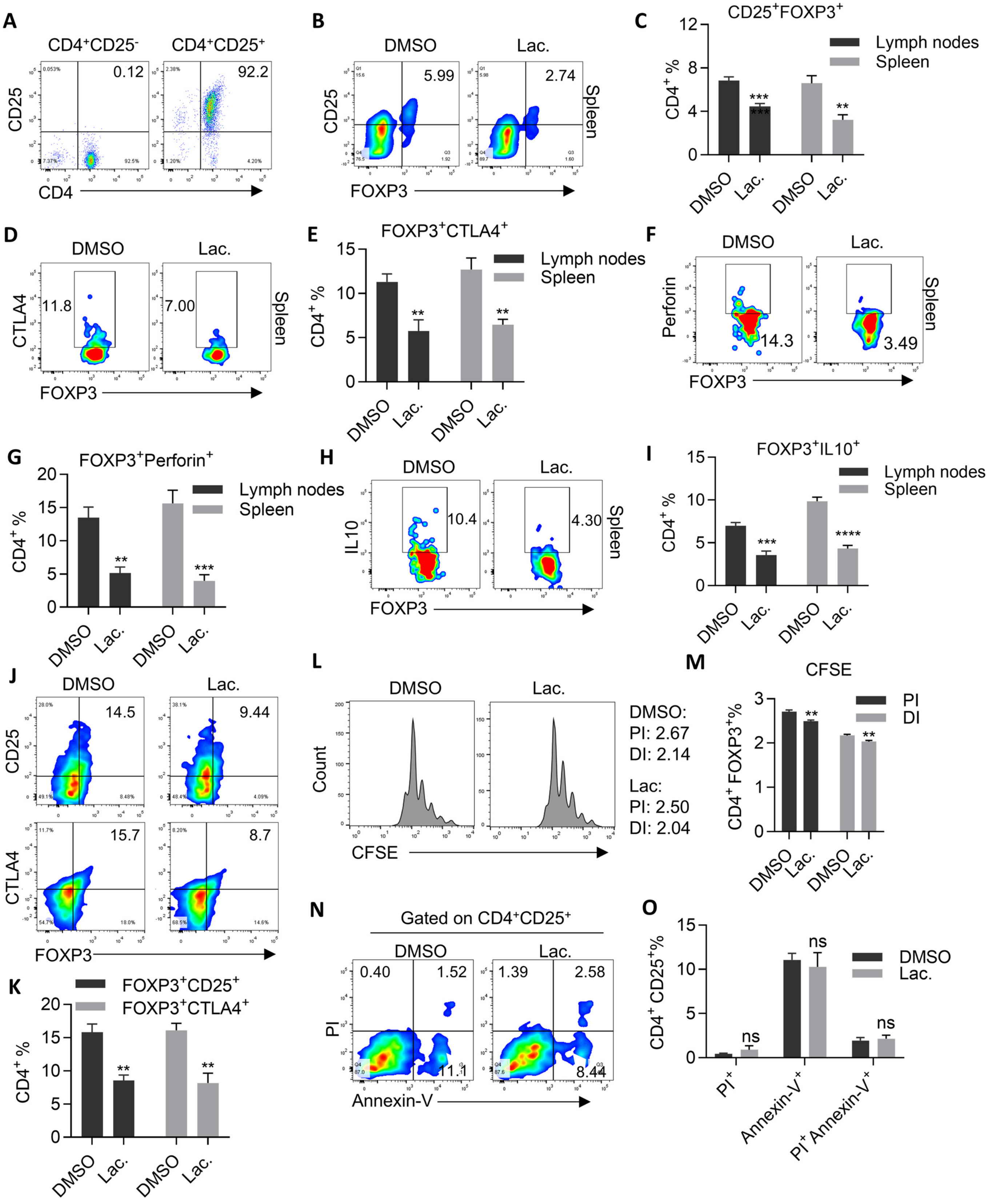

Figure EV1. Impact of Lac on Tregs.

(**A**) Tregs-purifying efficiency from mouse lymph nodes. Conventional $CD4^+$ T ($CD4^+CD25^-$) or Treg ($CD4^+CD25^+$) cells were purified from lymph nodes by negative selection with MACS before flow cytometry assay. (**B–I**) Flow cytometry analysis of the impact of Lac on marker protein expression by Tregs. The gating of $FOXP3^+CD25^+$ (**B**), $FOXP3^+CTLA4^+$ (**D**), $FOXP3^+Perforin^+$ (**F**) and $IL10^+$ (**H**) populations were determined against their FMO; Statistics analysis of Fig. 1D and EV1B (EV1C), Fig. 1E and EV1D (EV1E), Fig. 1F and EV1F (EV1G), Fig. 1G and EV1H (EV1I). Data are representative of three independent experiments and were analyzed by Student's *t* test. Error bars denote mean ± SD. *P* value: $FOXP3^+CD25^+$ (Lymph nodes), $***P = 0.0006$; $FOXP3^+CD25^+$ (Spleen), $**P = 0.0024$; $FOXP3^+CTLA4^+$ (Lymph nodes), $**P = 0.0035$; $FOXP3^+CTLA4^+$ (Spleen), $**P = 0.0017$; $FOXP3^+Perforin^+$ (Lymph nodes), $**P = 0.0014$; $FOXP3^+Perforin^+$ (Spleen), $***P = 0.0008$; $FOXP3^+IL10^+$ (Lymph nodes), $***P = 0.0006$; $FOXP3^+IL10^+$ (Spleen), $****P < 0.0001$. (**J**) Lac suppresses Tregs differentiation in vitro. Representative flow plots were gated on $CD4^+$ population for analysis. The $FOXP3^+CD25^+$ (upper panel) or $FOXP3^+CTLA4^+$ (lower panel) populations were determined against their FMO, respectively. (**K**) Statistics analysis of Fig. EV1J. Data are representative of three independent experiments and were analyzed by Student's *t* test. Error bars denote mean ± SD. *P* value: $FOXP3^+CD25^+$, $**P = 0.0010$; $FOXP3^+CTLA4^+$, $**P = 0.0016$. (**L**) Lac suppresses the proliferation of Tregs in vitro. Proliferation of Tregs were determined by dilution of CFSE through flow cytometry analysis. (**M**) Statistics of the percentages of proliferating Tregs of Fig. EV1L. Data are representative of three independent experiments and were analyzed by Student's *t* test. Error bars denote mean ± SD. *P* value: PI, $**P = 0.0014$; DI, $**P = 0.0041$. (**N**) Impact of Lac on apoptosis of Tregs. Cells were stained with PI and Annexin V-FITC for flow cytometry analysis. (**O**) Statistics of apoptosis population of Fig. EV1N. Data are representative of three independent experiments and were analyzed by Student's *t* test. Error bars denote mean ± SD. *P* value: PI^+, $P = 0.1653$; Annexin V^+, $P = 0.4850$; Annexin V^+PI^+, $P = 0.4890$. Source data are available online for this figure.

© The Author(s)

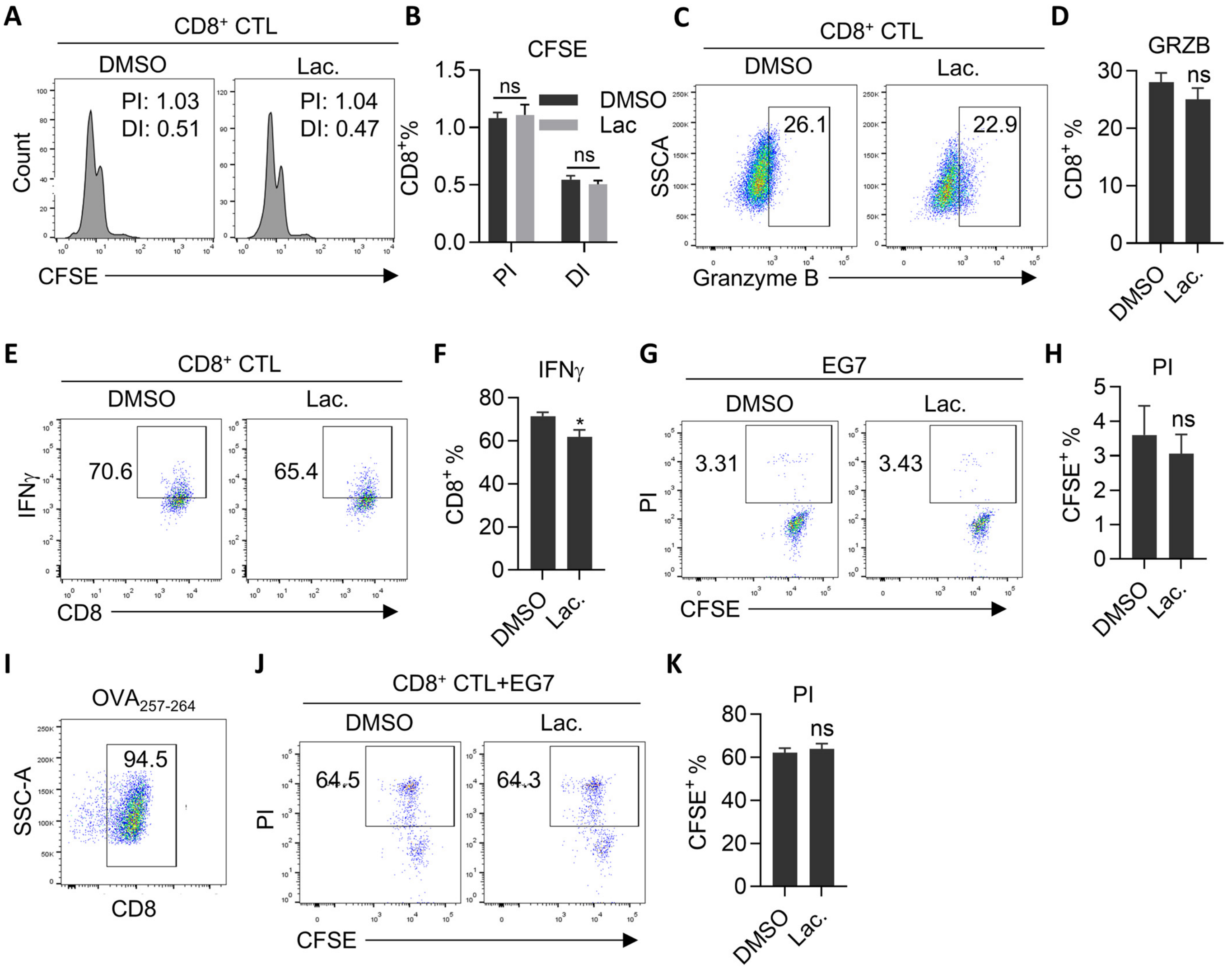

Figure EV2. Impact of Lac on the phenotype of CD8+ T cells.

(**A**) Lac exhibits limited impact on the proliferation of CD8+ T cells. Proliferation of OT-I cells were determined by dilution of CFSE through flow cytometry analysis on CD8a positive population. (**B**) Statistics of percentages of proliferating OT-I CTLs of Fig. EV2A. Data are representative of three independent experiments and were analyzed by Student's *t* test. Error bars denote mean ± SD. *P* value: PI, $P = 0.6702$; DI, $P = 0.2193$. (**C–F**) The mature CTLs and EG7 cells (CTLs:EG7 = 5:2) were co-cultured in the presence of DMSO or Lac (2 μM) for 6 h, followed by incubation with monensin for another 6 h. The cells were then stained with anti-CD8a and GRZB (EV2C) or anti-CD8a and IFNγ (EV2E) for flow cytometry analysis. Statistics of GRZB (EV2D) and IFNγ (EV2F) expression. Data are representative of three independent experiments and were analyzed by Student's *t* test. Error bars denote mean ± SD. *P* value: GRZB, $P = 0.1202$; IFNγ, $^{*}P = 0.0114$. (**G**) Lac exhibits no toxicity to EG7 cells. Toxicity of EG7 cells was determined by analyzing PI/CFSE double-positive population. (**H**) Statistics of the percentages of CFSE+PI+ EG7 cells of Fig. EV2G. Data are representative of three independent experiments and were analyzed by Student's *t* test. Error bars denote mean ± SD. *P* value: $P = 0.4103$. (**I**) Purity of $OVA_{257-264}$ peptide-induced splenic OT-I CTLs. (**J**) Impact of Lac treatment on cytotoxicity of CTLs. (**K**) Statistics of the percentages of PI/CFSE double-positive EG7 cells of Fig. EV2J. Data are representative of three independent experiments and were analyzed by Student's *t* test. Error bars denote mean ± SD. *P* value: $P = 0.3614$. Source data are available online for this figure.

© The Author(s)

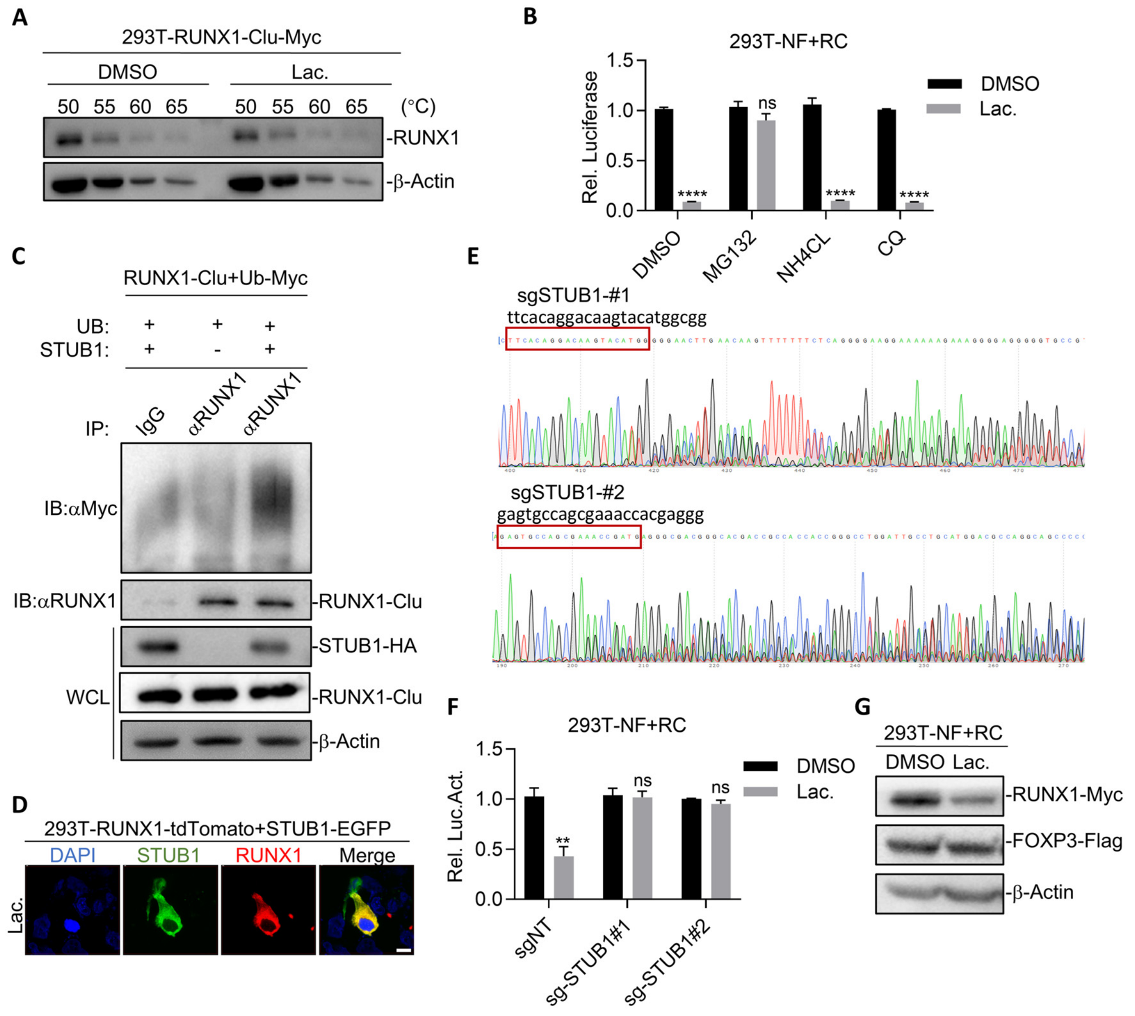

Figure EV3. STUB1 is required for Lac-induced RUNX1 degradation through proteasome pathway.

(**A**) Lac doesn't change the thermal stability of ectopically expressed RUNX1. (**B**) MG132, but not NH4Cl or CQ, stabilizes luciferase activity in response to Lac treatment in 293T-NF + RC cells. Data are representative of three independent experiments and were analyzed by Student's *t* test. Error bars denote mean ± SD. *P* value: DMSO, ****$P < 0.0001$; MG132, $P = 0.0537$; NH4CL, ****$P < 0.0001$; CQ, ****$P < 0.0001$. (**C**) STUB1-mediated induction of ubiquitination of RUNX1. (**D**) Lac promotes STUB1-mediated redistribution of RUNX1. Scale bar: 10 μm. (**E**) Sequence chromatograms of 293T-sgSTUB1 cells. (**F**) STUB1 knockout stabilizes the luciferase activity of 293T-NF + RC in response to Lac treatment in bimolecular fluorescence complementation assay. Data are representative of three independent experiments and were analyzed by Student's *t* test. Error bars denote mean ± SD. *P* value: sgNT, **$P = 0.0013$; sgSTUB1#1, $P = 0.7501$; sgSTUB1#2, $P = 0.0811$. (**G**) Lac promotes the degradation of RUNX1 but not FOXP3 in 293T-NF + RC cells. Source data are available online for this figure.

 © The Author(s)

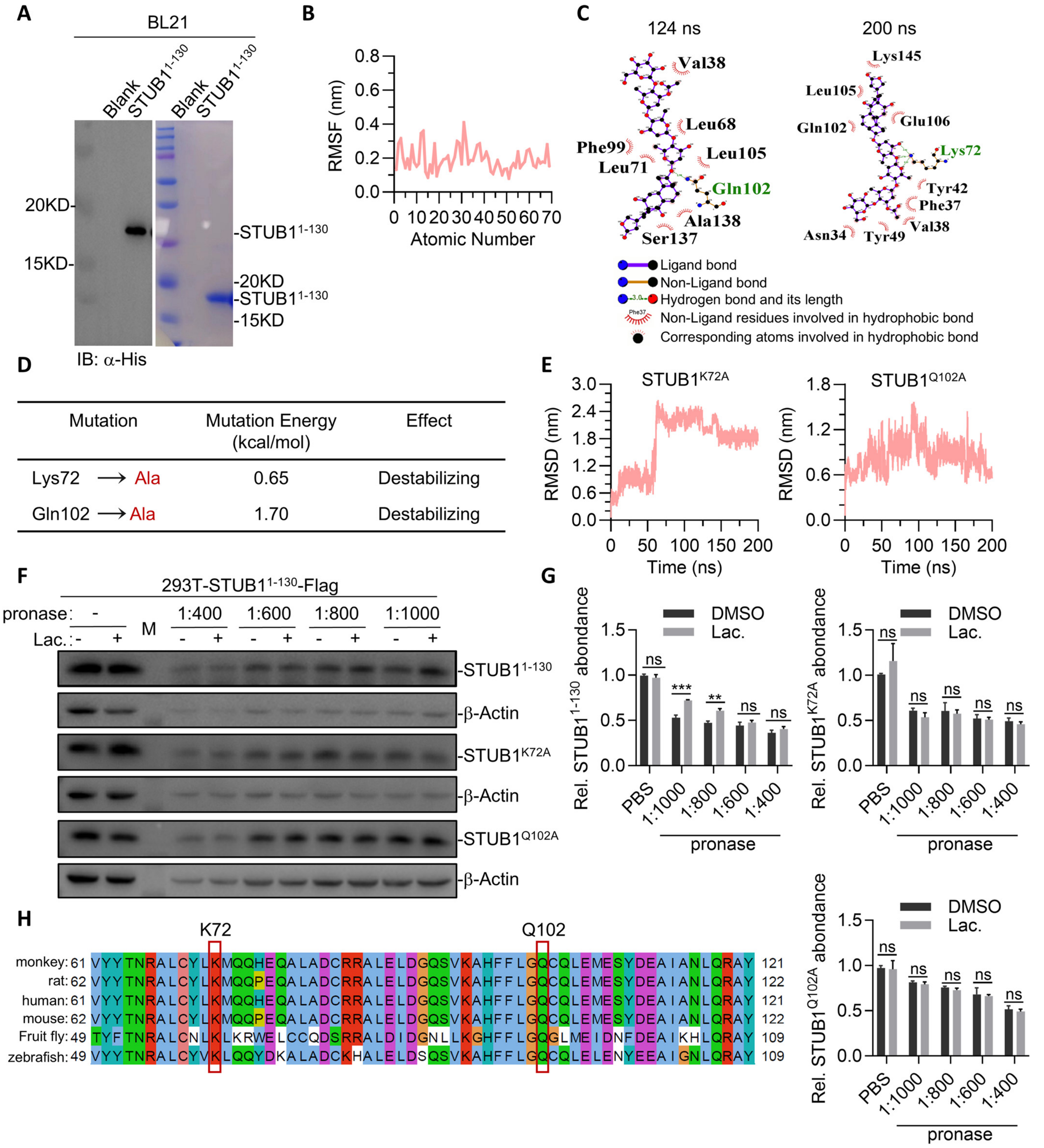
A
BL21
Blank
STUB1^1-130
Blank
STUB1^1-130
20KD-
15KD-
-STUB1^1-130
-20KD
-STUB1^1-130
-15KD
IB: α-His
B
RMSF (nm)
Atomic Number
C
124 ns
200 ns
Val38
Leu68
Phe99
Leu105
Leu71
Gln102
Ala138
Ser137
Lys145
Leu105
Gln102
Glu106
Lys72
Tyr42
Phe37
Val38
Asn34
Tyr49
Ligand bond
Non-Ligand bond
Hydrogen bond and its length
Non-Ligand residues involved in hydrophobic bond
Corresponding atoms involved in hydrophobic bond
D
Mutation | Mutation Energy (kcal/mol) | Effect
Lys72 ⟶ Ala | 0.65 | Destabilizing
Gln102 ⟶ Ala | 1.70 | Destabilizing
E
STUB1^K72A
STUB1^Q102A
RMSD (nm)
Time (ns)
F
293T-STUB1^1-130-Flag
pronase: - 1:400 1:600 1:800 1:1000
Lac.: - + M
-STUB1^1-130
-β-Actin
-STUB1^K72A
-β-Actin
-STUB1^Q102A
-β-Actin
G
Rel. STUB1^1-130 abondance
Rel. STUB1^K72A abondance
Rel. STUB1^Q102A abondance
DMSO
Lac.
ns
***
**
PBS
1:1000
1:800
1:600
1:400
pronase
H
K72
Q102
monkey: 61 VYYTNRALCYLKMQQHEQALADCRRALELDGQSVKAHFFLGQCQLEMESYDEAIANLQRAY 121
rat: 62 VYYTNRALCYLKMQQPEQALADCRRALELDGQSVKAHFFLGQCQLEMESYDEAIANLQRAY 122
human: 61 VYYTNRALCYLKMQQHEQALADCRRALELDGQSVKAHFFLGQCQLEMESYDEAIANLQRAY 121
mouse: 62 VYYTNRALCYLKMQQPEQALADCRRALELDGQSVKAHFFLGQCQLEMESYDEAIANLQRAY 122
Fruit fly: 49 TYFTNRALCNLKLKRWELCCQDSRRALDIDGNLLKGHFFLGQGLMEIDNFDEAIKHLQRAY 109
zebrafish: 49 VYYTNRALCYVKLQQYDKALADCKHALELDSQSVKAHFFLGQCQLELENYEEAIGNLQRAY 109

Figure EV4. Lac directly binds STUB1 at its TPR domain to enhance its affinity to RUNX1.

(**A**) IB and coomassie brilliant blue staining of purified STUB1$^{1\text{-}130}$. IB (left panel) and SDS-PAGE (right panel) analysis of purified STUB1$^{1\text{-}130}$ protein. (**B**) The root-mean-square deviations (RMSF) plot for docked Lac during MD simulations. (**C**) Ligplot of 2-D interaction of Lac-STUB1 at 124 ns and 200 ns in the MD simulations (The red arcs for hydrophobic interactions; green lines for hydrogen bonds). (**D**) Free energies predicted for bindings of Lac-STUB1^{K72A} (Lys72 to Ala) and Lac-STUB1^{Q102A} (Gln102 to Ala) with Lac. (**E**) Time-dependent RMSD plot of docked Lac in single-point mutants of STUB1 (K72A and Q102A) during molecular dynamics simulation. (**F**) Lac fails to protect ectopically expressed mutant STUB1 from pronase digestion through DARTS assay. (**G**) Statistics of relative abundance of mutant STUB1 of (**F**). Data are representative of three independent experiments and were analyzed by Student's *t* test. Error bars denote mean ± SD. *P* value: PBS, $P = 0.3196$, 1: 1000, $^{***}P = 0.0005$, 1: 800, $^{**}P = 0.0021$, 1: 600, $P = 0.2729$, 1: 400, $P = 0.1308$ for STUB1$^{1\text{-}130}$; PBS, $P = 0.2618$, 1: 1000, $P = 0.0892$, 1: 800, $P = 0.6736$, 1: 600, $P = 0.5848$, 1: 400, $P = 0.2594$ for STUB1$^{127\text{-}226}$; PBS, $P = 0.8264$, 1: 1000, $P = 0.3046$, 1: 800, $P = 0.1404$, 1: 600, $P = 0.7153$, 1: 400, $P = 0.4499$ for STUB1$^{223\text{-}303}$. (**H**) Sequence homology of STUB1 TPR domain. Alignment of STUB1 sequences from human (H. sapiens, 005852.2), zebrafish (D. rerio, 021325787.1), mouse (M. musculus, 062693.1), rat (R. norvegicus, 001020796.2), monkey (M. mulatta, 001244487.1), Fruit fly (D. melanogaster, 477441.1). Source data are available online for this figure.

 © The Author(s)

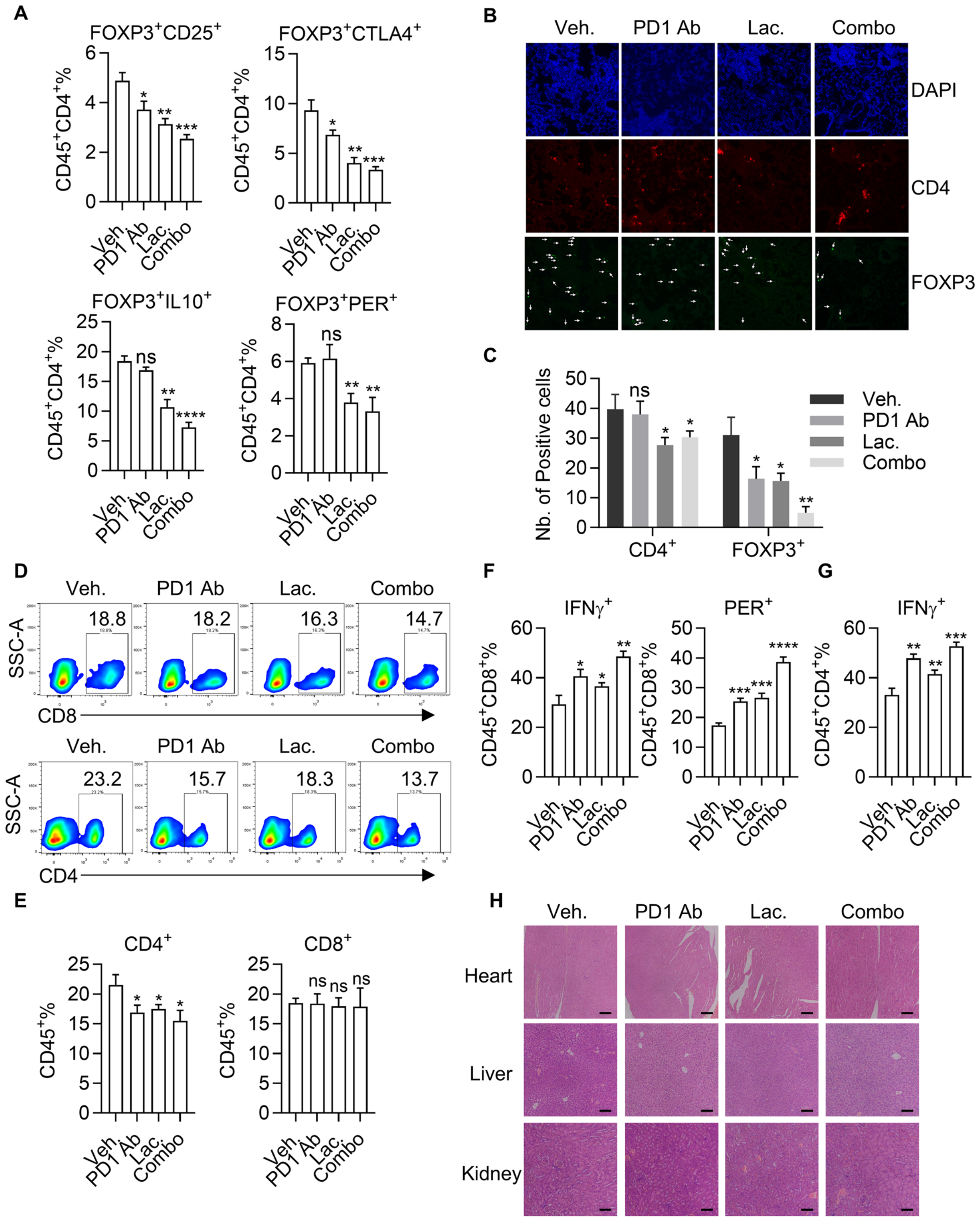
A
FOXP3+CD25+
FOXP3+CTLA4+
FOXP3+IL10+
FOXP3+PER+
CD45+CD4+%
Veh.
PD1 Ab
Lac.
Combo
ns
B
Veh.
PD1 Ab
Lac.
Combo
DAPI
CD4
FOXP3
C
Nb. of Positive cells
CD4+
FOXP3+
D
SSC-A
CD8
18.8
18.2
16.3
14.7
CD4
23.2
15.7
18.3
13.7
E
CD45+%
CD4+
CD8+
F
IFNγ+
PER+
CD45+CD8+%
G
CD45+CD4+%
H
Heart
Liver
Kidney

Figure EV5. Lac synergizes with PD-1 inhibitor to treat mutant KRAS-driven lung cancer.

(**A**) Statistics of the proportion of $FOXP3^+CD25^+$, $FOXP3^+CTLA4^+$, $FOXP3^+IL10^+$ and $FOXP3^+PER^+$ Tregs in tumor of Fig. 7G, H. Data are representative of three independent experiments and were analyzed by Student's *t* test. Error bars denote mean ± SD. *P* value: Veh. vs PD-1 Ab, $^*P = 0.0127$, Veh. vs Lac, $^{**}P = 0.0015$, Veh. vs Combo, $^{***}P = 0.0004$ for $FOXP3^+CD25^+$; Veh. vs PD-1 Ab, $^*P = 0.0218$, Veh. vs Lac, $^{**}P = 0.0016$, Veh. vs Combo, $^{***}P = 0.0007$ for $FOXP3^+CTLA4^+$; Veh. vs PD-1 Ab, $P = 0.0624$, Veh. vs Lac, $^{**}P = 0.0010$, Veh. vs Combo, $^{****}P < 0.0001$ for $FOXP3^+IL10^+$; Veh. vs PD-1 Ab, $P = 0.6317$, Veh. vs Lac, $^{**}P = 0.0027$, Veh. vs Combo, $^{**}P = 0.0049$ for $FOXP3^+PER^+$. (**B**) Immunofluorescence analysis of Tregs infiltration. Scale bar: 100 μm. (**C**) Statistics of the Tregs infiltration ($CD4^+$; $FOXP3^+$) in tumor of Fig. EV5B. Data are representative of three independent experiments and were analyzed by Student's *t* test. Error bars denote mean ± SD. *P* value: Veh. vs PD-1 Ab, $P = 0.6870$, Veh. vs Lac, $^*P = 0.0210$, Veh. vs Combo, $^*P = 0.0412$ for $CD4^+$; Veh. vs PD-1 Ab, $^*P = 0.0246$, Veh. vs Lac, $^*P = 0.0151$, Veh. vs Combo, $^{**}P = 0.0021$ for $FOXP3^+$. (**D**) Lac or PD-1 antibody treatment did not significantly alter the infiltration of $CD4^+$ and $CD8^+$ T cells into tumors. Representative flow plots were gated on $CD45^+$ population for analysis. (**E**) Statistics of the proportion of $CD8^+$ (right panel) and $CD4^+$ (left panel) in tumor of Fig. EV5D. Data are representative of three independent experiments and were analyzed by Student's *t* test. Error bars denote mean ± SD. *P* value: Veh. vs PD-1 Ab, $^*P = 0.0191$, Veh. vs Lac, $^*P = 0.0195$, Veh. vs Combo, $^*P = 0.0129$ for $CD4^+$; Veh. vs PD-1 Ab, $P = 0.9277$, Veh. vs Lac, $P = 0.5793$, Veh. vs Combo, $P = 0.7526$ for $CD8^+$. (**F**) Statistics of the proportion of $CD8^+IFN\gamma^+$ (left panel) and $CD8^+PER^+$ (right panel) in tumor of Fig. 7I. Data are representative of three independent experiments and were analyzed by Student's *t* test. Error bars denote mean ± SD. *P* value: Veh. vs PD-1 Ab, $^*P = 0.0124$, Veh. vs Lac, $^*P = 0.0318$, Veh. vs Combo, $^{**}P = 0.0013$ for $IFN\gamma^+$; Veh. vs PD-1 Ab, $^{***}P = 0.0005$, Veh. vs Lac, $^{***}P = 0.0007$, Veh. vs Combo, $^{****}P < 0.0001$ for PER^+. (**G**): Statistics of the proportion of $CD4^+IFN\gamma^+$ in tumor of Fig. 7J. Data are representative of three independent experiments and were analyzed by Student's *t* test. Error bars denote mean ± SD. *P* value: Veh. vs PD-1 Ab, $^{**}P = 0.0013$, Veh. vs Lac, $^{**}P = 0.0090$, Veh. vs Combo, $^{***}P = 0.0004$ for $IFN\gamma^+$. (**H**) Representative images of H&E staining of the indicated organs of treated mice. Scale bar: 100 μm. Source data are available online for this figure.

 © The Author(s)
